# Identification of asporin as a HER3 ligand exposes a therapeutic vulnerability in prostate cancer

**DOI:** 10.1172/jci.insight.187151

**Published:** 2025-08-22

**Authors:** Amanda B. Hesterberg, Hong Yuen Wong, Jorgen Jackson, Monika Antunovic, Brenda L. Rios, Evan Watkins, Riley E. Bergman, Brad A. Davidson, Sarah E. Ginther, Diana Graves, Elliott F. Nahmias, Jared A. Googel, Lillian B. Martin, Violeta Sanchez, Paula I. Gonzalez-Ericsson, Quanhu Sheng, Benjamin P. Brown, Jens Meiler, Kerry R. Schaffer, Jennifer B. Gordetsky, Ben H. Park, Paula J. Hurley

**Affiliations:** 1 Department of Medicine,; 2 Department of Pathology, Microbiology and Immunology, and; 3 Department of Biostatistics, Vanderbilt University Medical Center, Nashville, Tennessee, USA.; 4 Department of Chemistry,; 5 Vanderbilt Institute of Chemical Biology, and; 6 Center for Structural Biology, Vanderbilt University, Nashville, Tennessee, USA.; 7 Leipzig University Medical School, Institute for Drug Discovery, Leipzig, Germany.; 8 Vanderbilt-Ingram Cancer Center and; 9 Department of Urology, Vanderbilt University Medical Center, Nashville, Tennessee, USA.

**Keywords:** Cell biology, Oncology, Oncogenes, Prostate cancer, Signal transduction

## Abstract

Cancer-associated fibroblasts (CAFs) are part of the tumor microenvironment (TME) that enable cancer cells to establish metastases, but the mechanisms of these interactions are not fully known. Herein, we identified a paracrine mechanism in which CAF-secreted asporin (ASPN) activated ErbB signaling and subsequent migration of adjacent prostate cancer cells. Our data support that ASPN bound directly to the ligand binding domain of human epidermal growth factor 3 (HER3) and induced HER2/HER3 heterodimerization and activation of the PI3K, MAPK, and calcium pathways. Genetic and therapeutic inhibition of HER2/HER3 ablated ASPN-induced signaling and migration. Clinically, *ASPN* was detected in the stroma of HER2/HER3-expressing human metastatic prostate cancer, supporting the clinical relevance of these findings and highlighting a potential therapeutic vulnerability. Antibody-drug conjugate (ADC) therapies designed to target HER2 (trastuzumab-deruxtecan) or HER3 (patritumab-deruxtecan) significantly diminished prostate cancer cell growth in vitro and tumor size in vivo, despite *Aspn* in the TME. Collectively, these findings indicate ASPN functions as a HER3 ligand to induce cellular migration, and inhibition with anti-HER2 or anti-HER3 ADC therapies highlights potential clinical utility for patients with metastatic castration-resistant prostate cancer that expresses HER2 or HER3.

## Abstract 2

Stroma-secreted ASPN is a HER3 ligand, and ASPN-induced HER2/HER3-signaling and migration in prostate cancer cells can be inhibited with HER2/HER3 targeted therapies.

## Introduction

Rates of prostate cancer incidence and advanced-stage diagnoses are increasing (1, 2). Androgen deprivation therapy (ADT) in combination with novel hormonal therapies, including apalutamide, darolutamide, enzalutamide, and abiraterone, remains the current standard of care for patients with metastatic prostate cancer, sometimes with the addition of chemotherapy (3, 4). Despite initial responses, nearly all patients with advanced disease progress to metastatic castration-resistant prostate cancer (mCRPC), a lethal form of the disease. There is an urgent unmet need to understand the mechanisms driving prostate cancer progression and to identify alternative therapies for patients living with mCRPC.

It is well established that the ErbB family of receptors, including EGFR, human epidermal growth factor 2 (HER2), HER3, and HER4, stimulate tumor progression in many cancer types (5–10). Although genomic alterations in *ErbB* genes are uncommon in prostate cancer, emerging evidence suggests ErbB signaling serves an important role in advanced-stage disease, and targeting these receptors may have therapeutic potential (11–16). Ligand activation of ErbB signaling occurs by autocrine signaling within cancer cells or by paracrine interactions with the tumor microenvironment (TME). A major paracrine mechanism by which cancer cells interact with the TME is through intercellular crosstalk with cancer-associated fibroblasts (CAFs) (17, 18). CAFs are an abundant and heterogeneous population of cells with pleiotropic roles in cancer, and interactions between CAFs and cancer cells alter multiple tumor-related factors, including angiogenesis, extracellular matrix deposition, immune cell infiltration, migration, and therapy resistance (19, 20). A prior report demonstrated that neoadjuvant ADT induced prostate CAF secretion of the HER3 ligand, neuregulin 1β (NRG1β), in nearly a fifth of localized prostate cancers and increased resistance to antiandrogens through HER3 signaling in adjacent cancer cells (21). Identification of additional CAF-secreted factors that stimulate prostate cancer progression may reveal new therapeutic targets for patients with advanced-stage disease.

Asporin (ASPN) is a key developmental mesenchymal stromal gene that is reactivated in prostate CAFs and secreted as an extracellular protein into the TME (22–27). Elevated ASPN expression in the TME of localized prostate cancer is associated with increased grade and worse oncologic outcomes, including metastasis (23, 28–30). Patients with advanced prostate cancer have detectable levels of ASPN in their blood (31) as well as elevated stromal ASPN expression at metastatic sites (23). Functional studies have shown that secreted ASPN alters prostate cancer cell phenotypes by increasing cell migration and metastatic potential as demonstrated in both the B6CaP and PC3 models (23, 28, 32). While ASPN has been shown to regulate cancer cell migration through EGFR (33), TGF-β (34), and CD44 (35) in other cancer types, the mechanisms by which ASPN mediates prostate cancer migration are not fully known.

In this study, we sought to identify the molecular interactions between cancer-associated stromal ASPN and prostate cancer cells. We demonstrate that ASPN is a ligand of HER3 and induces HER3 heterodimerization with its preferred dimerization partner, HER2 (36). ASPN activates established ErbB-associated pathways, including PI3K, MAPK, and calcium signaling, in multiple prostate cancer cell lines to promote cell migration. Genetic and molecular inhibition of HER2/HER3 mitigates ASPN-induced signaling and cell migration, suggesting these receptors are required for paracrine activation by ASPN. Stromal expression of *ASPN* in the TME of HER2/HER3-expressing metastatic prostate cancers is frequently observed in patient samples, supporting the clinical relevance of these findings and highlighting a potential therapeutic vulnerability. Importantly, antibody-drug conjugate (ADC) therapies designed to target HER2 or HER3 significantly diminish prostate cancer cell growth in vitro and tumor size in vivo, despite ASPN in the TME. Together, these results identify ASPN as a HER3 ligand and suggest potential clinical utility of anti-HER2 or anti-HER3 ADCs for the treatment of metastatic prostate cancer.

## Results

### Stroma-secreted ASPN activates HER2/HER3 in prostate cancer cells.

**Figure 1:**
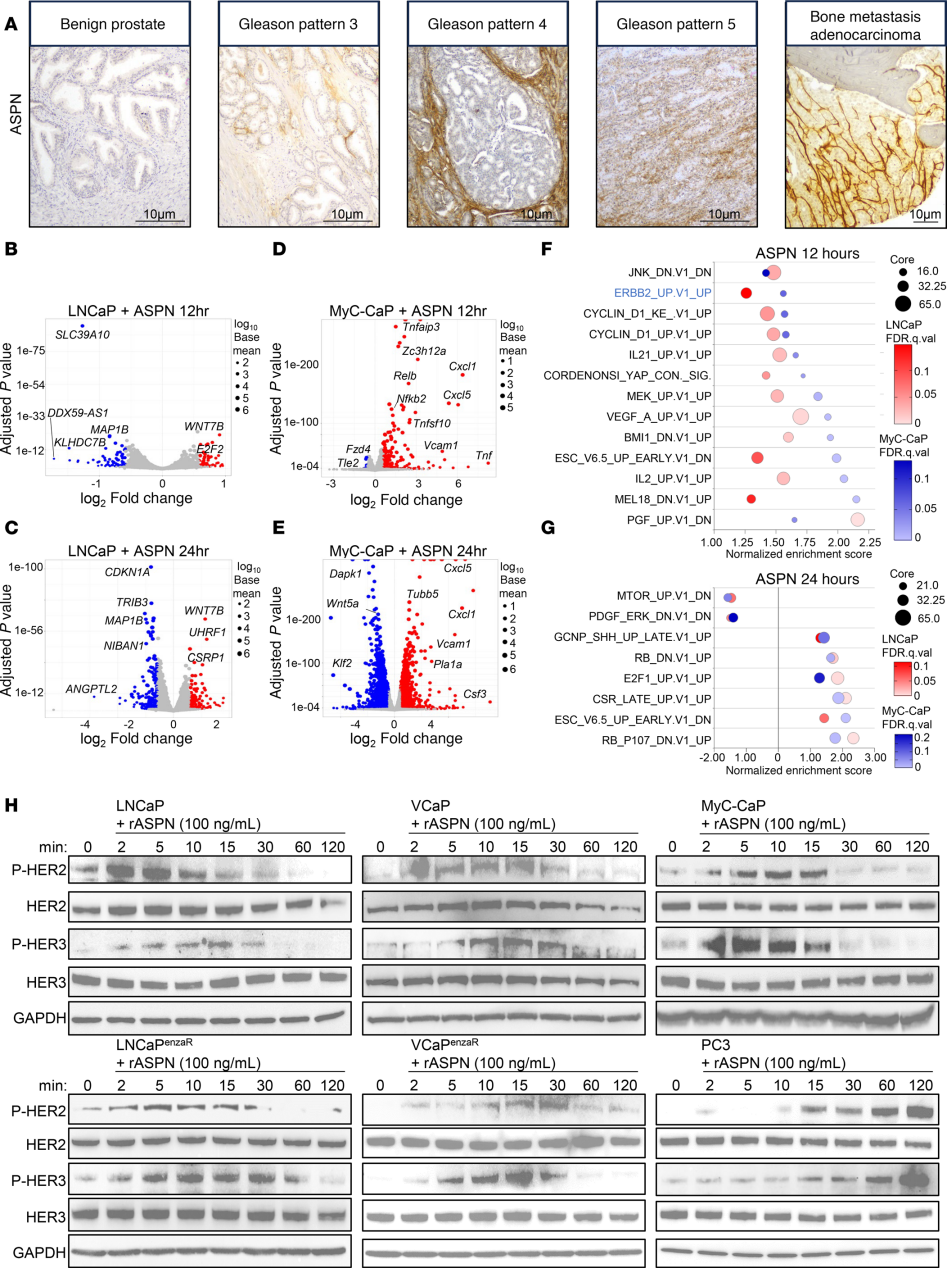
Stroma-secreted ASPN activates HER2/HER3 in prostate cancer cells. (**A**) Representative images of ASPN expression in the tumor microenvironment by IHC in benign prostate, localized prostate cancer of Gleason patterns 3–5, and metastatic prostate cancer. (**B** and **C**) Volcano plots of LNCaP cells treated with 100 ng/mL recombinant human ASPN for 12 (**B**) and 24 (**C**) hours compared with time 0. (**D** and **E**) Volcano plots of MyC-CaP cells treated with 100 ng/mL recombinant mouse ASPN for 12 (**D**) and 24 (**E**) hours compared with time 0. (**F** and **G**) Bubble plots of overlapping Oncogenic Signatures by GSEA of LNCaP and MyC-CaP cells treated with 100 ng/mL recombinant ASPN for 12 (**F**) and 24 (**G**) hours compared with time 0. (**H**) LNCaP, VCaP, MyC-CaP, LNCaP enzalutamide-resistant (LNCaP^enzaR^), VCaP enzalutamide-resistant (VCaP^enzaR^), and PC3 cells treated with 100 ng/mL recombinant human or mouse ASPN over a time course and assessed by immunoblotting for HER2 and HER3 activation (*n* = 6).

Prior studies have shown that ASPN^+^ CAFs are associated with increased Gleason pattern (24, 25, 28), worse oncologic outcomes including metastasis (28, 30), and therapy-resistant metastases (23); these findings are highlighted in representative patient samples (Figure 1A). As a secreted protein, ASPN has been shown to communicate throughout the TME to orchestrate cancer cell migration and metastatic development (23, 28, 34, 35, 37), yet the mechanisms driving these functions are not well defined. To elucidate the molecular interactions between secreted ASPN and prostate cancer cells, human (LNCaP) and mouse (MyC-CaP) prostate cancer cells were treated with recombinant human or mouse ASPN, respectively, for 0, 12, and 24 hours and then assessed by RNA sequencing (RNA-Seq). Extracellular ASPN induced widespread transcriptional changes in LNCaP and MyC-CaP (Figure 1, B–E, and Supplemental Figure 1A; supplemental material available online with this article; https://doi.org/10.1172/jci.insight.187151DS1). Comparison of overlapping Oncogenic Signatures from gene set enrichment analysis (GSEA) between ASPN-treated LNCaP and MyC-CaP showed an early enrichment of ERBB2, MEK, VEGF-A, cyclin D, YAP, and inflammatory-specific mRNA signatures at 12 hours, while signatures associated with RB1 loss and activation of E2F1 and SHH were enriched at 24 hours (Figure 1, F and G, and Supplemental Figure 1, B–F). ErbB2 (HER2) was an especially compelling candidate to assess for extracellular ASPN-induced activation as it is a cell surface receptor and member of the ErbB family that also includes EGFR, HER3, and HER4. To verify ASPN-induced HER2 pathway activation in human prostate cancer cells, androgen-responsive (LNCaP and VCaP), androgen receptor (AR) inhibitor–resistant (enzalutamide) (LNCaP^enzaR^ and VCaP^enzaR^), and androgen-insensitive (PC3, DU145) prostate cancer cells were treated with recombinant human ASPN and assessed by immunoblotting. Additionally, mouse androgen-responsive (MyC-CaP) prostate cancer cells were treated with recombinant mouse ASPN. Consistent with GSEA findings, recombinant ASPN induced activating phosphorylation of HER2 in all prostate cancer cell lines analyzed (Figure 1H and Supplemental Figure 2, A–D). ASPN also induced activating phosphorylation of HER3 (ErbB3), an ErbB family member that can heterodimerize with HER2 (36) (Figure 1H and Supplemental Figure 2, A–D). Interestingly, ASPN-induced phosphorylation of the ErbB family member EGFR at Y845 or Y1173 was undetectable to low in most prostate cancer cell lines assessed, including LNCaP, VCaP, LNCaP^enzaR^, VCaP^enzaR^, DU145, and MyC-CaP, especially in comparison with EGF-induced EGFR activation (Supplemental Figure 2, E–J). However, EGFR was phosphorylated in PC3 in response to ASPN, suggesting ASPN-induced receptor heterodimerization promiscuity exists between some cell lines (Supplemental Figure 2, C and D). The total expression of the other ErbB family member, HER4 (ErbB4), was low or undetectable in most prostate cancer cell lines assessed (Supplemental Figure 2K). Collectively, these findings support that extracellular ASPN is an activator of HER2 and HER3 in prostate cancer cells.

### ASPN-induced signaling overlaps but is distinct from NRG1β, a HER3 ligand.

**Figure 2:**
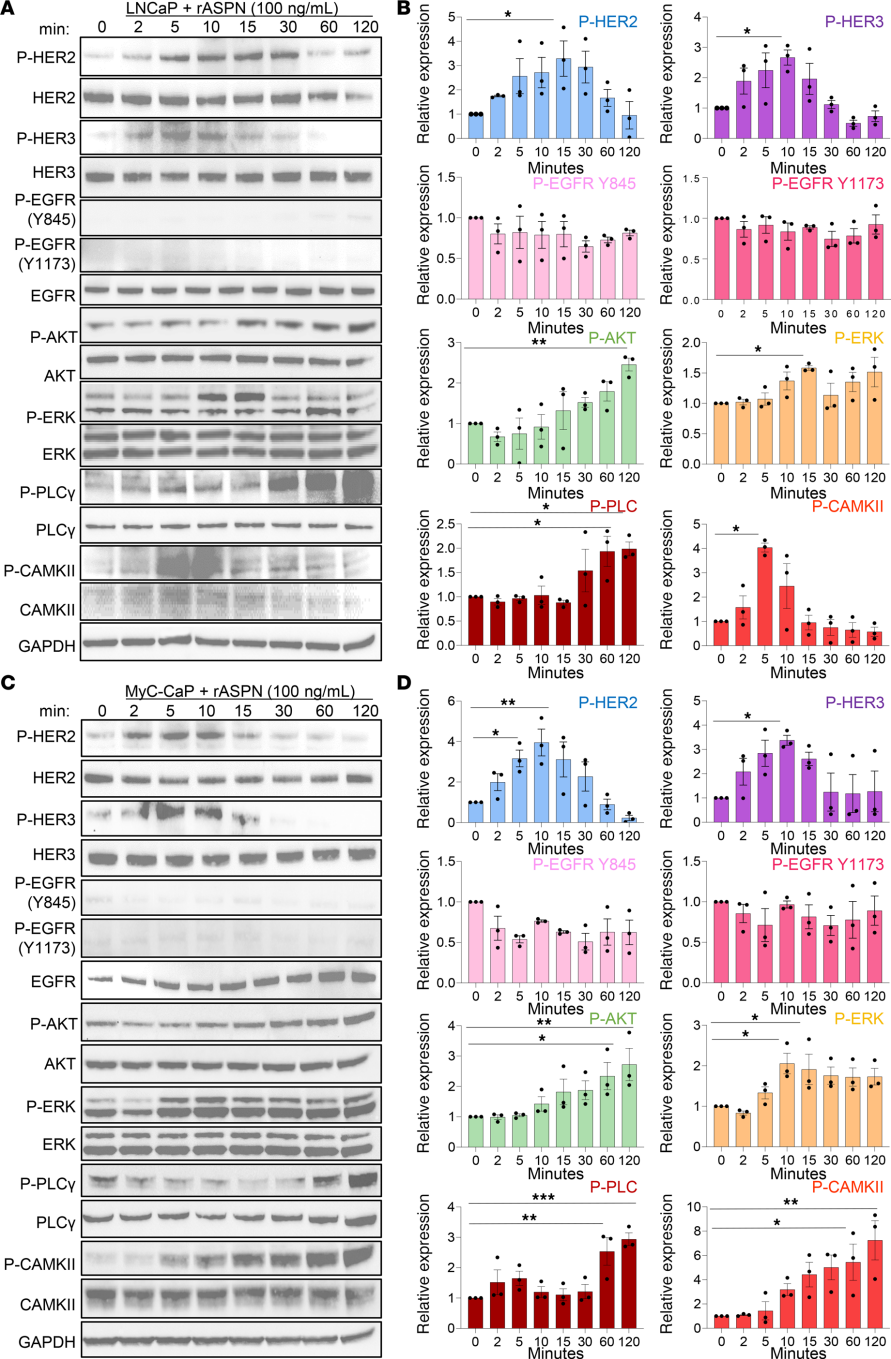
ASPN induces HER2/HER3 signaling in prostate cancer cells. (**A** and **B**) LNCaP cells treated with 100 ng/mL recombinant human ASPN over a time course and assessed by immunoblotting for HER2 and HER3 pathway activation (**A**) and quantification (**B**) (*n* = 3). (**C** and **D**) MyC-CaP cells treated with 100 ng/mL recombinant mouse ASPN over a time course and assessed by immunoblotting for HER2 and HER3 pathway activation (**C**) and quantification (**D**) (*n* = 3). For Western blots in **A** and **C**, total proteins were normalized to GAPDH, and phosphorylated proteins were normalized to GAPDH and total protein. Graphs in **B** and **D** are shown as mean ± SEM and analyzed by 1-way ANOVA with Dunnett’s post hoc analysis; *P ≤ 0.05, **P ≤ 0.01, ***P ≤ 0.001.

**Figure 3:**
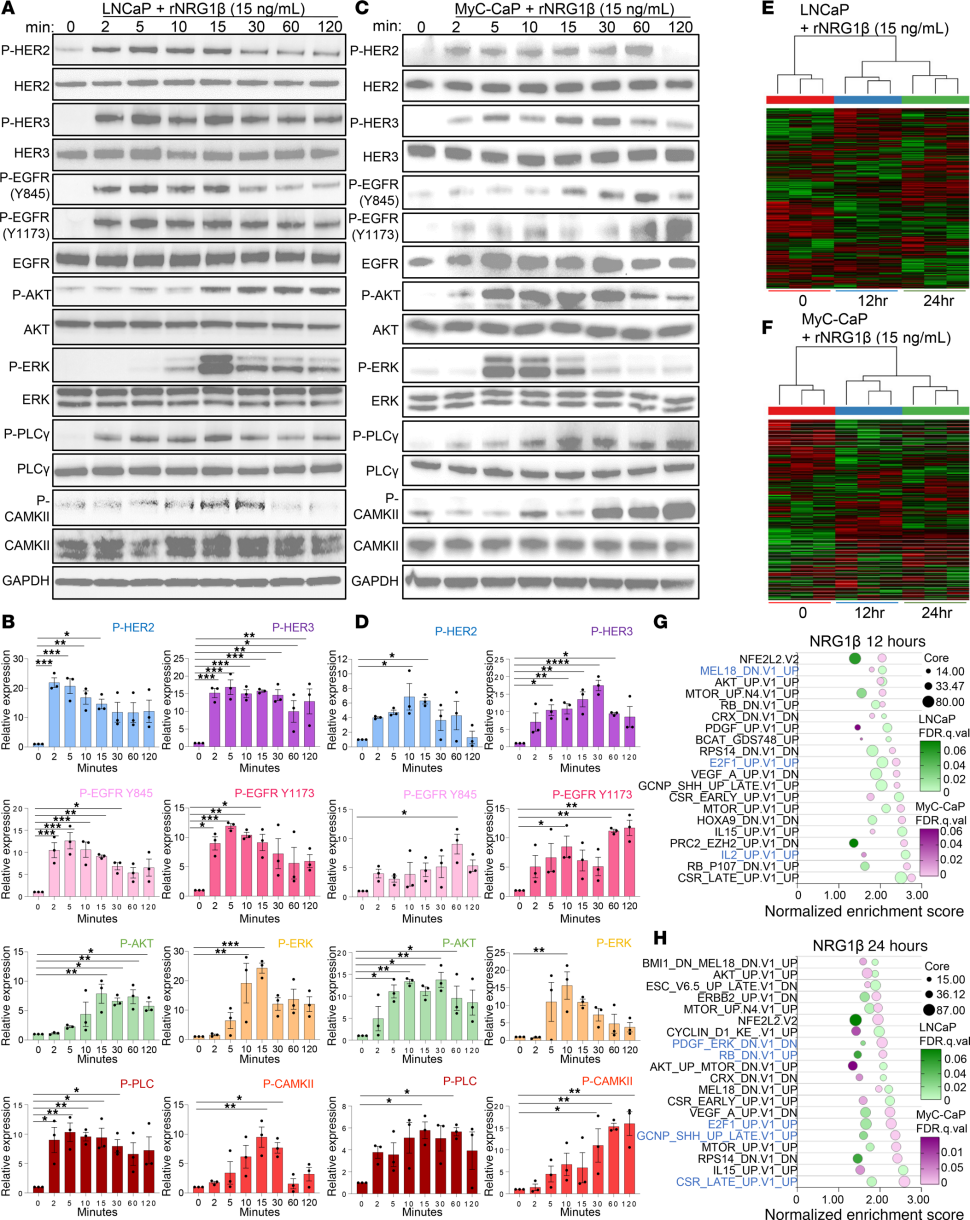
ASPN-induced signaling overlaps but is distinct from NRG1β, a HER3 ligand. (**A** and **B**) LNCaP cells treated with 15 ng/mL recombinant human NRG1β over a time course and assessed by immunoblotting for HER2 and HER3 pathway activation (**A**) and quantification (**B**) (*n* = 3). (**C** and **D**) MyC-CaP cells treated with 15 ng/mL recombinant mouse NRG1β over a time course and assessed by immunoblotting for HER2 and HER3 pathway activation (**C**) and quantification (**D**) (*n* = 3). (**E** and **F**) Heatmaps of LNCaP (**E**) and MyC-CaP (**F**) cells treated with 15 ng/mL recombinant human or mouse NRG1β for 0, 12, and 24 hours (*n* = 3). (**G** and **H**) Bubble plots of top 20 overlapping Oncogenic Signatures by GSEA of LNCaP and MyC-CaP cells treated with 15 ng/mL recombinant NRG1β for 12 hours (**G**) and 24 hours (**H**) compared with time 0. Pathways marked in blue represent signatures also upregulated in ASPN RNA-Seq at 12 and 24 hours. For Western blots in **A** and **C**, total proteins were normalized to GAPDH, and phosphorylated proteins were normalized to GAPDH and total protein. Graphs in **B** and **D** are shown as mean ± SEM and analyzed by 1-way ANOVA with Dunnett’s post hoc analysis; *P ≤ 0.05, **P ≤ 0.01, ***P ≤ 0.001, ****P ≤ 0.0001.

HER2/HER3 activation induces downstream signaling of multiple pathways, including PI3K/AKT (38), MAPK/ERK (39), and calcium signaling (6, 40). Consistent with HER2/HER3 activation, recombinant ASPN induced activating phosphorylations on AKT, ERK, and calcium pathway members (PLCγ, calcium/calmodulin-dependent protein II [CAMKII]) in human and mouse prostate cancer cells (Figure 2, A–D, and Supplemental Figure 2, A–D). HER2/HER3 signaling was also reproducible in human prostate cancer cells treated with conditioned media (CM) from patient-derived prostate CAFs (PCAFs) that express endogenous ASPN, supporting a paracrine interaction model (Supplemental Figure 2, L–N). Furthermore, recombinant ASPN-induced HER2/HER3 activation and downstream signaling in LNCaP and MyC-CaP showed similar patterns to the established HER3 ligand, NRG1β, but with altered kinetics and intensity when compared with NRG1β molar equivalents (41, 42) (Figure 3, A–D). In contrast with ASPN, NRG1β significantly induced EGFR phosphorylation in LNCaP and MyC-CaP, suggesting that ASPN has an overlapping, but distinct, ErbB activation profile compared with NRG1β (Figure 3, A–D). To compare ASPN and NRG1β signaling further, LNCaP and MyC-CaP were treated with recombinant NRG1β and assessed by RNA-Seq at 0, 12, and 24 hours (Figure 3, E and F). Comparison of overlapping Oncogenic Signatures by GSEA between ASPN and NRG1β-treated LNCaP and MyC-CaP cells showed enrichment of signatures associated with YAP, cyclin D, and immune signaling at 12 hours (Figure 3G and Supplemental Figure 3, A and B). In contrast with ASPN, ERBB2-downregulated genes were enriched at 12 hours in NRG1β-treated cells despite phosphorylation of HER2, HER3, and EGFR at earlier time points (Figure 3, A–D, and Supplemental Figure 3A). Signatures associated with RB1 loss and upregulation of E2F1 and SHH were observed at both 12 and 24 hours in NRG1β-treated cells while these changes were not observed until 24 hours in ASPN-treated cells (Figure 1G; Figure 3, G and H; and Supplemental Figure 3, A–D). Collectively, these findings demonstrate that like NRG1β, extracellular ASPN induces HER2 and HER3 activation and downstream signaling pathways, including PI3K/AKT, MAPK/ERK, and calcium signaling, in prostate cancer cells; however, ASPN-induced signaling and transcriptional changes demonstrate intensity, duration, and target repertoire differences compared with NRG1β.

### ASPN binds to the ligand binding domain of HER3.

**Figure 4:**
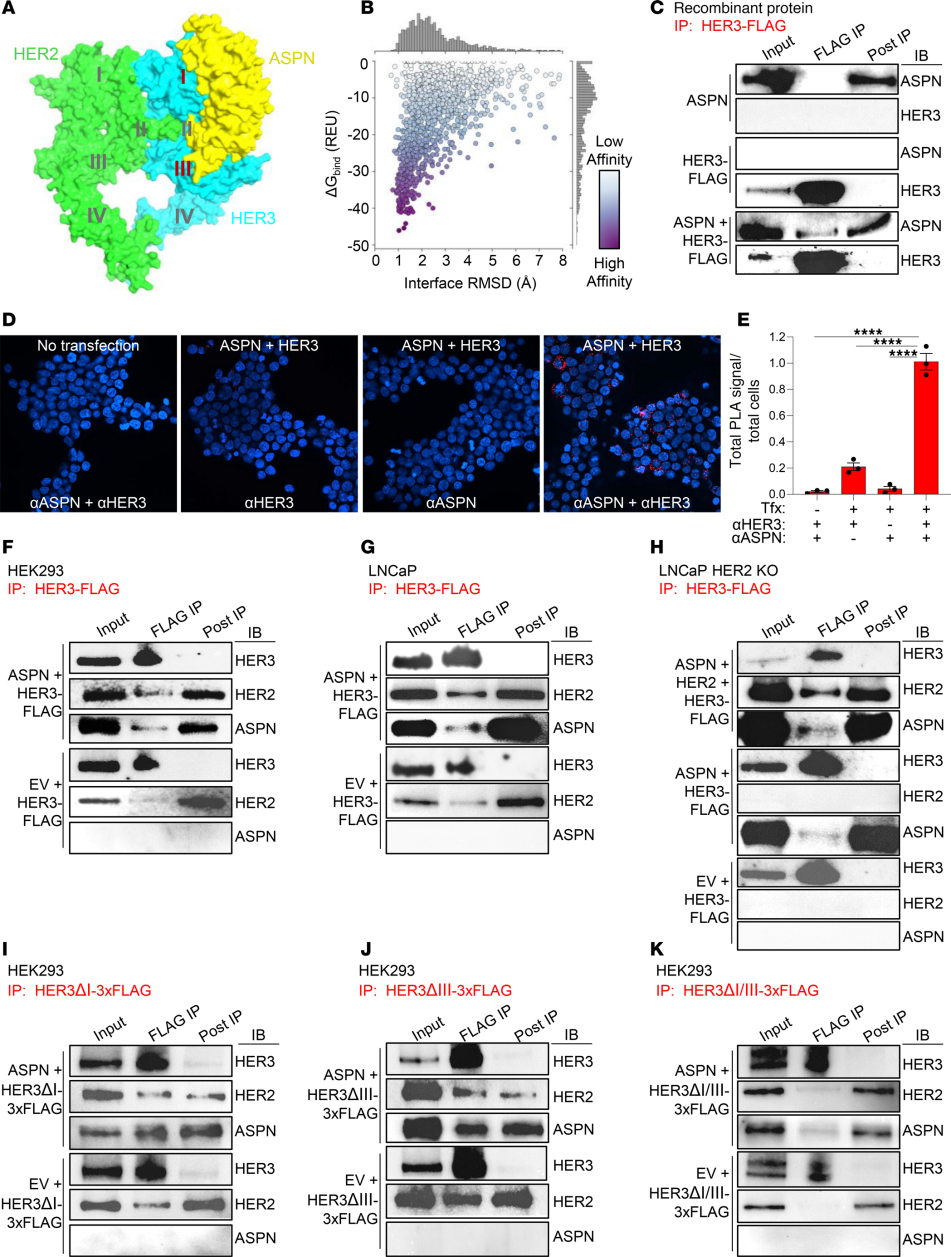
ASPN binds to the ligand binding domain of HER3. (**A**) Rosetta local docking funnel for ASPN (yellow) against the HER2 (green)/HER3 (blue) extracellular domain heterodimer of the AlphaFold2-predicted complex. Protein domains are labeled I-IV. Also shown in Supplemental Figure 4A for comparison with other models. (**B**) Scatterplot colored by Rosetta binding energy in REU using the REF2015 score function. 1D histograms represent the marginal distributions for the interface RMSD (top) and binding energy (bottom). Also shown in Supplemental Figure 4A for comparison with other models. (**C**) Recombinant human ASPN and recombinant human HER3-FLAG protein were incubated with vehicle or together in a cell-free assay, immunoprecipitated with anti-FLAG beads, and assessed for ASPN and HER3 by immunoblotting (*n* = 2). (**D** and **E**) HEK293 cells were transfected with ASPN-FLAG and HER3 or not transfected. Cells were assessed for protein interactions by proximity ligation assay (PLA) using confocal microscopy (**D**). Total PLA signal per total cell number was evaluated for each condition (no transfection: αASPN^+^αHER3 = 1,017 cells; ASPN and HER3 transfected: αHER3 alone = 1,705 cells, αASPN alone = 1,957 cells, and αASPN^+^αHER3 = 1,687 cells) (*n* = 3) (**E**). (**F**) HEK293 cells were transfected with HER3-FLAG and ASPN or empty vector (EV), immunoprecipitated with anti-FLAG beads, and assessed by immunoblotting (IB) for HER3, HER2, and ASPN (*n* = 2). (**G**) LNCaP cells were transfected with HER3-FLAG and ASPN or EV, immunoprecipitated with anti-FLAG beads, and assessed by immunoblotting for HER3, HER2, and ASPN (*n* = 2). (**H**) LNCaP HER2-KO cells were transfected with HER3-FLAG and HER2 and ASPN, ASPN, or EV; immunoprecipitated with anti-FLAG beads; and assessed by immunoblotting for HER3, HER2, and ASPN (*n* = 2). (**I**–**K**) HEK293 cells were transfected with HER3ΔI-3xFLAG (**I**), HER3ΔIII-3xFLAG (**J**), or HER3ΔI/III-3xFLAG (**K**) and ASPN or EV; immunoprecipitated with anti-FLAG beads; and assessed by immunoblotting for HER3, HER2, and ASPN (*n* = 2). Graphs are shown as mean ± SEM and analyzed by 1-way ANOVA with Tukey’s post hoc analysis; ****P ≤ 0.0001. REU, Rosetta energy units; RMSD, root mean square deviation; Tfx, transfection.

Extracellular ASPN activates HER2 and HER3. However, it is not known if ASPN directly complexes with HER2 and HER3 or if ASPN activates signaling through indirect mechanisms. Although HER2 does not have a known ligand (40), NRG1 and NRG2 are well-established ligands for HER3 (43). HER3 lacks an activation domain and must heterodimerize with other ErbB family members to induce signaling (44). To begin to assess if ASPN binds to HER2 or HER3, we applied computational algorithms using AlphaFold-Multimer (v2.3) with Rosetta refinement. Predicted binding structures with ASPN and the HER2/HER3 heterodimer were generated, and a final structural model was chosen based on the docking funnel convergence and overall binding energy (Figure 4, A and B, and Supplemental Figure 4A). Based on the final structure, ASPN showed preferential binding to HER3 compared with HER2 and was predicted to bind at the HER3 ligand binding domain (domains I and III).

To determine if ASPN binds directly to HER3 as structural models suggest, cell-free immunoprecipitation assays with recombinant human proteins were employed. Recombinant human ASPN was incubated with recombinant human HER3-FLAG, HER2-FLAG, or PDGFRβ-FLAG and then immunoprecipitated with anti-FLAG beads. PDGFRβ-FLAG was chosen as a putative negative control because of the lack of reported interactions between ASPN and the PDGFRβ pathway. ASPN immunoprecipitated with recombinant HER3, not with HER2 or PDGFRβ (Figure 4C and Supplemental Figure 4, B and C). These findings support that ASPN directly binds to HER3.

ASPN and HER3 binding interactions were next evaluated in a cellular model using a proximity ligation assay (PLA). HEK293 cells, which do not express HER3 or ASPN, were transfected with HER3 and ASPN-FLAG and then assessed for HER3-ASPN binding by PLA using confocal microscopy (Supplemental Figure 2K). ASPN and HER3 showed significant protein interactions at the cell membrane compared with controls as measured by fluorescence (Figure 4, D and E). ASPN and HER3 interactions were further examined in cellular models using co-immunoprecipitation. HEK293 cells were transfected with human HER3-FLAG and human ASPN or an empty vector, immunoprecipitated with anti-FLAG beads, and then assessed for HER3 co-immunoprecipitation with ASPN and endogenous HER2 by immunoblotting. Both ASPN and endogenous HER2 co-immunoprecipitated with HER3 (Figure 4F). Endogenous HER2 also co-immunoprecipitated with overexpressed HER3 in the absence of ASPN. Binding interactions were then assessed in LNCaP prostate cancer cells using the same transfection and immunoprecipitation protocol. Consistent with HEK293 cells, both ASPN and endogenous HER2 co-immunoprecipitated with HER3-FLAG. Endogenous HER2 also co-immunoprecipitated with HER3-FLAG in the absence of ASPN (Figure 4G). To determine if ASPN induces binding of endogenous HER3 and endogenous HER2, PC3 cells were treated with recombinant human ASPN, immunoprecipitated for endogenous HER3, and then assessed for HER2 binding. Indeed, treatment with recombinant ASPN induced binding between endogenous HER3 and endogenous HER2 (Supplemental Figure 4D). Consistent with these findings, CM from ASPN expressing PCAFs also induced endogenous HER3 and endogenous HER2 binding in PC3 cells (Supplemental Figure 4E).

To establish if HER2 is necessary for ASPN complexing with HER3 in a cellular model, ASPN and HER3 binding interactions were evaluated in LNCaP HER2-KO cells, which were generated using CRISPR/Cas9 (Supplemental Figure 4, F and G). LNCaP HER2-KO cells were initially transfected with HER3-FLAG, HER2, and ASPN; immunoprecipitated with anti-FLAG beads; and then assessed for co-immunoprecipitation of HER3 with ASPN and HER2 by immunoblotting. Both ASPN and HER2 co-immunoprecipitated with HER3-FLAG (Figure 4H). LNCaP HER2-KO cells were then transfected with HER3-FLAG and ASPN, immunoprecipitated with anti-FLAG beads, and assessed by immunoblotting. In the absence of HER2, ASPN still bound with HER3 (Figure 4H).

Structural models support that ASPN has key interactions with the HER3 ligand binding domain (domains I and III) and potentially within the ligand pocket (domain II). To assess this experimentally, ASPN binding interactions were evaluated with HER3 without domain I (ΔI), domain III (ΔIII), or both domains I and III (ΔI/III). HEK293 cells, which do not express detectable levels of HER3, were transfected with ASPN and HER3ΔI-FLAG, HER3ΔIII-FLAG, or HER3ΔI/III-FLAG; immunoprecipitated with anti-FLAG beads; and then assessed for co-immunoprecipitation of ASPN with HER3 mutants. ASPN bound to HER3ΔI and HER3ΔIII but showed limited binding to HER3ΔI/III (Figure 4, I–K), supporting that domains I and III are key for ASPN binding to HER3. Collectively, these findings demonstrate that extracellular ASPN binds directly to the ligand binding domain of HER3, and this complex is capable of binding with HER2.

### HER3 and HER2 are key mediators of ASPN-induced signaling.

**Figure 5:**
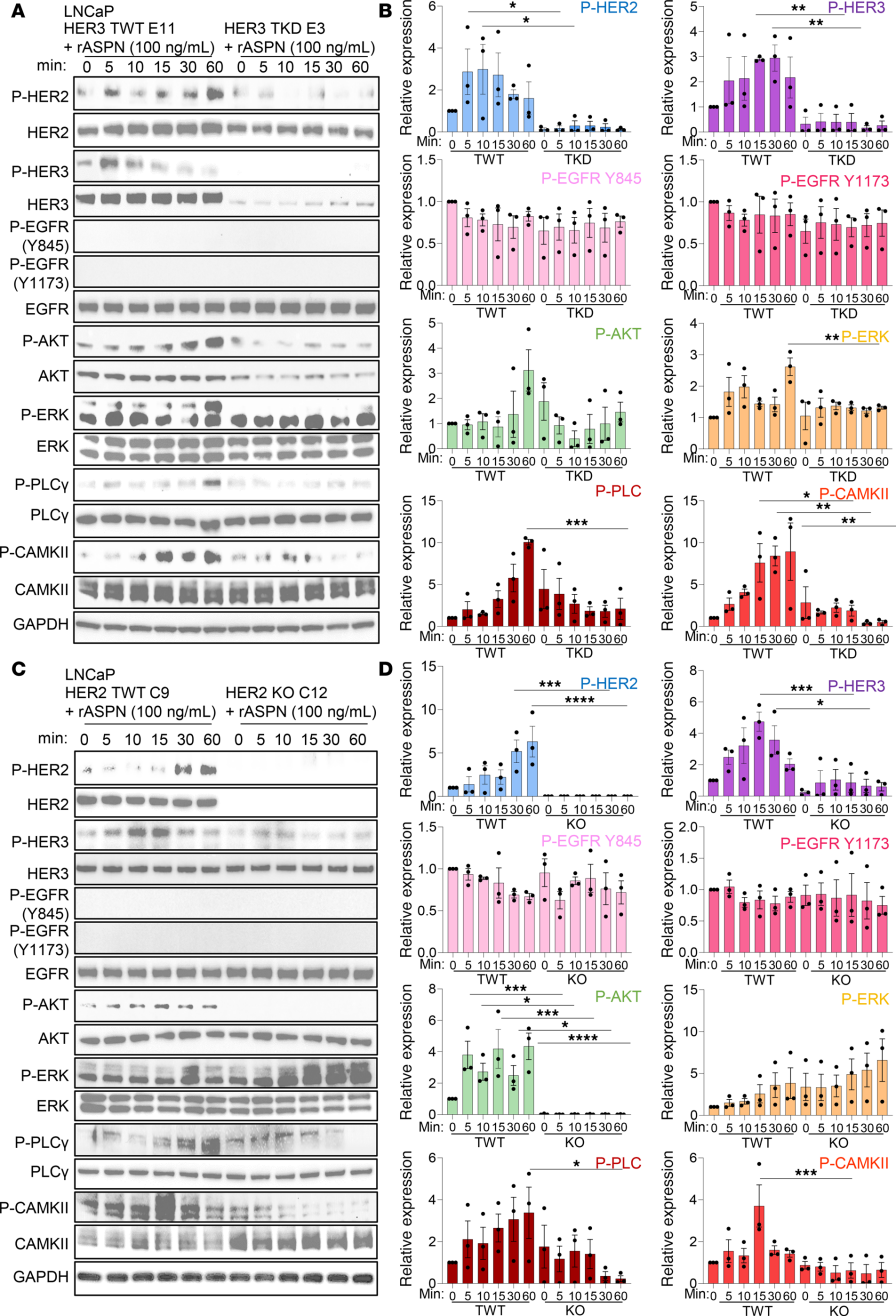
HER3 and HER2 are key mediators of ASPN-induced signaling. (**A** and **B**) LNCaP HER3 targeted wild-type (TWT) and LNCaP HER3 truncated knockdown (TKD) were treated with 100 ng/mL recombinant human ASPN over a time course and assessed by immunoblotting for HER2 and HER3 pathway activation (**A**) and quantification (**B**) (*n* = 3). (**C** and **D**) LNCaP HER2 TWT and LNCaP HER2-KO, without elevated baseline HER3, were treated with 100 ng/mL recombinant human ASPN over a time course and assessed by immunoblotting for HER2 and HER3 pathway activation (**C**) and quantification (**D**) (*n* = 3). Graphs are shown as mean ± SEM and analyzed by 1-way ANOVA with Šídák’s post hoc analysis; *P ≤ 0.05, **P ≤ 0.01, ***P ≤ 0.001, ****P ≤ 0.0001.

Our findings support a model in which ASPN binds directly to HER3, induces heterodimerization with HER2, and leads to activation of downstream pathway members AKT, ERK, PLCγ, and CAMKII in human and mouse prostate cancer cells. To determine the dependence of ASPN-induced signaling on HER3, LNCaP HER3 truncated knockdown (TKD) cells were generated using CRISPR/Cas9 with guides targeting the intracellular kinase domain. LNCaP HER3 targeted wild-type (TWT) control cells were also created, as these cells underwent the same gene-editing procedures but remained wild-type for HER3. Compared with LNCaP parental or LNCaP HER3 TWT cells, HER3 protein expression was truncated and reduced by approximately 90% in LNCaP HER3 TKD (Supplemental Figure 4, H and I). Independent clones of LNCaP HER3 TWT and LNCaP HER3 TKD were treated with recombinant human ASPN and assessed by immunoblotting for signal activation. In LNCaP HER3 TKD cells, ASPN did not induce HER2 phosphorylation, suggesting that HER3 is necessary for ASPN-induced HER2 activation (Figure 5, A and B, and Supplemental Figure 5A). Consistent with LNCaP parental, ASPN did not induce the phosphorylation of EGFR in LNCaP HER3 TKD cells. Additionally, ASPN-induced phosphorylation of downstream pathway members, including AKT, ERK, PLCγ, and CAMKII, was abrogated in HER3 TKD cells. To further support the importance of HER3 in ASPN-induced signaling, LNCaP HER3 TKD were transfected with either WT HER3 or the ΔI/III HER3 mutant, treated with recombinant human ASPN, and then assessed by immunoblot for HER2/HER3 pathway activation. Notably, ASPN activated HER2/HER3 signaling in the cells transfected with WT HER3, but signaling was diminished in cells transfected with the ΔI/III HER3 mutant (Supplemental Figure 5B). Combined, these findings identify HER3 as a key mediator of ASPN-induced signaling and demonstrate the importance of HER3 domains I and III for proper ASPN binding and signal transduction.

To determine if HER2 is necessary for ASPN-induced signaling, extracellular ASPN-induced pathway activation was assessed in independent clones of LNCaP HER2 TWT and LNCaP HER2 KO cells. Compared with LNCaP TWT clones, LNCaP HER2 KO clones showed variable HER3 and p-ERK expression, with 2 of the 3 clones having significantly elevated HER3 and p-ERK expression at baseline (Figure 5, C and D, and Supplemental Figure 5, C and D). Regardless of baseline HER3 and p-ERK expression, ASPN did not significantly induce the phosphorylation of AKT, PLCγ, and CAMKII in the absence of HER2. These findings highlight the importance of HER2 for ASPN-induced signaling. Overall, findings from genetic models support that both HER3 and HER2 are important mediators of ASPN-induced signaling.

### Tucatinib, a small molecule inhibitor of HER2, restricts ASPN-induced signaling and prostate cancer cell migration.

**Figure 6:**
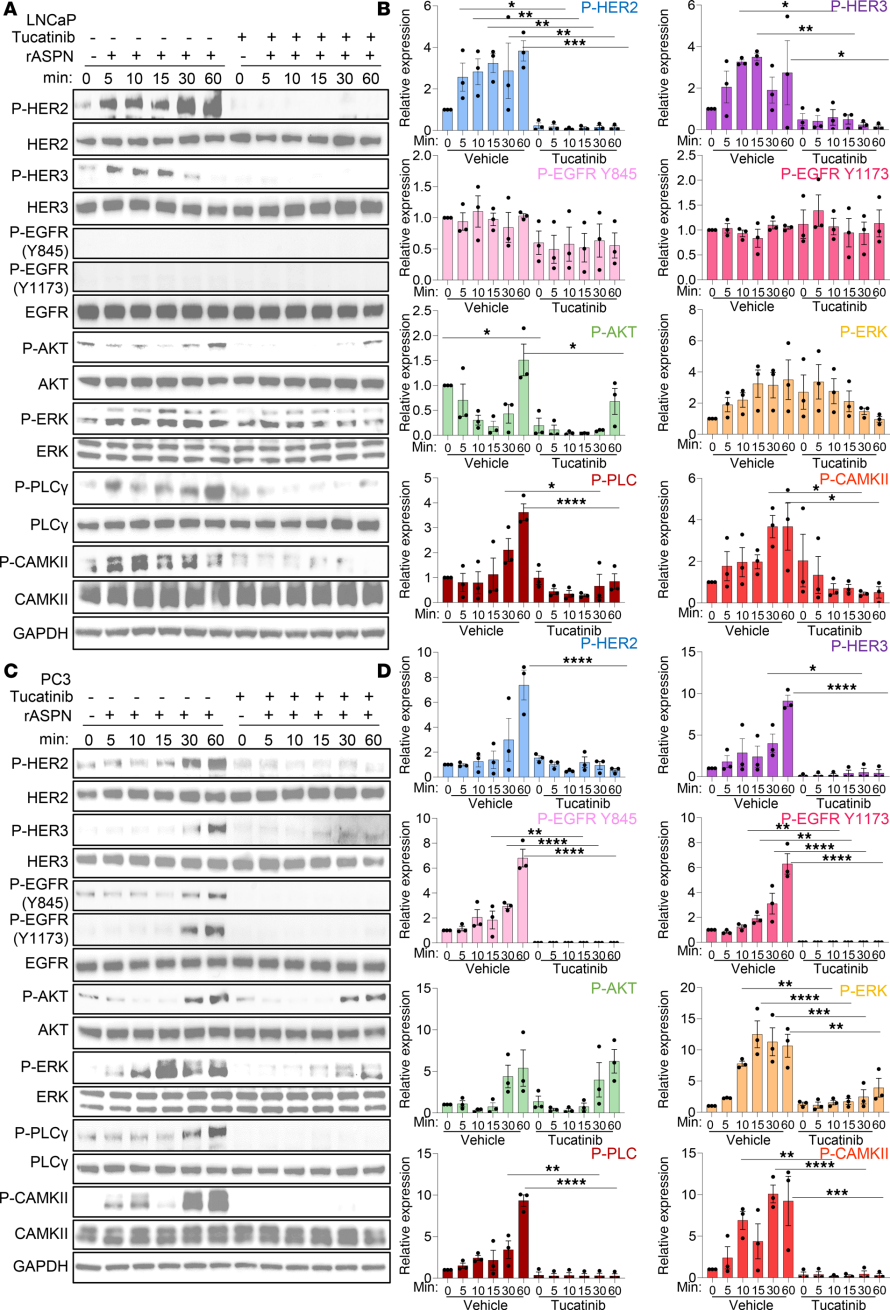
Tucatinib, a small molecule inhibitor of HER2, restricts ASPN-induced signaling in prostate cancer cells. (**A** and **B**) LNCaP cells were treated with 100 ng/mL recombinant human ASPN with or without 0.5 μM tucatinib and then assessed for HER2 and HER3 pathway activation by immunoblotting (**A**) and quantification (**B**) (*n* = 3). (**C** and **D**) PC3 cells were treated with 100 ng/mL recombinant human ASPN with or without 20 μM tucatinib and then assessed for HER2 and HER3 pathway activation by immunoblotting (**C**) and quantification (**D**) (*n* = 3). Graphs are shown as mean ± SEM and analyzed by 1-way ANOVA with Šídák’s post hoc analysis; *P ≤ 0.05, **P ≤ 0.01, ***P ≤ 0.001, ****P ≤ 0.0001.

Due to the critical role of HER2 in ASPN signaling, therapies targeting HER2 may offer a new method for inhibiting ASPN-induced signaling. To determine the efficacy of HER2-targeted therapies in restricting ASPN-induced signaling, parental LNCaP and PC3 cells were treated with ASPN plus tucatinib or vehicle. Tucatinib is a small molecule HER2 inhibitor approved for HER2^+^ breast cancer (45–47). In both LNCaP and PC3, tucatinib reduced ASPN-mediated phosphorylation of HER2, HER3, ERK, PLCγ, and CAMKII, while phosphorylation of AKT was variable between cell lines (Figure 6, A–D). These findings indicate that ASPN-induced signaling is inhibited by therapeutically targeting HER2 with tucatinib.

**Figure 7:**
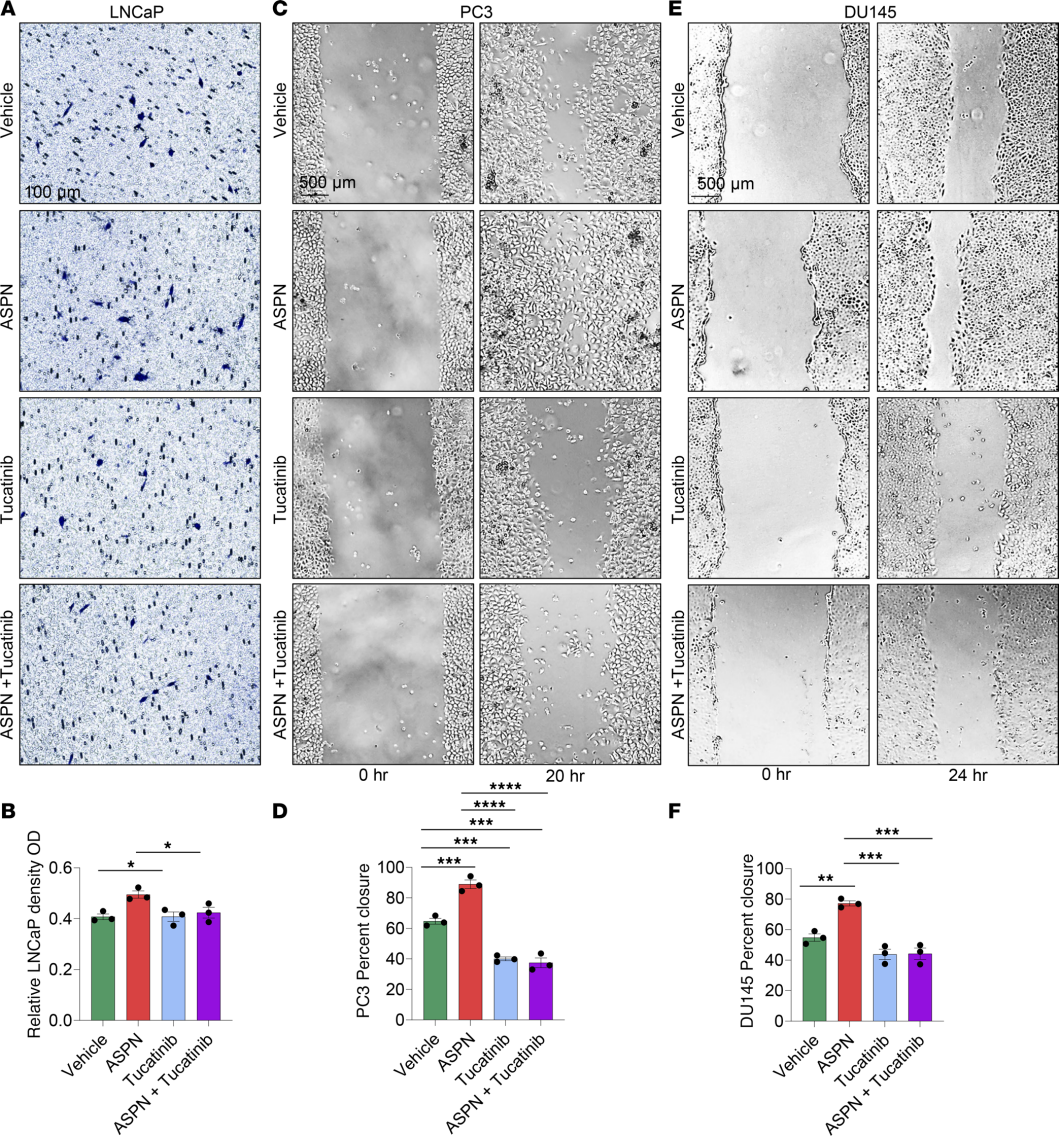
Tucatinib, a small molecule inhibitor of HER2, restricts ASPN-induced prostate cancer cell migration. (**A** and **B**) Microscopic images of Transwell migration assay at 24 hours of LNCaP treated with vehicle, 100 ng/mL recombinant human ASPN, 20 μM tucatinib, or ASPN and tucatinib (**A**). Colorimetric quantification of Transwell assay performed at 570 nm and quantified using ImageJ (NIH) (**B**) (*n* = 3). (**C** and **D**) Microscopic images of scratch assay at 0 and 20 hours of PC3 treated with vehicle, 100 ng/mL recombinant human ASPN, 20 μM tucatinib, or ASPN and tucatinib (**C**). Scratch assay quantification using ImageJ (**D**) (*n* = 3). (**E** and **F**) Microscopic images of scratch assay at 0 and 24 hours of DU145 treated with vehicle, 100 ng/mL recombinant human ASPN, 20 μM tucatinib, or ASPN and tucatinib (**E**). Scratch assay quantification using ImageJ (**F**) (*n* = 3). Graphs are shown as mean ± SEM and analyzed by 1-way ANOVA with Dunnett’s post hoc analysis; *P ≤ 0.05, **P ≤ 0.01, ***P ≤ 0.001, ****P ≤ 0.0001.

Prior studies have shown that ASPN^+^ CAFs induce cancer cell migration (23, 28, 35, 37), but the mechanisms for this are not fully understood. To determine if ASPN mediates migration through HER2, we initially assessed ASPN-induced migration in LNCaP cells in the presence of tucatinib. LNCaP cells were treated with vehicle, recombinant human ASPN, tucatinib, or ASPN plus tucatinib and then assessed for migration by Transwell assay. LNCaP cells alone were minimally migratory as assessed by Transwell assays. However, ASPN significantly increased LNCaP migration, while tucatinib ablated this effect (Figure 7, A and B). Compared with LNCaP cells, PC3 and DU145 cells were more migratory at baseline. Therefore, a scratch assay was utilized to assess ASPN-induced migration in these cells. Like LNCaP, ASPN induced cell migration in PC3 and DU145 cells, which was inhibited by cotreatment with tucatinib (Figure 7, C–F). Combined, these findings demonstrate that ASPN-induced signaling and cell migration are restricted by HER2 inhibition with tucatinib.

### Stromal expression of ASPN occurs in the TME of HER2/HER3-expressing metastatic prostate cancer.

**Figure 8:**
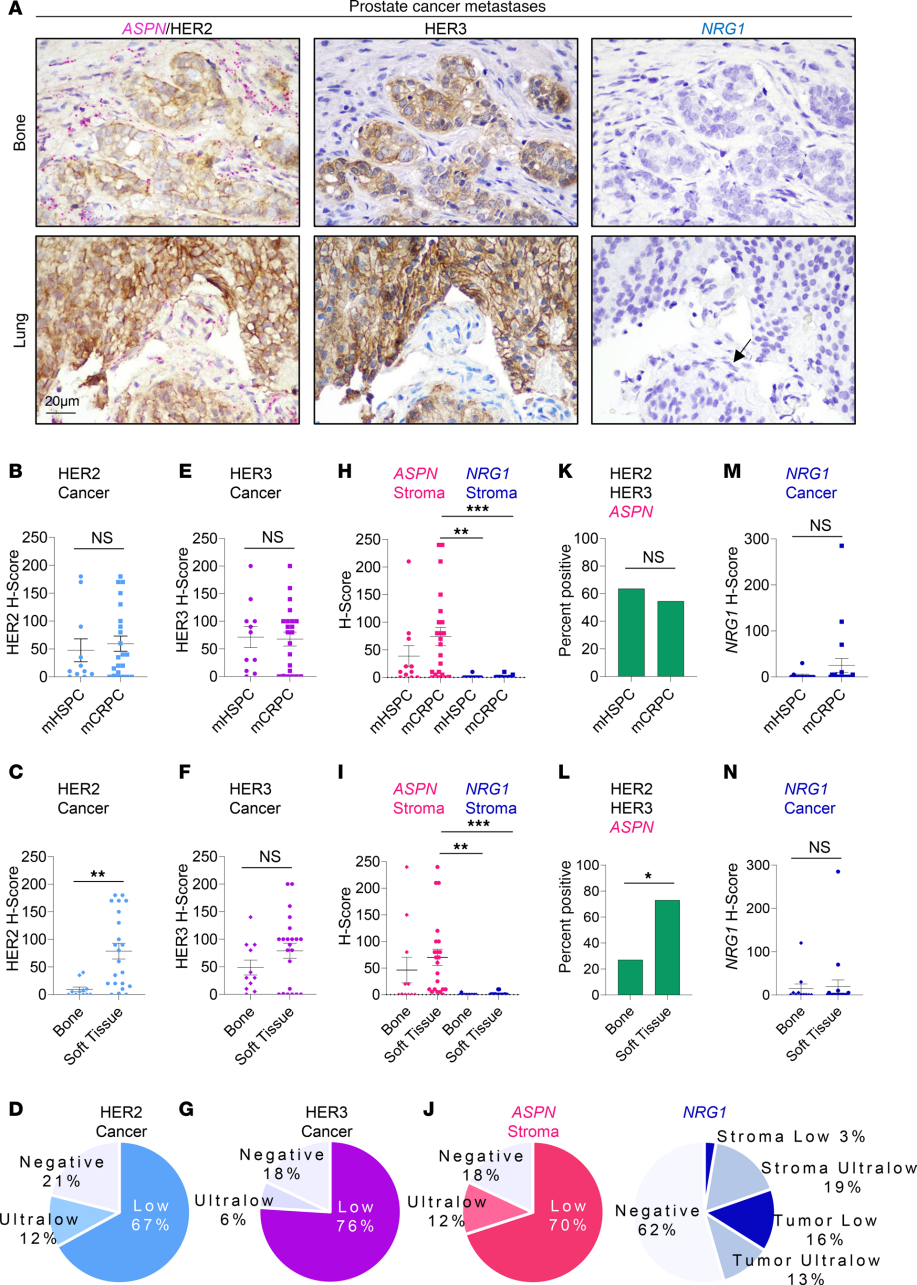
Stromal expression of *ASPN* occurs in the TME of HER2/HER3-expressing metastatic prostate cancer. (**A**) Representative images of distant prostate cancer metastases analyzed by dual IHC for HER2 and RNAscope for *ASPN* (*n* = 33), IHC for HER3 (*n* = 33), and RNAscope for *NRG1* (*n* = 31). The black arrow highlights an *NRG1*-positive cell. (**B**–**D**) Quantification of HER2 expression in cancer cells by IHC as determined by H-Score (0, 1^+^, 2^+^, 3^+^ intensity × percent positive) and compared by mHSPC (*n* = 11) versus mCRPC (*n* = 22) (**B**), bone (*n* = 11) versus soft tissue (*n* = 22) (**C**), and all sites (*n* = 33) (**D**). (**E**–**G**) Quantification of HER3 expression in cancer cells by IHC as determined by H-Score (0, 1^+^, 2^+^, 3^+^ intensity × percent positive) and compared by mHSPC versus mCRPC (**E**), bone versus soft tissue (**F**), and all sites (**G**). (**H**–**J**) Quantification of *ASPN* (*n* = 33) and *NRG1* (*n* = 31) expression in metastatic prostate cancer stroma by RNAscope as determined by H-Score (0, 1^+^, 2^+^, 3^+^ intensity × percent positive) and compared by mHSPC (*n* = 11) versus mCRPC (*n* = 22 for *ASPN* and *n* = 20 for *NRG1*) (**H**), bone (*n* = 11) versus soft tissue (*n* = 22 for *ASPN* and *n* = 20 for *NRG1*) (**I**), and all sites (*n* = 33 for *ASPN* and *n* = 31 for *NRG1*) (**J**). (**K** and **L**) Percentage of samples that expressed HER2, HER3, and *ASPN* by mHSPC versus mCRPC (**K**) and bone versus soft tissue (**L**). (**M** and **N**) Quantification of *NRG1* (*n* = 31) expression in cancer cells by RNAscope as determined by H-Score (0, 1^+^, 2^+^, 3^+^ intensity × percent positive) and compared by mHSPC (*n* = 11) versus mCRPC (*n* = 20) (**M**) and bone (*n* = 11) versus soft tissue (*n* = 20) (**N**). Graphs shown as mean ± SEM and analyzed by Student’s 2-tailed *t* test (**B**, **C**, **E**, **F**, **M**, and **N**) or 1-way ANOVA with Tukey’s post hoc analysis (**H** and **I**). Graphs are shown as percent and analyzed by Fisher’s exact test (**K** and **L**); *P ≤ 0.05, **P ≤ 0.01, ***P ≤ 0.001.

Our data demonstrate that ASPN is a previously unidentified activator of HER2/HER3 signaling and HER2-dependent migration in prostate cancer cells, thereby supporting a role for this signaling axis in advanced prostate cancer. To assess this pathway in patient tissue samples, HER2 and *ASPN* expression levels were examined in metastases from 33 patients by dual immunohistochemistry (IHC) (HER2) and RNAscope (*ASPN*) (Supplemental Figure 6A). Dual IHC/RNAscope was optimized to meet metrics for diagnostic HER2 IHC staining (Figure 8A and Supplemental Figure 6, B–D). HER3 was similarly optimized and assessed on serial sections by IHC (Figure 8A and Supplemental Figure 6, C and D). HER2 expression was quantified in accordance with breast cancer clinical guidelines for intensity (0, 1^+^, 2^+^, 3^+^) and percent positive staining, and it was reported as an H-Score (intensity × percent positive staining) as well as HER2-low (IHC 1–2^+^ ≥ 10% of cancer cells) and HER2-ultralow (IHC 1^+^ at < 10% of cancer cells). HER3 and *ASPN* expression were similarly assessed by H-Score. Expression of *ASPN*, HER2, and HER3 was examined in metastatic hormone-sensitive prostate cancer (mHSPC) and mCRPC. Metastatic prostate cancer was isolated from various sites including bone, lung, brain, liver, lymph node, adrenal gland, and colon (Figure 8A and Supplemental Figure 6, A and D). In this cohort, 67% of metastatic samples were HER2-low while an additional 12% of samples were HER2-ultralow (Figure 8, A–D, and Supplemental Figure 6E). HER2 H-Score was not significantly different between mHSPC and mCRPC metastases; however, it was significantly increased in prostate cancer metastases from soft tissue sites compared with bone sites (Figure 8, B and C). Seventy-six percent of the metastatic samples were HER3-low, with an additional 6% of samples having HER3-ultralow staining; H-scores were similar between metastatic sites (Figure 8, A and E–G, and Supplemental Figure 6D). Stromal *ASPN* expression was observed in 82% of prostate cancer metastases, and over 50% of metastases were low/ultralow for both HER2 and HER3 and had stromal *ASPN* expression (Figure 8, A and H–L, and Supplemental Figure 6D). These findings indicate that *ASPN* is found in the TME of HER2/HER3-expressing metastatic prostate cancer, thereby supporting a role for the ASPN-HER2/HER3 signaling axis in metastatic prostate cancer.

A prior study found that NRG1 was detected at low levels in CAFs from mCRPC (11), but how NRG1 expression compares with ASPN is not known. To assess stromal expression of *NRG1* in mHSPC and mCRPC samples, serial FFPE sections were analyzed using RNAscope for *NRG1*, quantified for intensity (0, 1^+^, 2^+^, 3^+^) and percent positive staining, and reported as an H-Score (intensity × percent positive staining). Stromal expression of *NRG1* was minimal and significantly less than *ASPN* in mCRPC (Figure 8, A and H–J, and Supplemental Figure 6, D and F). While ASPN expression is restricted to the stroma of prostate cancer metastases, NRG1 expression has been reported in cancer cells (11). Consistent with this, a small subset of mHSPC and mCRPC showed cancer cell expression of *NRG1* (Figure 8, J, M, and N). Collectively, these findings support that stroma-derived ASPN mediates paracrine activation of HER2/HER3 in metastatic prostate cancer while a subset of cancers may have additional autocrine or paracrine activation of HER2/HER3 signaling through NRG1.

### ADCs designed to target HER2 or HER3 restrict growth of prostate cancer cells in vitro and in vivo.

**Figure 9:**
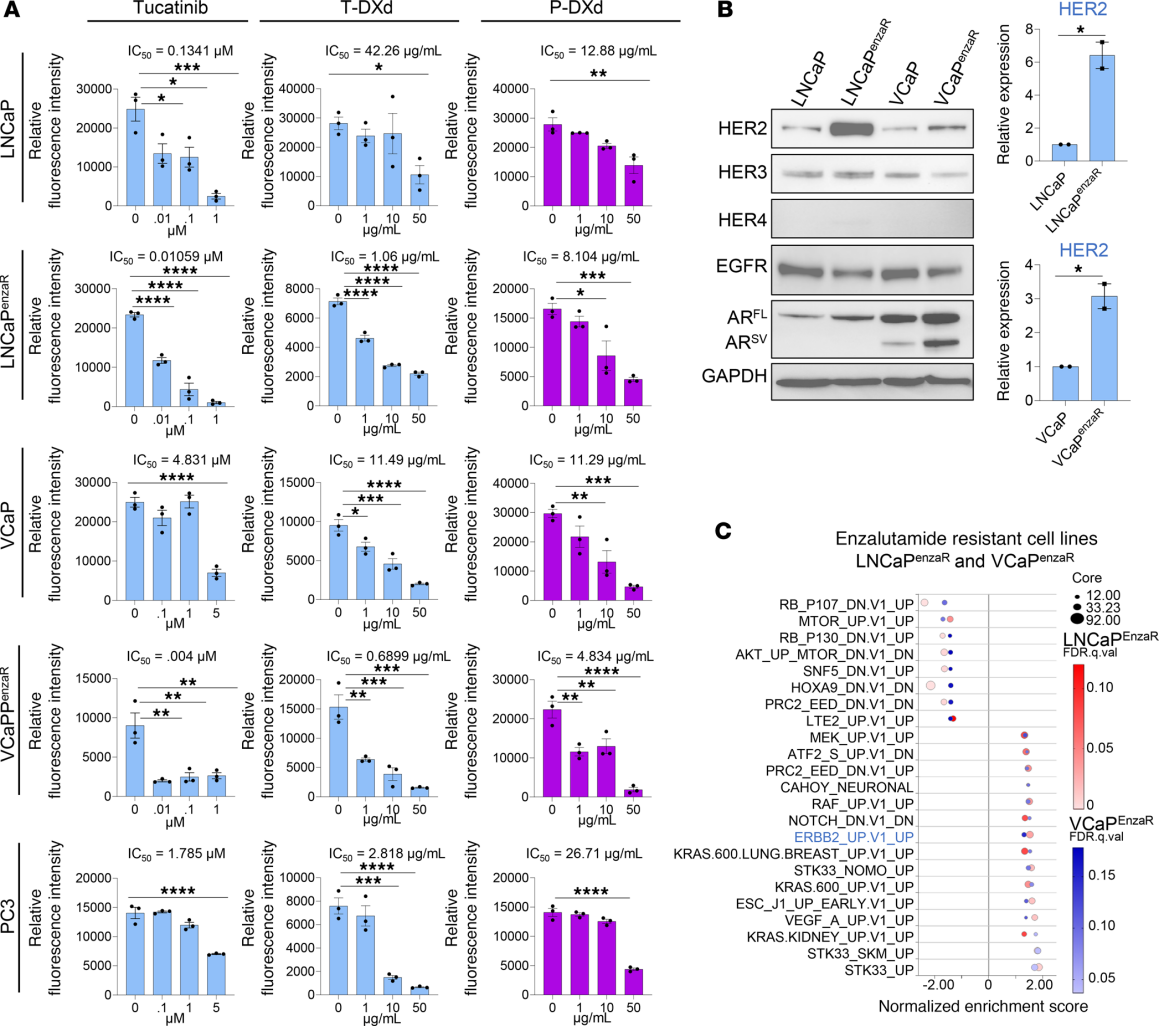
Antibody drug conjugates designed to target HER2 or HER3 restrict growth of prostate cancer cells in vitro. (**A**) LNCaP, LNCaP^enzaR^, VCaP, VCaP^enzaR^, and PC3 treated with increasing concentrations of tucatinib, trastuzumab-deruxtecan (T-DXd), and patritumab-deruxtecan (P-DXd). Inferred quantification of cell number by relative fluorescence intensity using CyQuant (*n* = 3). (**B**) Immunoblots for ErbB family members and AR including both the full-length (AR^FL^) and the truncated splice variant (AR^SV^) in LNCaP, LNCaP^enzaR^, VCaP, and VCaP^enzaR^ and corresponding quantification (*n* = 2). (**C**) Bubble plots of overlapping Oncogenic Signatures by GSEA of LNCaP^enzaR^ and VCaP^enzaR^ cells compared with LNCaP and VCaP, respectively. Graphs are shown as mean ± SEM and analyzed by 1-way ANOVA with Dunnett’s post hoc analysis (**A**) or Student’s 2-tailed *t* test (**B**); *P ≤ 0.05, **P ≤ 0.01, ***P ≤ 0.001, ****P ≤ 0.0001.

These data support a mechanism of HER3/HER2 activation and migration by ASPN in prostate cancer, thereby highlighting HER2 and HER3 as potential therapeutic vulnerabilities in metastatic prostate cancer. Clinical trials of antibody-based therapies directed against HER2/HER3, including trastuzumab (48–50) and pertuzumab (51, 52), however, lacked efficacy in patients with mCRPC. Consistent with trial results, trastuzumab and pertuzumab did not inhibit the growth of LNCaP, LNCaP^enzaR^, VCaP, VCaP^enzaR^, or PC3, all cell lines derived from metastatic prostate cancers (Supplemental Figure 7, A and B). Findings were similar for disitamab, a HER2-directed antibody therapy, and patritumab, a HER3-directed antibody therapy (Supplemental Figure 7, C and D). In contrast with therapeutic antibodies, prostate cancer cells showed sensitivity to increasing dosages of tucatinib, a small molecule HER2 inhibitor (Figure 9A). While tucatinib is currently approved for the treatment of HER2^+^ (IHC 3^+^ or IHC 2^+^/ISH^+^) breast cancer (45, 53), our findings, which are consistent with prior reports (54–59), indicate that few prostate cancers meet HER2^+^ breast cancer classification criteria and are, instead, HER2-low or HER2-ultralow (Figure 8, A–D). On the other hand, trastuzumab deruxtecan (T-DXd), an ADC, has shown efficacy in HER2^+^, HER2-low, and HER2-ultralow metastatic breast cancer (60, 61) as well as in HER2^+^ and HER2-low endometrial, cervical, ovarian, and bladder cancers (62). A case report recently showed efficacy of T-DXd in a patient with HER2-low mCRPC who had progressed on multiple lines of therapy (16), and T-DXd is now being assessed in a phase II clinical trial for mCRPC (ClinicalTrials.gov NCT06610825). Within our panel of prostate cancer cells, T-DXd significantly decreased cell growth of all cells analyzed (Figure 9A). Like T-DXd, disitamab vedotin (DV) is an ADC designed to target HER2 with the monoclonal antibody hertuzumab conjugated to the microtubule inhibitor monomethyl auristatin E (MMAE). DV is currently approved for second-line treatment of HER2^+^ urothelial carcinoma (63). DV also decreased cell growth in all metastatic prostate cancer cell lines analyzed (Supplemental Figure 7E). We next tested the sensitivity of these cells to P-DXd, which is an ADC designed to target HER3 and is currently being assessed in multiple phase II clinical trials (NCT05865990, NCT04676477, NCT04699630, and NCT06172478). P-DXd also inhibited the growth of prostate cancer cells in vitro (Figure 9A). Since comparable concentrations of trastuzumab, disitamab, or patritumab did not affect cell growth, T-DXd–, DV-, and P-DXd–induced toxicity was likely due to the conjugated deruxtecan or MMAE (Figure 9A and Supplemental Figure 7, A and C–E). To determine if ASPN restricts therapeutic efficacy, LNCaP, LNCaP^enzaR^, VCaP, VCaP^enzaR^, and PC3 were treated with therapy alone or in combination with ASPN. Consistent with signaling and migration studies, the efficacy of tucatinib, T-DXd, DV, or P-DXd at inhibiting cell growth was not diminished by ASPN in vitro (Supplemental Figure 8, A–D). Collectively, these findings indicate that monoclonal antibody therapies directed against HER2 and HER3 lack efficacy in metastatic prostate cancer cells, but corresponding ADCs may have therapeutic potential.

Interestingly, LNCaP^enzaR^ and VCaP^enzaR^ were more sensitive to tucatinib and T-DXd compared with their parental counterparts, LNCaP and VCaP (Figure 9A). Consistent with prior studies supporting HER2/HER3 signaling as a potential mechanism to bypass AR dependency in mCRPC (21, 64), LNCaP^enzaR^ and VCaP^enzaR^ had increased HER2 expression compared with LNCaP and VCaP (Figure 9B). RNA-Seq followed by GSEA indicated that both LNCaP^enzaR^ and VCaP^enzaR^ were enriched for the ERBB2-specific gene signature compared with parental cells (Figure 9C and Supplemental Figure 8, E–H). These findings suggest that some anti-HER2 therapies may have increased efficacy in enzalutamide-resistant prostate cancers when HER2 expression is elevated.

**Figure 10:**
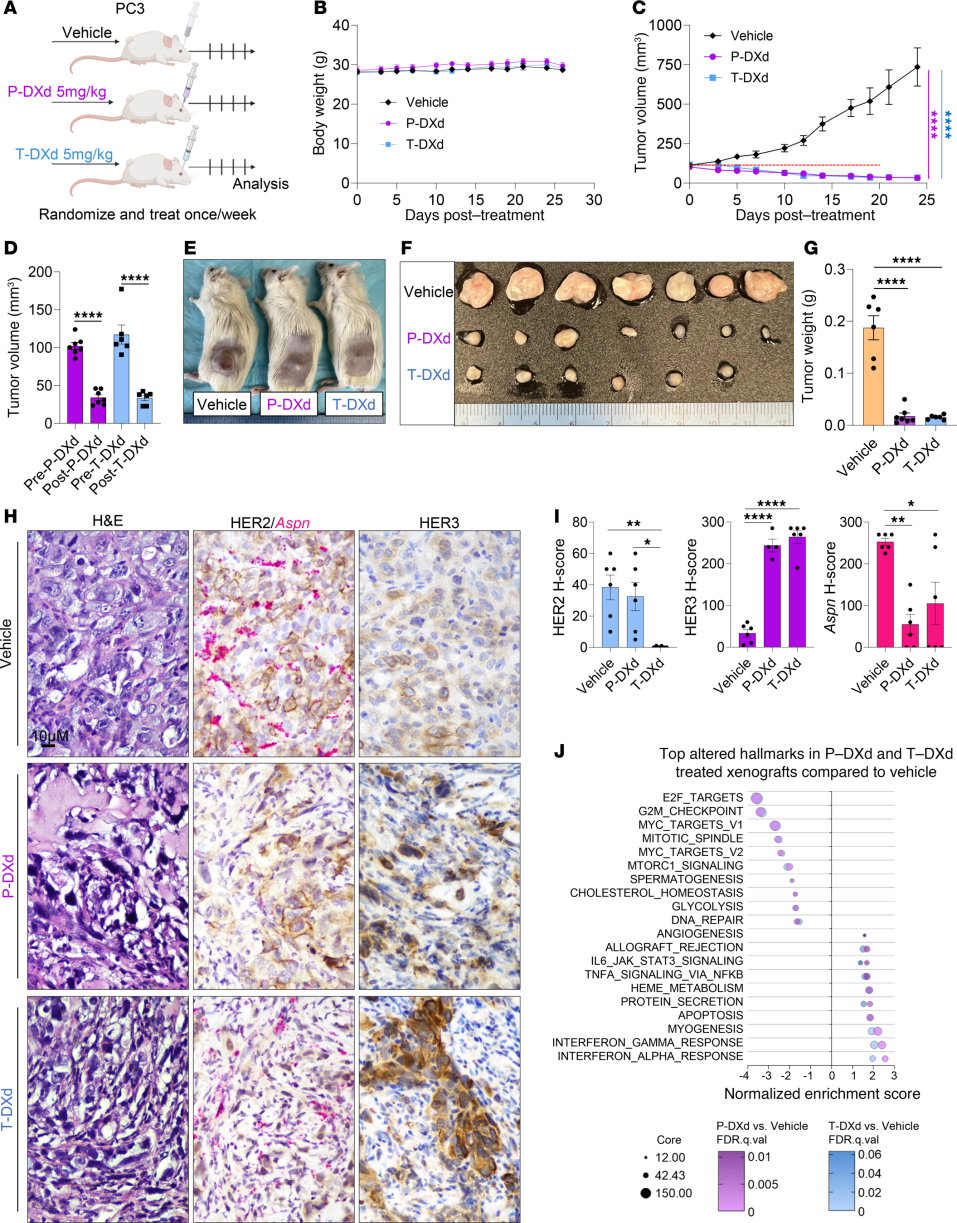
ADCs designed to target HER2 or HER3 restrict growth of prostate cancer cells in vivo. (**A**) Schematic of PC3 subcutaneous xenografts grown to approximately 100 mm^3^ in NSG mice and then treated with vehicle (*n* = 6), 5 mg/kg P-DXd (*n* = 7), or 5 mg/kg T-DXd (*n* = 6) by retro-orbital injection weekly for 4 cycles. (**B**) Total weight of NSG mice with PC3 xenografts treated with vehicle, P-DXd, or T-DXd. (**C**) Growth curves of PC3 xenografts in NSG mice treated with vehicle, P-DXd, or T-DXd. (**D**) Tumor volume prior to P-DXd (pre-P-DXd) and T-DXd (pre-T-DXd) and after 4 cycles of weekly P-DXd (post-P-DXd) and T-DXd (post-T-DXd). (**E** and **F**) Photograph of representative NSG mice with PC3 xenografts (**E**) or isolated xenografts (**F**) treated with vehicle, P-DXd, or T-DXd at experimental endpoint. The fourth vehicle tumor was excluded from analyses due to the lack of a palpable tumor at treatment initiation. (**G**) Tumor weight of isolated PC3 xenografts in NSG mice treated with vehicle, P-DXd, or T-DXd at experimental endpoint. (**H** and **I**) Representative H&E, dual IHC/RNAscope for HER2 (IHC) and *Aspn* (RNAscope), and IHC for HER3 of PC3 xenografts treated with vehicle, P-DXd, or T-DXd. HER2, HER3, and *Aspn* expression (**H**) quantified by H-score (intensity × percent) (**I**). (**J**) Bubble plots of top 20 overlapping Hallmarks by GSEA of T-DXd– and P-DXd–treated xenografts compared with vehicle. Graphs shown as mean ± SEM and analyzed by multiple Student’s *t* test (**C**) or 1-way ANOVA with Tukey’s (**D**) or Dunnett’s post hoc analysis (**G** and **I**); *P ≤ 0.05, **P ≤ 0.01, ***P ≤ 0.001, ****P ≤ 0.0001.

Due to its clinical efficacy in multiple other HER2-low cancers (62), including a case report of a patient with HER2-low mCRPC (16), T-DXd has potential to benefit patients with HER2-low mCRPC. Since P-DXd is designed similarly to T-DXd, it may also have efficacy in patients with HER3-low cancers (65). To assess the efficacy of T-DXd and P-DXd in a preclinical mouse model of HER2-low/HER3-low mCRPC with infiltrating *Aspn*^+^ stroma, PC3 xenografts were grown in NOD/SCID-gamma (NSG) mice (Figure 10A). PC3 expressed comparable levels of HER2 and HER3 as MCF7, a HER2-low metastatic breast cancer cell line (Supplemental Figure 2K). Notably, recombinant mouse ASPN induced HER2/HER3 signaling in human PC3 cells in vitro, which could be blocked by tucatinib, supporting the potential for infiltrating mouse *Aspn*^+^ stroma to induce HER2/HER3 signaling in PC3 xenografts (Supplemental Figure 9, A and B). Once PC3 tumors reached approximately 100 mm^3^, mice were randomized and treated with 5 mg/kg P-DXd, 5 mg/kg T-DXd, or vehicle once a week for 4 cycles. Treatment with P-DXd or T-DXd significantly decreased PC3 xenograft growth compared with vehicle without overt toxicity as demonstrated by stable mouse weight (Figure 10, B–D, and Supplemental Figure 9, C–F). Importantly, treatment with T-DXd or P-DXd significantly decreased tumor size from baseline (Figure 10D and Supplemental Figure 9F). Consistent with tumor measurements during therapy, treatment with P-DXd or T-DXd significantly decreased final tumor weight compared with vehicle, with comparable tumor weights between the 2 drug treatment arms (Figure 10, E–G, and Supplemental Figure 9, G–I).

Xenografts were assessed by histology and RNA-Seq at experimental endpoint. Evaluation of tissue by hematoxylin and eosin (H&E) staining indicated that residual disease in P-DXd– or T-DXd–treated mice showed tumor heterogeneity, with a subset of cancer cells having hyperchromatic, irregular, spindled, and pyknotic nuclei and being surrounded by a reactive fibromyxoid stroma, indicative of response to therapy (Figure 10H and Supplemental Figure 9J). IHC showed that vehicle-treated PC3 xenografts were HER2-low and HER3-low with robustly infiltrating *Aspn*^+^ stroma, which is consistent with prostate cancer metastases in patients (Figure 10, H and I, and Supplemental Figure 9, K–N). Membranous expression of HER2 in residual disease following P-DXd was comparable to vehicle. However, cytoplasmic staining was heterogenous with a subset of cancer cells having high expression. In contrast with P-DXd, HER2 expression was decreased and largely cytoplasmic following T-DXd treatment (Figure 10, H and I, and Supplemental Figure 9, K–N). HER3 expression was significantly increased in residual disease following T-DXd or P-DXd when compared with vehicle (Figure 10, H and I, and Supplemental Figure 9M). Vehicle-treated xenografts were strongly associated with *Aspn*^+^ stroma. However, P-DXd– and T-DXd–treated xenografts were heterogenous for the number of infiltrating *Aspn*^+^ stromal cells, with some tumors having a reduction in *Aspn*^+^ stromal cells (Figure 10, H and I, and Supplemental Figure 9N). RNA-Seq followed by GSEA of P-DXd– and T-DXd–treated xenografts compared with vehicle demonstrated increased cell death– and immune-related Hallmarks, such as apoptosis and interferon gamma response, while proliferation-associated Hallmarks, including E2F targets, G2M checkpoint, MYC targets V1, MYC targets V2, and mitotic spindle, were decreased (Figure 10J and Supplemental Figure 9, O and P). Hallmarks were nearly identical between P-DXd– and T-DXd–treated xenografts compared with vehicle. Indeed, only 5 genes were significant for differential expression between P-DXd– and T-DXd–treated xenografts. Similarity in efficacy and gene expression in residual disease between P-DXd and T-DXd is consistent with both ADCs having the same drug conjugate, deruxtecan. Collectively, these findings support that HER2-low and HER3-low prostate cancer cell growth can be effectively targeted with P-DXd and T-DXd despite the presence of ASPN in the TME.

## Discussion

Our study establishes ASPN as a HER3 ligand and a robust activator of HER2/HER3 signaling. Extracellular ASPN leads to increased cellular migration in a HER2-dependent manner, and this phenotype is abrogated by a HER2-targeted therapy. Stromal expression of *ASPN* in the TME of HER2- and HER3-expressing metastatic prostate cancer supports a role for this pathway in metastatic disease and highlights a potential therapeutic vulnerability, especially due to the development of ADCs designed to target HER2 and HER3. Indeed, T-DXd and P-DXd both inhibited the growth of prostate cancer cells in vitro, with significant reduction of tumor size in vivo, despite the presence of stromal *Aspn*. These findings highlight the potential for ADCs targeting HER2 and HER3 to improve outcomes in a substantial number of patients with advanced prostate cancer.

HER2/HER3 have well-established roles in tumor progression and are associated with poor prognosis, metastases, therapy resistance, and decreased survival (5, 66). These receptors are activated by ligands in normal cells, but signaling often becomes dysregulated in cancers through receptor mutation or amplification (67). Although genomic alterations in *ErbB* genes are uncommon in prostate cancer, recent reports suggest HER2 and HER3 have important roles in advanced-stage disease. Specifically, HER2 has been shown to increase ligand-independent activation of AR (68, 69), transactivation of AR (70), and cancer cell growth at metastatic sites (59). Studies also indicate HER2 and HER3 have increased expression in mCRPC and are associated with worse outcomes (11, 57–59, 71). HER2 and HER3 activation may be mediated in part through NRG1β, a HER3 ligand (11, 21). However, mechanisms driving HER2 and HER3 activation in metastatic prostate cancers are not fully known. Our study identifies ASPN as a ligand of HER3 and activator of HER2/HER3 signaling in prostate cancer. Our research supports that stroma-secreted ASPN binds to HER3 on adjacent prostate cancer cells and induces phosphorylation of its preferential dimerization partner, HER2, thereby activating multiple ErbB-associated downstream signaling pathways and increasing cellular migration. ASPN induces overlapping but distinct signaling compared with NRG1β, with notable differences in EGFR activation. While NRG1β induces significant phosphorylation of EGFR, ASPN activation of EGFR is limited, suggesting that ASPN-induced ErbB signaling in prostate cancer is primarily through HER2 and HER3. In addition to signaling variances, ASPN and NRG1β have noteworthy expression divergences. NRG1 expression was reported to be absent in therapy-naive localized prostate cancer but induced in the stroma following neoadjuvant ADT in a small subset of patients (21). Consistent with a prior report, tumor stroma showed minimal expression of *NRG1* in prostate cancer metastases, while a small subset of metastatic prostate cancer cells expressed NRG1 (11). In contrast, stromal expression of ASPN is associated with Grade Group of localized prostate cancer (24, 28) and was detected in over 70% of mHSPC and mCRPC samples within our patient cohort. Importantly, over half of metastatic sites demonstrated expression of HER2 and HER3 with stromal expression of *ASPN*, suggesting this signaling mechanism may have critical roles in metastatic prostate cancer.

HER2 has been proposed as a rational therapeutic target because of its association with advanced-stage prostate cancer; however, prior clinical trials with anti-HER2 therapies, including lapatinib, pertuzumab, and trastuzumab, have shown limited clinical benefit (48–52, 72, 73). Potential reasons for lack of therapeutic efficacy in prostate cancer include low recruitment size, heavily pretreated patients, and failure to assess HER2 expression. Consistent with prior clinical trials, our studies showed monoclonal antibodies targeting HER2 were not effective at restricting prostate cancer cell growth in vitro. Thus, both preclinical studies and clinical trials indicate that monoclonal antibodies targeting HER2 lack efficacy for the treatment of advanced prostate cancer. In contrast with monoclonal antibodies, our findings highlight the potential for certain ADCs to have clinical efficacy in advanced prostate cancer. T-DXd is an ADC consisting of a monoclonal antibody against HER2 (trastuzumab), a cleavable linker, and a cytotoxic topoisomerase I inhibitor (deruxtecan). T-DXd is proposed to function by engaging the HER2 receptor to promote ADC internalization and targeted payload release. Intriguingly, T-DXd has been shown to have clinical efficacy against a broad range of tumor types with varied HER2 expression. In metastatic breast cancer, T-DXd is currently approved for HER2^+^ (IHC 3^+^ or IHC 2^+^/ISH^+^), HER2-low (IHC 1^+^ or IHC 2^+^/ISH^–^), and HER2-ultralow (IHC 0 with membrane staining) cancers, with HER2-ultralow and HER2-low breast cancer patients demonstrating similar benefit (60, 61). Results from the phase II DESTINY-PanTumor02 clinical trial suggest T-DXd has therapeutic utility in other advanced HER2^+^ and potentially HER2-low and HER2-ultralow cancers, including endometrial, cervical, ovarian, and bladder (62). Although prostate cancer patients were not included in the DESTINY-PanTumor02 clinical trial, a recent case report supports that T-DXd may also have efficacy in patients with HER2-low mCRPC (16). Potential reasons driving the broad clinical efficacy of T-DXd in patients with low or heterogeneous HER2 expression may be the membrane permeability of the payload following intracellular cleavage and subsequent diffusion into neighboring cells or the ability of extracellular proteases in the TME, such as cathepsins, to cleave the ADC linker and facilitate payload release (74). These findings potentially reduce the requirement of HER2 expression for T-DXd payload release and suggest T-DXd, and other similarly designed ADCs, may have even broader tumor-agnostic implications than previously determined. Overall, these findings suggest certain ADCs may have therapeutic potential in multiple solid tumors, including metastatic prostate cancer.

Our studies showed that T-DXd and P-DXd inhibited HER2-low/HER3-low prostate cancer cells in vitro with nearly identical efficacy in a preclinical in vivo model. Our data indicate that 79% of metastatic prostate cancer samples were HER2-low/ultralow while 82% of patients had HER3-low/ultralow metastases, which is consistent with prior reports (57, 59, 75). Interestingly, HER2 expression was enriched in prostate cancer metastases in soft tissue compared with bone. However, the impact of differential fixation methods between soft tissue and bone on HER2 antibody staining is uncertain but may have implications for the optimal site of biopsy for clinical HER2 assessment. Despite this variable, HER2 and HER3 were expressed in most prostate cancer metastases, which suggests that T-DXd and P-DXd have potential to benefit a substantial number of patients with metastatic prostate cancer. Our work also underscores the potential impact of ASPN in the TME on the efficacy of HER2/HER3-targeted therapies. Structural studies of ASPN binding to HER3 provide insights for therapeutic response and resistance, especially for HER3-targeted therapies. Computational binding predictions suggest ASPN binds to the ligand binding domain of HER3. While NRG1β fits within the HER3 ligand binding pocket, ASPN is larger, and modeling indicates that it extends beyond the pocket and may therefore obstruct a substantial portion of HER3. P-DXd targets the extracellular domain of HER3. However, its binding epitope is not well described (76). Interestingly, P-DXd shows in vitro and in vivo efficacy, even in the presence of *Aspn*^+^ cancer-associated stromal cells, suggesting that P-DXd may have a higher affinity to HER3 than ASPN or the P-DXd binding epitope is not obstructed by ASPN binding. T-DXd and DV bind to HER2 at domain IV (77) while tucatinib binds to the intracellular kinase domain of HER2 (78). Based on computational studies, ASPN-HER3 binding should not interfere with HER2 inhibition by T-DXd, DV, or tucatinib. Consistent with this, our studies demonstrate that T-DXd, DV, and tucatinib inhibit prostate cancer cell growth despite the presence of ASPN and suggest anti-HER2 therapies could have clinical efficacy, even with ASPN in the TME. Although these therapies demonstrate efficacy in the presence of ASPN, future studies are needed to evaluate efficacy in patients with metastatic prostate cancer.

The role of ASPN-induced activation of HER2/HER3 in prostate cancer has potential for translation to other solid tumors. ASPN has previously been shown to increase tumor progression in multiple cancer types, including pancreatic (35), colorectal (79), gastric (37), breast (80), and bladder (81). While previous studies have explored the mechanistic role of ASPN in other cancer-related pathways, including CD44 (37), TGF-β (34), and EGFR (79), to the best of our knowledge, our study is the first to report extracellular ASPN directly binds to HER3 and activates signaling through heterodimerization with HER2. Future investigations are necessary to determine whether ASPN-induced activation of HER2/HER3 is present in other tumor types and has therapeutic potential.

In summary, our data identify ASPN as a stroma-secreted HER3 ligand that promotes prostate cancer cell migration through HER2/HER3 signaling. ADCs designed to target HER2 and HER3 diminish prostate cancer growth. These findings provide rationale for further studies to determine the efficacy of therapeutically targeting ASPN-HER2/HER3 signaling in patients with mCRPC and potentially other solid tumor types.

## Methods

### Sex as a biological variable.

For patient samples our study exclusively examined metastatic prostate cancer tissue from male patients because prostate cancer is a biologic male–specific cancer. Metastatic prostate cancer biopsies or surgical resections at Vanderbilt University Medical Center with evaluable tissue were identified from 33 patients between 2015 and 2023. H&E slides from all cases were reviewed by a genitourinary pathologist. All cases have associated deidentified clinical data obtained from electronic medical records.

For animal studies our study exclusively examined male mice because prostate cancer is a biologic male–specific cancer. Male NOD.Cg-*Prkdc^scid^* *Il2rg^tm1Wjl^*/SzJ (NSG) mice were purchased from The Jackson Laboratory and maintained under sterile housing conditions.

### Procedures.

For initial in vivo assessment of T-DXd, 1 × 10^6^ PC3 cells in a 1:1 mixture of ice-cold PBS and Matrigel (Corning) were subcutaneously injected into the flanks of 11-week-old NSG male mice (100 μL/mouse) (*n* = 20). Mice were monitored and tumor measurements were obtained 3 times a week using electronic calipers once tumors were palpable. Tumor volume was calculated by [(length)^2^ × width]/2, where length represents the longest tumor measurement. When tumors were approximately 90 mm^3^, mice were randomized and treated with 10 mg/kg T-DXd (Selleck Chem) or vehicle control (PBS) once a week via retro-orbital injection for 5 cycles. One T-DXd–treated mouse died prior to experimental endpoint from causes unrelated to the tumor or therapy. To compare T-DXd and P-DXd in vivo, 1 × 10^6^ PC3 cells in a 1:1 mixture of ice-cold PBS and Matrigel (Corning) were subcutaneously injected into the flanks of 10-week-old NSG male mice (100 μL/mouse) (*n* = 21). Mice were monitored and tumors were measured 3 times a week using electronic calipers once tumors were palpable. When tumors were approximately 100 mm^3^, mice were randomized and treated with 5 mg/kg P-DXd (Selleck Chem), 5 mg/kg T-DXd (AstraZeneca), or vehicle control (PBS) once a week via retro-orbital injection for 4 cycles. One mouse died prior to treatment from causes unrelated to the tumor or therapy, and 1 mouse was excluded from the final analyses due to the lack of a palpable tumor at treatment initiation. In both experiments, tumors were harvested for analysis at experimental endpoint. Tumors were weighed, photographed, fixed in 10% neutral buffer formalin overnight with gentle rocking, paraffin-embedded, and then sectioned. Tumor sections were stained by H&E following manufacturer’s instructions (Abcam). Tumor sections were also examined by dual IHC/RNAscope for HER2 and ASPN and by IHC for HER3 as described above. Tumors from vehicle, 5 mg/kg T-DXd, and 5 mg/kg P-DX-d mice were also analyzed by RNA-Seq. In brief, tumor tissue (30 mg or less) was placed in a round-bottom Eppendorf and lysed on a TissueLyser II machine (QIAGEN) for 2 minutes at 30 Hz with 350 μL Buffer RLT plus 0.5% v/v Reagent Dx (QIAGEN) and a 5 mm stainless steel bead (QIAGEN) per tube. Total RNA was extracted from the homogenized lysate using the RNeasy Protect Cell Mini Kit (QIAGEN) as per manufacturer’s protocol. RNA was analyzed by RNA-Seq as described in Supplemental Methods.

### Statistics.

Statistical comparisons between 2 groups were performed using a 2-tailed Student’s *t* test. Statistical comparisons between 2 categorical variables were performed using a Fisher’s exact test. Statistical comparisons between multiple groups were performed using 1-way ANOVA with Tukey’s, Dunnett’s, or Šídák’s post hoc analysis, as indicated in the figure legends. IC_50_ calculations were determined by graphing drug concentration versus cell viability and analyzed with nonlinear regression ([inhibitor] versus response, variable slope, 4 parameters, with interpolation). Only *P* values less than 0.05 were considered statistically significant. *P* values were indicated with asterisks in the figures. All statistical analyses were performed using GraphPad Prism Software.

### Study approval.

The human study protocol was approved by the Vanderbilt University Medical Center IRB. All animal experiments were performed under a Vanderbilt University Medical Center–approved IACUC protocol.

### Data availability.

Methods pertaining to cell lines, recombinant protein, RNA-Seq, immunoblotting, computational binding prediction, PLA, co-immunoprecipitation, gene targeting with CRISPR/Cas9, cell migration assays, IHC, RNA-Protein Integrated Co-detection (RNAscope with IHC), RNA ISH, and inhibitor assays can be found in the Supplemental Methods.

Gene expression data generated in this study are publicly available in National Center for Biotechnology Information (NCBI) Gene Expression Omnibus (GEO) at GSE271579, GSE271580, GSE271738, GSE271739, GSE271740, GSE271742, and GSE284054. Supporting data values from the main manuscript and the supplement are available in a single Excel file titled Supporting Data Values in the supplement.

## Author contributions

Conception and design were contributed by ABH, HYW, BPB, JM, KRS, JBG, BHP, and PJH. Data acquisition and analysis were contributed by ABH, HYW, JJ, MA, BLR, EW, REB, BAD, SEG, DG, EFN, JAG, LBM, VS, PIGE, QS, BPB, KRS, JBG, BHP, and PJH. Data interpretation was contributed by all authors. Manuscript preparation was contributed by all authors. ABH is listed as the first co–first author due to her higher level of contribution to conception, design, data interpretation, and manuscript preparation.

## Supplementary Material

Supplemental data

Unedited blot and gel images

Supporting data values

## Version 1 08/22/2025

Electronic publication
