# Supplementary material for: Identification of asporin as a HER3 ligand exposes a therapeutic vulnerability in prostate cancer
Source: JCI Insight. 2025 Aug 22;10(16):e187151. doi: 10.1172/jci.insight.187151 (PMC12406728; doi:10.1172/jci.insight.187151)
Supplement: Supplemental data [file jciinsight-10-187151-s057.pdf]

TITLE: Identification of Asporin as a HER3 ligand exposes a therapeutic vulnerability in prostate cancer

AUTHORS: Amanda B. Hesterberg, Hong Yuen Wong, Jorgen Jackson, Monika Antunovic, Brenda L. Rios, Evan Watkins, Riley E. Bergman, Brad A. Davidson, Sarah E. Ginther, Diana Graves, Elliott F. Nahmias, Jared A. Googel, Lillian B. Martin, Violeta Sanchez, Paula I. Gonzalez-Ericsson, Quanhu Sheng, Benjamin P. Brown, Jens Meiler, Kerry R. Schaffer, Jennifer B. Gordetsky, Ben H. Park, Paula J. Hurley

SUPPLEMENTAL FIGURES:

Supplemental Figure 1 | Altered GSEA Oncogenic Signatures in ASPN treated prostate cancer cells.

Supplemental Figure 2 | ASPN induced HER2/HER3 signaling in prostate cancer cells.

Supplemental Figure 3 | Altered GSEA Oncogenic Signatures in NRG1β treated prostate cancer cells.

Supplemental Figure 4 | ASPN binds to the ligand binding domain of HER3.

Supplemental Figure 5 | HER2 and HER3 are key mediators of ASPN signaling.

Supplemental Figure 6 | Patient characteristics and antibody controls for HER2, ASPN, HER3, and NRG1β.

Supplemental Figure 7 | Prostate cancer cell response to therapies designed to target either HER2 or HER3.

Supplemental Figure 8 | ASPN does not restrict the in vitro efficacy of therapies designed to target HER2 or HER3 in prostate cancer cells.

Supplemental Figure 9 | Antibody drug conjugate designed to target HER2 (T-DXd) restricts growth of prostate cancer cells in vivo.

SUPPLEMENTAL TABLE:

Supplemental Reagents Table

SUPPLEMENTAL METHODS:

Cell lines
Recombinant protein
RNA sequencing
Immunoblotting
Computational binding prediction
Proximity ligation assay
Co-immunoprecipitation
Gene targeting with CRISPR-Cas9
Cell migration assays
Immunohistochemistry
RNA-protein integrated co-detection (RNAScope® with IHC)
RNA in situ hybridization
Inhibitor assays

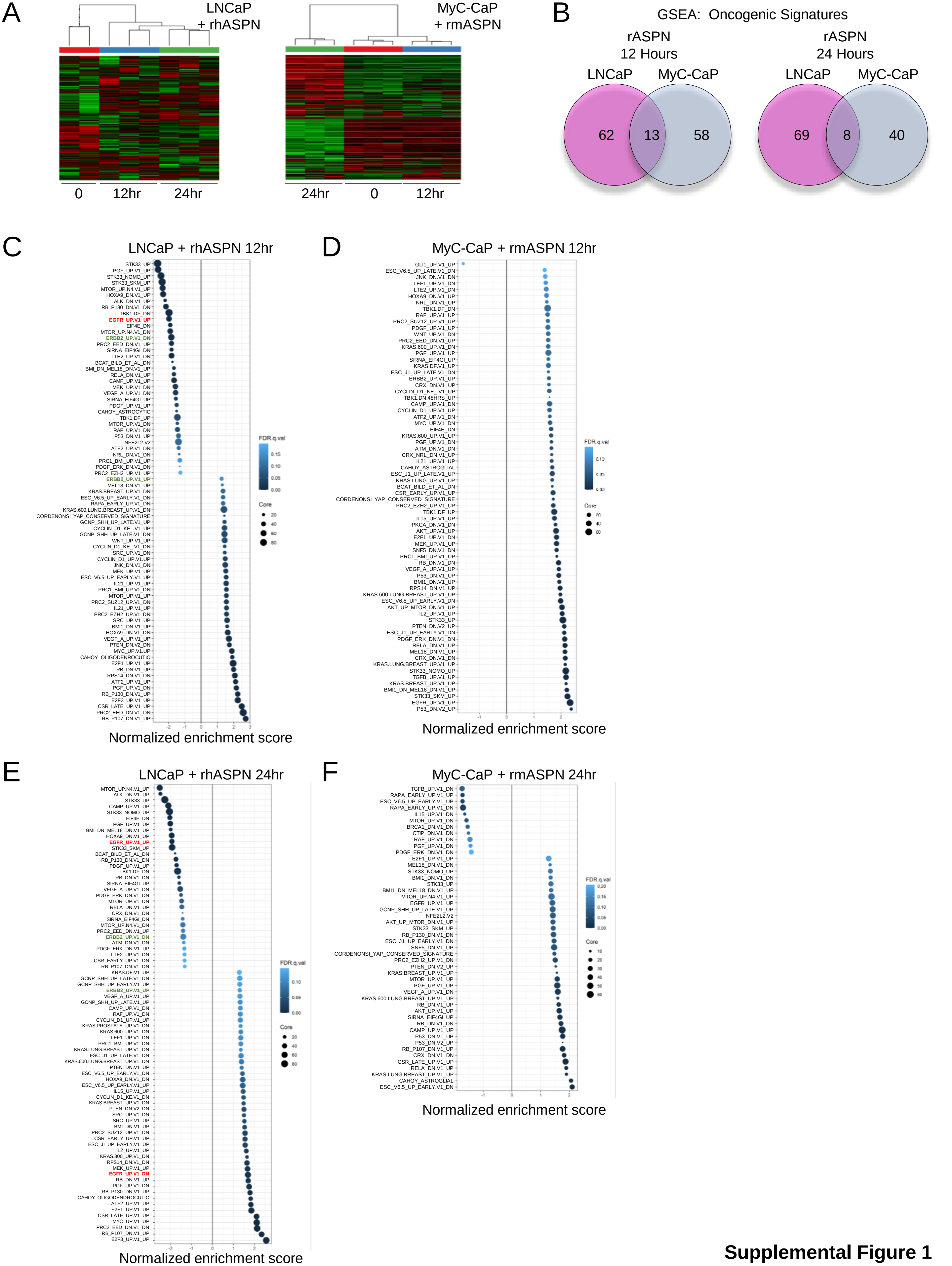

Supplemental Figure 1

Supplemental Figure 1 | Altered GSEA Oncogenic Signatures in ASPN treated prostate cancer cells. A, Heatmaps of LNCaP and MyC-CaP cells treated with 100 ng/mL recombinant human or mouse ASPN for 0, 12, and 24 hours and assessed by RNA-Seq (n=2-3). **B,** Venn Diagram of Oncogenic Signatures by GSEA from RNA-Seq of LNCaP and MyC-CaP cells treated with 100 ng/ml recombinant ASPN at 12 (left) and 24 (right) hours compared to 0 (n=2-3). **C-F,** Bubble plots of Oncogenic Signatures by GSEA from RNA-Seq of LNCaP and MyC-CaP cells treated with 100 ng/ml recombinant ASPN for 12 (C, D) and 24 (E, F) hours compared to time 0 (n=2-3).

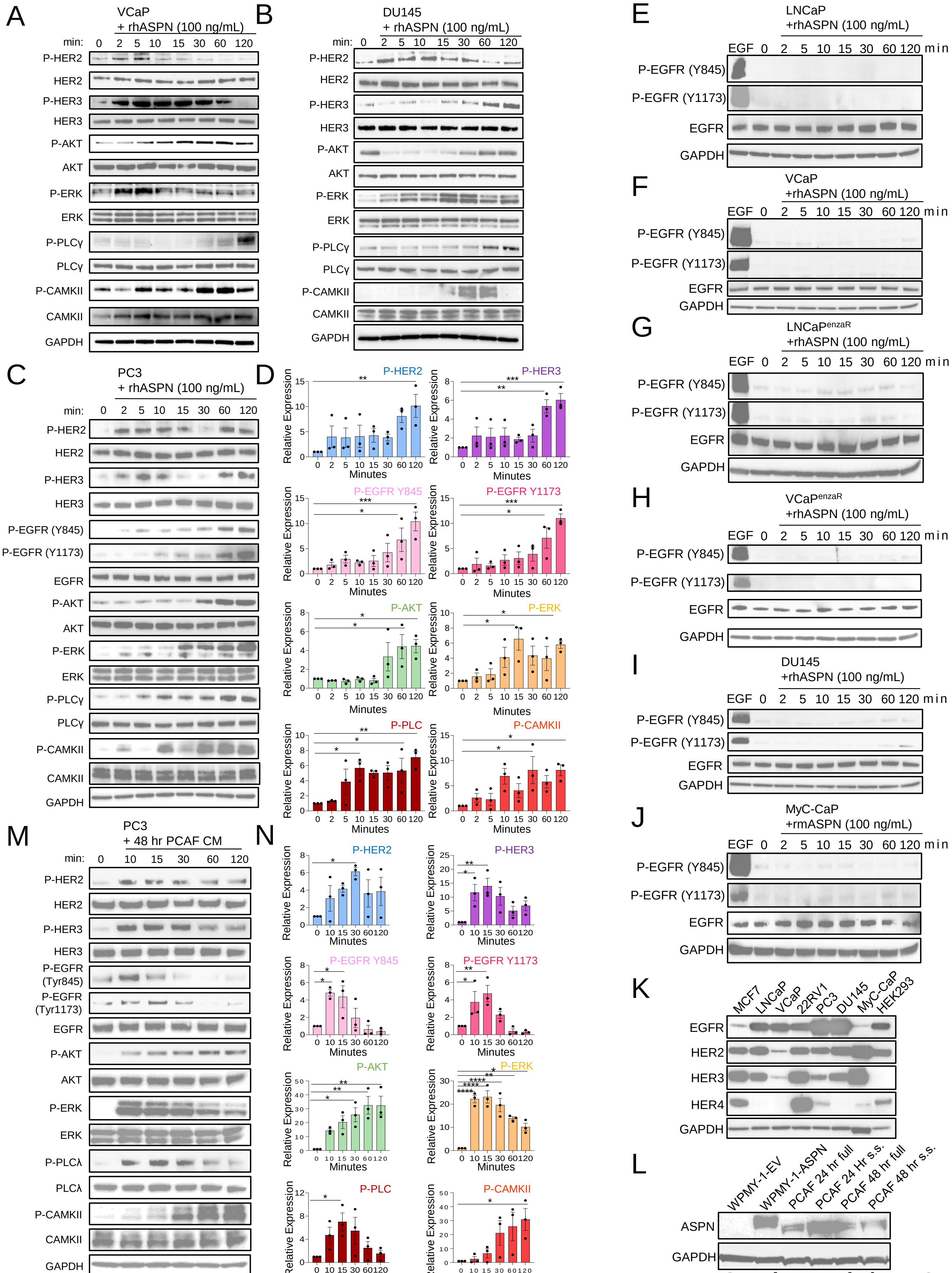

Supplemental Figure 2

Supplemental Figure 2 | ASPN induced HER2/HER3 signaling in prostate cancer cells. A-D, VCaP (A), DU145 (B), and PC3 (C) cells treated with 100 ng/ml recombinant human ASPN over a time course and assessed by immunoblotting for HER2 and HER3 pathway activation (n=2-3). Quantification of PC3 cells in D (n=3). **E-J,** LNCaP (E), VCaP (F), LNCaP^{enzaR} (G), VCaP^{enzaR} (H), DU145 (I), and MyC-CaP (J) treated with 10 ng/mL recombinant human or mouse EGF for 10 minutes or 100 ng/mL human or mouse ASPN over a time course and assessed by immunoblotting for EGFR activation. **K,** MCF7, LNCaP, VCaP, 22RV1, PC3, DU145, MyC-CaP, and HEK293 assessed by immunoblotting for ErbB family members. **L,** Benign prostate fibroblast cell line (WPMY-1) transfected with empty vector (WPMY-1-EV) or ASPN-3XFLAG (WPMY-1-ASPN-FLAG) and patient derived prostate CAF (PCAF) in full serum (full) and serum starved (s.s.) conditions were assessed by immunoblotting for ASPN. **M-N,** PC3 cells treated with conditioned media (CM) from 48-hour serum starved ASPN expressing PCAF and assessed by immunoblotting for HER2 and HER3 pathway activation (M) followed by quantification (N) (n=3). Total proteins normalized to GAPDH, and phosphorylated proteins normalized to GAPDH and total protein. Graphs are shown as mean ± SEM and analyzed by One-way ANOVA with Dunnet post hoc analysis; *P≤0.05, **P≤0.01, ***P≤0.001 ****P≤0.0001.

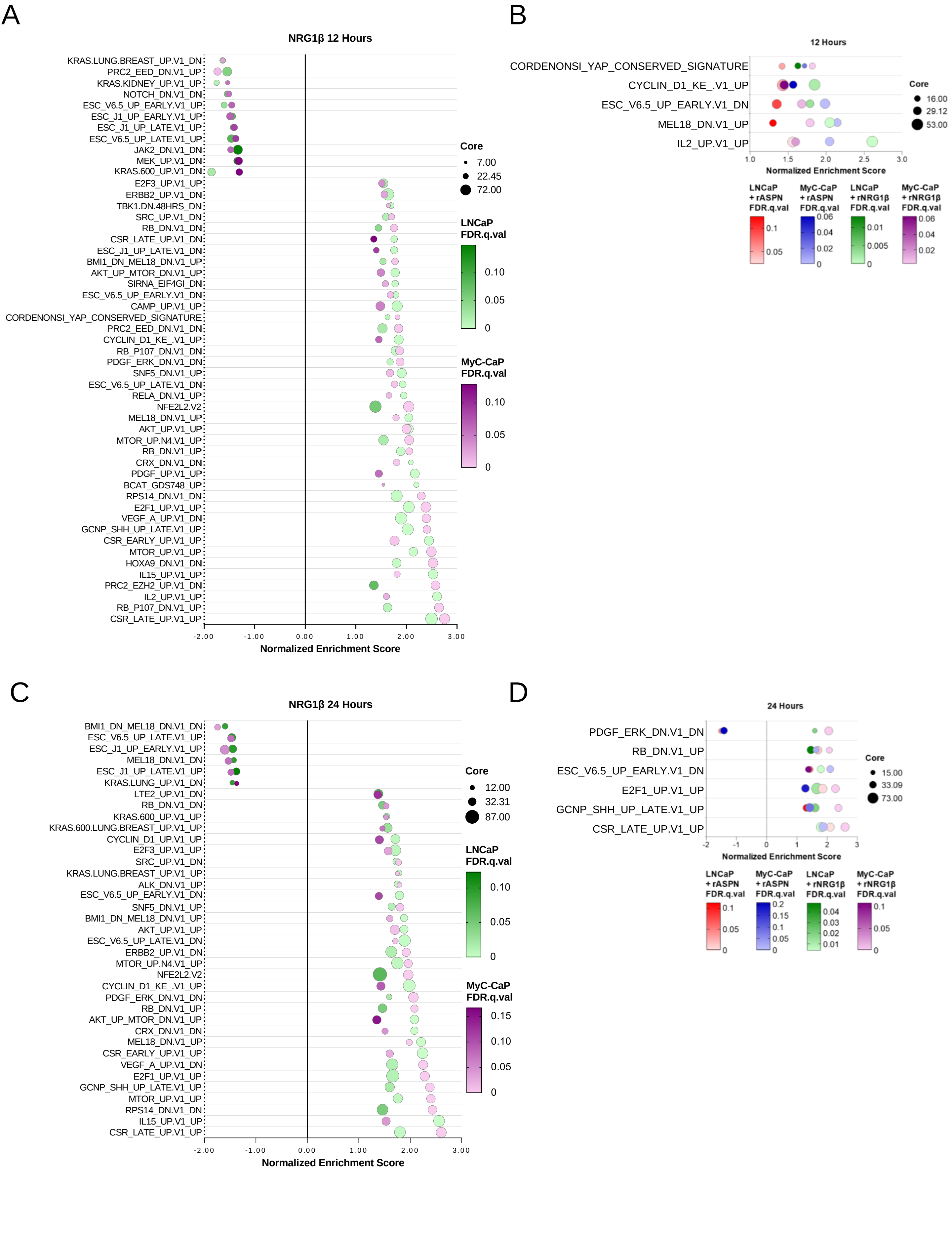

Supplemental Figure 3

Supplemental Figure 3 | Altered GSEA Oncogenic Signatures in NRG1β treated prostate cancer cells. A, Bubble plots of overlapping Oncogenic Signatures by GSEA from RNA-Seq of LNCaP and MyC-CaP cells treated with 15 ng/mL recombinant human or mouse NRG1β for 12 hours compared to time 0 (n=3). **B,** Overlapping Oncogenic Signatures by GSEA from RNA-Seq of LNCaP and MyC-CaP cells treated with either 15 ng/mL rNRG1β or 100 ng/mL rASPN for 12 hours compared to time 0 (n=2-3). **C,** Bubble plots of overlapping Oncogenic Signatures by GSEA from RNA-Seq of LNCaP and MyC-CaP cells treated with 15 ng/mL recombinant human or mouse NRG1β for 24 hours compared to time 0 (n=3). **D,** Overlapping Oncogenic Signatures by GSEA from RNA-Seq of LNCaP and MyC-CaP cells treated with either 15 ng/mL rNRG1β or 100 ng/mL rASPN for 24 hours compared to time 0 (n=2-3).

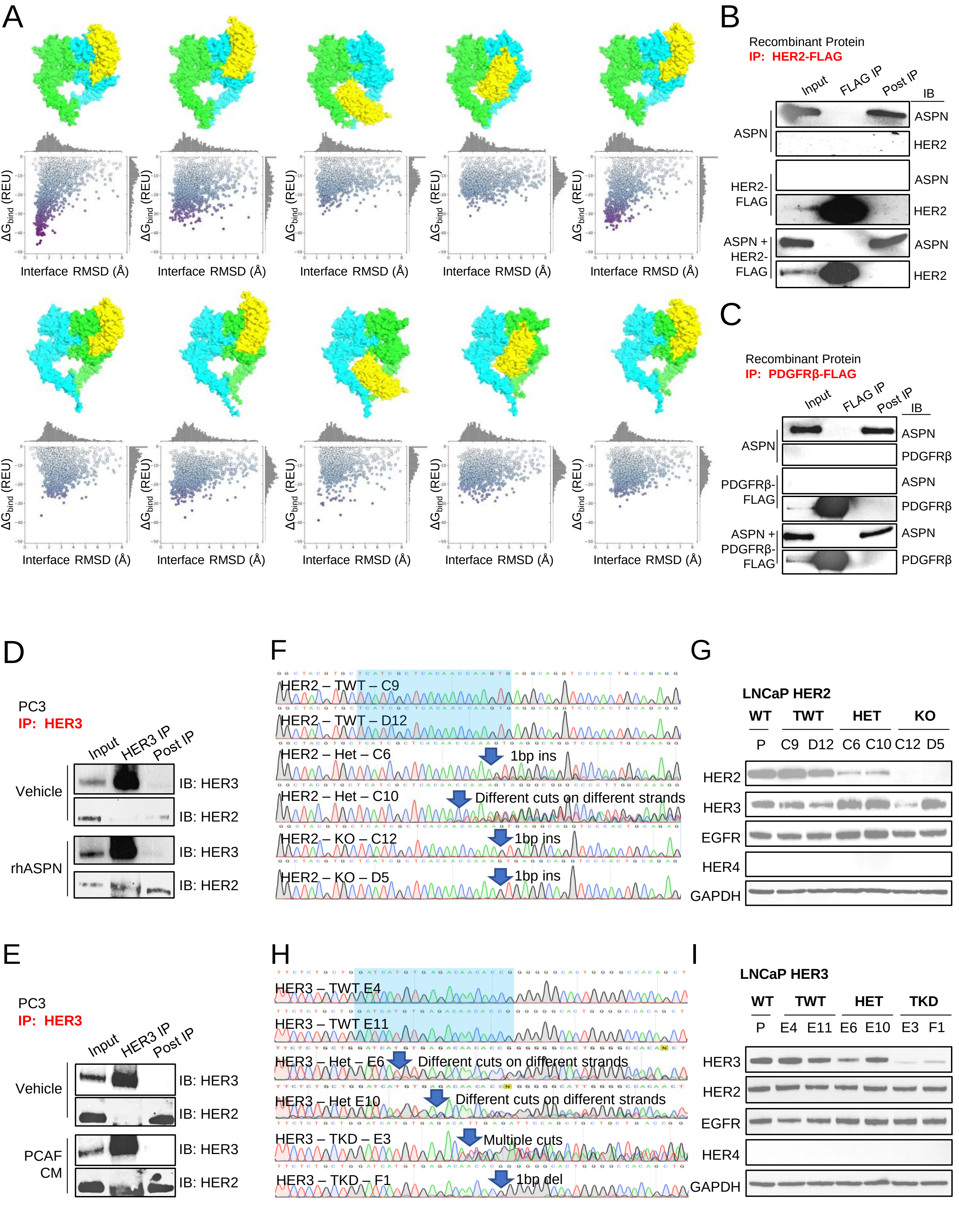

Supplemental Figure 4

Supplemental Figure 4 | ASPN binds to the ligand binding domain of HER3. A, Rosetta local docking funnel for ASPN (yellow) against the HER2 (green) / HER3 (blue) extracellular domain heterodimer of the AlphaFold2-predicted complex. Scatter plot colored by Rosetta binding energy in Rosetta energy units (REU) using the REF2015 score function. 1D histograms represent the marginal distributions for the interface RMSD (top) and binding energy (bottom). Left top also shown in Figure 4A and included here for comparison. **B, C,** Recombinant human ASPN and recombinant human HER2-FLAG (B) or recombinant human PDGFRβ-FLAG (C) were incubated either with vehicle or together in a cell-free assay, then immunoprecipitated with anti-FLAG beads, and assessed for ASPN and HER2 (B) or PDGFRβ (C) by immunoblotting (n=2). **D,** PC3 cells were treated with vehicle or recombinant human ASPN (100 ng/mL). Cell lysates were normalized and incubated with an anti-HER3 antibody overnight. HER3 was immunoprecipitated using Protein A Agarose beads, washed, and analyzed by immunoblotting for HER3 and HER2 (n=2). **E,** PC3 cells were treated with vehicle or conditioned media (CM) from ASPN-expressing prostate cancer associated fibroblasts (PCAF)**.** Cell lysates were normalized and incubated with an anti-HER3 antibody overnight. HER3 was immunoprecipitated using Protein A Agarose beads, washed, and analyzed by immunoblotting for HER3 and HER2 (n=2). **F,** DNA sequencing traces of HER2 targeted wild type (TWT), HER2 heterozygous knock out (HET), and HER2 knockout (KO) LNCaP independent clones. **G**, ErbB family member expression in HER2 TWT, HER2 HET, and HER2 KO LNCaP independent clones as assessed by immunoblotting. **H,** DNA sequencing traces of HER3 TWT, HER3 HET, and HER3 truncated knockdown (TKD) LNCaP independent clones. **I**, Expression of ErbB family members in HER3 TWT, HER3 HET, and HER3 TKD LNCaP independent clones as assessed by immunoblotting.

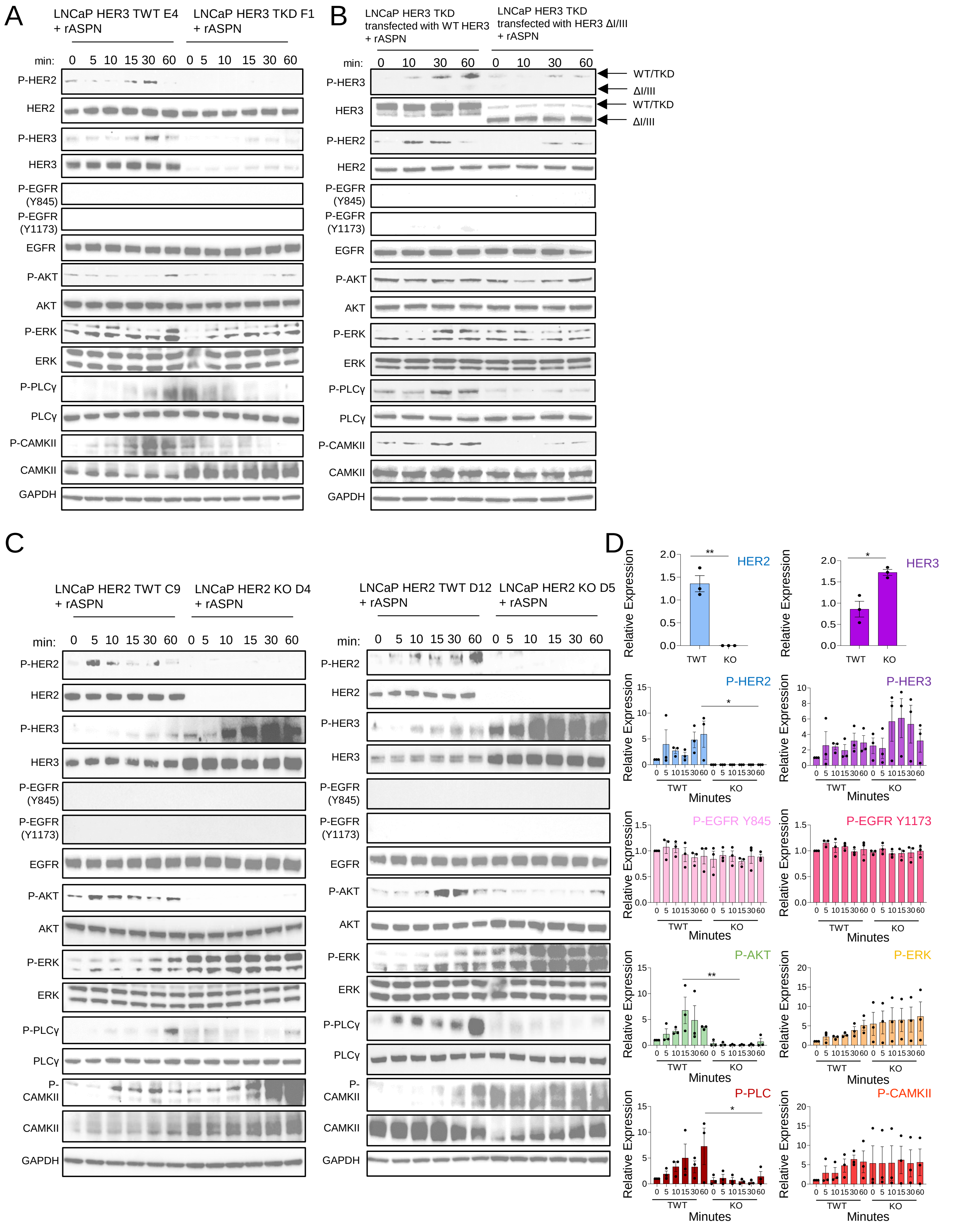

Supplemental Figure 5

Supplemental Figure 5 | HER2 and HER3 are key mediators of ASPN signaling. A, LNCaP targeted wild type (TWT) and LNCaP HER3 truncated knockdown (TKD) cells were treated with 100ng/ml recombinant human ASPN over a time course and assessed by immunoblotting for HER2 and HER3 pathway activation. **B,** LNCaP HER3 TKD cells were transfected with HER3 WT or HER3 ΔI/III mutant then treated with 100ng/ml recombinant human ASPN over a time course and assessed by immunoblotting for HER2 and HER3 pathway activation. **C, D,** LNCaP TWT and LNCaP HER2 KO (with increased baseline HER3 expression) were treated with 100 ng/ml recombinant human ASPN over a time course and assessed by immunoblotting for HER2 and HER3 pathway activation (C) and quantification (D) (n=3). Graphs are shown as mean ± SEM and analyzed by One-way ANOVA with Sidak's post hoc analysis; *P≤0.05, **P≤0.01.

A

Clinical Characteristics of Prostate Cancer Metastases (n=33)	
**Age (years), Median (range)**	72 (59-86)
**Year, Median (range)**	2019 (2015-2023)
**Race**	
**White, Non-Hispanic**	29 (88%)
**White, Hispanic**	1 (3%)
**Black, Non-Hispanic**	3 (9%)
**PSA (ng/mL), Median (range)**	13.7 (<0.1->5,000)
**Site of Sampled Metastasis**	
**Bone**	11 (33%)
**Lung**	7 (21%)
**Brain**	8 (24%)
**Liver**	3 (9%)
**Other**	4 (12%)
**Androgen Responsive**	
**mHSPC**	11 (33%)
**mCRPC**	19 (58%)
**mNEPC/SCPC/BCPC**	3 (9%)
**Systemic Therapies**	
**Castrate: Medically or Surgically**	25 (76%)
**NHT**	19 (58%)
**Chemotherapy**	8 (24%)
**Lines of Systemic Therapies**	
**0**	8 (24%)
**1**	3 (9%)
**2**	10 (30%)
**≥3**	12 (36%)

B Breast Cancer Controls for HER2 Grading

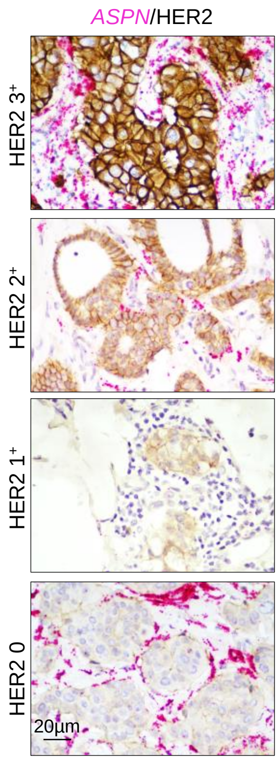

C Benign Host Tissue Adjacent to Prostate Cancer Metastasis

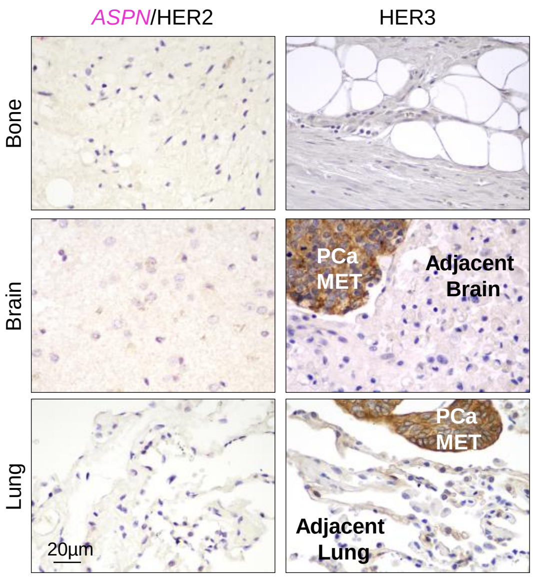

D Prostate Cancer Metastases

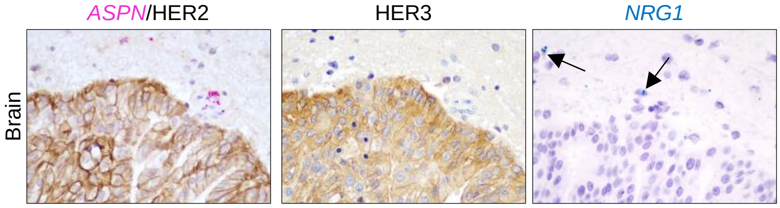

E

**HER2 and HER3 expression by IHC (1+, 2+) in prostate cancer metastases (bone, lung, brain, liver, lymph node, and other).**				
**Hormone Status**	**HER2-low 1+ or 2+ ≥10% (%)**	**HER2-ultralow 1+ at <10% (%)**	**HER3-low 1+ or 2+ ≥10% (%)**	**HER3-ultralow 1+ at <10% (%)**
**mHSPC**	7/11 (64%)	2/11 (18%)	9/11 (82%)	1/11 (9%)
**mCRPC**	15/22 (68%)	2/22 (9%)	16/22 (73%)	1/22 (5%)
**Total**	22/33 (67%)	4/33 (12%)	25/33 (76%)	2/33 (6%)

F

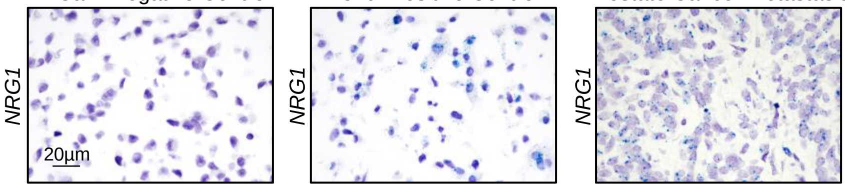

Supplemental Figure 6

Supplemental Figure 6 | Patient characteristics and antibody controls for HER2, ASPN, HER3, and NRG1β. A, Clinical characteristics of prostate cancer metastases (n=33). **B,** Representative images of dual IHC/RNAscope for HER2/*ASPN* on breast cancer control tissues with validated clinical HER2 IHC scores of 0, 1^+, 2^+, and 3^+. **C,** Representative images of dual IHC/RNAscope for HER2/*ASPN* on benign tissue adjacent to prostate cancer metastases. **D,** Representative images of distant prostate cancer metastases analyzed by dual IHC for HER2 and RNAscope for *ASPN* (n=33), IHC for HER3 (n=33), and RNAscope for *NRG1* (n=31). **E,** HER2 expression by IHC (1^+, 2^+) in prostate cancer metastases. **F,** Representative images of RNAscope for *NRG1* in a negative control (LNCaP), a positive control (A549), and a positive prostate cancer metastasis.

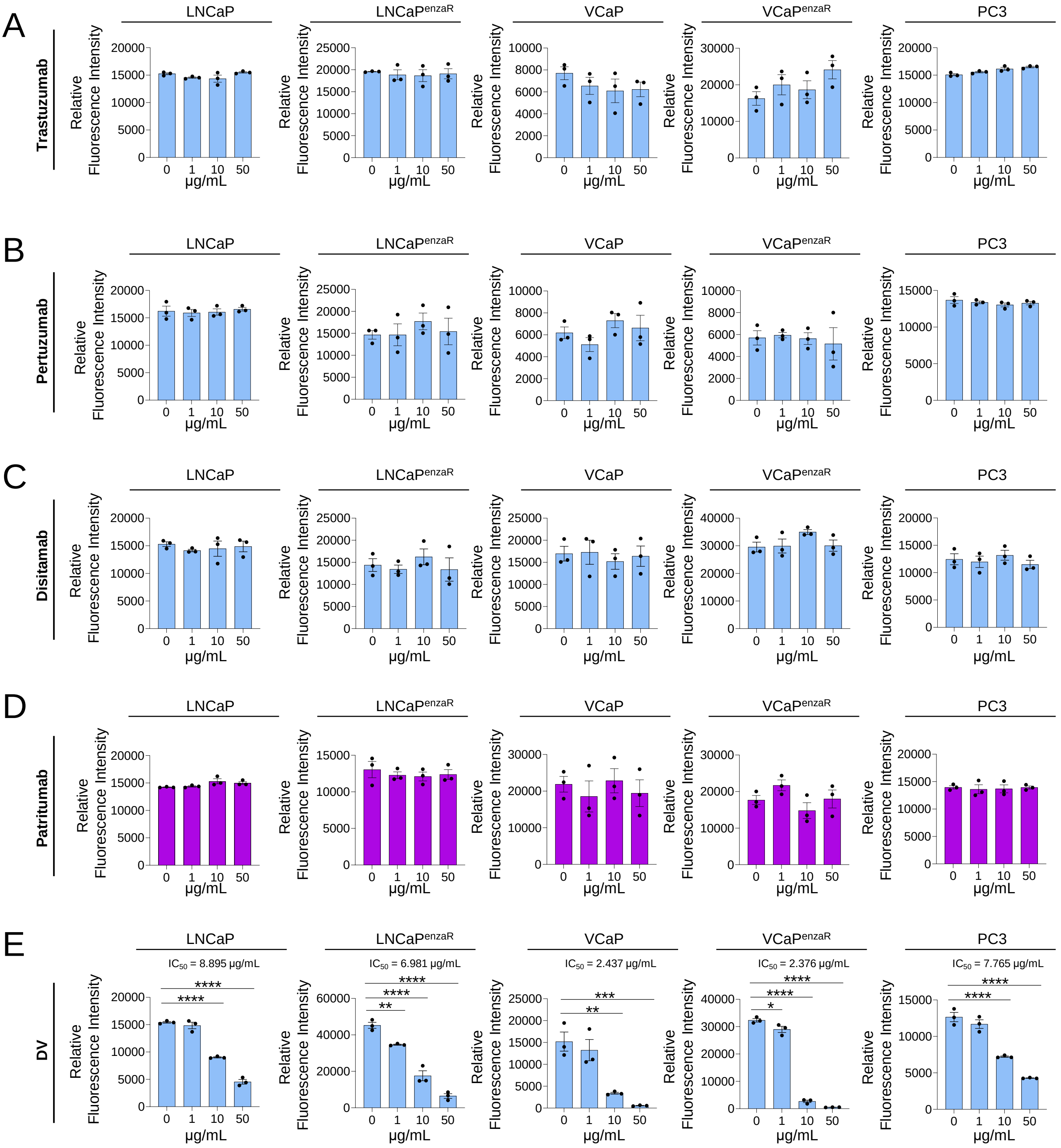

Supplemental Figure 7

Supplemental Figure 7 | Prostate cancer cell response to therapies designed to target either HER2 or HER3. A-E, LNCaP, $LNCaP^{enzaR}$, VCaP, $VCaP^{enzaR}$ and PC3 cells treated with increasing concentrations of trastuzumab (A), pertuzumab (B), disitamab (C), patritumab (D), or disitamab vedotin (DV) (E) for 5 days (LNCaP, PC3) or 10 days ($LNCaP^{enzaR}$, VCaP, $VCaP^{enzaR}$). Inferred quantification of cell number by relative fluorescence intensity using CyQuant (n=3). Graphs are shown as mean ± SEM and analyzed by One-way ANOVA with Dunnett's post hoc analysis; *P≤0.05, **P≤0.01, ***P≤0.001, ****P≤0.0001.

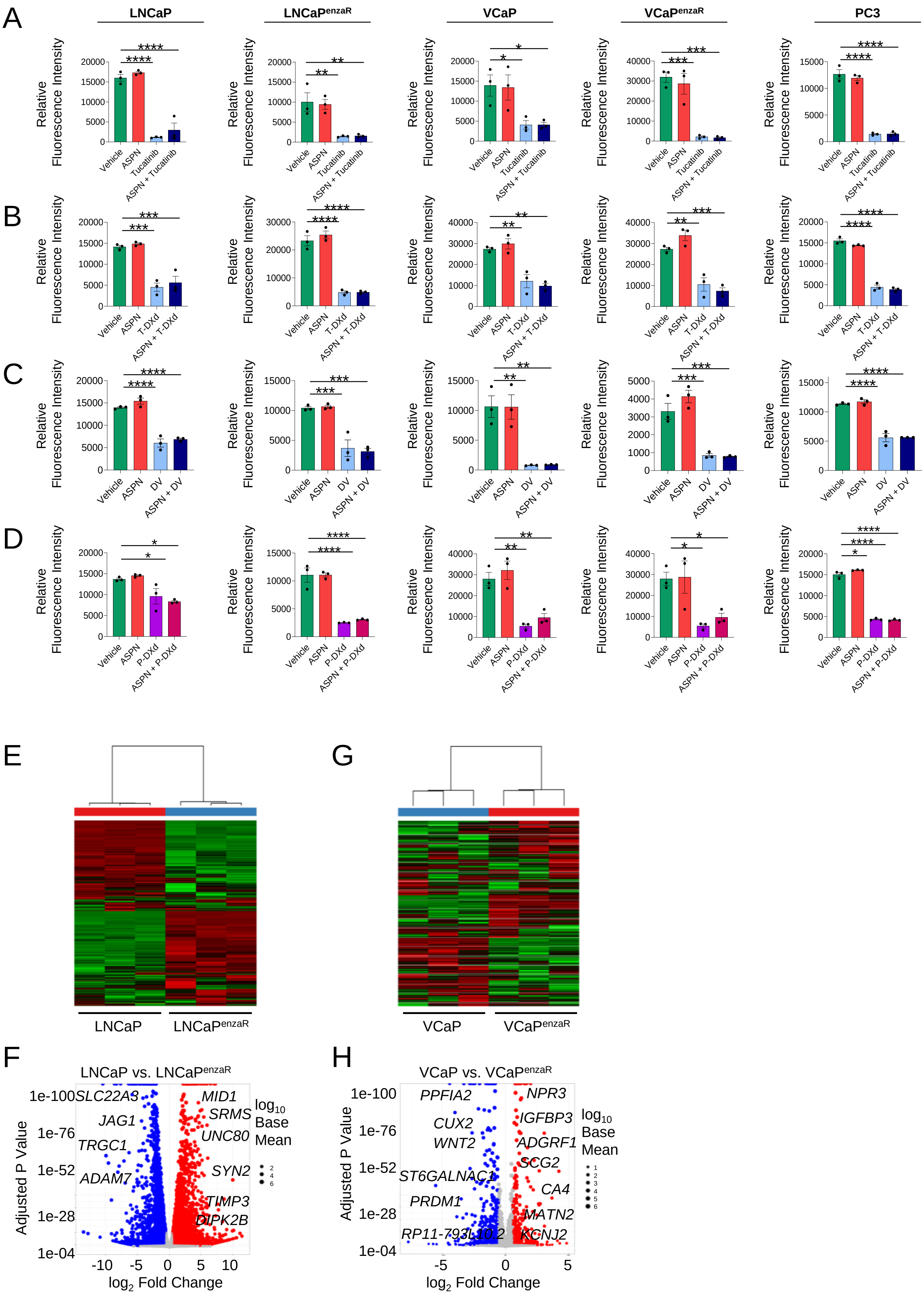

Supplemental Figure 8

Supplemental Figure 8 | ASPN does not restrict the in vitro efficacy of therapies designed to target HER2 or HER3 in prostate cancer cells. A-D, LNCaP, $LNCaP^{enzaR}$, VCaP, $VCaP^{enzaR}$ and PC3 treated with vehicle, 20 µM tucatinib (A), 50 µg/mL T-DXd (B), 50 µg/mL DV (C), or 50 µg/mL P-DXd (D), 100 ng/mL rASPN, or 100 ng/mL rASPN in combination with drug. Inferred quantification of cell number by relative fluorescence intensity using CyQuant (n=3). **E,** Heatmap of LNCaP and $LNCaP^{enzaR}$ assessed by RNA-Seq (n=3). **F**, Volcano plots comparing LNCaP and $LNCaP^{enzaR}$ cells assessed by RNA-Seq (n=3). **G,** Heatmap of VCaP and $VCaP^{enzaR}$ assessed by RNA-Seq (n=3). **H,** Volcano plots comparing VCaP and $VCaP^{enzaR}$ cells assessed by RNA-Seq (n=3). Graphs are shown as mean ± SEM and analyzed by One-way ANOVA with Dunnett's post hoc analysis (A-D); *P≤0.05, **P≤0.01, ***P≤0.001, ****P≤0.0001.

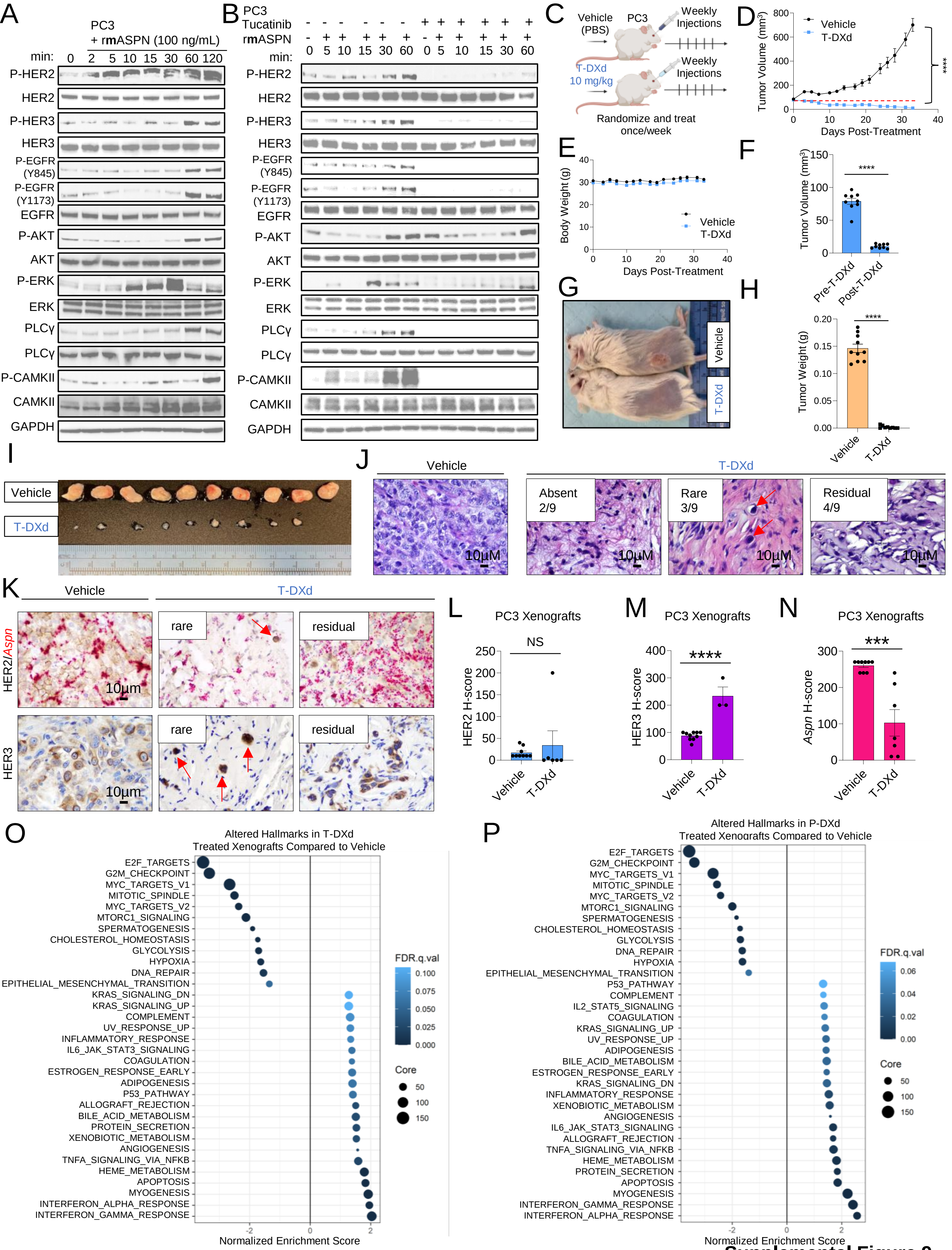

Supplemental Figure 9

Supplemental Figure 9 | Antibody drug conjugate designed to target HER2 (T-DXd) restricts growth of prostate cancer cells in vivo. A, PC3 cells (human) treated with 100 ng/mL recombinant mouse ASPN over a time course and assessed by immunoblotting for HER2 and HER3 pathway activation. **B,** PC3 cells (human) pre-treated with tucatinib (20µM) or vehicle for 1 hour followed by treatment with 100 ng/mL recombinant mouse ASPN over a time course and assessed by immunoblotting for HER2 and HER3 pathway activation (n=2). **C,** Schematic of PC3 subcutaneous xenografts grown to approximately 90mm^3 in NSG mice and then treated with vehicle (n=10) or 10 mg/kg T-DXd (n=9) by retro-orbital injection weekly for 5 cycles. **D,** Growth curves of PC3 xenografts in NSG mice treated with vehicle or T-DXd. **E,** Total weight of NSG mice with PC3 xenografts treated with vehicle or T-DXd. **F,** Tumor volume prior to T-DXd (pre-T-DXd) and after 5 cycles of weekly T-DXd (post-T-DXd). **G,** Photograph of representative of NSG mice with PC3 xenografts treated with vehicle or T-DXd at experimental endpoint. **H,** Tumor weight of isolated PC3 xenografts in NSG mice treated with vehicle or T-DXd at experimental endpoint. **I,** Photograph of isolated PC3 xenografts from NSG mice treated with vehicle or T-DXd at experimental endpoint. **J**, Representative H&E of PC3 xenografts treated with vehicle or T-DXd. Representative H&E images of absence of disease (left), rare cancer cells highlighted by red arrows (middle), and residual disease (right) are shown for T-DXd treated mice. **K,** Representative dual IHC/RNAscope for HER2 (IHC) and *Aspn* (RNAscope) and IHC for HER3 in PC3 xenografts treated with vehicle or T-DXd. Representative images of IHC/RNAscope of rare cancer cells highlighted by red arrows (left) and residual disease (right) are shown for T-DXd treated mice. **L, M,** Quantification of HER2 (L) and HER3 (M) expression by IHC as determined by H-Score (0, 1^+, 2^+, 3^+ intensity x percent positive) in PC3 xenografts treated with vehicle or T-DXd. **N,** Quantification of *Aspn* expression by RNAscope as determined by H-Score (0, 1^+, 2^+, 3^+ intensity x percent positive) in PC3 xenografts treated with vehicle or T-DXd. **O, P,** Bubble plots of Hallmarks by GSEA of T-DXd (O) and P-DXd (P) treated xenografts compared to vehicle. Graphs are shown as mean ± SEM and analyzed by multiple Student's t-test (D) or Student's t-test (F, H, L, M, N); ***P≤0.001, ****P≤0.0001.

Key Resources

REAGENT or RESOURCE	SOURCE	IDENTIFIER		NOTE
**Antibodies for Immunoblotting**				
P-HER3	Cell Signaling	Cat# 4791	RRID:AB 2099709	WB: 1:500 (BSA)
HER3	Cell Signaling	Cat# 12708	RRID:AB 2721919	WB: 1:1000 (BSA); IHC 1:50; IF: 1:100
P-HER2	Cell Signaling	Cat# 2243	RRID:AB 490899	WB: 1:500 (BSA)
HER2	Cell Signaling	Cat# 2165	RRID:AB 10692490	WB: 1:1000 (BSA)
P-EGFR (Y845)	Cell Signaling	Cat# 2231	RRID:AB 1264155	WB: 1:500 (BSA)
P-EGFR (Y1173)	Cell Signaling	Cat# 4407	RRID:AB 331795	WB: 1:500 (BSA)
EGFR	Cell Signaling	Cat# 4267	RRID:AB 2895042	WB: 1:1000 (BSA)
P-AKT	Cell Signaling	Cat# 4060	RRID:AB 2315049	WB: 1:5000 (BSA)
AKT	Cell Signaling	Cat# 9272	RRID:AB 329827	WB: 1:1000 (milk)
P-ERK	Cell Signaling	Cat# 4370	RRID:AB 2315112	WB: 1:1000 (BSA)
ERK	Cell Signaling	Cat# 9102	RRID:AB 330744	WB: 1:1000 (milk)
P-PLCγ	Cell Signaling	Cat# 2821	RRID:AB 330855	WB: 1:500 (BSA)
PLCγ	Cell Signaling	Cat# 2822	RRID:AB 2163702	WB: 1:1000 (milk)
P-CAMKII	Cell Signaling	Cat# 12716	RRID:AB 2713889	WB: 1:500 (BSA)
CAMKII	Cell Signaling	Cat# 3362	RRID:AB 2067938	WB: 1:500 (BSA)
GAPDH	Cell Signaling	Cat# 5174	RRID:AB 10622025	WB: 1:1000 (milk)
Flag-tag rabbit	Cell Signaling	Cat# 14793	RRID:AB 2572291	WB: 1:1000 (BSA)
Flag-tag mouse	Cell Signaling	Cat #:8146	RRID:AB 10950495	IF: 1:200
His-tag	Cell Signaling	Cat# 2365	RRID:AB 2115720	WB: 1:1000 (BSA)
ASPN	Sigma	Cat# HPA008435	RRID:AB 1845112	WB: 1:1000 (BSA)
Androgen Receptor	Cell Signaling	Cat# 5153	RRID:AB 10691711	WB: 1:2000 (milk)
anti-rabbit secondary, HRP	Cell Signaling	Cat# 7074	RRID:AB 2099233	WB: 1:1000-1:2000
**Recombinant Proteins**				
rhASPN	MyBioSource	MBS1292257		
rmASPN	Origene	TP505760		
rhNRG1	R&Dsystems	396-HB		
rmNRG1	R&Dsystems	9875-NR		
rhHER3-flag	Origene	TP309954		
rhHER2-flag	Origene	TP312583		
rhPDGFRβ-flag	Origene	TP306377		
rhEGF	R&Dsystems	236-EG		
rmEGF	R&Dsystems	2028-EG		
**Plasmids and constructs**				
HER3-FLAG	Origene	RC212583		
HER3-no flag	SinoBiological	HG10201-UT		
HER2-FLAG	Origene	RC212583		
HER2-no flag	AddGene	16257		
pIRES-Neo3	Clontech	631621		
ASPN-D14 no tag	Modified from Clontech pIRES-Neo3			PMID: 26446945
ASPN-D14-3xFLAG	Modified from Clontech pIRES-Neo3			PMID: 26446945
pRP[Exp]-CMV>{hERBB3[NM 001982.4]*(ΔDomain I)}/3xFLAG	VectorBuilder	Vector ID: VB240411-1594yjb		
pRP[Exp]-CMV>{hERBB3[NM 001982.4]*(ΔDomain III)}/3xFLAG	VectorBuilder	Vector ID: VB240411-1595cyh		
pRP[Exp]-CMV>{hERBB3[NM 001982.4]*(ΔDomain I&III)}/3xFLAG	VectorBuilder	Vector ID: VB240729-1665wbu		
pBABE-puro-hTERT	Addgene	1771		
**CRISPR guides**				
HER2 sgRNA	IDT			TCATCGCTCACAACCAAGTG
HER3 sgRNA	IDT			ATCATGTGAGACAACACCGG
Alt-R™ S.p. HiFi Cas9 Nuclease V3	IDT	1081060		
Lipofectamine™ CRISPRMAX™ Cas9 Transfection Reagent	ThermoFisher	CMAX00008		
**Sequencing Primers**				
HER2 Forward	IDT			GCAAGGTAGGGCGTGATCTT
HER2 Reverse	IDT			GCCCAGAAGGGACACCATTT
HER3 Forward	IDT			TGGACCATGCCCACATTGTAA
HER3 Reverse	IDT			GCCCCCAGACAAGCAGTTC
**DNA purification and PCR amplification**				
Wizard SV96 Gnomic DNA Purification	Promega	A2370		
Phusion Mastermix	Thermofisher	F531L		
QIAquick 96 PCR purification kit	Qiagen	28181		
**Inhibitors for drug assays**				
Tucatinib	SelleckChem	S8362		
Pertuzumab	SelleckChem	A2008		
Trastuzumab	SelleckChem	A2007		
Trastuzumab deruxtecan	SelleckChem	E0200		
Patritumab deruxtecan	SelleckChem	D4004		
Patritumab	SelleckChem	A2506		
Disitamab	MedChemExpress	HY-P99854		
Disitamab Vedotin	SelleckChem	D4049		
Enzalutamide	SelleckChem	S1250		
**Cell Viability**				
CyQuant NF Cell Proliferation Assay	ThermoFisher	C35006		
GloMax Explorer	Promega			
Live Cell Imaging Solution	Invitrogen	A59688DJ		
**Proximity Ligation Assay**				
DuoLink® *In Situ* Detection Reagents Orange	MilliporeSigma	DUO92007		
DuoLink® *In Situ* PLA® Probe Anti-Mouse MINUS	MilliporeSigma	DUO92004		
DuoLink® *In Situ* PLA® Probe Anti-Rabbit PLUS	MilliporeSigma	DUO92002		
DuoLink® *In Situ* Mounting Media with DAPI	MilliporeSigma	DUO82040		
Flag-tag mouse	Cell Signaling	Cat #:8146	RRID:AB 10950495	IF: 1:200
HER3	Cell Signaling	Cat# 12708	RRID:AB 2721919	IF: 1:100
**Co-Immunoprecipitation**				
Lipofectamine 3000	ThermoFisher	L3000015		
IgG agarose beads	Sigma	A0919		
Sigma M2 Flag beads	Sigma	M8823		
3x Flag peptide	Sigma	F4799		
Protein A agarose beads	Cell Signaling		9863	
**Next Generation Sequencing**				
NovaSeq 6000	Illumina			
RNeasy Protect Cell Mini Kit	Qiagen	74624		
Reagen Dx	Qiagen	19088		
Stainless Steel Beads	Qiagen	69989		
**IHC**				
Anti-ASPN antibody	Sigma	Cat# HPA008435	RRID:AB 1845112	IHC: 1:400
Anti-HER3 antibody	Cell Signaling	Cat# 12708	RRID:AB 2721919	IHC: 1:50
Antibody diluent	ThermoFisher	3218		
BLOXALL® Endogenous Blocking Solution, Peroxidase and Alkaline Phosphatase	Vector Laboratories	SP-6000-100		
IHC Antigen Retrieval Solution - High pH	Invitrogen	00-4956-58		
ImmPACT® DAB (3,3'-Diaminobenzidine) Substrate Kit, Peroxidase (HRP)	Vector Laboratories	SK-4105		
ImmPRESS® HRP Horse Anti-Rabbit IgG Polymer Detection Kit, Peroxidase	Vector Laboratories	MP-7401		
Mayer's Hematoxylin Solution	Sigma	MHS32		
Protein Block Serum-Free	Dako	X0909		
Target Retrieval Solution Ready to Use	Dako	S1700		
H&E Staining Kit	Abcam	ab245880		
**RNA-Protein Integrated Co-detection (RNAScope® with IHC)**				
Anti-HER2 antibody	Cell Signaling	Cat# 2165	RRID:AB 10692490	IHC: 1:50
RNA-Protein Co-detection Ancillary Kit	ACD	323180		
RNAscope® Probe - Hs-ASPN-C2 - Homo sapiens asporin (ASPN), transcript variant 2, mRNA	ACD	404481-C2		
RNAscope® Probe - Mm-Aspn-C2 - Mus musculus asporin (Aspn) transcript variant 1 mRNA	ACD	502051-C2		
RNAScope Probe NRG1 - C1	ACD	311181-C1		
RNAscope® Probe Diluent	ACD	300041		
RNAscope® 2.5 HD Duplex Reagent Kit	ACD	322430		
**Cell Migration Assay**				
QCM™ 24-well Migration Assay	Millipore	ECM508		
**PCAF Generation**				
Tumor Dissociation Kit	Miltenyi Biotec	130-095-929		
Red Blood Cell Lysis Solution	Miltenyi Biotec	130-094-183		
RoosterNourish-MSC media	RoosterBio	K82003		
In Vivo Studies				
Matrigel	Corning	354234		
**Software and Algorithms**				
Cutadapt v4.5	https://cutadapt.readthedocs.io/en/v4.5/			
DESeq2 v1.30.1	https://bioconductor.org/packages/release/bioc/html/DESeq2.html			
FastQC v0.11.9	https://www.bioinformatics.babraham.ac.uk/projects/fastqc			
featureCounts v2.0.2	https://subread.sourceforge.net/featureCounts.html			
Genome Gencode GRCh38.p13	https://www.gencodegenes.org/human/release 38.html			
Genome Gencode GRCm38.p6	https://www.gencodegenes.org/mouse/release M24.html			
GraphPad Prism 10	https://www.graphpad.com/			
GSEA v4.3.2	https://www.gsea-msigdb.org/gsea/index.jsp			
GSEA DB v2022.1.Hs	https://software.broadinstitute.org/cancer/software/gsea/wiki/index.php/MSigDB v2022.1.Hs Release Notes			
GTF Gene Annotation Gencode v38	https://www.gencodegenes.org/human/release 38.html			
GTF Gene Annotation Gencode vM24	https://www.gencodegenes.org/mouse/release M24.html			
Heatmap3 v1.1.9	https://cran.r-project.org/web/packages/heatmap3/index.html			
ImageJ 1.54g	https://imagej.net/			
ImageJ Plugin: Wound Healing Size Tool Updated (2021)	https://github.com/AlejandraArnedo/Wound-healing-size-tool			
PEPPI	https://zhanggroup.org/PEPPI/			
QuPath v0.3.0	https://qupath.github.io			
SnapGene	https://www.snapgene.com/			
STAR v2.7.11a	https://github.com/alexdobin/STAR			
WebGestaltR v0.4.6	https://cran.r-project.org/web/packages/WebGestaltR/index.html			
**Sequencing Data**				
RNA-sequencing: ASPN-treated LNCaP	GEO	GSE271579 (Token: wnslisqexfcllch)		
RNA-sequencing: NRG1-treated LNCaP	GEO	GSE271580 (Token: ciatiqkctxshzqv)		
RNA-sequencing: ASPN-treated MyC-CaP	GEO	GSE271738 (Token: evyrqmwklnqtdwd)		
RNA-sequencing: Enza-resistant LNCaP	GEO	GSE271739 (Token: wnubokawxdonbed)		
RNA-sequencing: NRG1-treated MyC-CaP	GEO	GSE271740 (Token: mpmjyuyuxtufbin)		
RNA-sequencing: Enza-resistant VCaP	GEO	GSE271742 (Token: wtqhomqcdbkdhwn)		
RNA-sequencing: T-DXd and P-DXd treated tumors	GEO	GSE284054 (Token: wzixqwwmdzcppkp)		

SUPPLEMENTAL METHODS

Cell lines

LNCaP, VCaP, MyC-CaP, PC-3, DU145, HEK293, and MCF7 were obtained from ATCC. LNCaP and VCaP enzalutamide resistant cells (LNCaP^{enzaR} and VCaP^{enzaR}) were generated from parental lines by continuous treatment with 10 μM enzalutamide and then maintained on 5 μM enzalutamide. Cell lines were maintained in either DMEM (DU145, VCaP, MyC-CaP, HEK293) or RPMI 1640 (PC3, LNCaP, 22RV1) supplemented with 10% fetal bovine serum (Gibco) and 1% penicillin/streptomycin (Gibco). Prostate CAF (PCAF) were isolated from prostate tissue obtained at radical prostatectomy. A rapid frozen H&E of isolated tissue was used for GU pathologist verification of prostate cancer with cribriform morphology. Prostate cancer tissue was dissociated using the Tumor Dissociation Kit (Miltenyi Biotec) as per manufacturer's protocol (m_imp_tumor_01 program before the 37C_h_TDK2 program). After removal of erythrocytes with the Red Blood Cell Lysis Solution (Miltenyi Biotec), cells were cultured in RoosterNourish-MSC media (RoosterBio) supplemented with 1% penicillin/streptomycin (Gibco) to enrich for PCAF. The retroviral pBABE-puro-hTERT vector system (Addgene) was used for PCAF immortalization. STR profiling and *Mycoplasma* testing was performed to authenticate cell lines (Johns Hopkins University Genetic Resources Core Facility).

Recombinant protein

Mouse and human recombinant NRG1β were purchased from R&D Systems. Mouse recombinant ASPN was purchased from Origene. Human recombinant ASPN was synthesized by MyBioSource to ensure it was the D14 variant.

RNA sequencing

Cells were plated in triplicate in 6 well plates (500,000-750,000 cells/well) and allowed to adhere for 24-48 hrs. Cells were starved in serum-free and calcium-free media (Vanderbilt Cell Culture Core) for 6-12 hours prior to treatment with buffer, 100 ng/mL rASPN (MyBioSource, Origene) or 15 ng/mL rNRG1 (R&D Systems). Total RNA was extracted from treated cells with the RNeasy Protect Cell Mini Kit (Qiagen) as per manufacturer's protocol. Illumina TruSeq mRNA sequencing libraries were prepared and sequenced at multiplex paired-end 150 bp on the Illumina NovaSeq 6000 (Illumina). Reads were trimmed to remove adapter sequences using Cutadapt v4.5

and aligned to corresponding genomes (Gencode GRCh38.p13 for human and Gencode GRCm38.p6 for mouse) using STAR v2.7.11a. GTF gene annotation (Gencode v38 for human and Gencode vM24 for mouse) was provided to STAR to improve the accuracy of mapping. Quality control on both raw reads and adaptor-trimmed reads was performed using FastQC v0.11.9 (www.bioinformatics.babraham.ac.uk/projects/fastqc). One replicate of LNCaP cells at time zero was not included in the final analyses as it did not meet the quality control threshold. featureCounts v2.0.2 was used to count the number of mapped reads to each gene. Heatmap3 v1.1.9 was used for cluster analysis and visualization. Significantly differential expressed genes with absolute fold change ≥ 1.5 and FDR adjusted p value ≤ 0.05 were detected by DESeq2 v1.30.1. Gene set enrichment analysis was performed using GSEA package v4.3.2 on database v2022.1.Hs. Over-representation analysis was performed on differentially expressed genes using the WebGestaltR package v0.4.6. The bulk RNA-sequencing data generated in this study is deposited at the Gene Expression Omnibus (GEO) database.

Immunoblotting

Cells were plated in 6 well plates (500,000-750,000 cells/well) and allowed to adhere for 24-48 hours. Cells were starved in serum-free and calcium-free media (Vanderbilt Cell Culture Core) for 18 hours overnight prior to treatment with 100 ng/mL rASPN (MyBioSource, Origene), 15 ng/mL rNRG1 (R&D Systems) or 10 ng/mL rEGF (R&D Systems). Cell lysates were collected in RIPA buffer (Pierce) supplemented with EDTA-free protease/phosphatase inhibitor tablets (Pierce), sonicated, and spun at max speed for 5 min at 4°C. Protein concentration was normalized using the BCA kit (Pierce). Sample buffer (ThermoFisher, 4x NuPage) and DTT (1M) were added, and samples boiled for 10 min at 70°C. Samples were loaded onto 4-12% NuPage gels (Invitrogen) and ran for 40 min at 200 V in 1x MOPS buffer. Proteins were transferred on the Bio-Rad Trans Blot Turbo high molecular weight setting to 0.2 μM PVDF membranes (Bio-Rad Trans-Blot Turbo Packs), blocked for 1 hr in 5% bovine serum albumin (BSA) or milk, and incubated with indicated primary antibody (catalog numbers and concentrations can be found in supplemental reagents table) overnight at 4°C. Next day, membranes were washed 3 times for 5 min each with TBST, incubated with secondary for 1 hr at room temperature (RT), washed 3 times for 5 min each with TBST, developed using enhanced chemiluminescence (ThermoFisher, femto), and imaged on the ChemiDoc (BioRad). For inhibitor experiments, cells were pre-treated with inhibitor for 1 hour

prior to treatment with recombinant protein. Western blot quantification and analysis was performed using ImageJ.

Computational binding prediction

Computational structural models of the interaction between the HER2/HER3 heterodimer and ASPN were generated using a two-stage modeling approach with AlphaFold2 and Rosetta. First, AlphaFold-Multimer (v2.3) was used to generate candidate complexes of ASPN with HER2/HER3. Each complex was refined using Rosetta's Relax protocol (alternating rotamer repacking and torsion-space minimization) utilizing the REF2015 score function, and coordinate restraints were applied to backbone heavy atoms during refinement. Next, Rosetta refinement docking was performed on AlphaFold2 candidate complexes in two stages: (1) low-resolution centroid-based docking utilizing the interchain centroid scoring weights followed by (2) high-resolution all-atom docking utilizing the REF2015 score function patched with docking weights. For this analysis, default initial random docking perturbations (3Å and 8°) were used. The Monte Carlo Metropolis (MCM) stages of the docking protocol were performed with step sizes of 0.25Å and 5°. The final docked pose interface was further refined with additional relax. The final binding energy calculation was performed by calculating the total energy of the final docked pose and subtracting from it the total energy of the system when ASPN was separated from the complex. Prior to calculating the energy of the separated system, the interface residues were repacked. The final model was chosen based on its docking funnel convergence and overall binding energy. Figures and plots were generated with PyMOL (v2.5), Matplotlib, and/or Seaborn.

Proximity ligation assay

Protein interaction was assessed using the Duolink® proximity ligation fluorescent assay per manufacturer's protocol (Millipore Sigma, DUO92102). In brief, HEK293 cells were plated at 50,000 cells per chamber in 4 well glass chamber slides with removeable wells (ThermoFisher) and allowed to adhere overnight. Next day, cells were transfected with equal amounts of HER3-WT and ASPN-3xFLAG tag plasmid in serum free media using Lipofectamine 3000 (ThermoFisher) per manufacturer's protocol. After 48 hours, cells were fixed in 4% PFA (Affymatrix) in PBS for 20 min at RT, washed twice with PBS, and permeabilized with ice cold methanol for 20 min at -20°C. Cells were washed twice with PBS, blocked in Duolink block solution for 1 hr at 37°C, and stained

overnight at 4°C with anti-HER3 rabbit (Cell Signaling, 1:100) and anti-FLAG mouse (Cell Signaling, 1:200) diluted in antibody dilution buffer. Next day, cells were washed twice with 5% BSA in PBS then incubated for 1 hr at 37°C with PLUS/MINUS probes diluted 1:5 in antibody dilution buffer. Cells were washed twice with 1x Wash Buffer A (10 mM Tris, pH-7.4, 150 mM NaCl, and 0.05% Tween) and incubated with ligation mix (1x ligation buffer with ligase diluted 1:40) for 30 min at 37°C. Cells were washed twice more with 1x Wash Buffer A then incubated with amplification mix (1x amplification buffer with polymerase diluted 1:80) for 100 min at 37°C. Cells were washed twice with 1x Wash Buffer B (200 mM Tris, pH-7.5, 100 mM NaCl) then quickly with 0.01x Wash Buffer B. Chambers were removed and slides cover slipped using mounting media with DAPI (Millipore Sigma). Image acquisition was performed by taking a 10 µm Z-stack on a Nikon Spinning Disc Fluorescent Microscope using a 60x objective. Total PLA signal and cell count were analyzed using ImageJ.

Co-immunoprecipitation

For recombinant protein approach, 5.46 nM recombinant proteins were added to 1x binding buffer (100 mM sodium phosphate, pH 8, 600 mM NaCl, 0.2% Tween) and incubated for 1 hr at 4°C. Samples were incubated with anti-FLAGM2 magnetic beads (Sigma) for 1 hr at 4°C, washed twice each with 1 mL binding buffer and 1 mL 1x TBS buffer, respectively, and eluted with 150 ng/µL FLAG peptide (Sigma) by rotating for 30 min at 4°C. Samples were prepared for western blot as described above and ran on 4-12% NuPage BOLT gels (Invitrogen). For in vitro Co-IP, using HER3-FLAG plasmids, cells were plated ($0.7x10^6$ cells/T25 or $3.0x10^6$ cells/T75) and allowed to adhere overnight. Plasmids of interest were transfected in serum free media using Lipofectamine 3000 (ThermoFisher) per manufacturer's protocol. After 48 hours, cells were crosslinked (PBS+ 1% formaldehyde) and harvested with ice cold PBS. Samples were spun at 300xg for 5 min, PBS removed, and pellet resuspended in 500 µL ice cold lysis buffer (50 mM Tris-HCl, pH 7.5, 1.5 mM MgCl2, 150 mM NaCl, 1 mM EGTA, 2 mM EDTA, 10% glycerol, 0.5% NP-40, 0.25% Triton X-100) supplemented with protease and phosphatase inhibitors (Sigma, 100x). Cells were rotated at 4°C for 30 min, sonicated, and spun at 14,000g for 5 min at 4°C. Samples were normalized using the BCA assay (Pierce), pre-cleared for 1 hr at 4°C with mouse IgG-agarose beads (Sigma) and incubated overnight at 4°C with anti-FLAG M2 magnetic beads (Sigma). The next day, beads were washed twice each with 1 mL lysis buffer and 1 mL 1x TBS buffer, respectively. Samples were eluted with 150 ng/µL FLAG peptide (Sigma) by rotating for 30 min at 4°C and prepared for western blot,

as described above. For in vitro Co-IP using truncated HER3-3xFLAG plasmids, samples were eluted with 1000 ng/μL for 1-18 hr. IP solution was removed and beads were boiled with 50 μL1x Master Mix (Thermofisher 4x NuPage buffer and 1M DTT) for 10 min at 72°C before adding back to IP sample. All other samples were prepared for western blot as described previously. For HER3 endogenous Co-IP, $2.5x10^6$ cells/T75 were plated, allowed to adhere and serum/calcium starved overnight. Next day, cells were treated with either 100-200 ng/mL rhASPN (MyBioSource) or CAF conditioned media. Cells were then crosslinked (PBS+1% formaldehyde), washed with ice cold PBS and harvested in 1x lysis buffer buffer (50 mM Tris-HCl, pH 7.5, 1.5 mM MgCl2, 150 mM NaCl, 1 mM EGTA, 2 mM EDTA, 10% glycerol, 0.5% NP-40, 0.25% Triton X-100) supplemented with protease and phosphatase inhibitors (Pierce). Samples rotated for 1 hr at 4°C to finish lysing, then spun at 14,000g for 10 min. Supernatants were transferred to a new tube, normalized, and pre-cleared with protein A agarose beads (Cells Signaling) for 1 hr at 4°C. 0.9 mg of protein was incubated with anti-HER3 primary antibody overnight (Cell Signaling, 1:50). Next day, samples were incubated for 2 hr at 4°C with protein A agarose beads (Cell Signaling) and beads were washed 3 times with 1x lysis buffer. Beads were boiled with 50 μL1x Master Mix (Thermofisher 4x NuPage buffer and 1M DTT) for 10 min at 72°C to elute and analyzed by western blot, as described above.

Gene targeting with CRISPR-Cas9

Gene targeting of *ERBB2* and *ERBB3* was performed using CRISPR-Cas9. In brief, the targeted CRISPR guides were designed and validated using NCBI Primer Blast. HER2 guides targeted exon 3 of the extracellular domain and HER3 guides targeted exon 20 of the intracellular kinase domain. Delivery of CRISPR reagents was accomplished using the IDT Alt-R CRISPR-Cas9 System: Cationic lipid delivery of CRISPR ribonucleoprotein complexes into mammalian cells protocol (https://www.idtdna.com/pages/support/guides-and-protocols) and the Lipofectamine CRISPRMAX kit (ThermoFisher) per manufacturer's protocol. The Ribonucleoprotein (RNP) Complex was assembled by incubating 1 μM CRISPR-Cas9 sgRNA (IDT), 1 μM HiFi Cas9 Nuclease V3 protein (IDT) and 1x Cas9 PLUS reagent (Invitrogen) in Opti-MEM for 5 min at room temperature. Transfection complexes were formed by combining the assembled RNP complex with the CRISPRMAX transfection reagent (ThermoFisher) and incubating for an additional 20 min. Cells of interest were harvested, resuspended in complete media without antibiotics, and plated in triplicate in 96 well plates with 40,000 cells per well and 10 nM

RNP complex. Following a 48 hr incubation, cells were pooled, and single cell diluted in complete media with antibiotics. Single cell clones were identified, and colonies expanded for 3-4 weeks. Clones were validated by Sanger Sequencing and western blot. For sequencing, gDNA was isolated with the Wizard SV96 Genomic Purification system (Promega). Regions of interest were PCR amplified using Phusion Mastermix (ThermoFisher) and PCR product purified using the QIAquick 96 PCR purification kit (Qiagen). Samples were submitted to Genewiz for Sanger Sequencing and trace files assessed for mutations using SnapGene. Genotypes (TWT, heterozygous knockout, and homozygous knockout) were validated by western blot.

Cell migration assays

Transwell assay was performed on LNCaP as per manufacturer's protocol of the QCM™ 24-well Migration Assay (Millipore). Briefly, 50-70% confluent cells were serum-starved for 24 hrs, then 30,000 cells were seeded per upper chamber (8 μm pores) in respective conditions in serum-free media, with 20% FBS media as attractant in the lower chamber. After 24 hrs, migrated cells were harvested by staining and imaged, and the colorimetric agent was extracted for detection on a microplate reader (570 nm on accuSkan FC, Fisher Scientific). Scratch assays were performed on PC3 and DU145. 50,000 cells were seeded per 24-well well and cultured to full confluency. Confluent cells were starved overnight for at least 12 hrs (serum-free calcium-free media for PC3 and serum-free media for DU145), pre-treated with tucatinib in starvation media for 1 hr, and scratched with a 200 μL pipette tip in a t-cross within each well. Cells were then treated with respective conditions in 10% FBS media. Images of the same scratch regions (ensured by manually added fiducial on bottom of wells) were taken at initiation and termination timepoints (0-20 hrs for PC3 and 0-24 hrs for DU145). Percent closure of scratches was analyzed with the ImageJ plugin, Wound Healing Size Tool Updated. 100 ng/mL of rASPN and 20 μM of tucatinib were used, with DMSO as vehicle control.

Immunohistochemistry

ASPN IHC was done as previously described (23, 25, 28). Briefly, formalin-fixed paraffin-embedded (FFPE) tissue sections were deparaffinized, steamed in antigen retrieval buffer (Dako) for 40 min, blocked with protein block serum-free solution (Dako), and incubated with anti-ASPN primary antibody (Sigma, 1:400) overnight at 4°C. Slides were then incubated with secondary antibodies (Vector Laboratories), developed with 3,3'-

Diaminobenzidine kit (Vector Laboratories), and counterstained with Mayer's hematoxylin (Sigma). HER3 IHC was performed similarly but steamed in high pH antigen retrieval buffer (Invitrogen), treated with BLOXALL® (Vector Laboratories) for 10 min before protein blocking, and incubated with anti-HER3 primary antibody (Cell Signaling Technology, 1:50). IHC slides were analyzed by a pathologist and quantified for expression intensity (0, 1^+, 2^+, 3^+) and percent of staining to calculate an H-Score (expression intensity x percent positive cells).

RNA-protein integrated co-detection (RNAScope® with IHC)

Co-detection of RNA (ASPN) and protein (HER2) was performed as per manufacturer's (Advanced Cell Diagnostics) Technical Note, Integrated Co-Detection Workflow (ICW, version MK 51-149/Rev B), with optimized conditions. Briefly, human surgical and biopsy metastatic prostate cancer and prostate cancer xenograft FFPE tissue sections (4 µm) were deparaffinized and treated with hydrogen peroxide, steamed in freshly made target retrieval buffer for 25 min, then incubated with anti-HER2 antibody (Cell Signaling Technology, 1:50) overnight at 4°C. Post-primary fixation was performed for 60 min and protease treatment was performed for 25 min. Slides were then hybridized with *ASPN* RNAScope®, incubated with secondary antibodies (Vector Laboratories), developed with 3,3'-Diaminobenzidine kit (Vector Laboratories), and counterstained with Mayer's hematoxylin (Sigma). Slides were analyzed by pathologists and quantified for expression intensity (0, 1^+, 2^+, 3^+) and percent of staining to calculate an H-Score (expression intensity x percent positive cells). HER2-low was defined as HER2 IHC of 1^+ or 2^+ in ≥ to 10% of the cancer cells. HER2-ultra low was defined as HER2 IHC of 1^+ or 2^+ in < 10% of the cancer cells.

RNA in situ hybridization

Surgical and biopsy metastatic prostate cancer FFPE tissue sections (4 µm) were analyzed for *NRG1* expression using RNAscope® 2/5 HD Duplex Assay according to manufacturer's (Advanced Cell Diagnostics) instructions and as previously described (24, 25). Slides were analyzed by a pathologist and quantified for expression intensity (0, 1^+, 2^+, 3^+) and percent of staining to calculate an H-Score (expression intensity x percent positive cells).

Inhibitor assays

Drug titrations were performed by plating 1,000-5,000 cells/well in triplicate in 96 well plates. Cells were allowed to adhere for 1-3 days before treatment with vehicle (DMSO or PBS) or inhibitor (tucatinib, trastuzumab, pertuzumab, disitimab, patritumab, T-DXd, DV, P-DXd). Fresh drugs were added every other day and cell proliferation assessed 5-10 days post-treatment using the CyQuant NF Cell Proliferation Assay (ThermoFisher). Cells were incubated for 30 min with 1x NF dye in Live Cell Imaging Solution (ThermoFisher) and fluorescence intensity measured using the GloMax Plate Reader (Promega). For ASPN treated cells, cells were treated every day for 5-10 days.
