# Supplementary material for: Identification of asporin as a HER3 ligand exposes a therapeutic vulnerability in prostate cancer
Source: JCI Insight. 2025 Aug 22;10(16):e187151. doi: 10.1172/jci.insight.187151 (PMC12406728; doi:10.1172/jci.insight.187151)

Table	Title
1H - LNCaP	LNCaP + ASPN, HER2/HER3 western blots
1H - VCaP	VCaP + ASPN, HER2/HER3 western blots
1H - MyC-CaP	MyC-CaP + ASPN, HER2/HER3 western blots
1H - LNCaP EnzaR	LNCaP EnzaR + ASPN, HER2/HER3 western blots
1H - VCaP EnzaR	VCaP EnzaR + ASPN, HER2/HER3 western blots
1H - PC3	PC3 + ASPN, HER2/HER3 western blots
2A	LNCaP+ASPN western blots
2C	MyC-CaP+ASPN western blots
3A	LNCaP+NRG1 western blots
3C	MyC-CaP+NRG1 western blots
4C	Recombinant HER3 Co-IP
4F	HEK293 HER3-FLAG Co-IP
4G	LNCaP Parental HER3-FLAG Co-IP
4H	LNCaP HER2 KO HER3-FLAG Co-IP
4I	HEK293 HER3ΔI-3xFLAG Co-IP
4J	HEK293 HER3ΔIII-3xFLAG Co-IP
4K	HEK293 HER3ΔI/III-3xFLAG Co-IP
5A	LNCaP HER3 TKD ASPN western blots
5C	LNCaP HER2 KO ASPN western blots
6A	LNCaP Tucatinib ASPN western blots
6C	PC3 Tucatinib ASPN western blots
9B	ErbB protein expression LNCaP Parental/EnzaR, VCaP Parental/EnzaR
S2A	VCaP+ASPN western blots
S2B	DU145+ASPN western blots
S2C	PC3+ASPN western blots
S2E	LNCaP+ASPN with EGF control western blots
S2F	VCaP+ASPN with EGF control western blots
S2G	LNCaP EnzaR+ASPN with EGF control western blots
S2H	VCaP EnzaR+ASPN with EGF control western blots
S2I	DU145+ASPN with EGF control western blots
S2J	MyC-CaP+ASPN with EGF control western blots
S2K	ERBB protein expression all cell lines
S2L	ASPN expression in PCAF
S2M	PC3 cells treated with PCAF conditioned media western blots
S4B	Recombinant HER2 Co-IP
S4C	Recombinant PDGFRB Co-IP
S4D	PC3 endogenous HER3 co-IP treated with recombinant ASPN western blots
S4E	PC3 endogenous HER3 co-IP treated with PCAF CM western blots
S4G	LNCaP HER2 KO validation western blots
S4I	LNCaP HER3 TKD validation western blots
S5A	LNCaP HER3 TKD + rASPN western blots - additional clones
S5B	LNCaP TKD transfected with WT HER3 or HER3ΔI/III + rASPN western blots
S5C	LNCaP HER2 KO + rASPN western blots - additional clones
S9A	PC3 + recombinant mouse ASPN western blots
S9B	PC3 + tucatinib + recombinant mouse ASPN western blots

Full Unedited Blots for Figure 1H LNCaP

Lanes: Hi Calcium, 0, 2, 5, 10, 15, 30, 60, 120

P-HER2

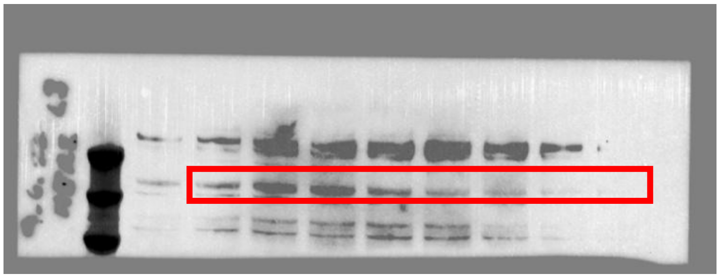

HER2

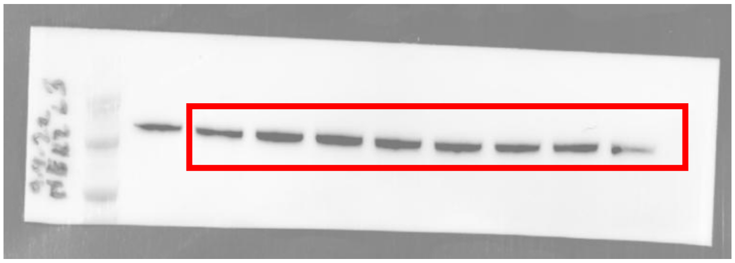

P-HER3

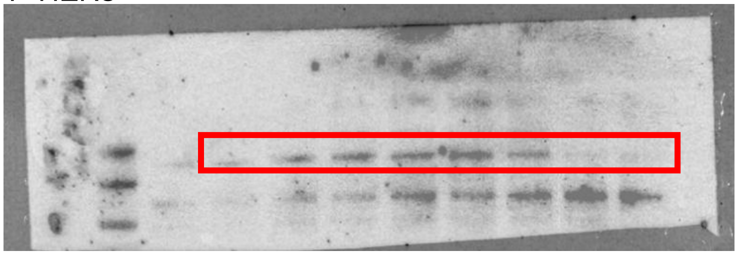

HER3

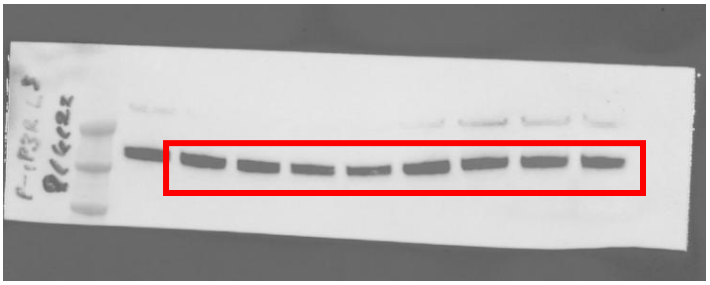

Full Unedited Blots for Figure 1H LNCaP Continued

GAPDH

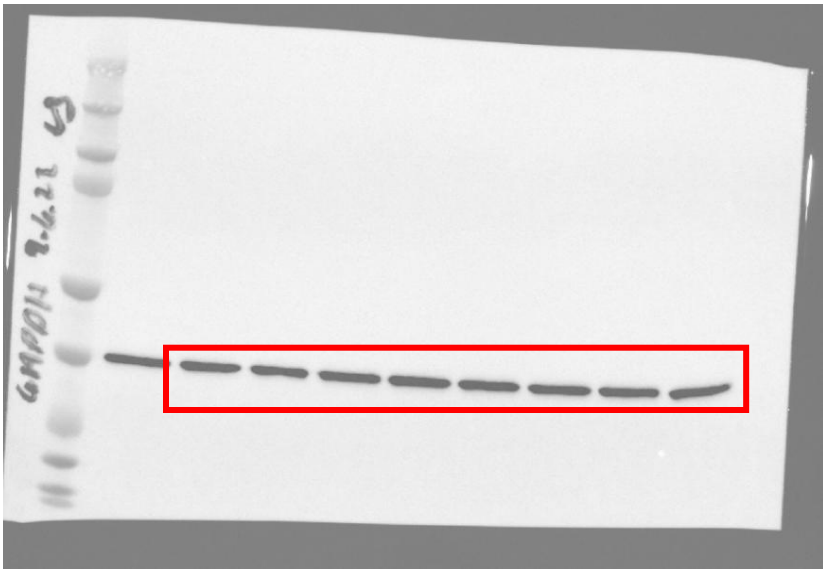

Full Unedited Blots for Figure 1H VCaP

Lanes:0, 2, 5, 10, 15, 30, 60, 120

P-HER2

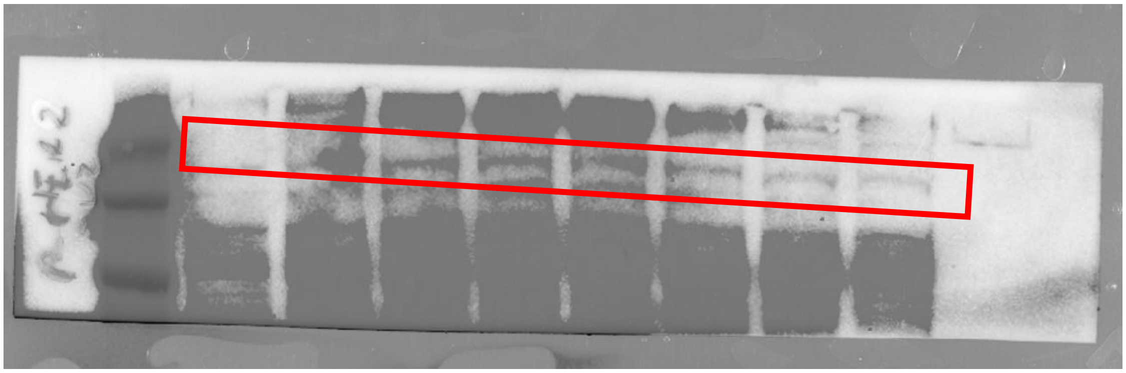

HER2

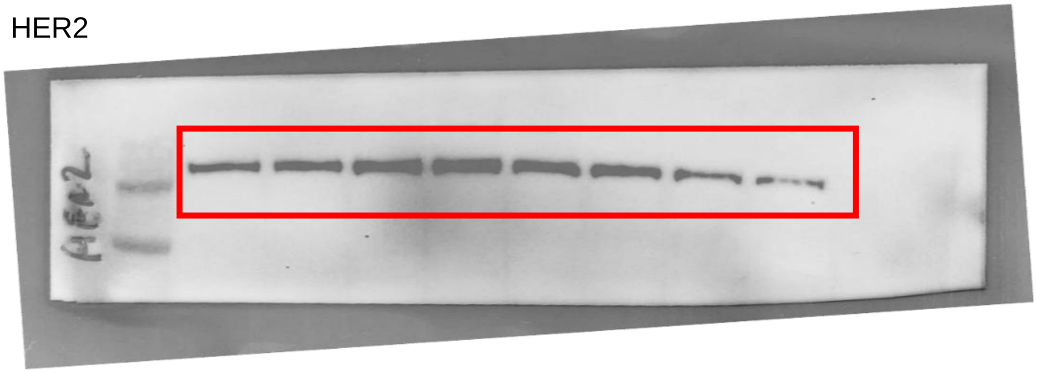

P-HER3

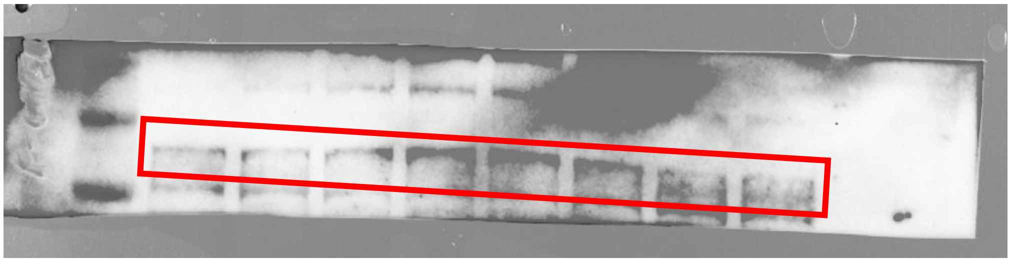

HER3

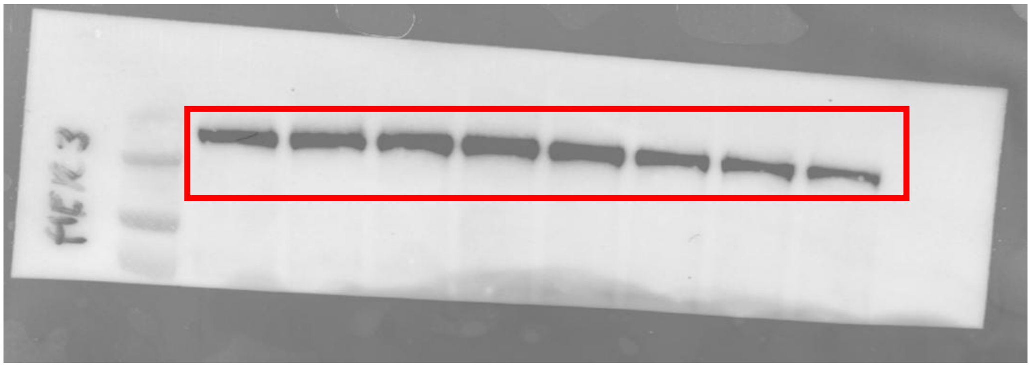

Full Unedited Blots for Figure 1H VCaP Continued

GAPDH

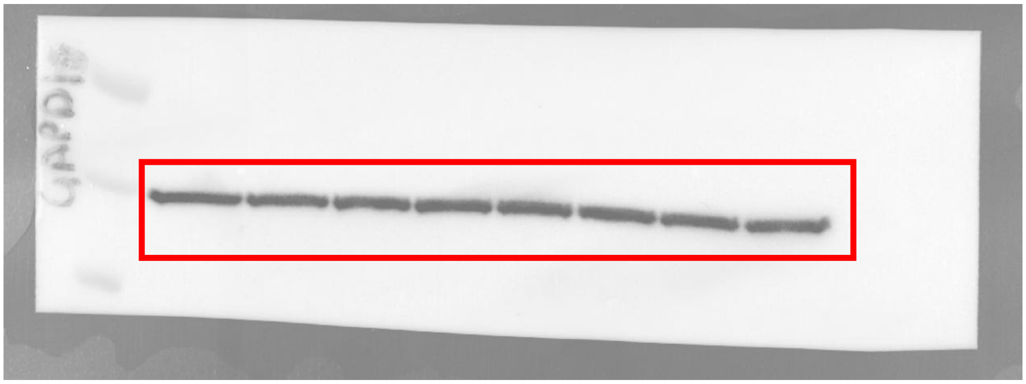

Full Unedited Blots for Figure 1H MyC-CaP

Lanes: Hi Calcium, 0, 2, 5, 10, 15, 30, 60, 120

P-HER2

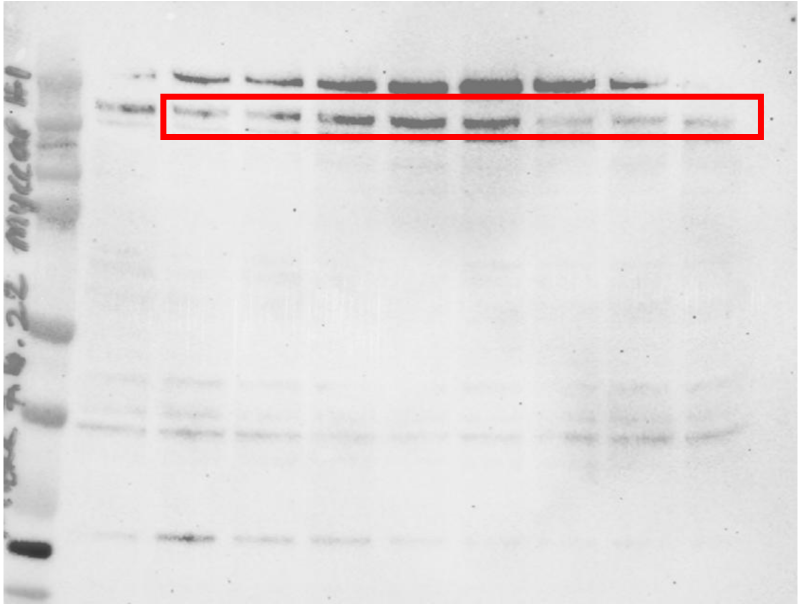

HER2

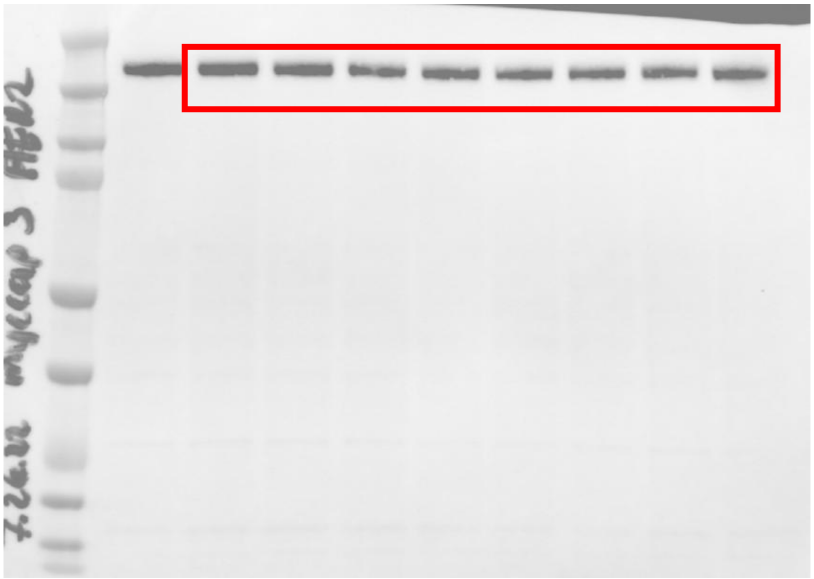

Full Unedited Blots for Figure 1H MyC-CaP Continued

P-HER3

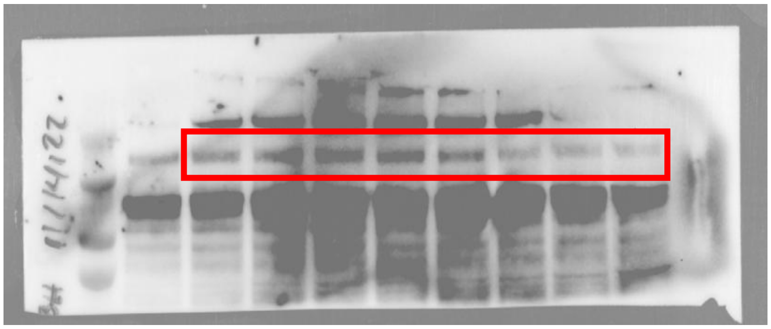

HER3

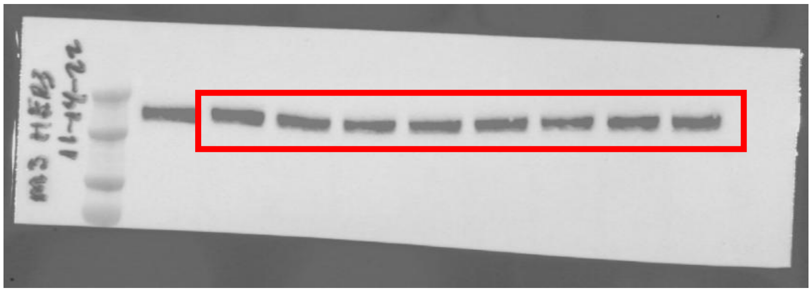

GAPDH

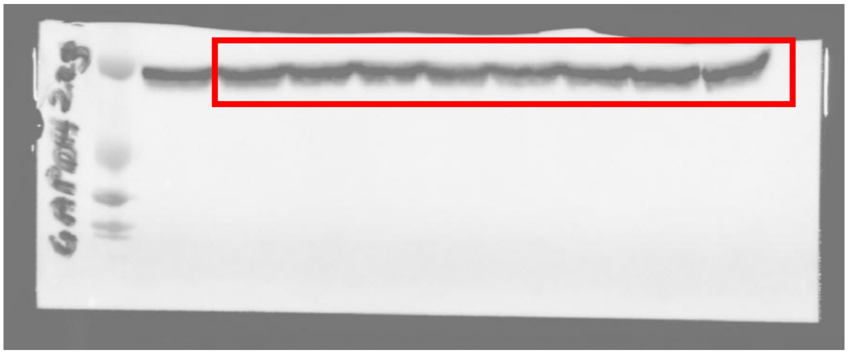

Full Unedited Blots for Figure 1H LNCaP EnzaR

Lanes:0, 2, 5, 10, 15, 30, 60, 120

P-HER2

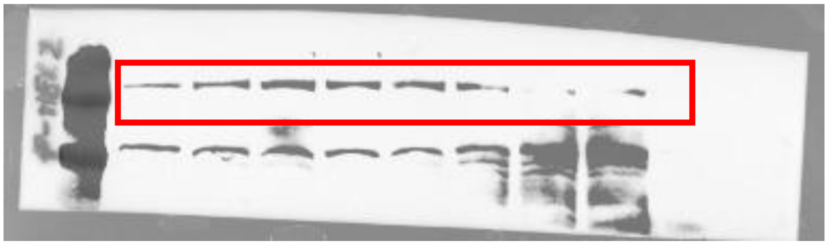

HER2

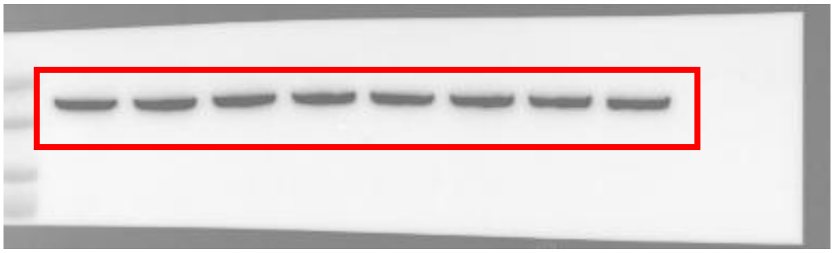

P-HER3

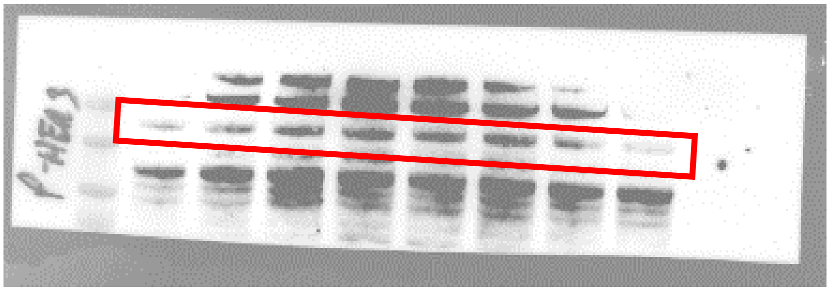

HER3

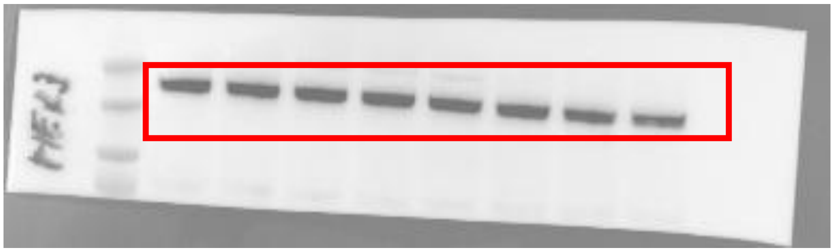

GAPDH

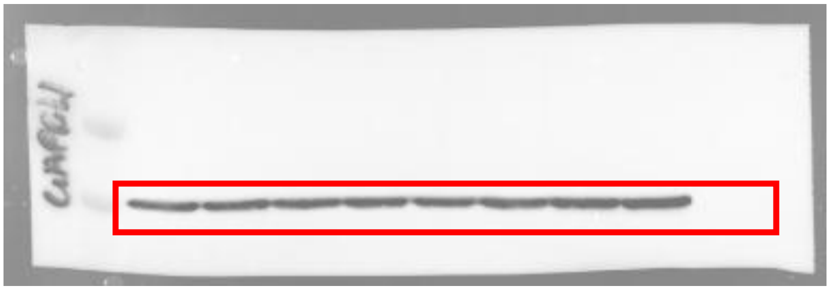

Full Unedited Blots for Figure 1H VCaP EnzaR

Lanes:0, 2, 5, 10, 15, 30, 60, 120

P-HER2

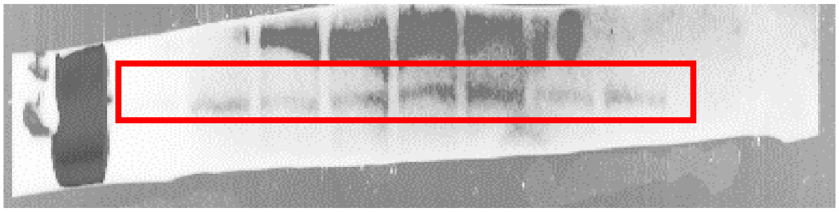

HER2

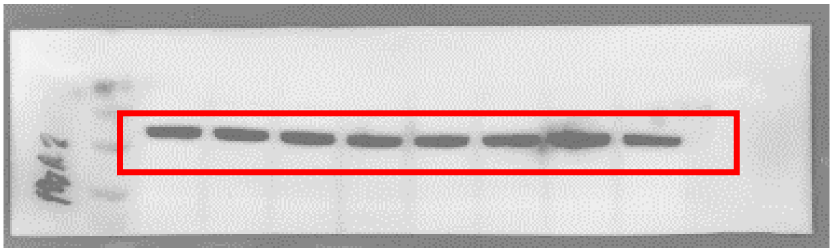

P-HER3

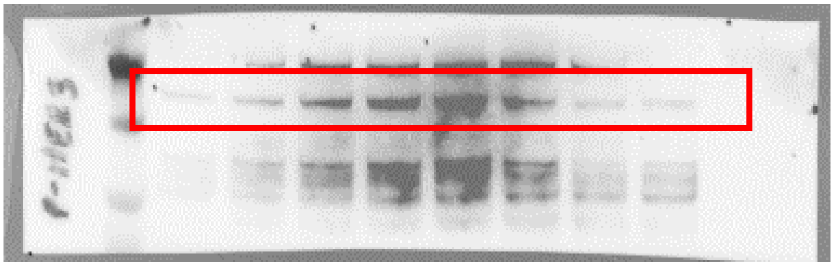

HER3

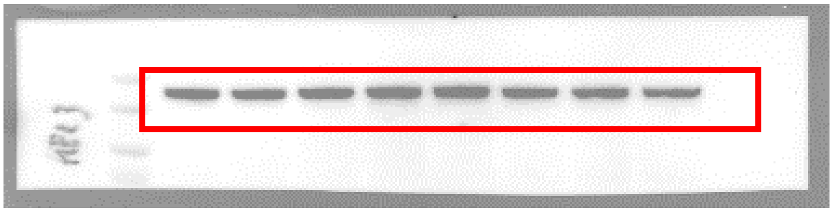

GAPDH

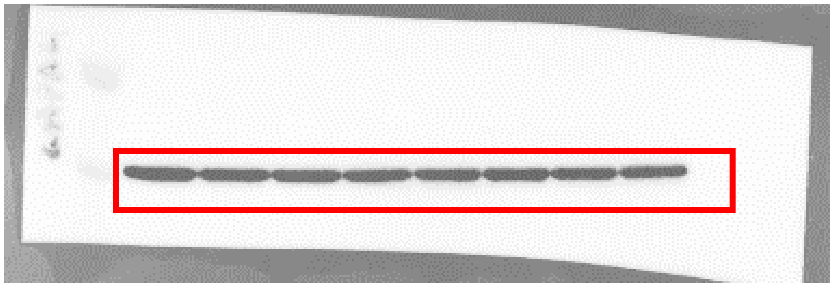

Full Unedited Blots for Figure 1H PC3

Lanes:0, 2, 5, 10, 15, 30, 60, 120

P-HER2

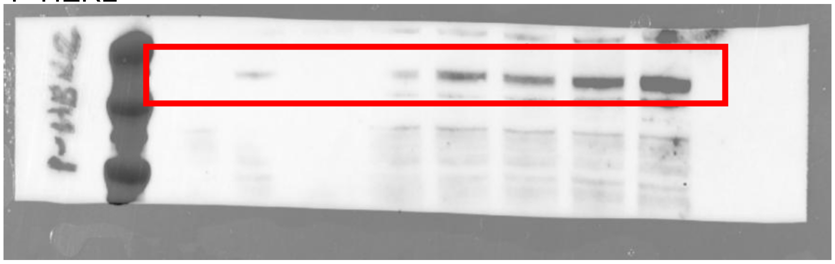

HER2

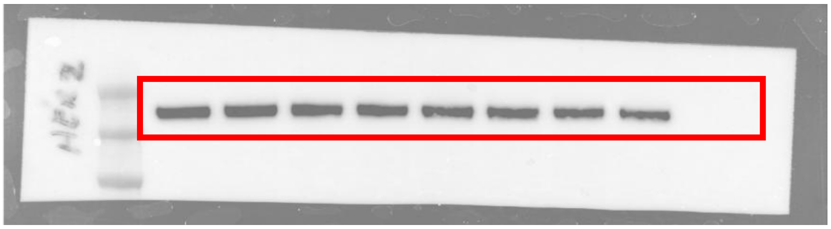

P-HER3

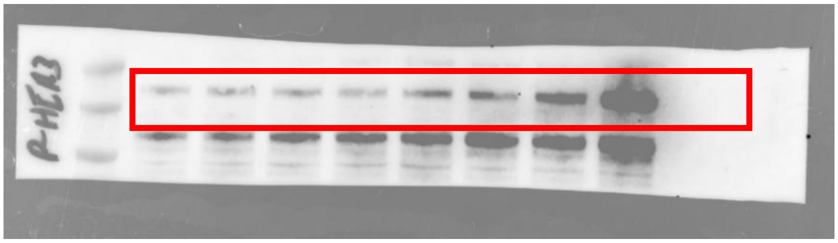

HER3

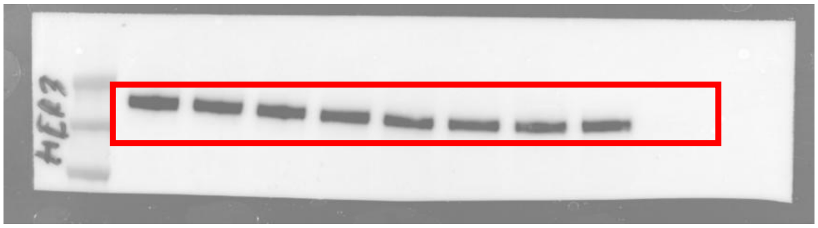

GAPDH

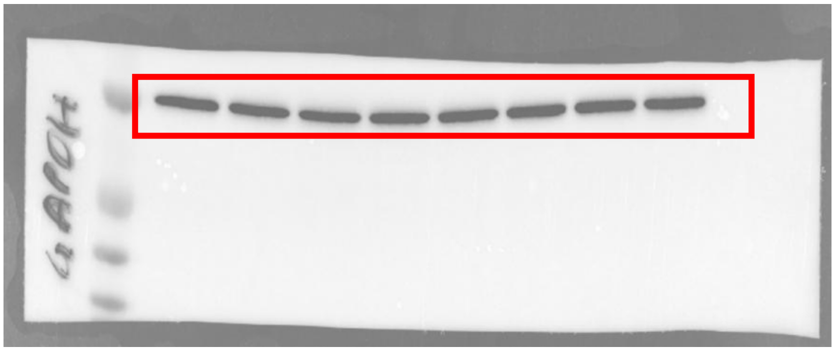

Full Unedited Blots for Figure 2A

Lanes: Hi Calcium, 0, 2, 5, 10, 15, 30, 60, 120 unless noted

P-HER2

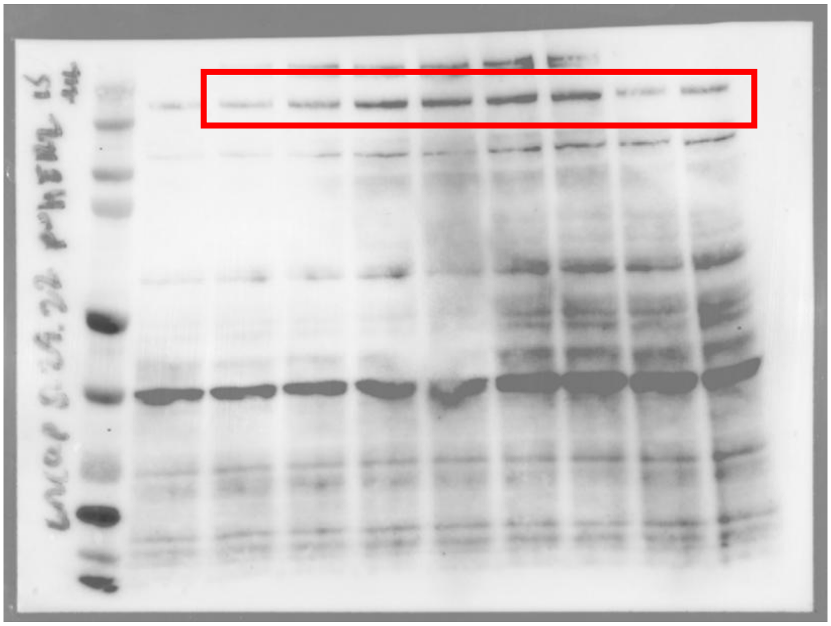

HER2

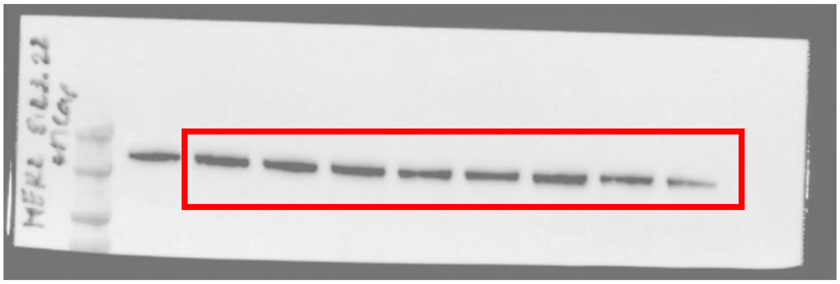

P-HER3

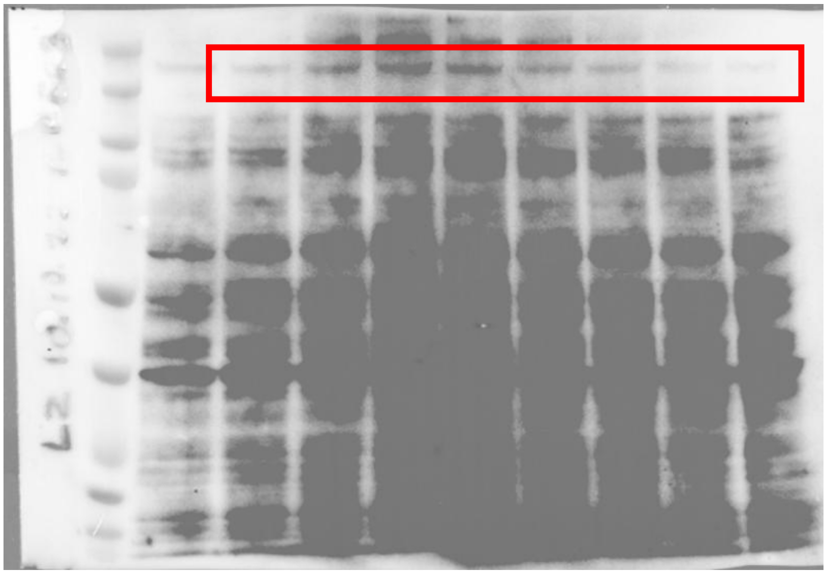

Full Unedited Blots for Figure 2A Continued

HER3

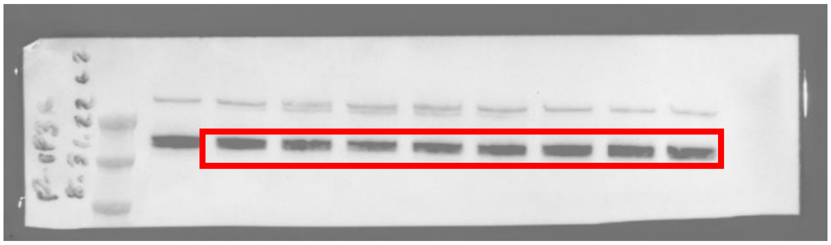

Lanes: EGF control, 0, 2, 5, 10, 15, 30, 60, 120

P-EGFR Y845

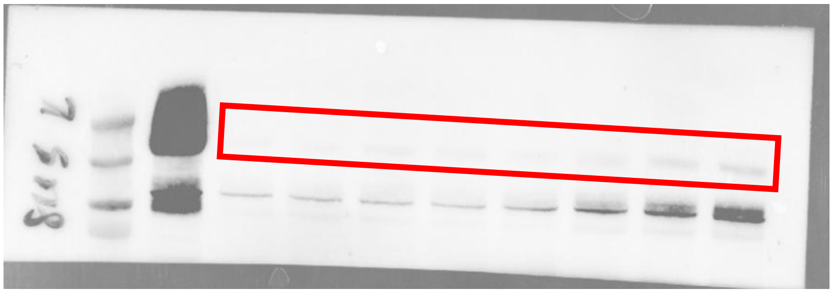

Lanes: EGF control, 0, 2, 5, 10, 15, 30, 60, 120

P-EGFR Y1173

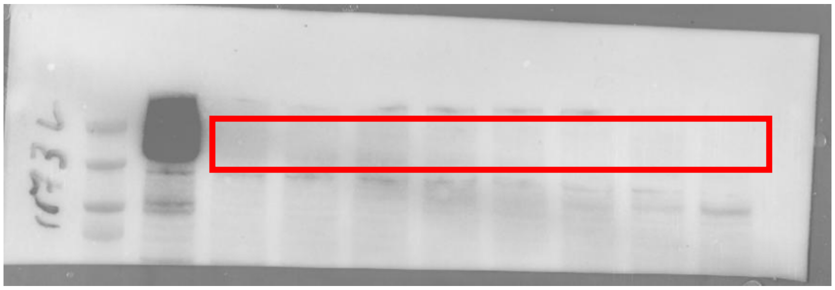

Lanes: EGF control, 0, 2, 5, 10, 15, 30, 60, 120

EGFR

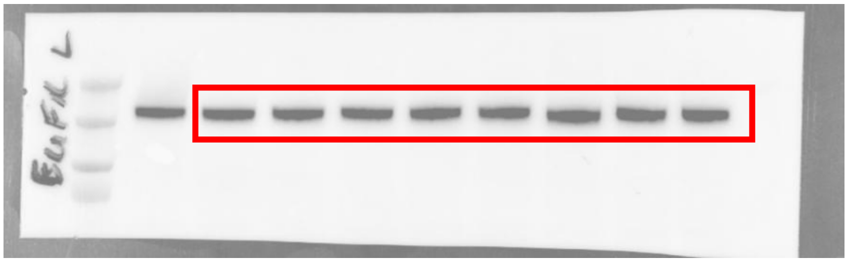

Full Unedited Blots for Figure 2A Continued

P-AKT

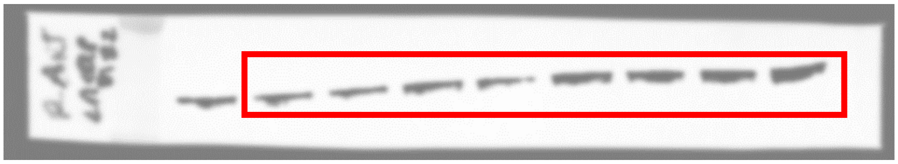

AKT

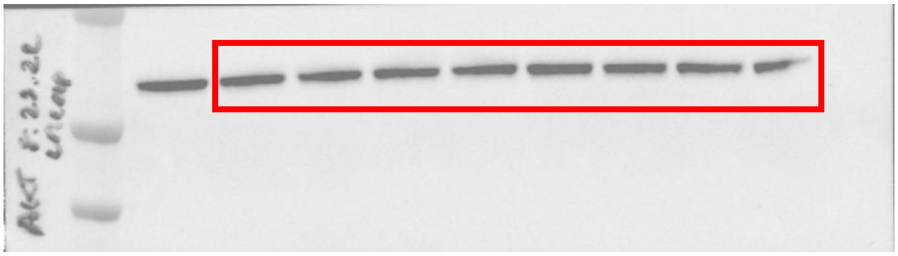

P-ERK

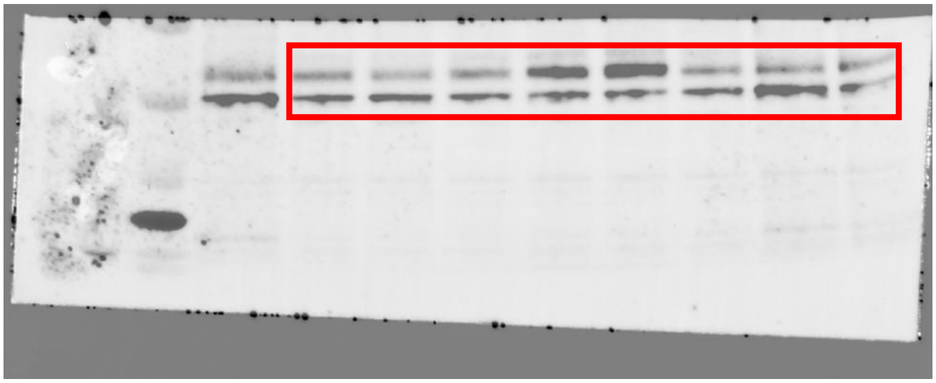

ERK

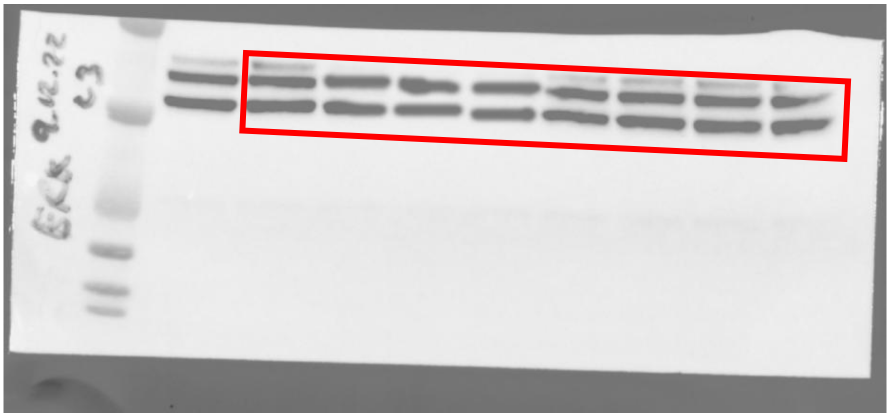

P-PLC

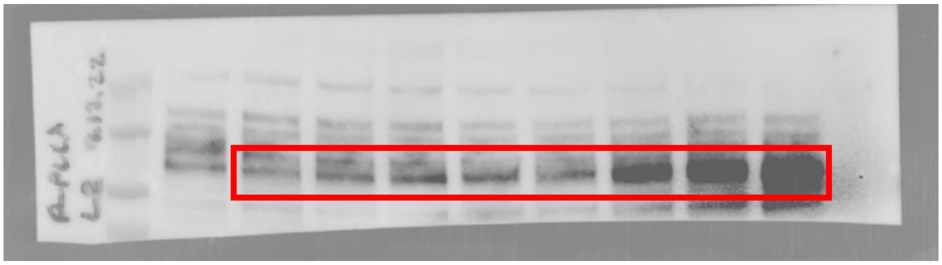

Full Unedited Blots for Figure 2A Continued

PLC

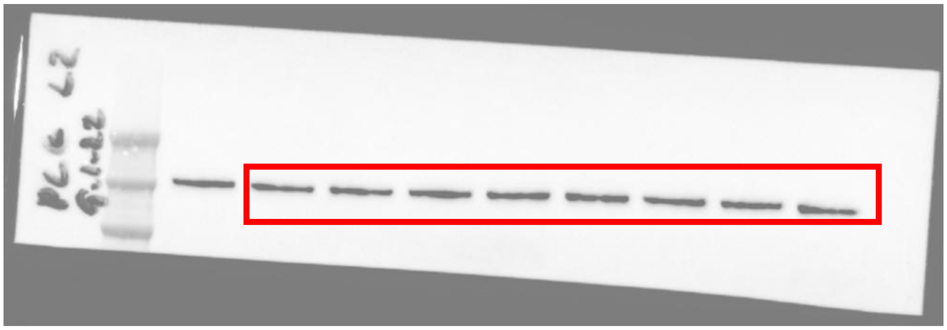

P-CAMKII

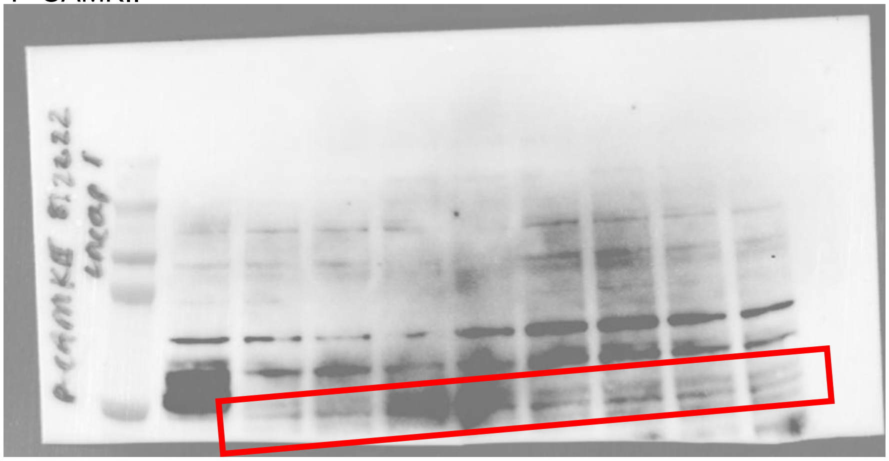

CAMKII

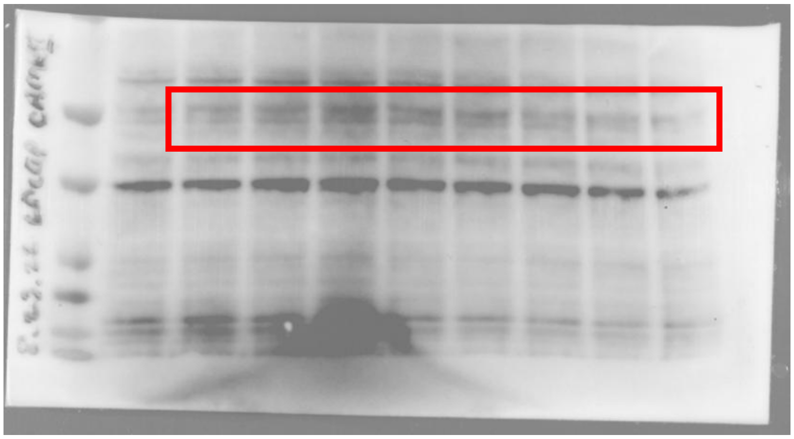

GAPDH

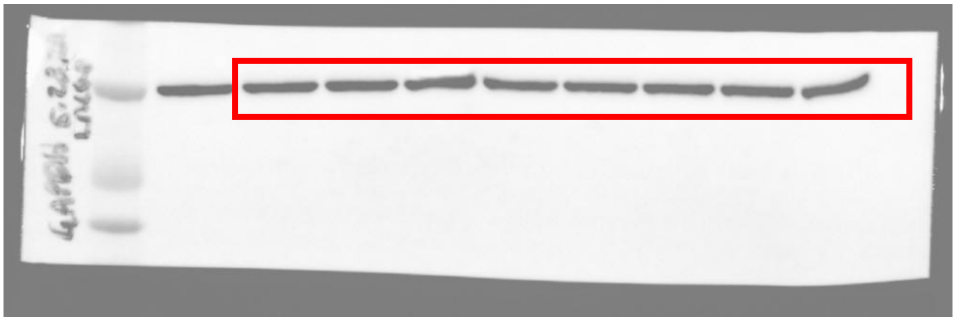

Full Unedited Blots for Figure 2C

Lanes: Hi Calcium, 0, 2, 5, 10, 15, 30, 60, 120 unless noted

P-HER2

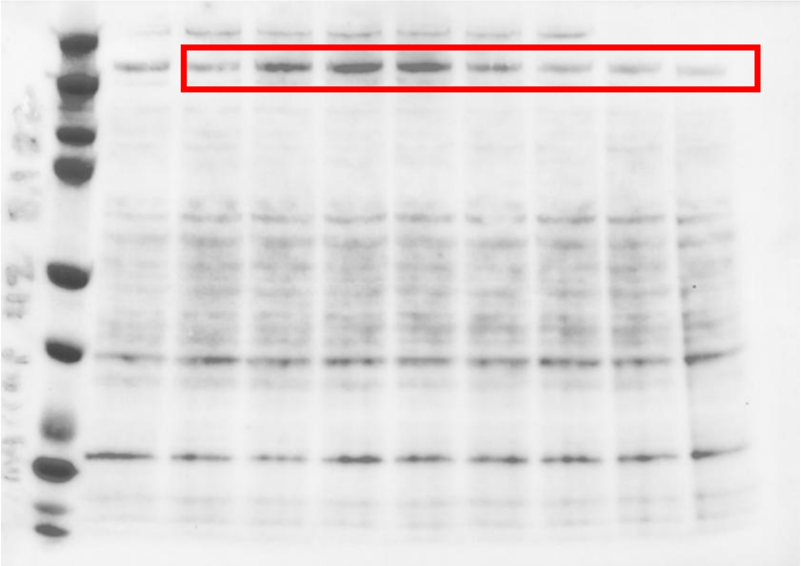

HER2

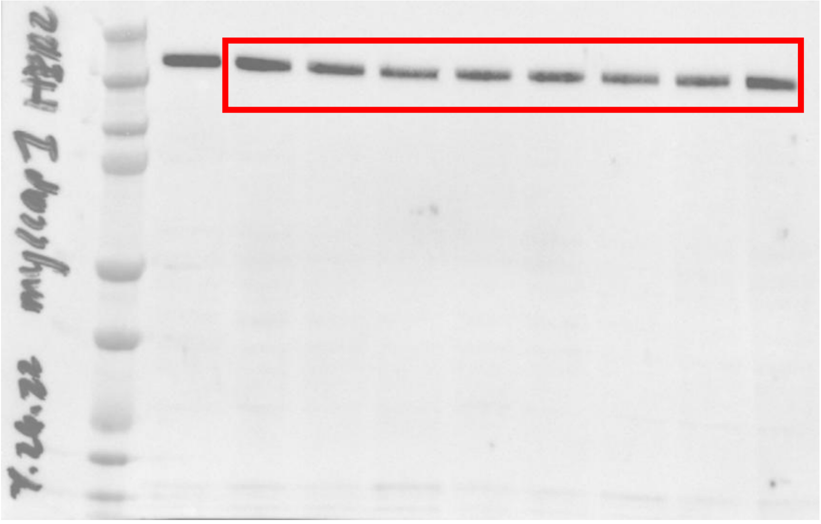

P-HER3

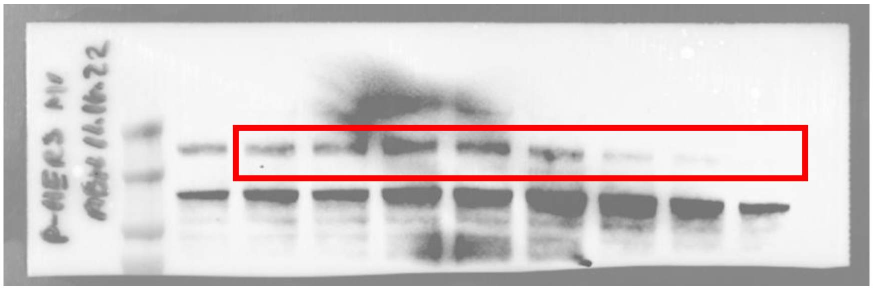

Full Unedited Blots for Figure 2C Continued

HER3

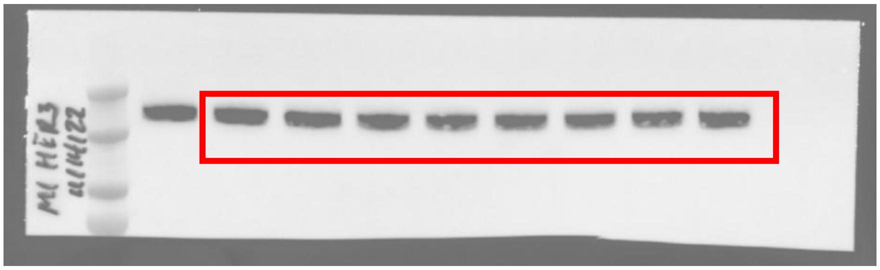

Lanes: EGF control, 0, 2, 5, 10, 15, 30, 60, 120

P-EGFR Y845

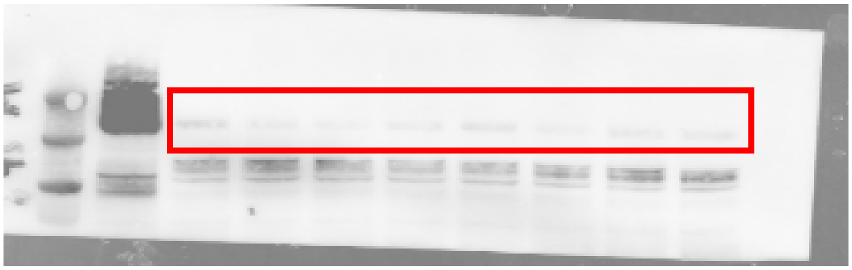

Lanes: EGF control, 0, 2, 5, 10, 15, 30, 60, 120

P-EGFR Y1173

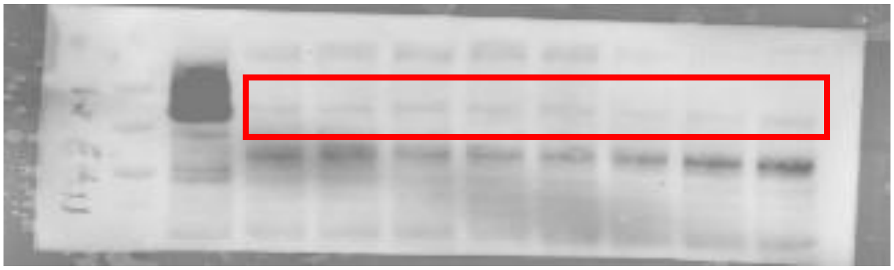

Lanes: EGF control, 0, 2, 5, 10, 15, 30, 60, 120

EGFR

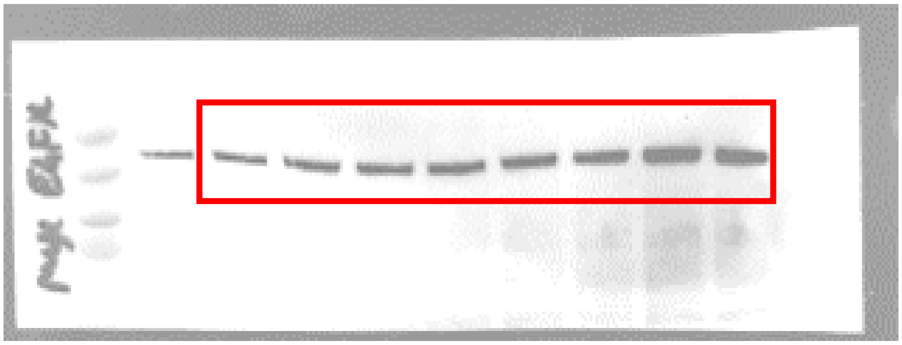

Full Unedited Blots for Figure 2C Continued

P-AKT

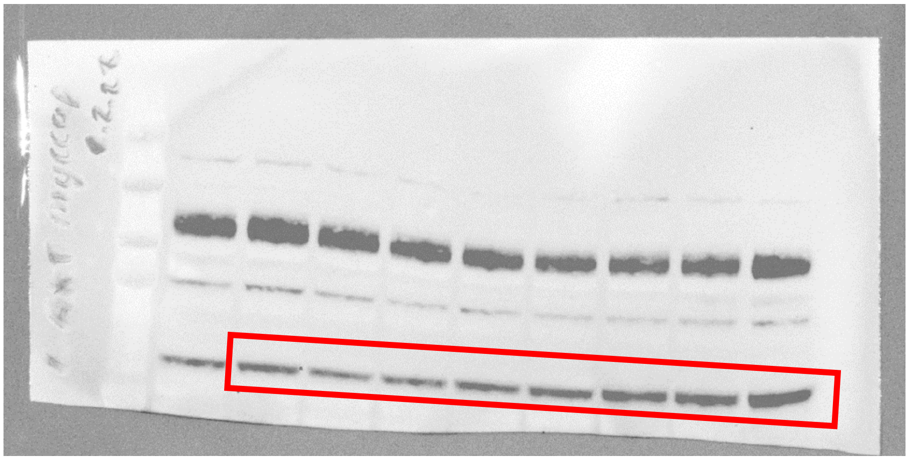

AKT

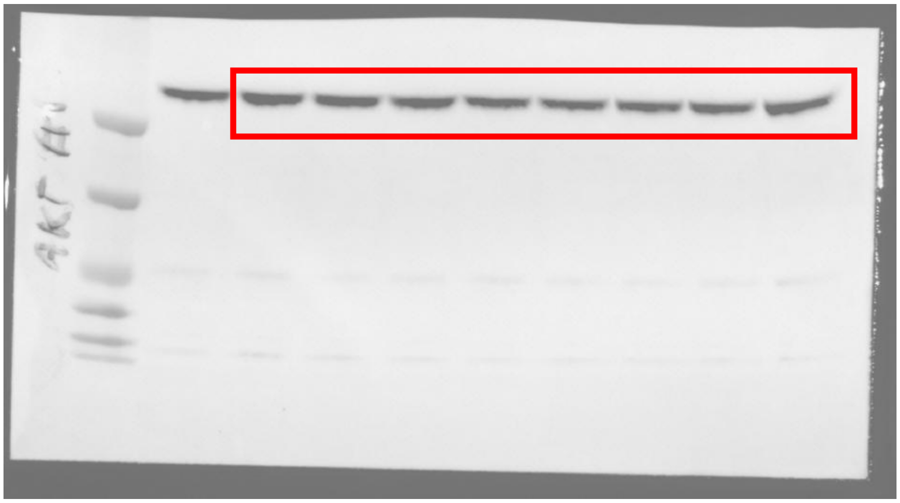

P-ERK

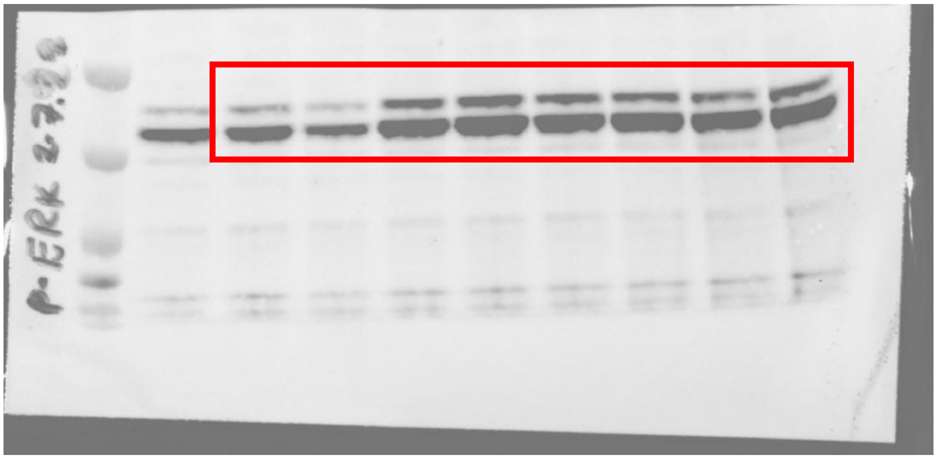

Full Unedited Blots for Figure 2C Continued

ERK note: stripped prior AKT blot

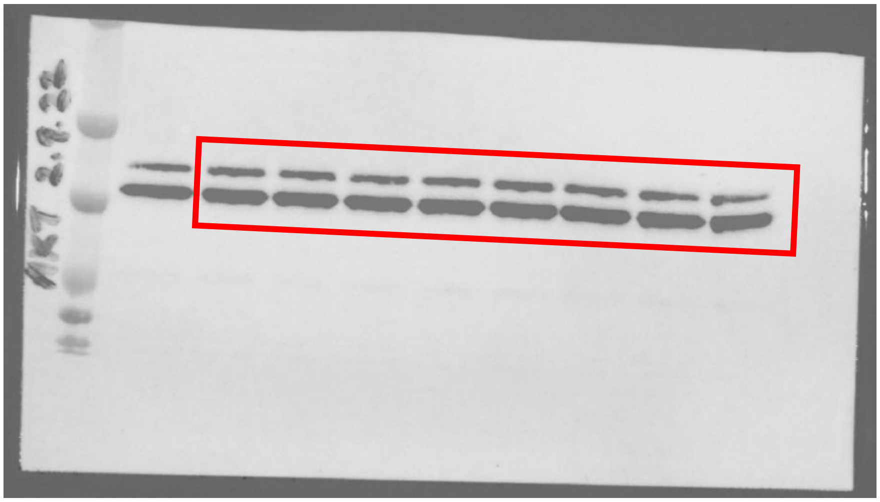

P-PLC

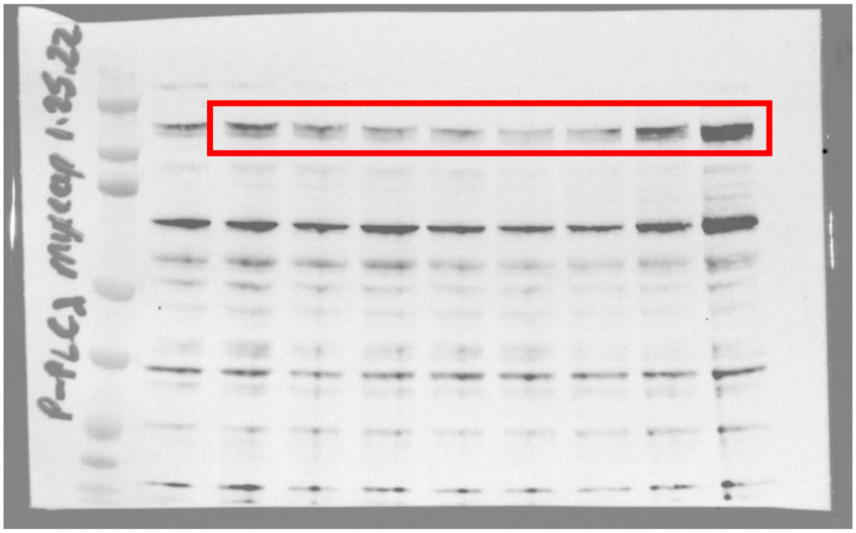

PLC note: stripped prior NFAT blot

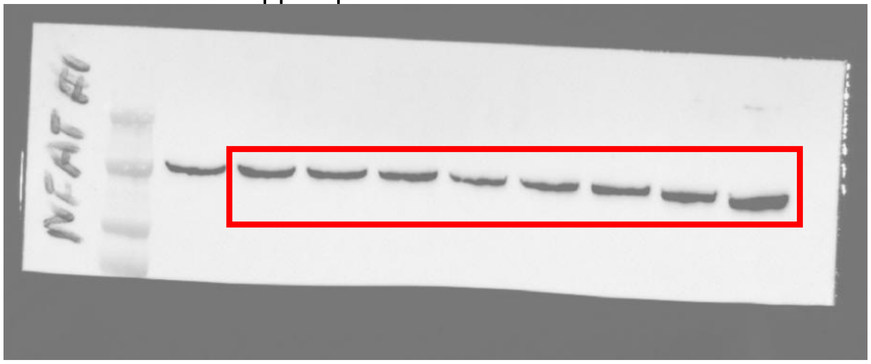

Full Unedited Blots for Figure 2C Continued

P-CAMKII

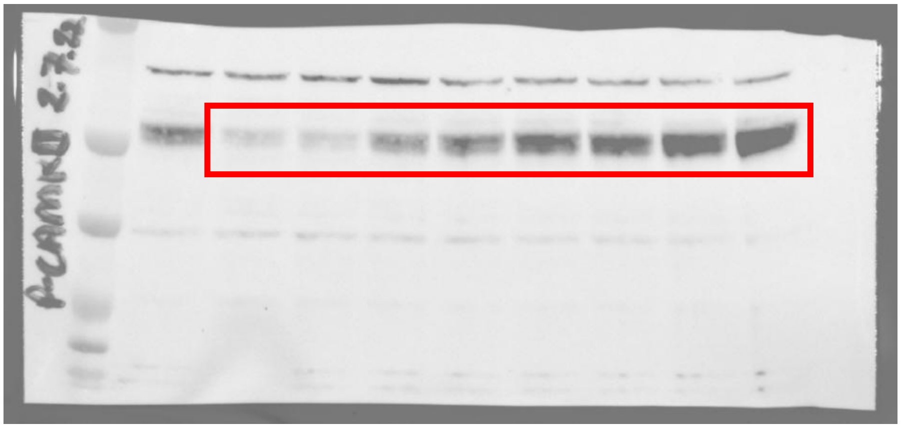

CAMKII

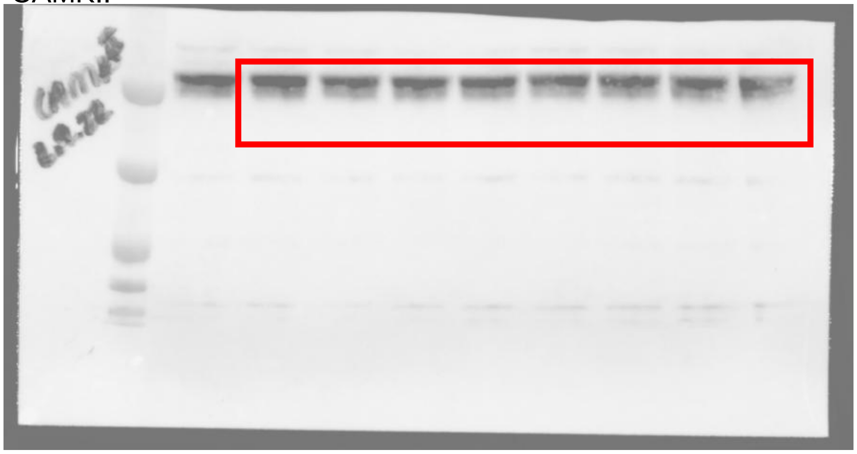

GAPDH

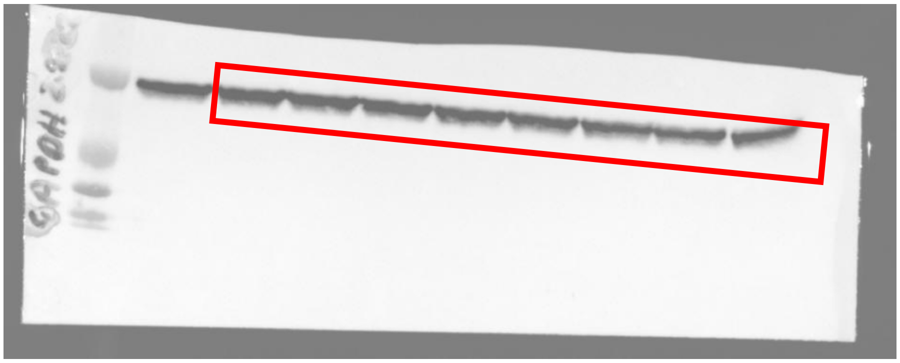

Full Unedited Blots for Figure 3A

Lanes: 0, 2, 5, 10, 15, 30, 60, 120 unless noted

P-HER2

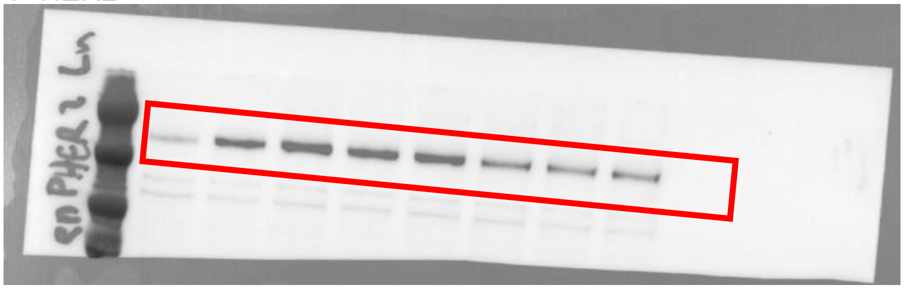

HER2

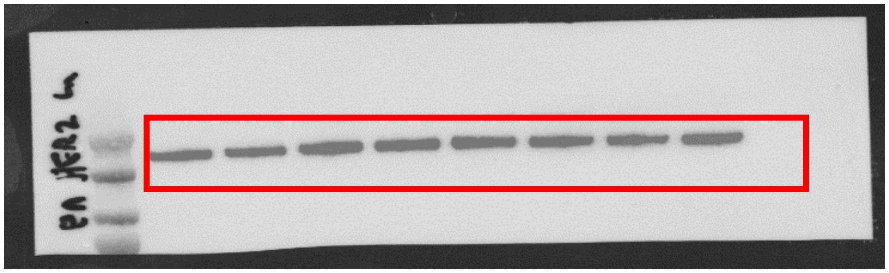

P-HER3

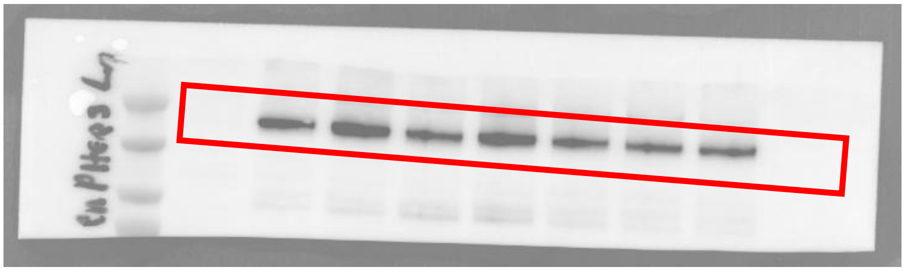

HER3

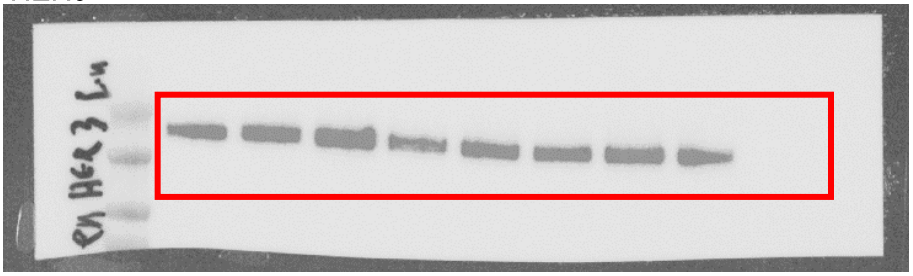

Full Unedited Blots for Figure 3A Continued

P-EGFR Y845

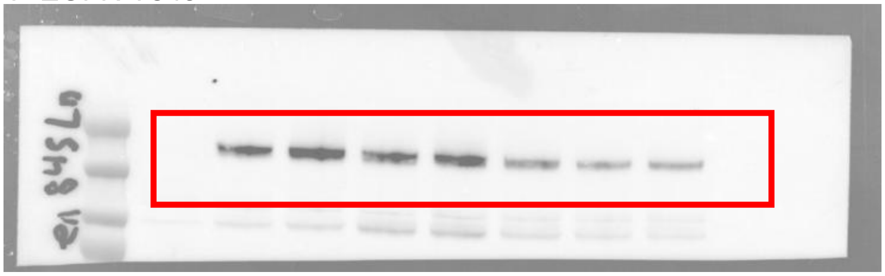

P-EGFR Y1173

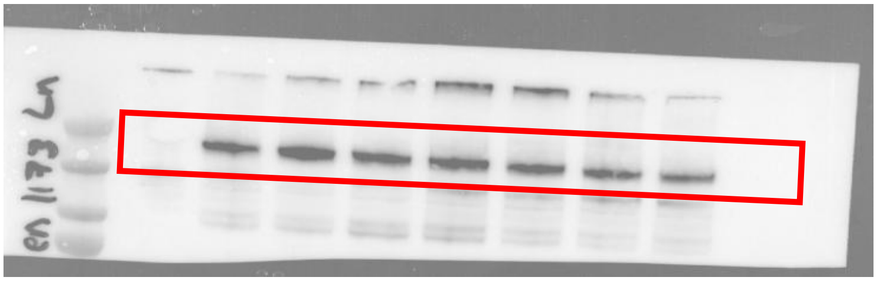

EGFR

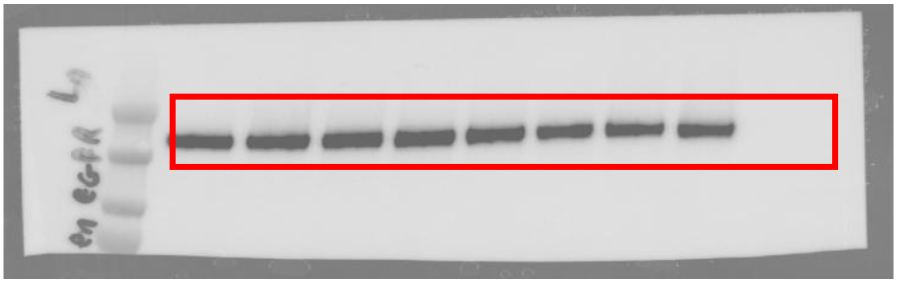

P-AKT

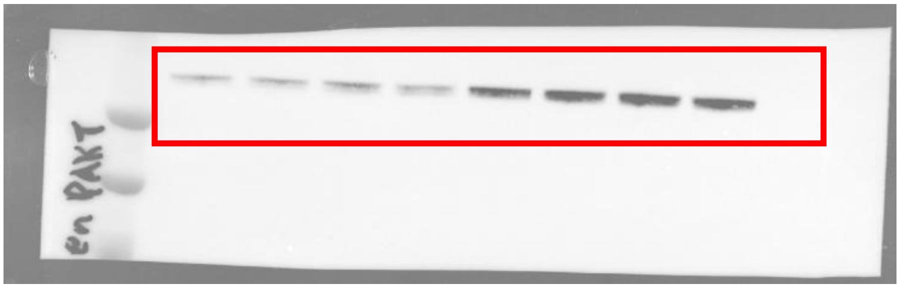

AKT

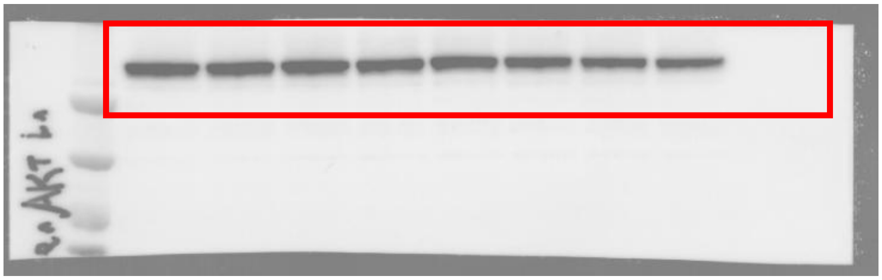

Full Unedited Blots for Figure 3A Continued

P-ERK

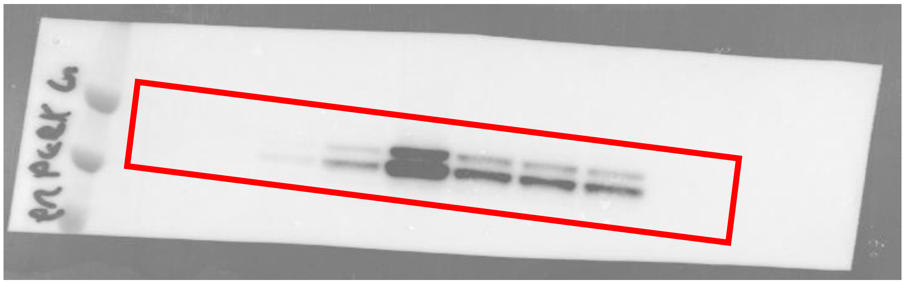

ERK

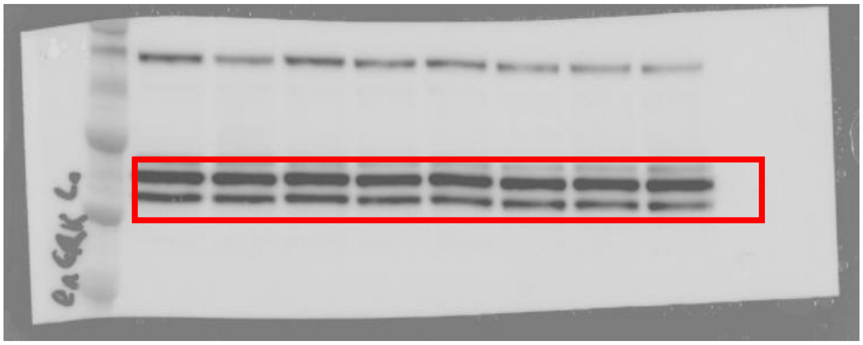

P-PLC

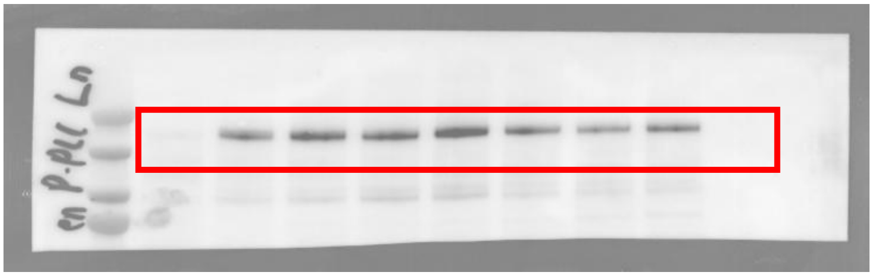

PLC

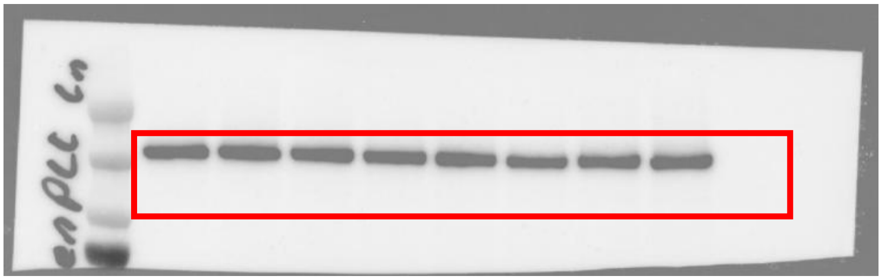

Full Unedited Blots for Figure 3A Continued

P-CAMKII

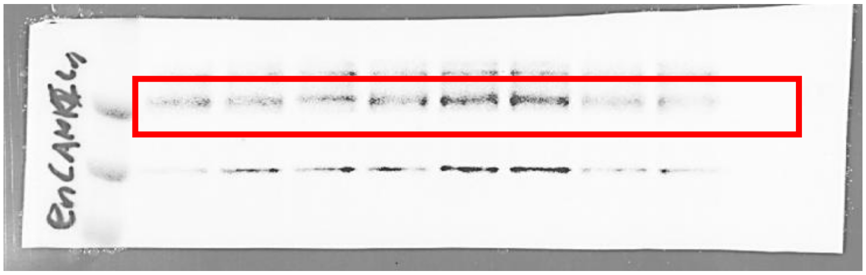

CAMKII

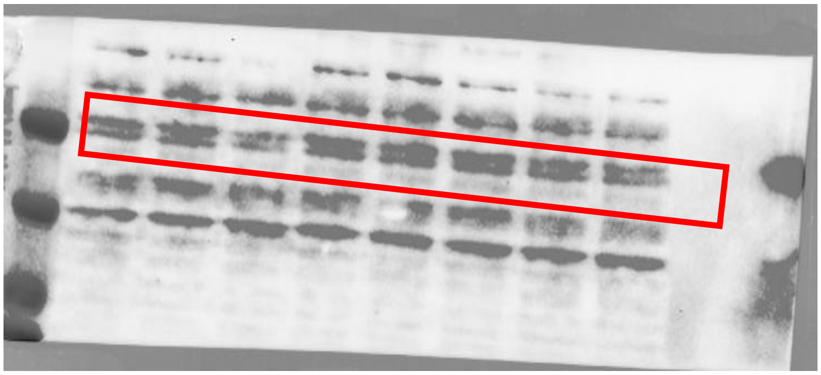

GAPDH

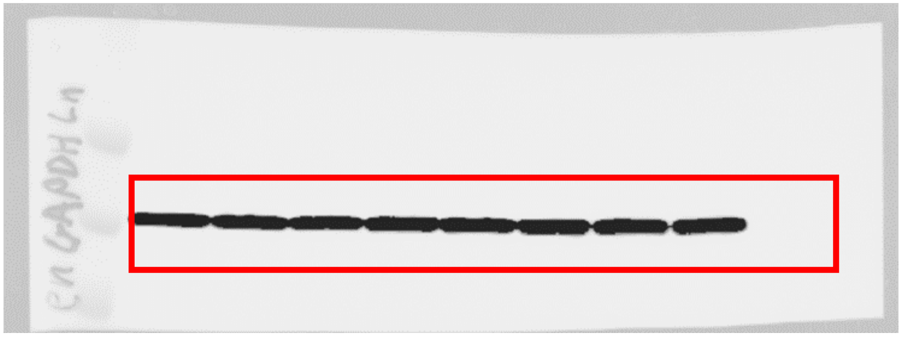

Full Unedited Blots for Figure 3C

Lanes: 0, 2, 5, 10, 15, 30, 60, 120 unless noted

P-HER2

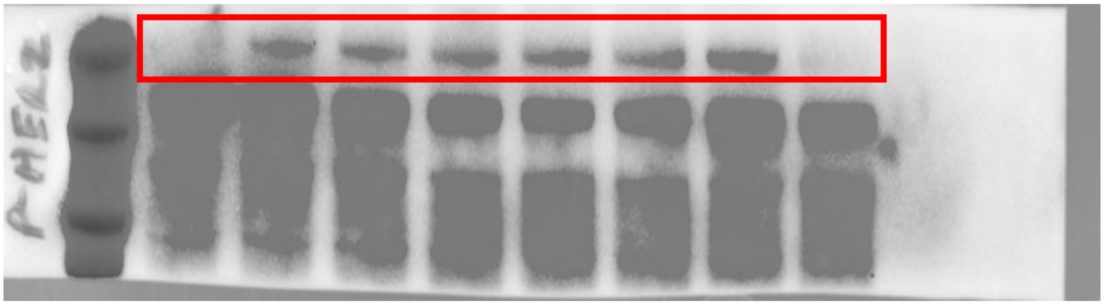

HER2

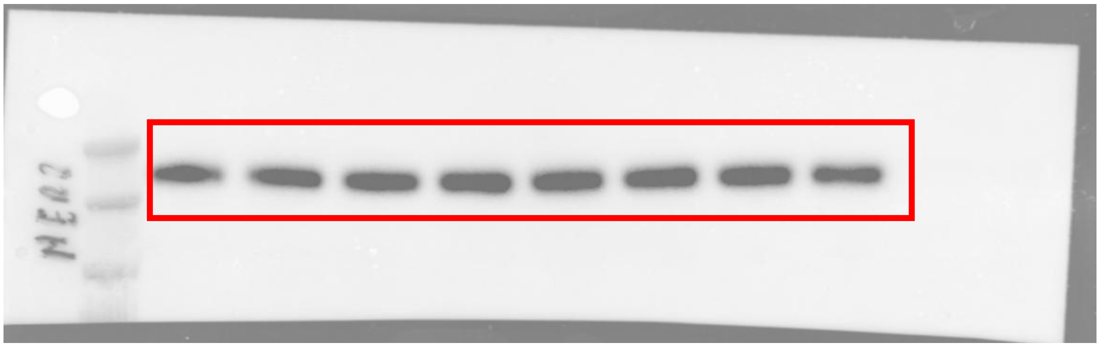

P-HER3

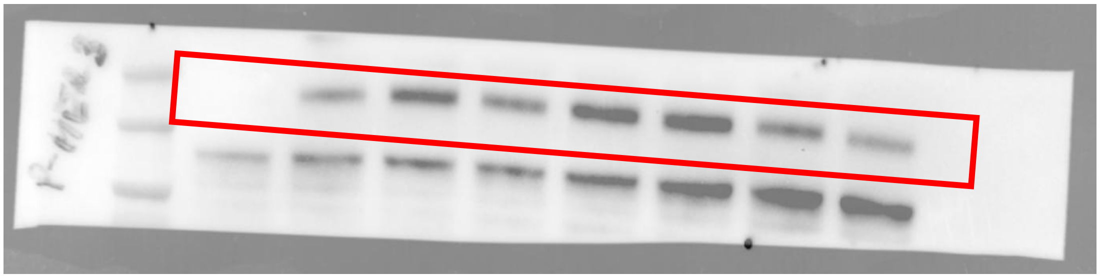

HER3

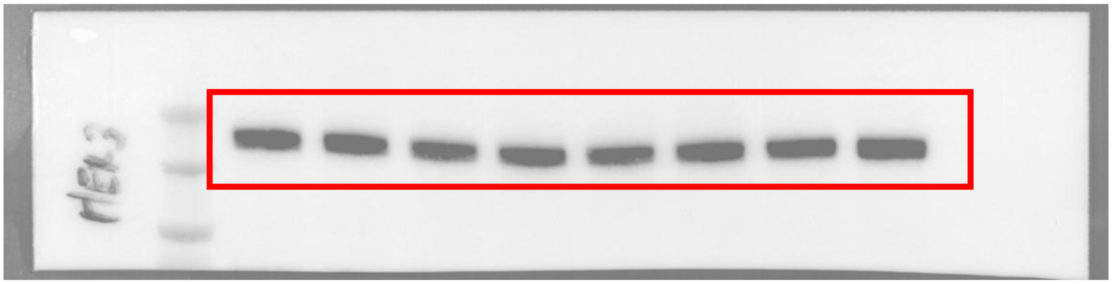

Full Unedited Blots for Figure 3C Continued

P-EGFR Y845

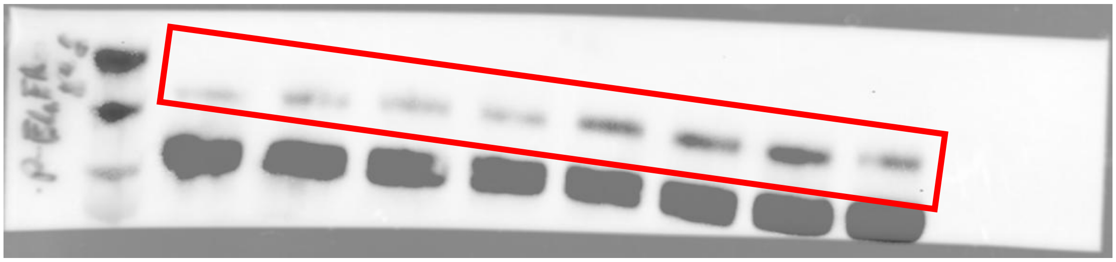

P-EGFR Y1173

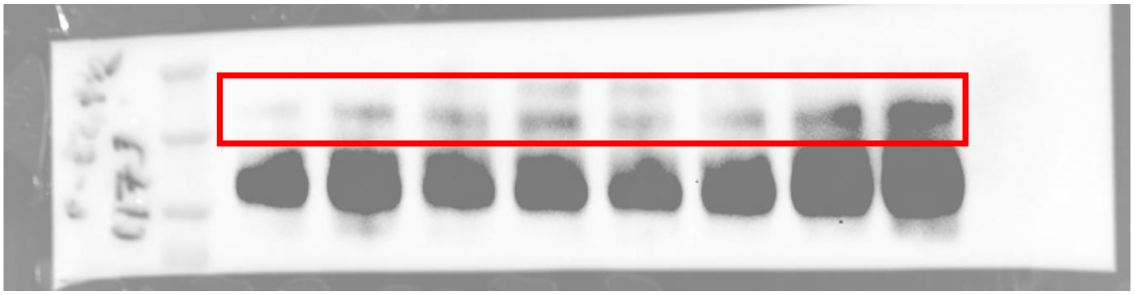

EGFR

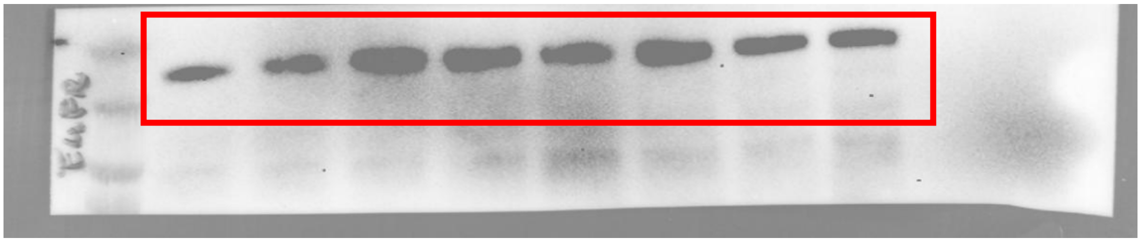

P-AKT

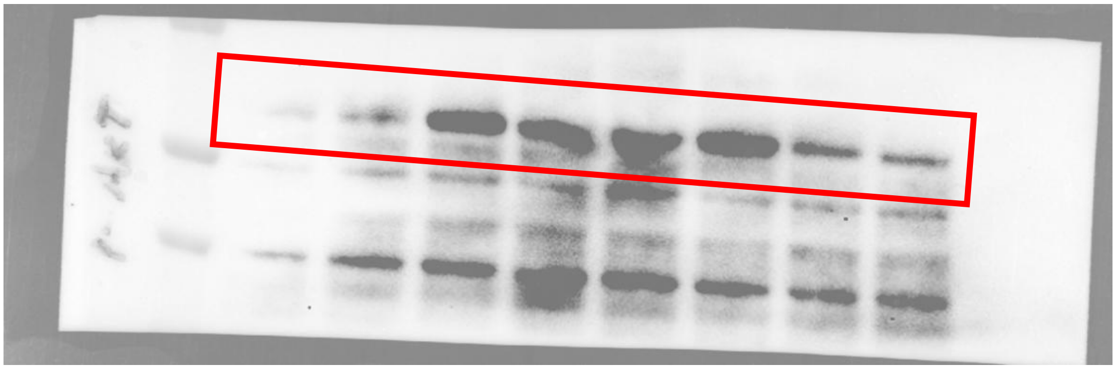

Full Unedited Blots for Figure 3C Continued

AKT

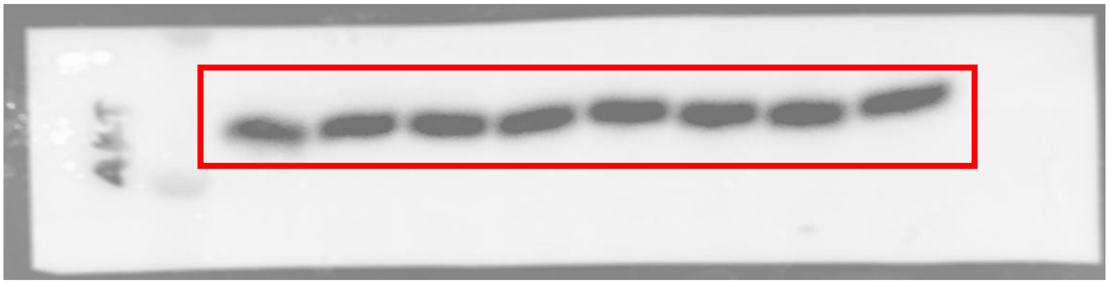

P-ERK

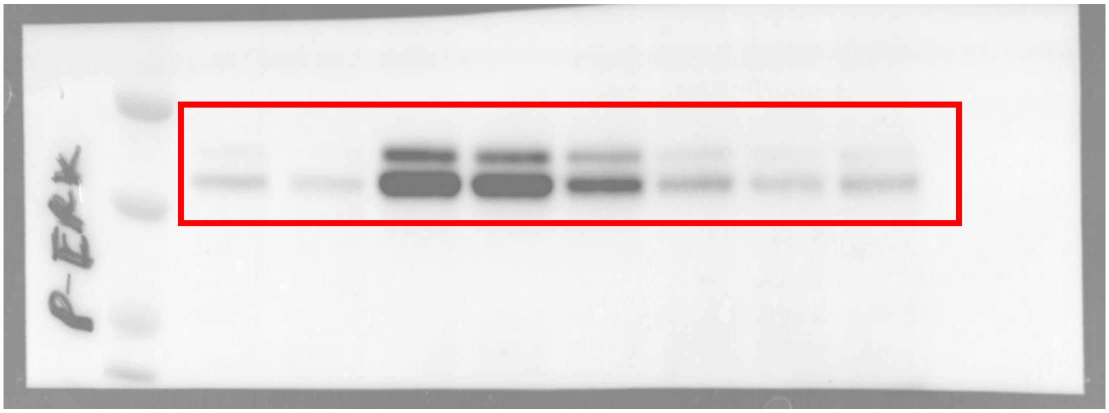

ERK

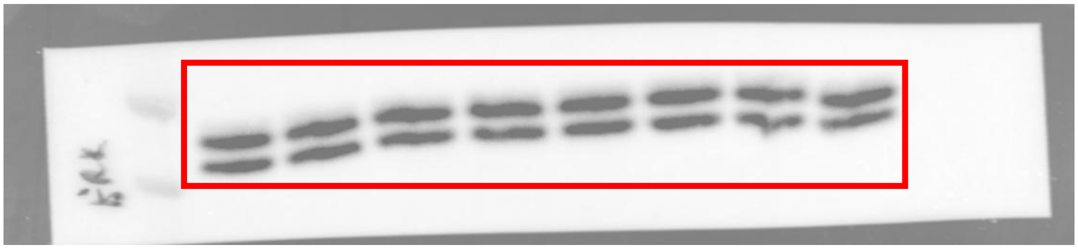

P-PLC

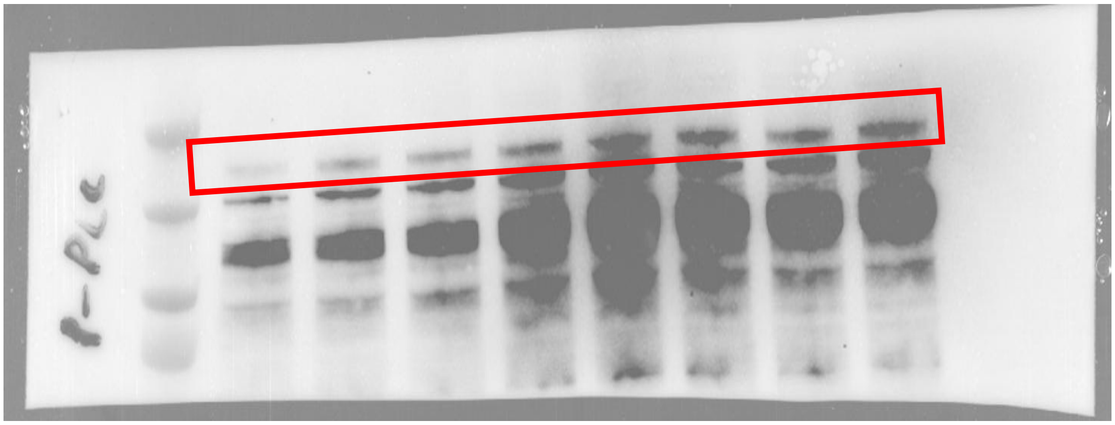

Full Unedited Blots for Figure 3C Continued

PLC

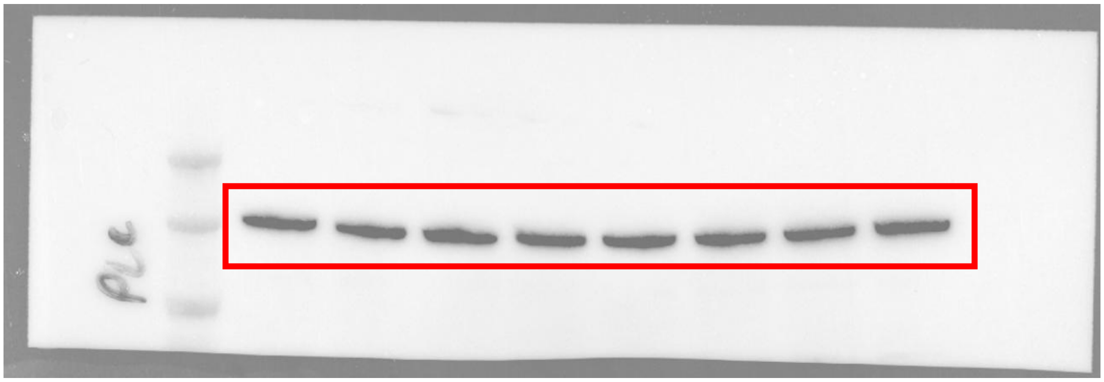

P-CAMKII

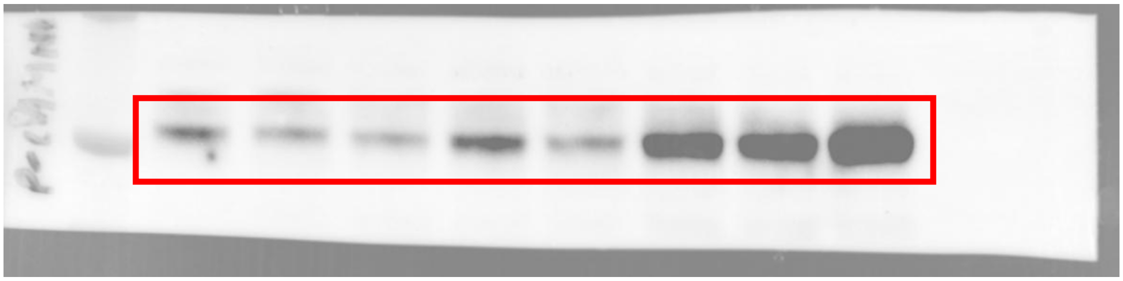

CAMKII

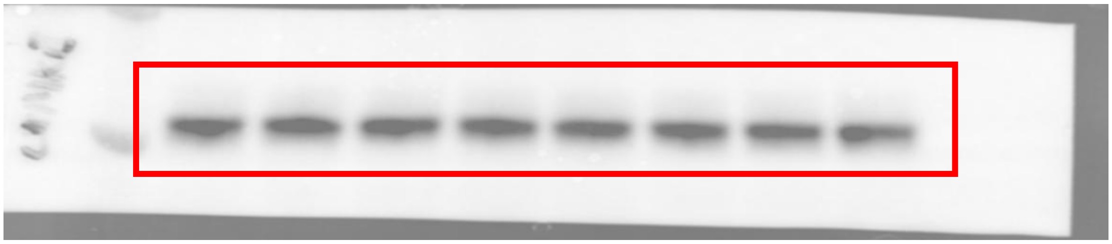

GAPDH

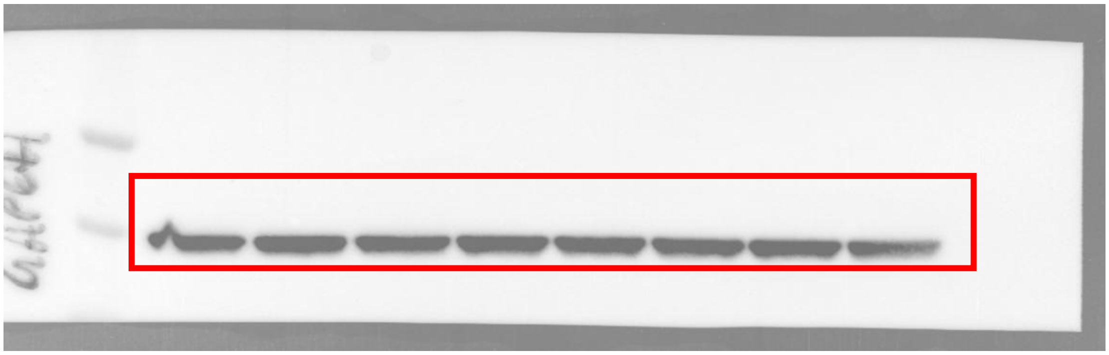

Full Unedited Blots for Figure 4C
Recombinant Protein - HER3-FLAG

HER3
Lanes: HER3 input, HER3 IP, HER3 Post-IP
ASPN input, ASPN IP, ASPN Post-IP
HER3+ASPN Input, HER3+ASPN IP, HER3+ASPN Post-IP

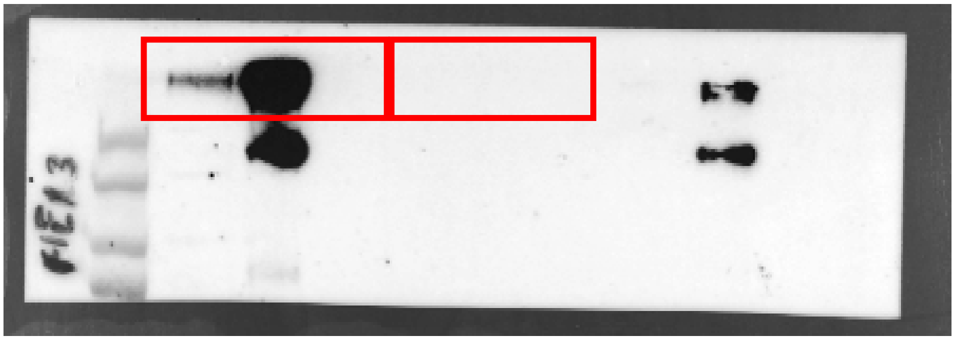

HER3 (exposed longer to draw out band)
Lanes: HER3 input, HER3 IP, HER3 Post-IP
ASPN input, ASPN IP, ASPN Post-IP
HER3+ASPN Input, HER3+ASPN IP, HER3+ASPN Post-IP

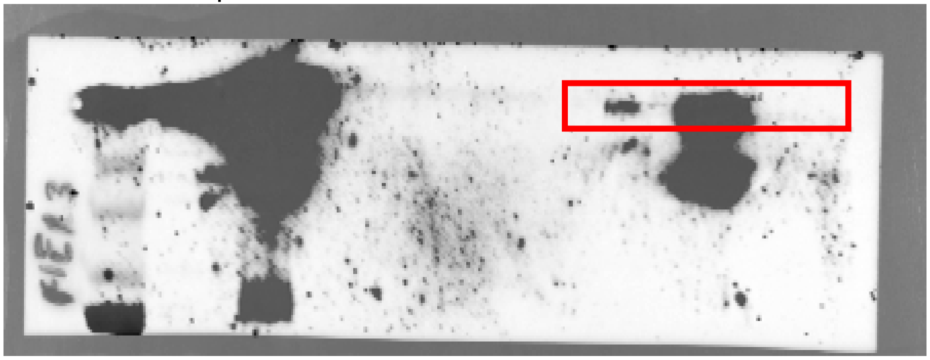

ASPN
Lanes: HER3 input, HER3 IP, HER3 Post-IP
ASPN input, ASPN IP, ASPN Post-IP
HER3+ASPN Input, HER3+ASPN IP, HER3+ASPN Post-IP

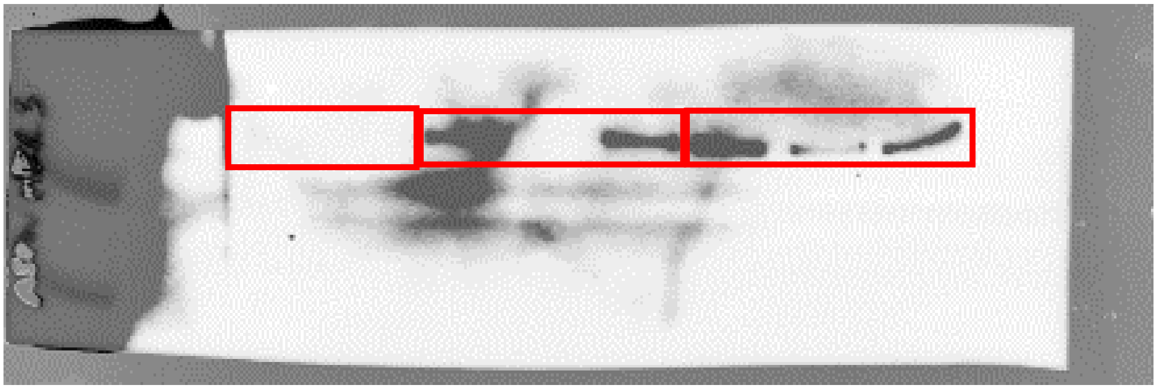

Full Unedited Blots for Figure 4F

HEK293 HER3-FLAG

HER3
Lanes: HER3+EV Input, HER3+EV IP, HER3+EV Post-IP
HER3+ASPN Input, HER3+ASPN IP, HER3+ASPN Post-IP

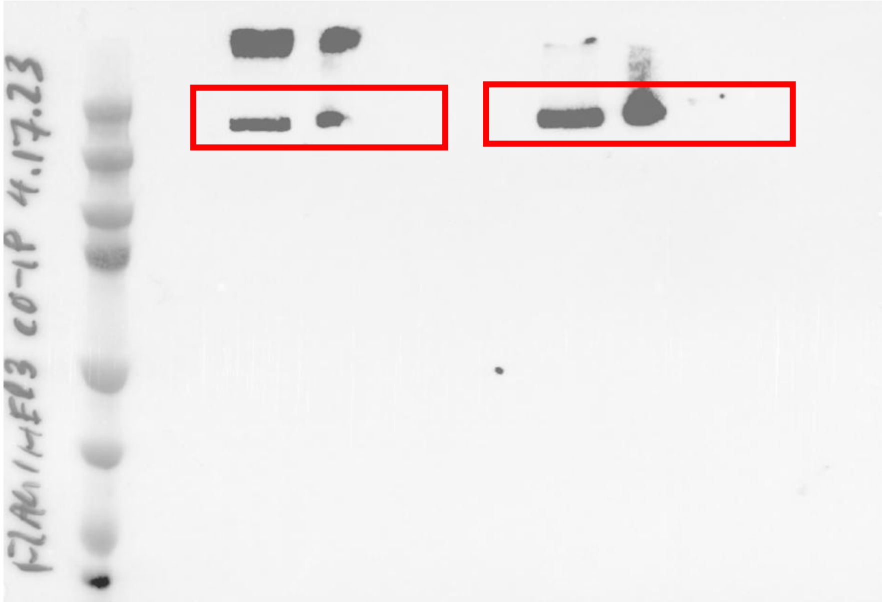

ASPN
Lanes: HER3+EV Input, HER3+EV IP, HER3+EV Post-IP
HER3+ASPN Input, HER3+ASPN IP, HER3+ASPN Post-IP

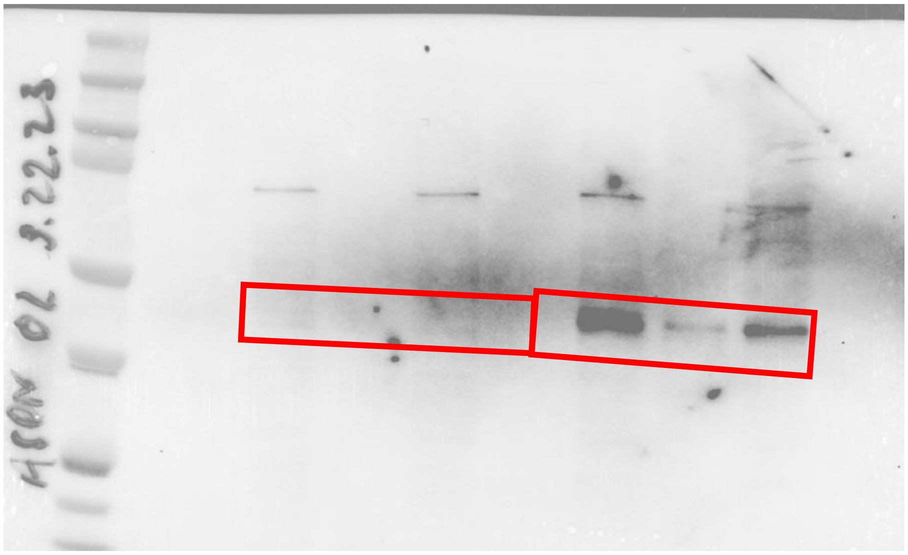

Full Unedited Blots for Figure 4F Continued

HER2

Lanes: HER3+EV Input, HER3+EV IP, HER3+EV Post-IP

HER3+ASPN Input, HER3+ASPN IP, HER3+ASPN Post-IP

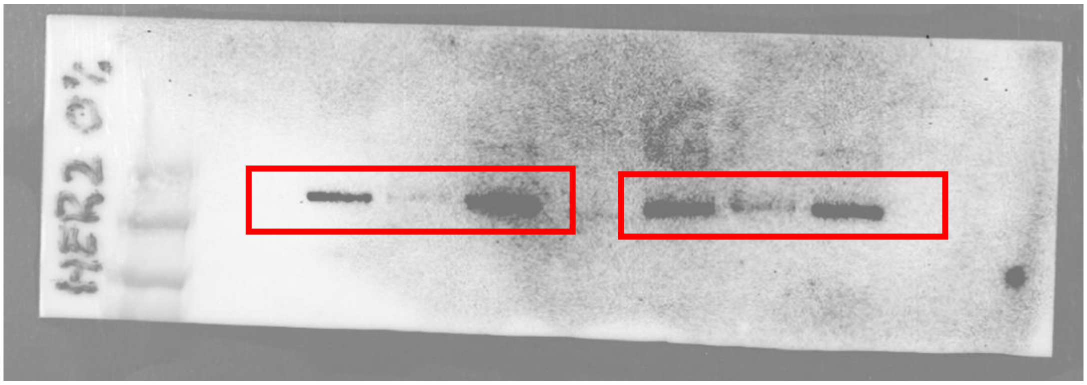

Full Unedited Blots for Figure 4G

LNCaP HER3-FLAG

HER3

Lanes: HER3+EV Input, HER3+EV IP, HER3+EV Post-IP

HER3+ASPN Input, HER3+ASPN IP, HER3+ASPN Post-IP

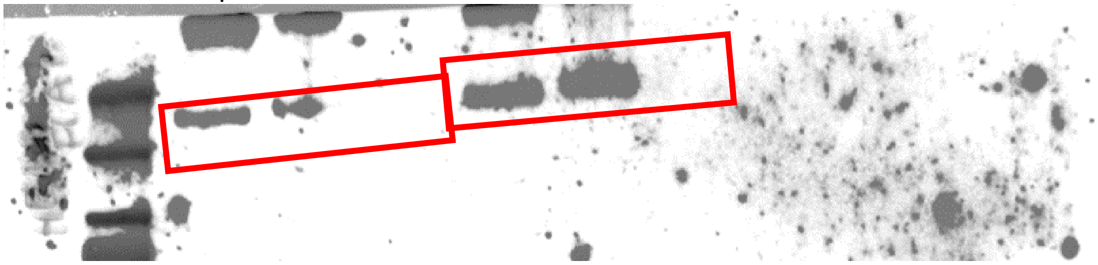

ASPN

Lanes: HER3+EV Input, HER3+EV IP, HER3+EV Post-IP

HER3+ASPN Input, HER3+ASPN IP, HER3+ASPN Post-IP

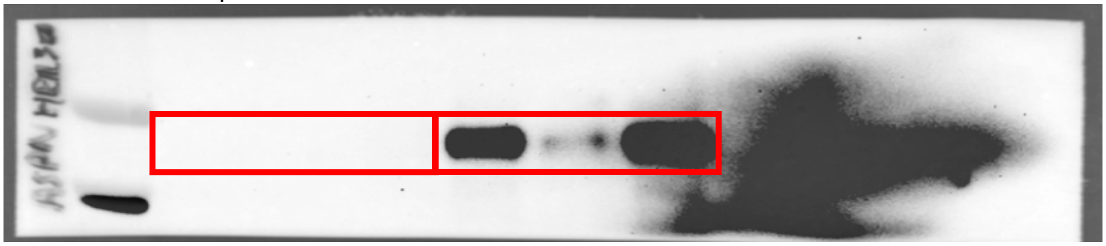

HER2

Lanes: HER3+EV Input, HER3+EV IP, HER3+EV Post-IP

HER3+ASPN Input, HER3+ASPN IP, HER3+ASPN Post-IP

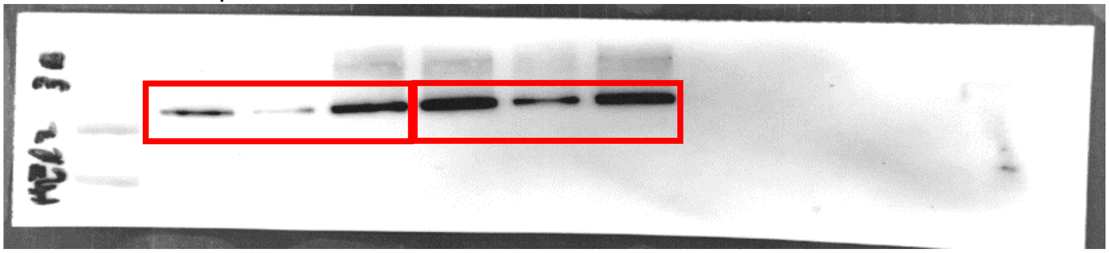

Full Unedited Blots for Figure 4H

LNCaP HER2 KO HER3-FLAG

HER3
Lanes: HER3+EV Input, HER3+EV IP, HER3+EV Post-IP
HER3+ASPN Input, HER3+ASPN IP, HER3+ASPN Post-IP
HER3+ASPN+HER2 Input, HER3+ASPN+HER3 IP, HER3+ASPN+HER2 Post-IP

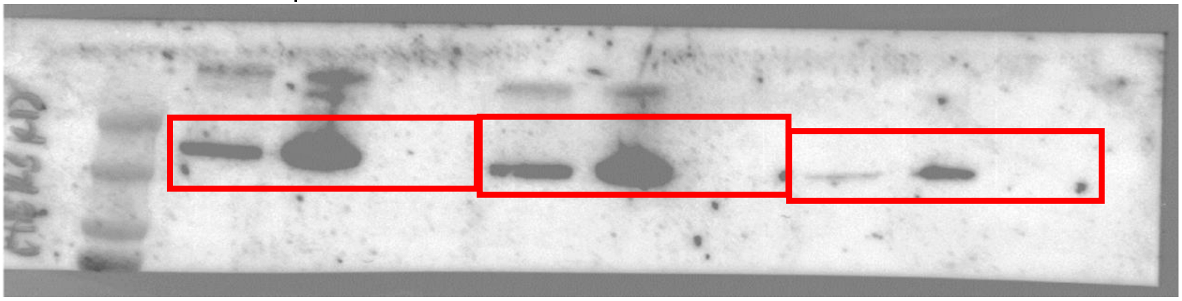

ASPN
Lanes: HER3+EV Input, HER3+EV IP, HER3+EV Post-IP
HER3+ASPN Input, HER3+ASPN IP, HER3+ASPN Post-IP
HER3+ASPN+HER2 Input, HER3+ASPN+HER3 IP, HER3+ASPN+HER2 Post-IP

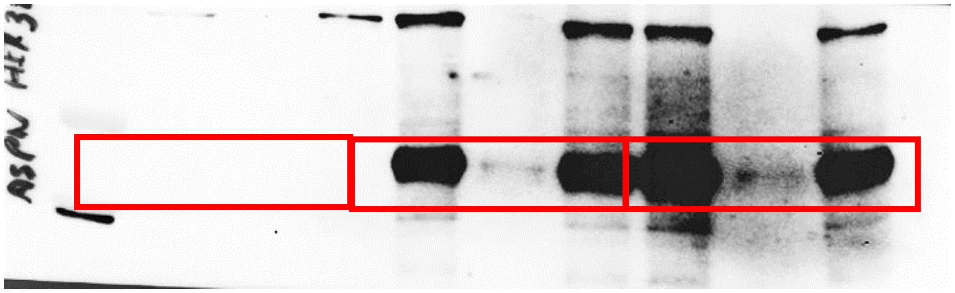

HER2
Lanes: HER3+EV Input, HER3+EV IP, HER3+EV Post-IP
HER3+ASPN Input, HER3+ASPN IP, HER3+ASPN Post-IP
HER3+ASPN+HER2 Input, HER3+ASPN+HER3 IP, HER3+ASPN+HER2 Post-IP

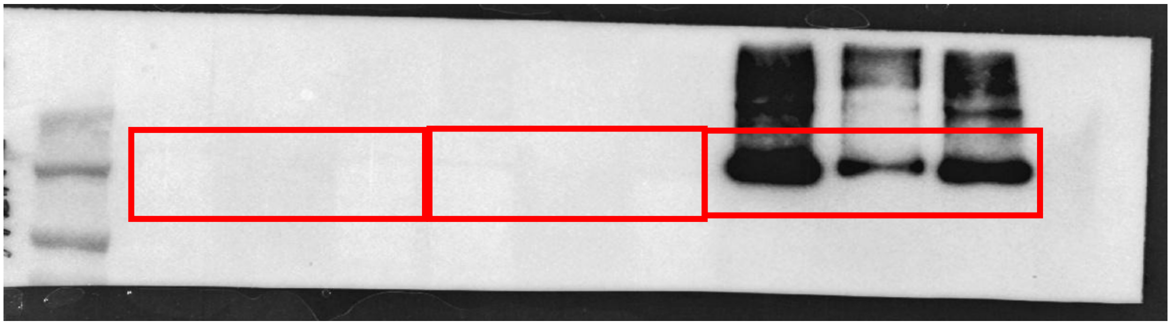

Full Unedited Blots for Figure 4I

HEK293 HER3ΔI-3xFLAG

HER3

Lanes: HER3ΔI+EV Input, HER3ΔI+EV IP, HER3ΔI+EV Post-IP,HER3ΔI+ASPN Input, HER3ΔI+ASPN IP, HER3ΔI+ASPN Post-IP

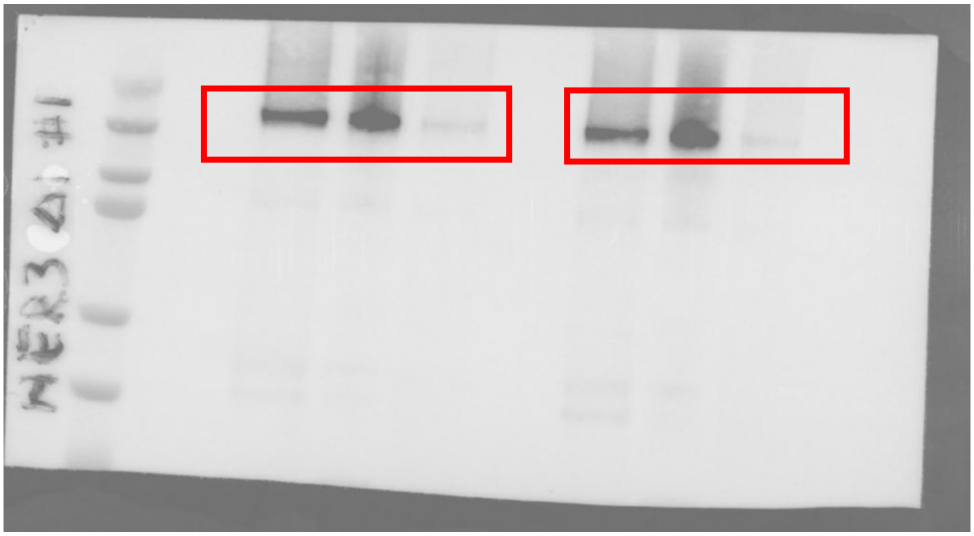

HER2

Lanes: HER3ΔI+EV Input, HER3ΔI+EV IP, HER3ΔI+EV Post-IP,HER3ΔI+ASPN Input, HER3ΔI+ASPN IP, HER3ΔI+ASPN Post-IP

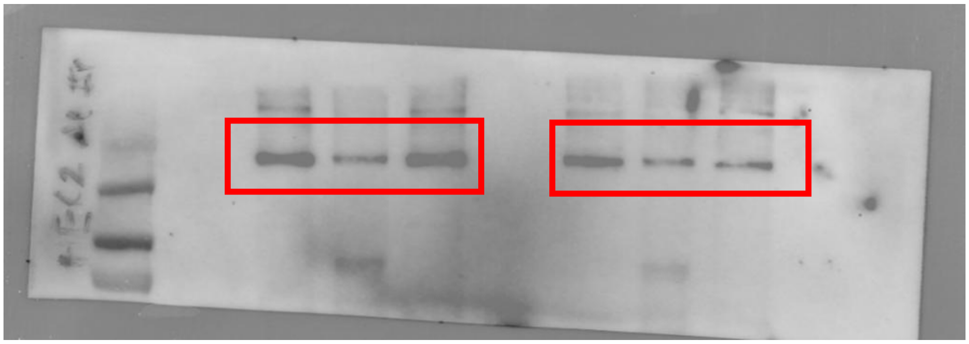

ASPN

Lanes: HER3ΔI+EV Input, HER3ΔI+EV IP, HER3ΔI+EV Post-IP,HER3ΔI+ASPN Input, HER3ΔI+ASPN IP, HER3ΔI+ASPN Post-IP

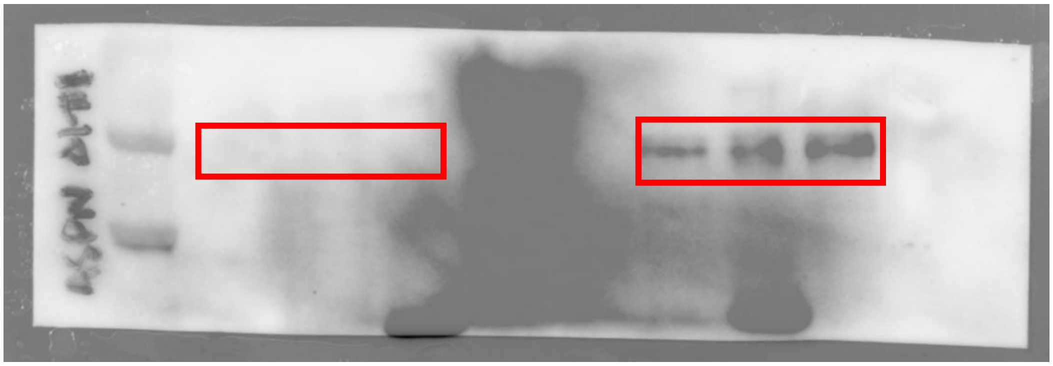

Full and Unedited Blots for Figure 4J

HEK293 HER3ΔIII-3xFLAG

HER3

Lanes: HER3ΔIII+EV Input, HER3ΔIII+EV IP, HER3ΔIII+EV Post-IP, HER3ΔIII+ASPN Input, HER3ΔIII+ASPN IP, HER3ΔIII+ASPN Post-IP

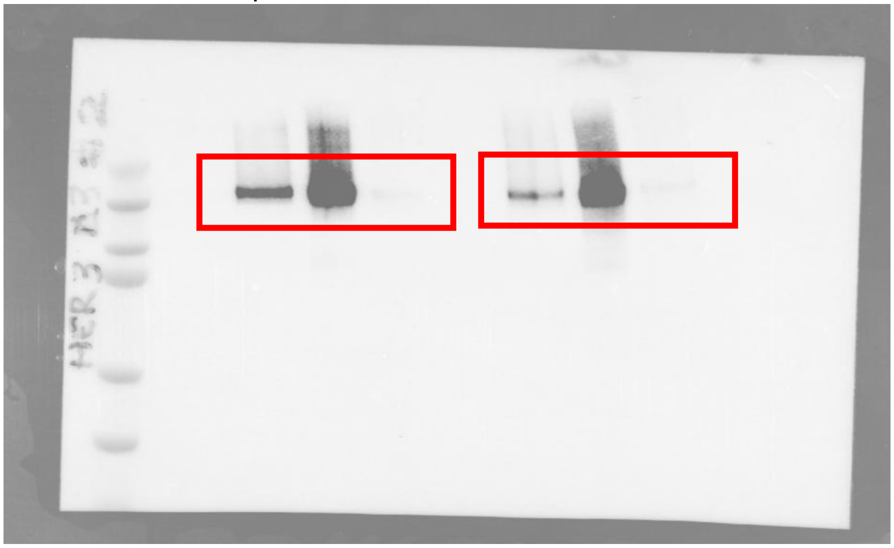

HER2

Lanes: HER3ΔIII+EV Input, HER3ΔIII+EV IP, HER3ΔIII+EV Post-IP, HER3ΔIII+ASPN Input, HER3ΔIII+ASPN IP, HER3ΔIII+ASPN Post-IP

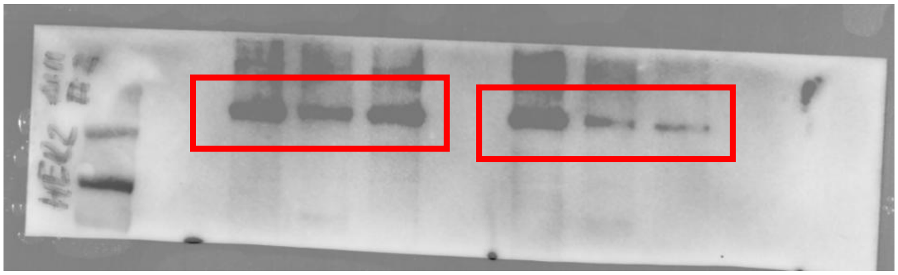

ASPN

Lanes: HER3ΔIII+EV Input, HER3ΔIII+EV IP, HER3ΔIII+EV Post-IP, HER3ΔIII+ASPN Input, HER3ΔIII+ASPN IP, HER3ΔIII+ASPN Post-IP

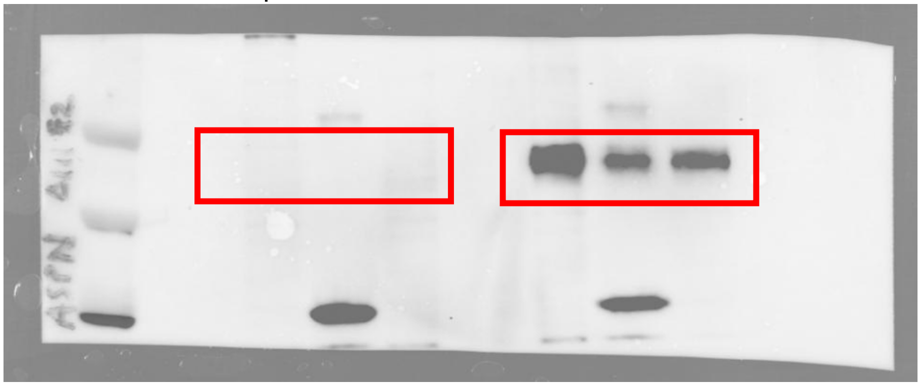

Full and Unedited Figure 4K

HEK293 HER3ΔI/III-3xFLAG

HER3

Lanes: HER3Δ I/III + EV Input, HER3Δ I/III + EV IP, HER3Δ I/III + Post-IP, HER3Δ I/III + ASPN Input, HER3Δ I/III + ASPN IP, HER3Δ I/III + ASPN Post-IP, ASPN only Input, ASPN only IP, ASPN only Post-IP

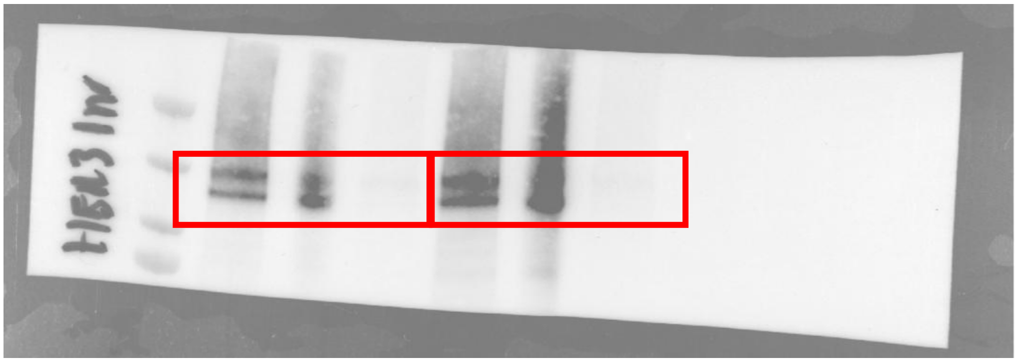

HER2

Lanes: HER3Δ I/III + EV Input, HER3Δ I/III + EV IP, HER3Δ I/III + Post-IP, HER3Δ I/III + ASPN Input, HER3Δ I/III + ASPN IP, HER3Δ I/III + ASPN Post-IP, ASPN only Input, ASPN only IP, ASPN only Post-IP

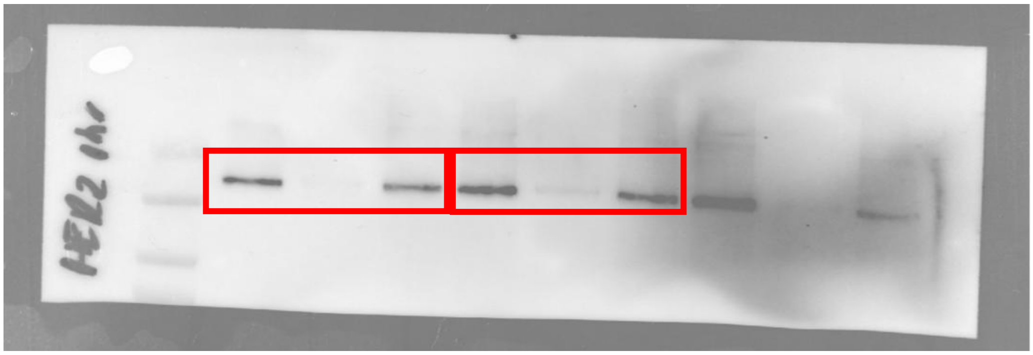

ASPN

Lanes: HER3Δ I/III + EV Input, HER3Δ I/III + EV IP, HER3Δ I/III + Post-IP, HER3Δ I/III + ASPN Input, HER3Δ I/III + ASPN IP, HER3Δ I/III + ASPN Post-IP, ASPN only Input, ASPN only IP, ASPN only Post-IP

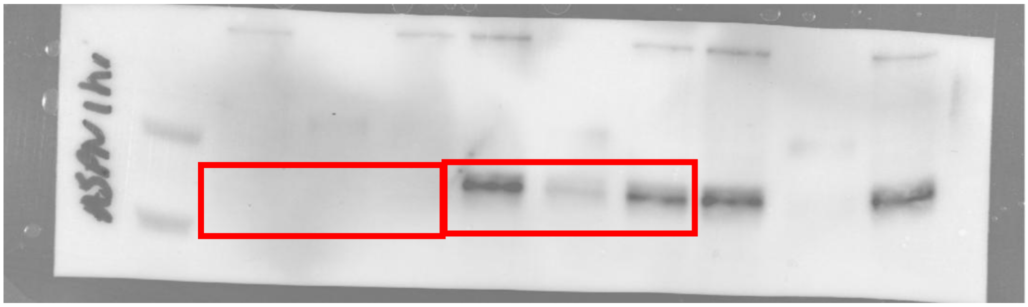

Full and Unedited Blots for Figure 5A

HER3 TWT E11, HER3 TKD E3
Lanes: TWT 0, 5, 10, 15, 30, 60, KD 0, 5, 10, 15, 30, 60 unless noted
P-HER2

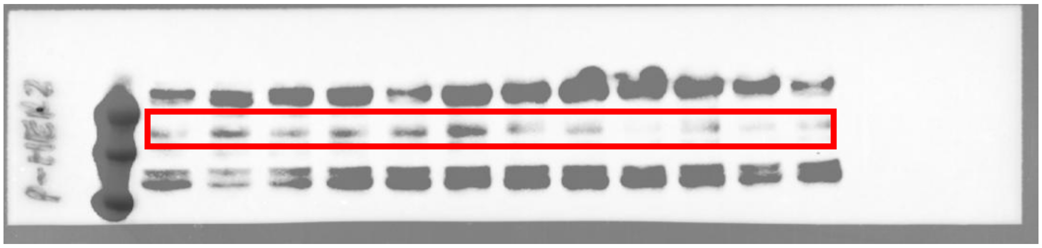

HER2

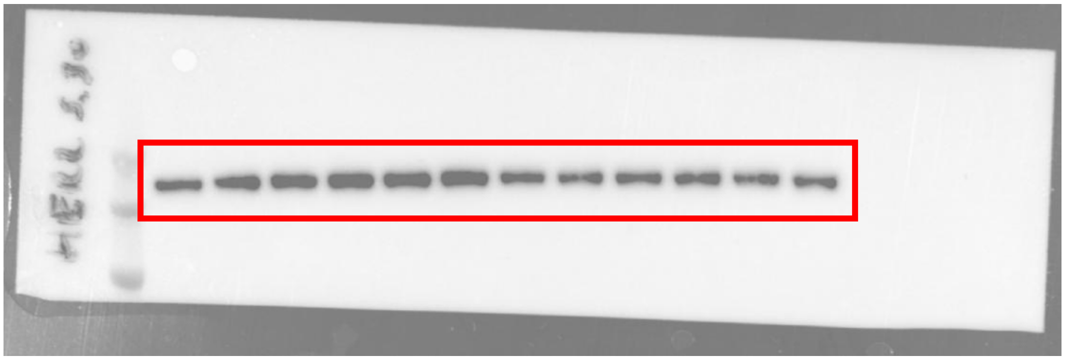

P-HER3

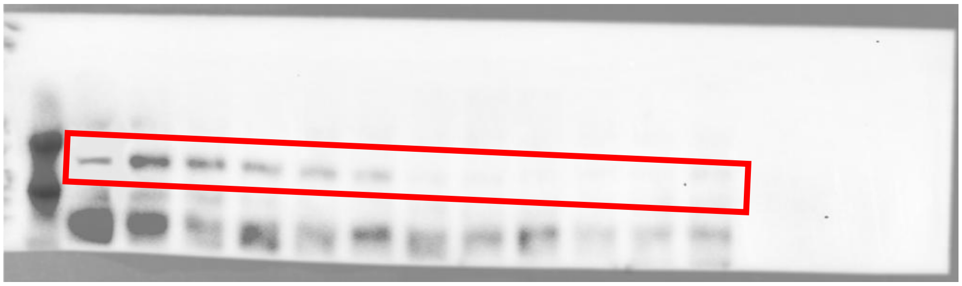

HER3

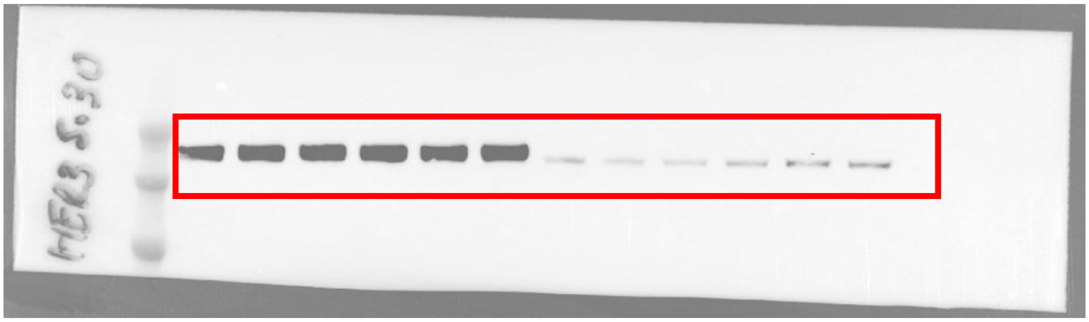

Full and Unedited Blots for Figure 5A Continued

Lanes: EGF ctrl, TWT 0, 5, 10, 15, 30, 60, KD 0, 5, 10, 15, 30, 60
P-EGFR Y845

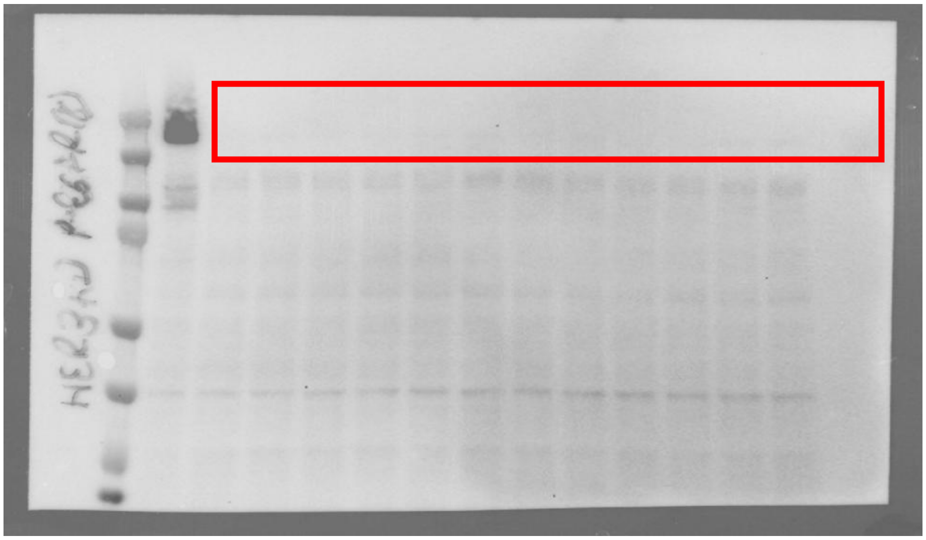

Lanes: EGF ctrl, TWT 0, 5, 10, 15, 30, 60, KD 0, 5, 10, 15, 30, 60
P-EGFR Y1173

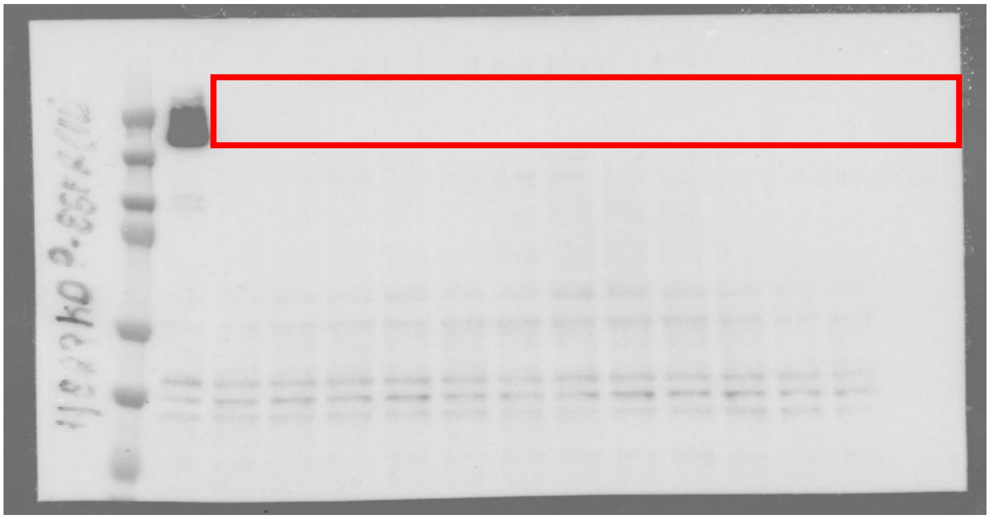

Full and Unedited Blots for Figure 5A Continued

Lanes: EGF ctrl, TWT 0, 5, 10, 15, 30, 60, KD 0, 5, 10, 15, 30, 60

EGFR

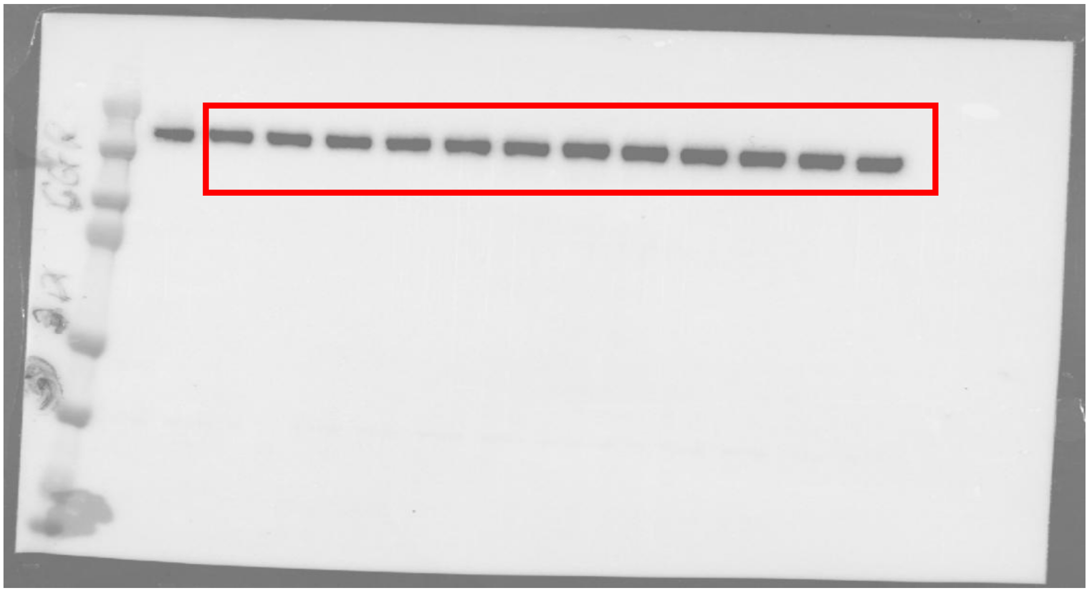

P-AKT

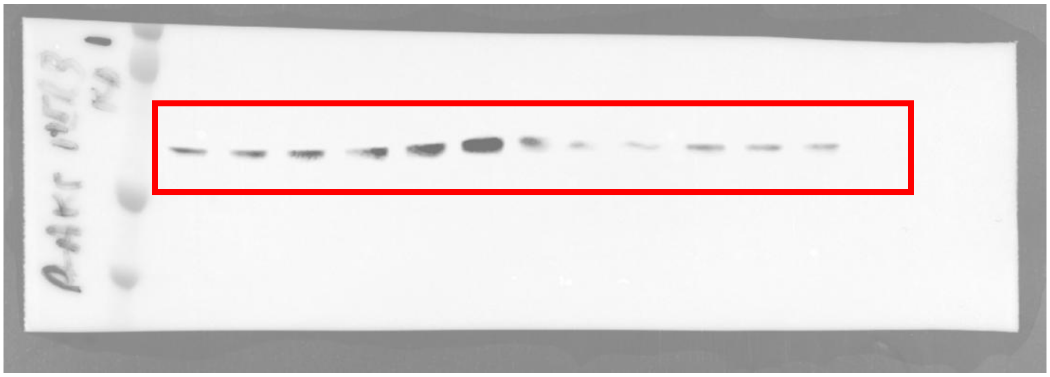

AKT

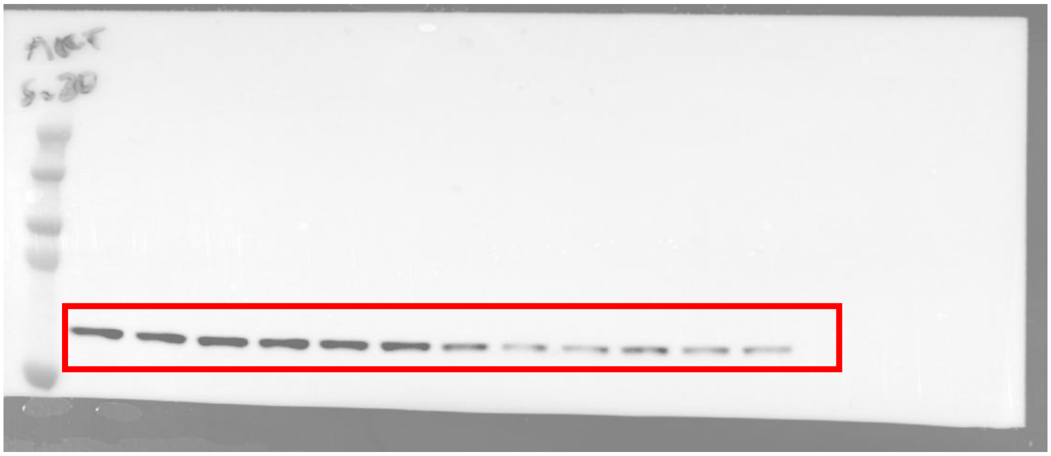

Full and Unedited Blots for Figure 5A Continued

P-ERK

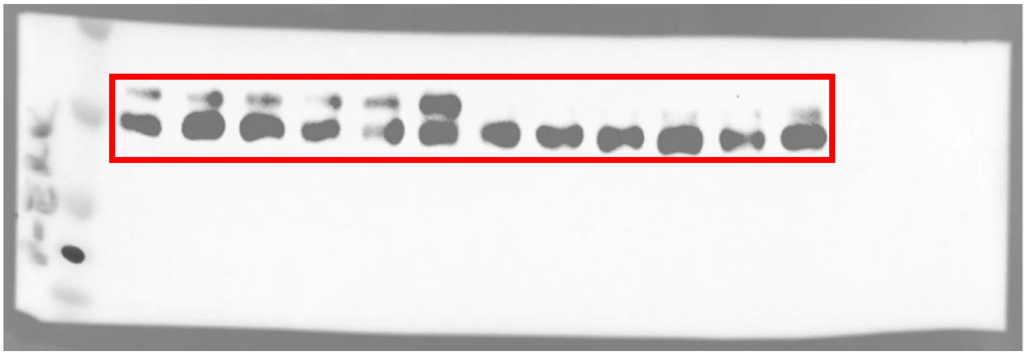

ERK

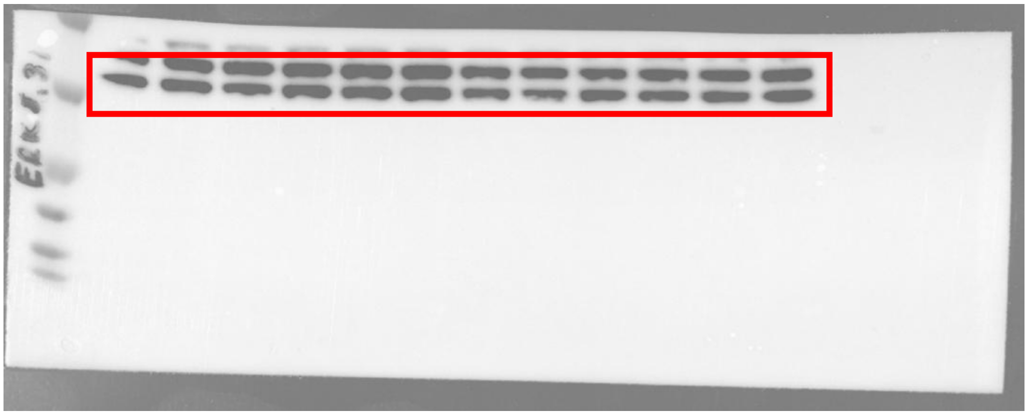

P-PLC

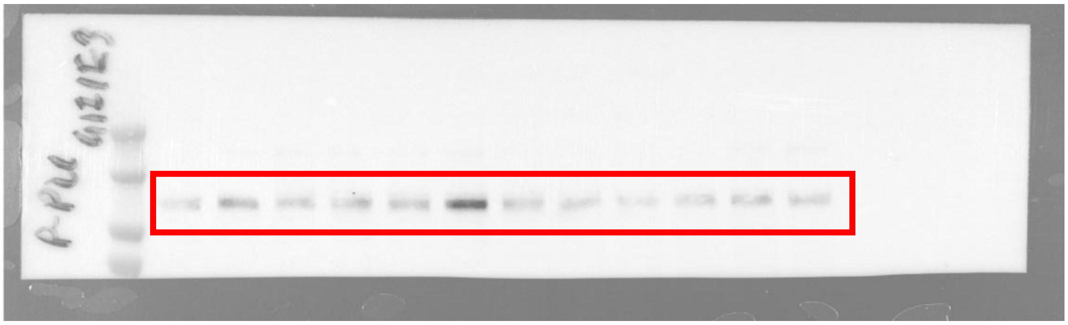

PLC

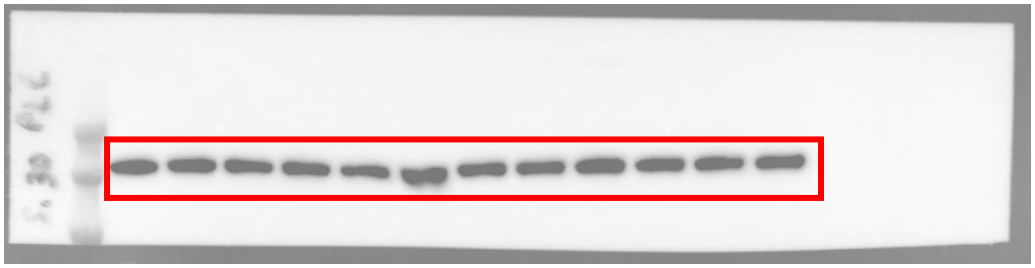

Full and Unedited Blots for Figure 5A Continued

P-CAMKII

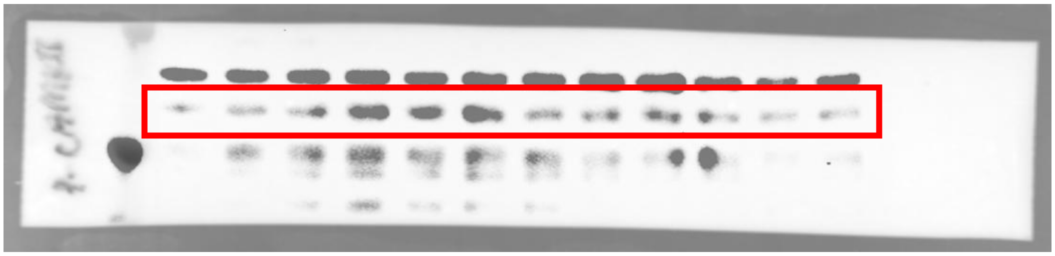

CAMKII

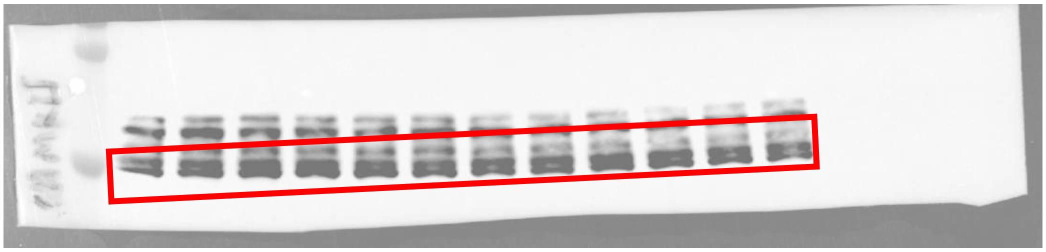

GAPDH

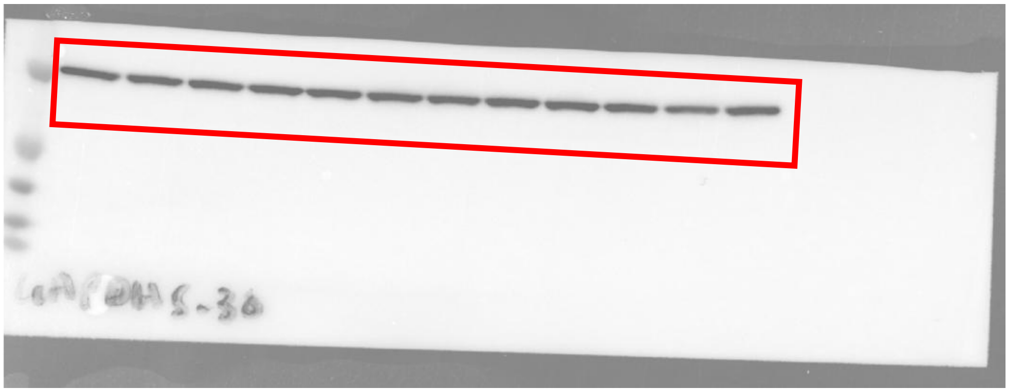

Full and Unedited Blots for Figure 5C

HER2 TWT C9, HER2 KO C12
Lanes: TWT 0, 5, 10, 15, 30, 60, KD 0, 5, 10, 15, 30, 60 unless noted
P-HER2

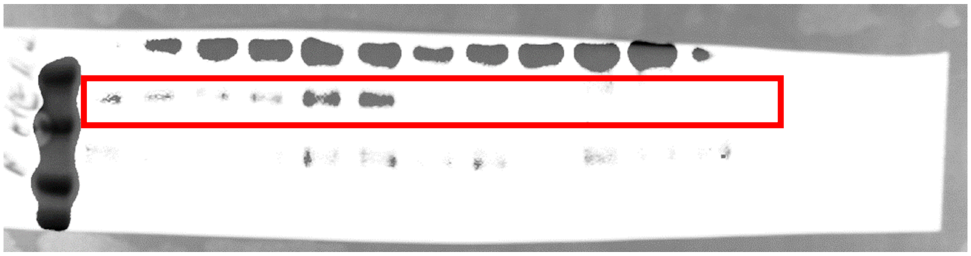

HER2

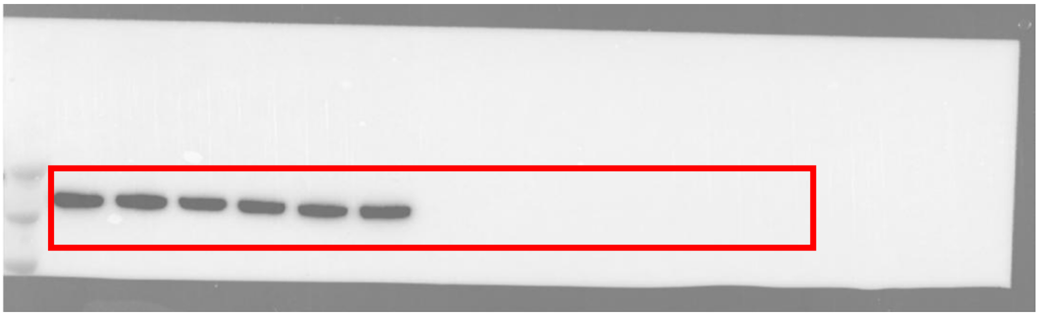

P-HER3

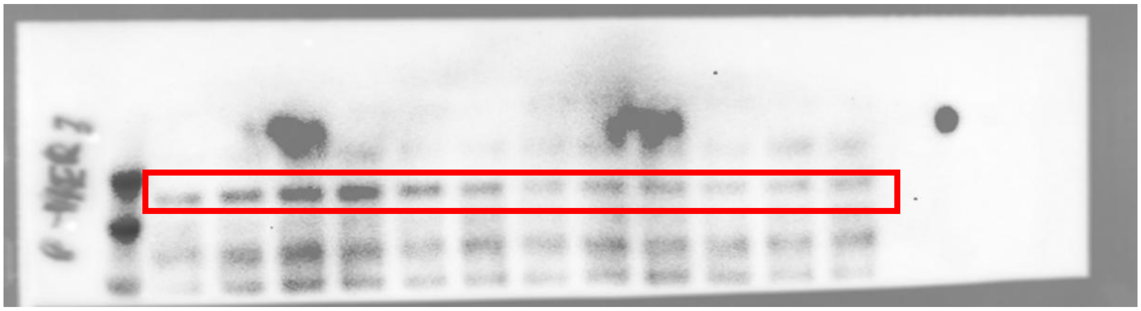

HER3

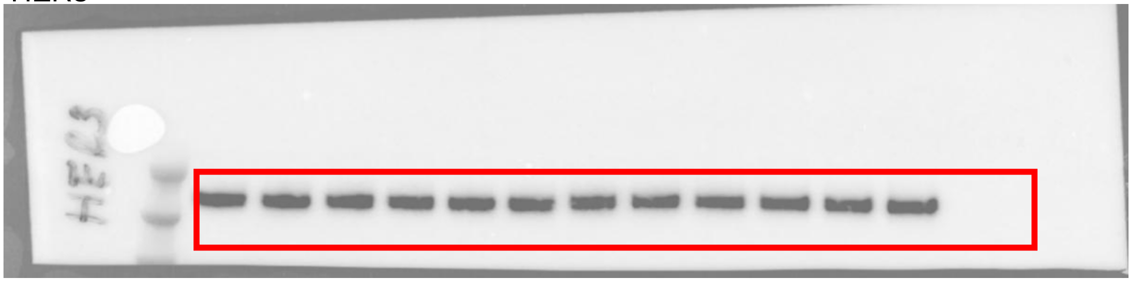

Full and Unedited Blots for Figure 5C Continued

Lanes: EGF ctrl, TWT 0, 5, 10, 15, 30, 60, KO 0, 5, 10, 15, 30, 60
P-EGFR Y845

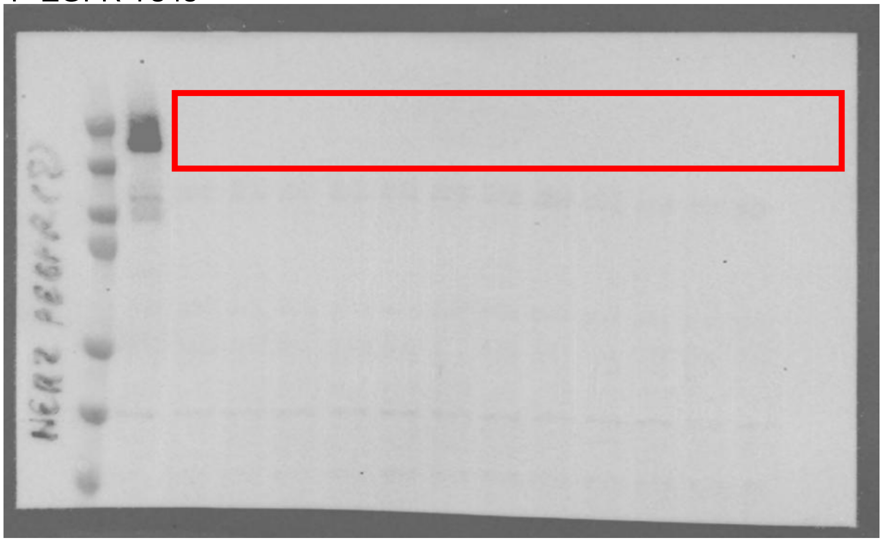

Lanes: EGF ctrl, TWT 0, 5, 10, 15, 30, 60, KO 0, 5, 10, 15, 30, 60
P-EGFR Y1173

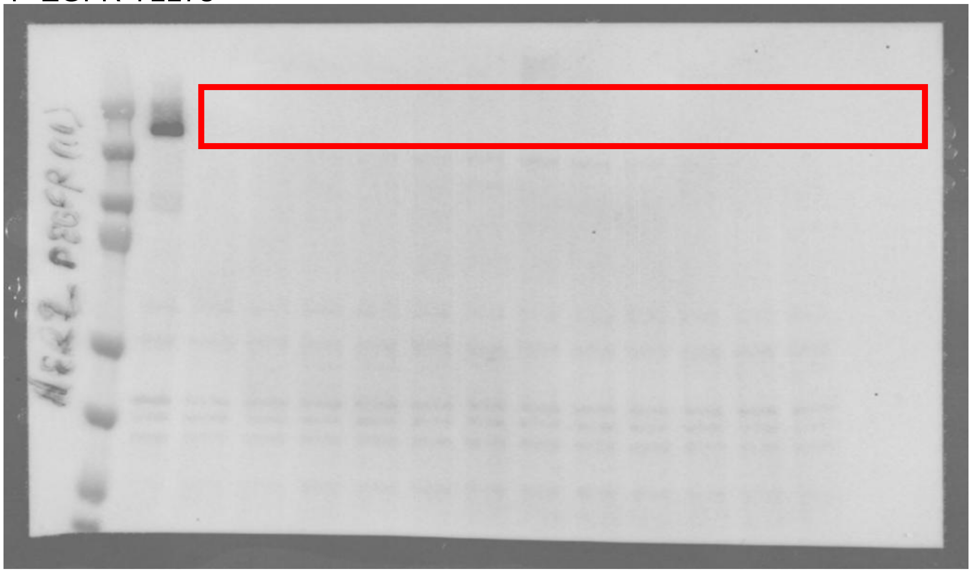

Full and Unedited Blots for Figure 5C Continued

Lanes: EGF ctrl, TWT 0, 5, 10, 15, 30, 60, KO 0, 5, 10, 15, 30, 60

EGFR

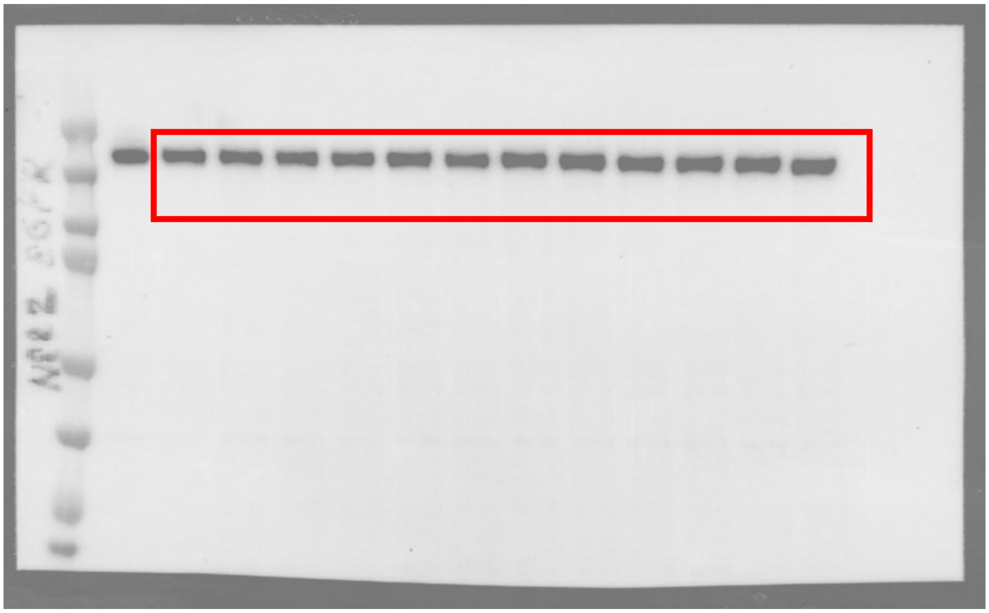

P-AKT

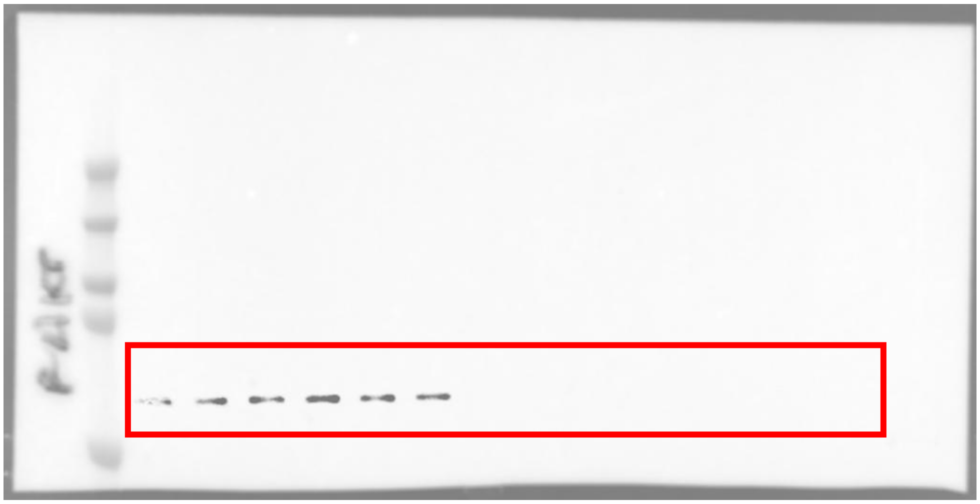

AKT

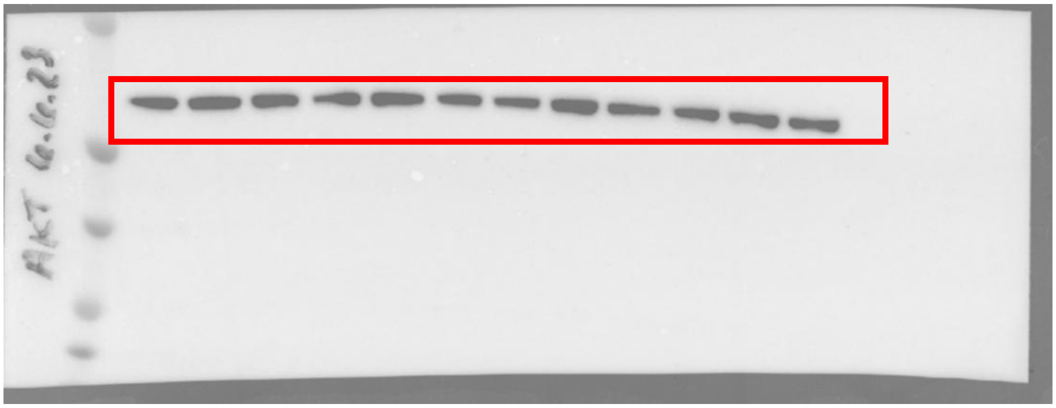

Full and Unedited Blots for Figure 5C Continued

P-ERK

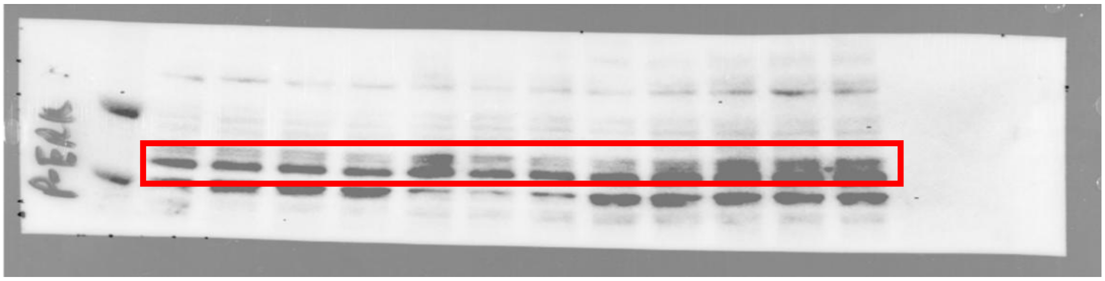

ERK

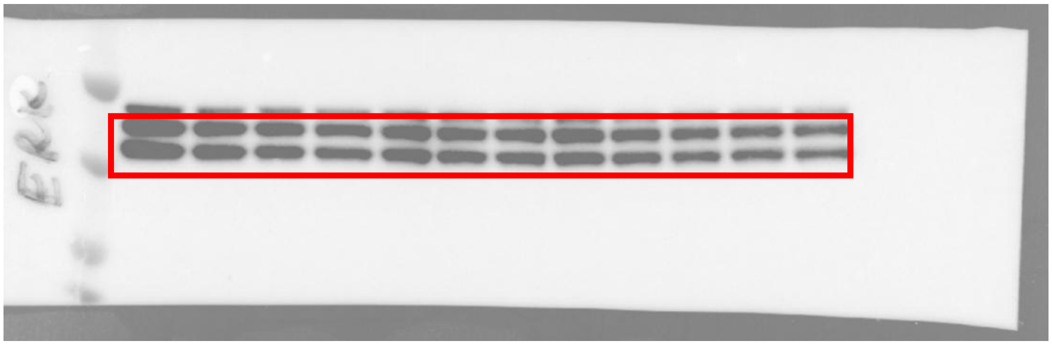

P-PLC

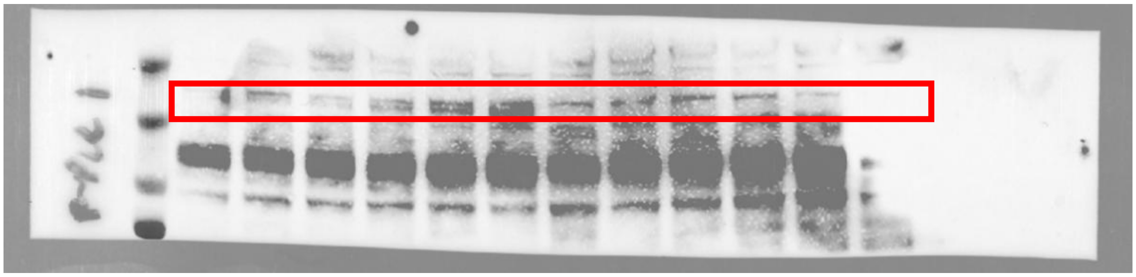

PLC

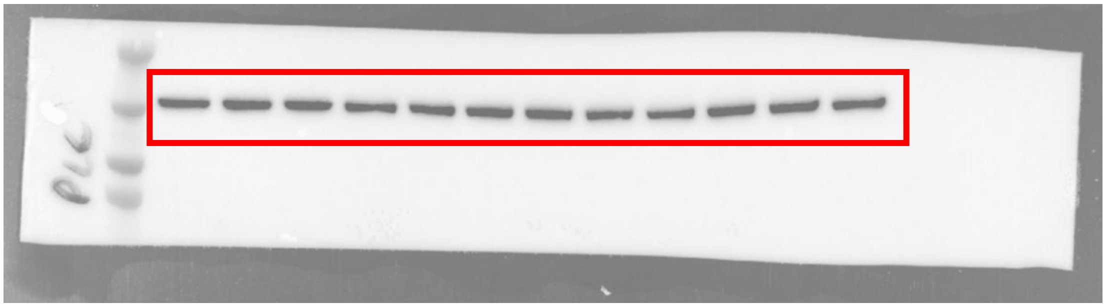

Full and Unedited Blots for Figure 5C Continued

P-CAMKII

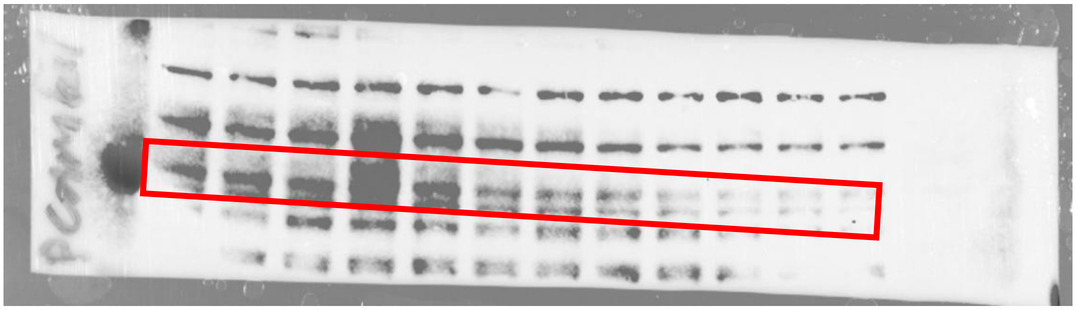

CAMKII

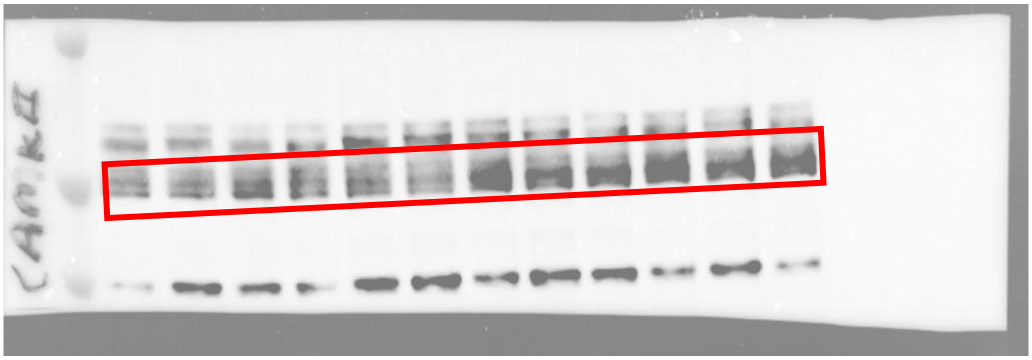

GAPDH

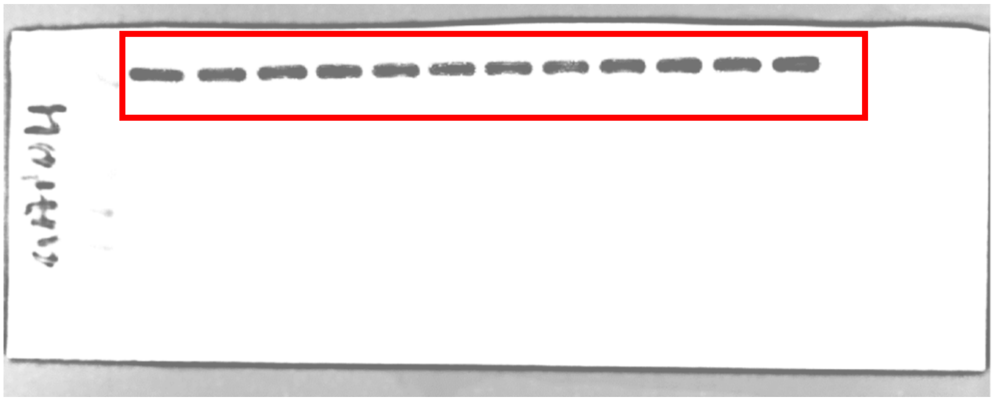

Full and Unedited Blots for Figure 6A

Lanes: ASPN 0, 5, 10, 15, 30, 60, ASPN+Tuc 0, 5, 10, 15, 30, 60 unless noted

P-HER2

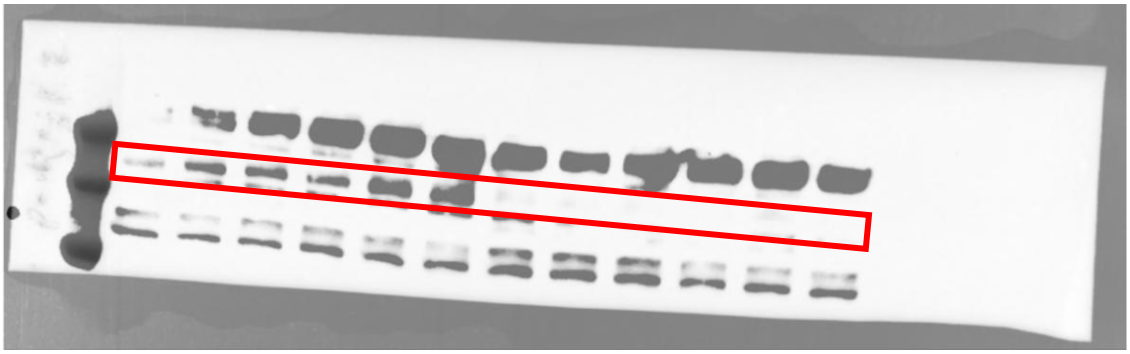

HER2

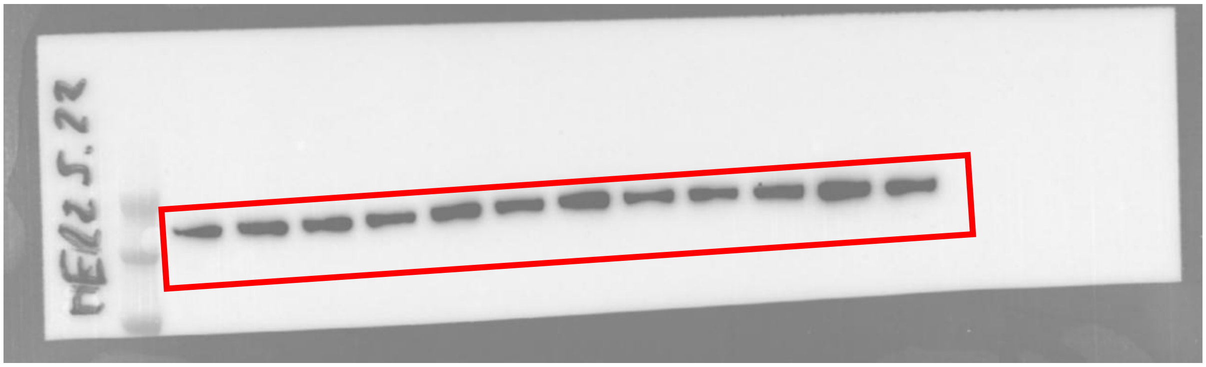

P-HER3

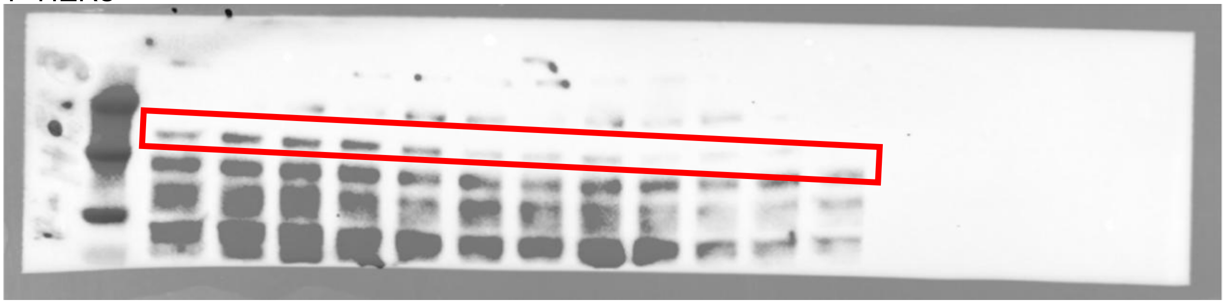

HER3

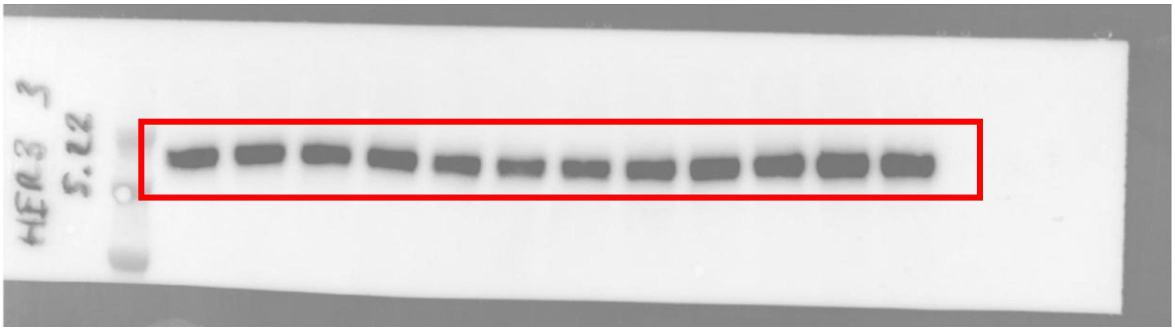

Full and Unedited Blots for Figure 6A Continued

Lanes: EGF ctrl, ASPN 0, 5, 10, 15, 30, 60, ASPN+Tuc 0, 5, 10, 15, 30, 60

P-EGFR Y845

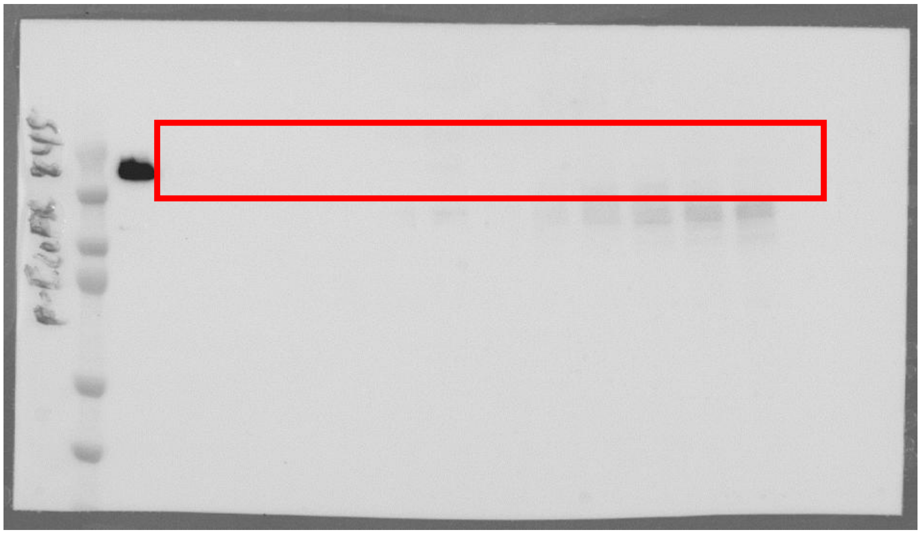

Lanes: EGF ctrl, ASPN 0, 5, 10, 15, 30, 60, ASPN+Tuc 0, 5, 10, 15, 30, 60

P-EGFR Y1173

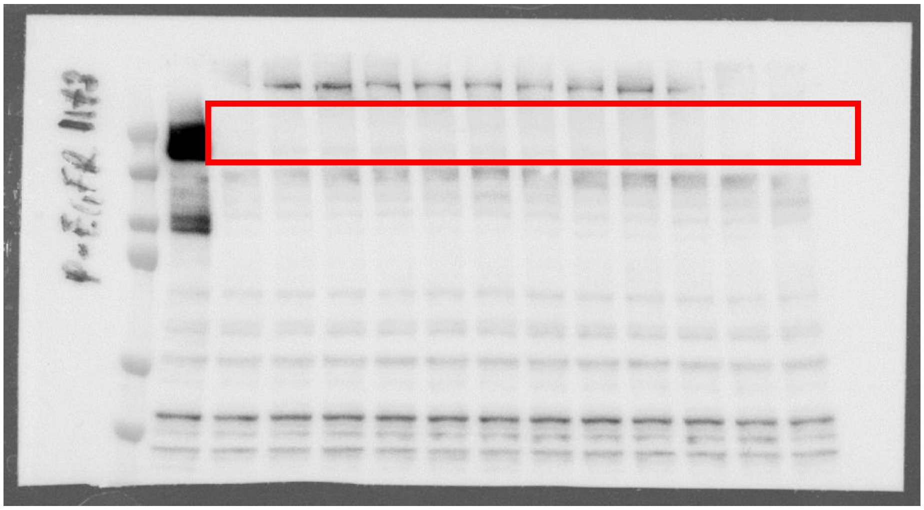

Lanes: EGF ctrl, ASPN 0, 5, 10, 15, 30, 60, ASPN+Tuc 0, 5, 10, 15, 30, 60

EGFR

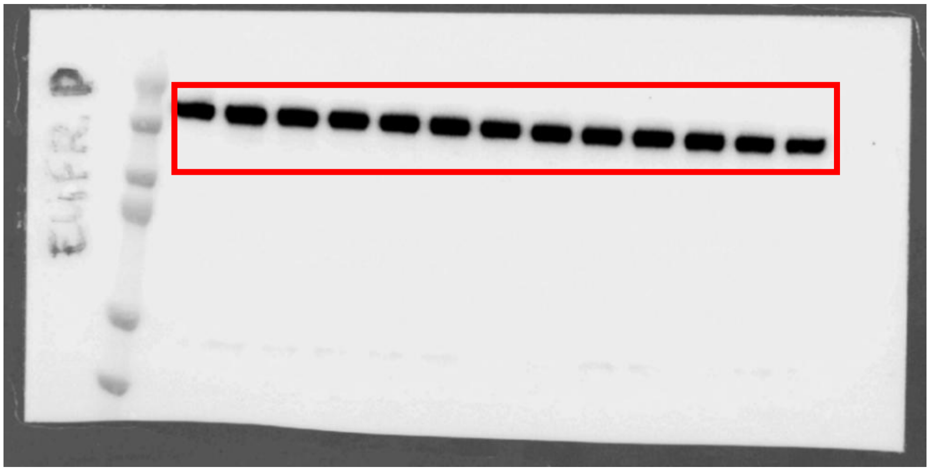

Full and Unedited Blots for Figure 6A Continued

P-AKT

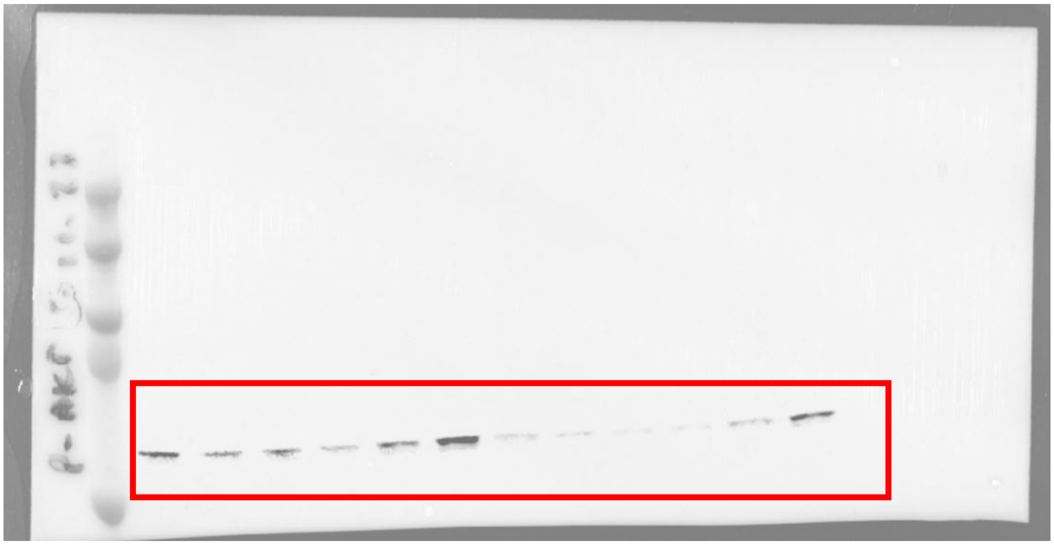

AKT

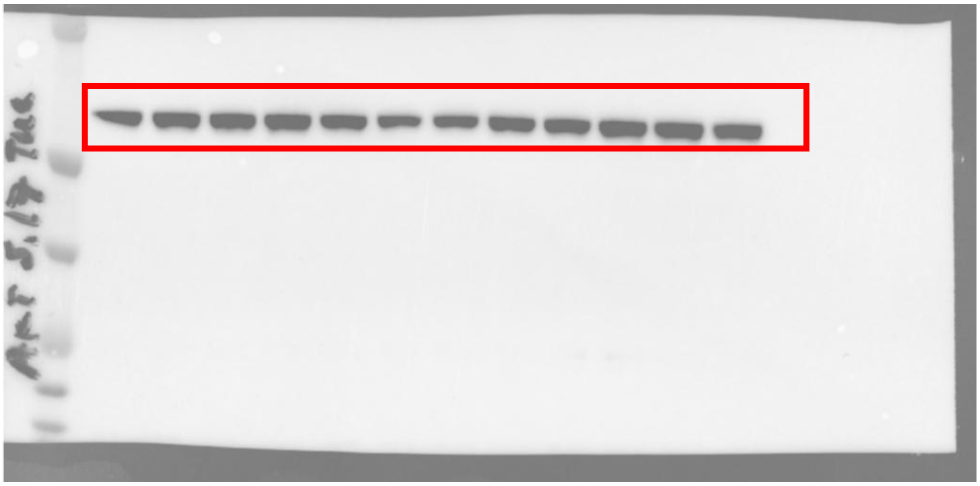

P-ERK

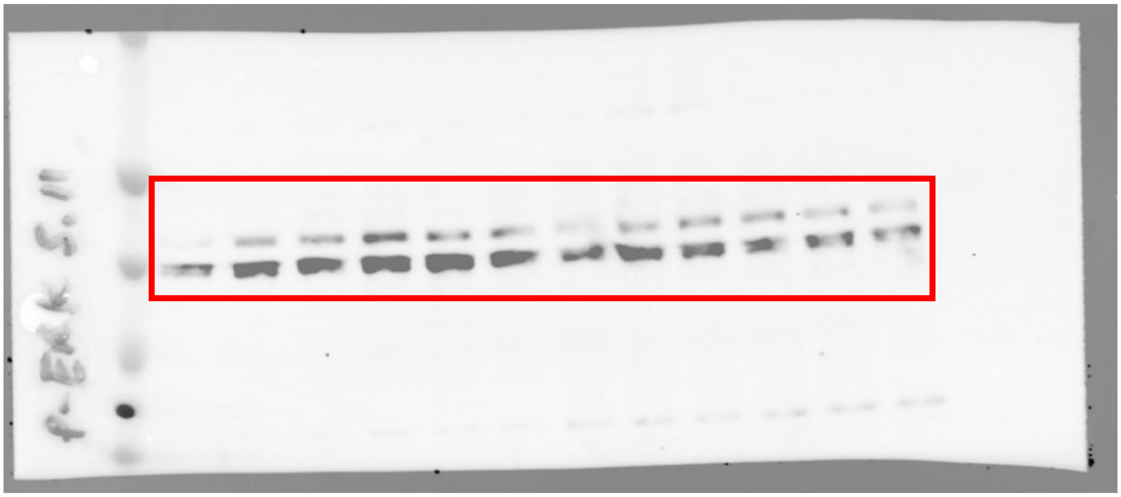

Full and Unedited Blots for Figure 6A Continued

ERK

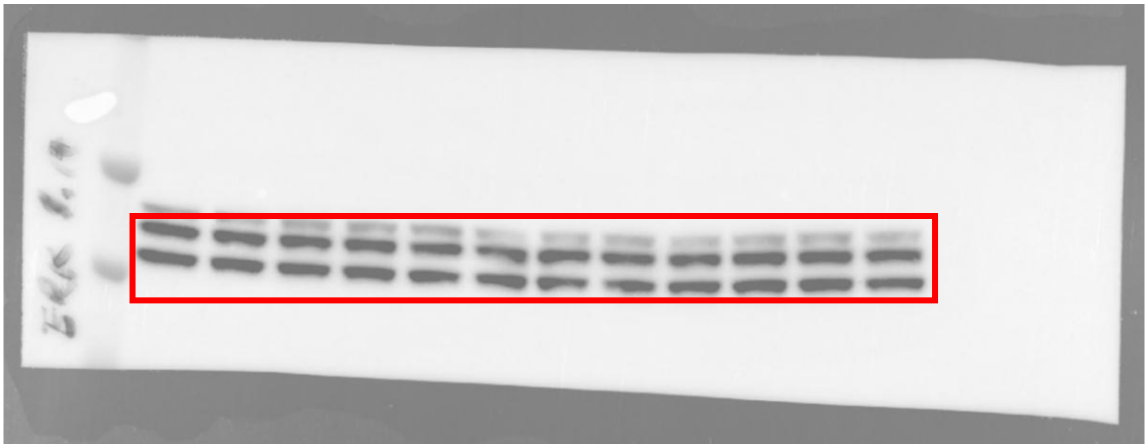

P-PLC

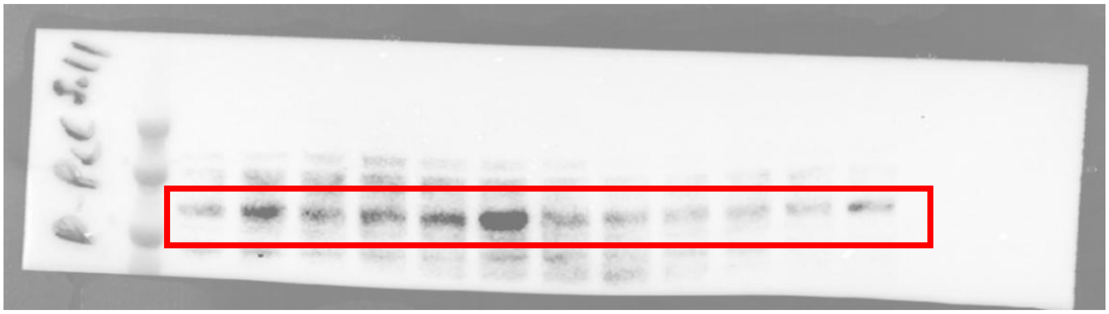

PLC

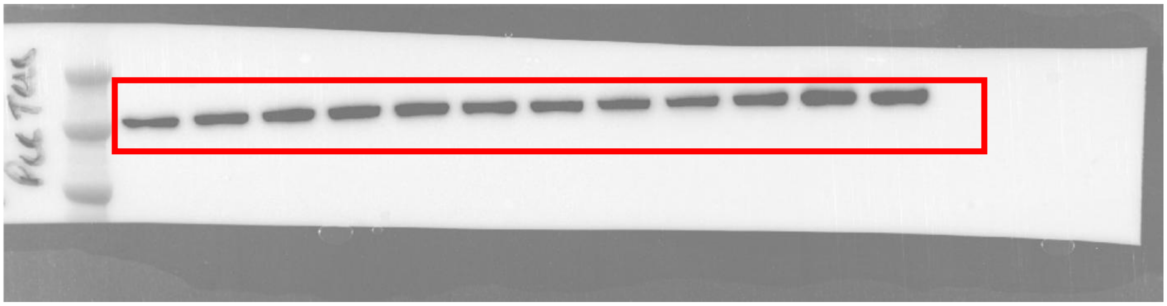

Full and Unedited Blots for Figure 6A Continued

P-CAMKII

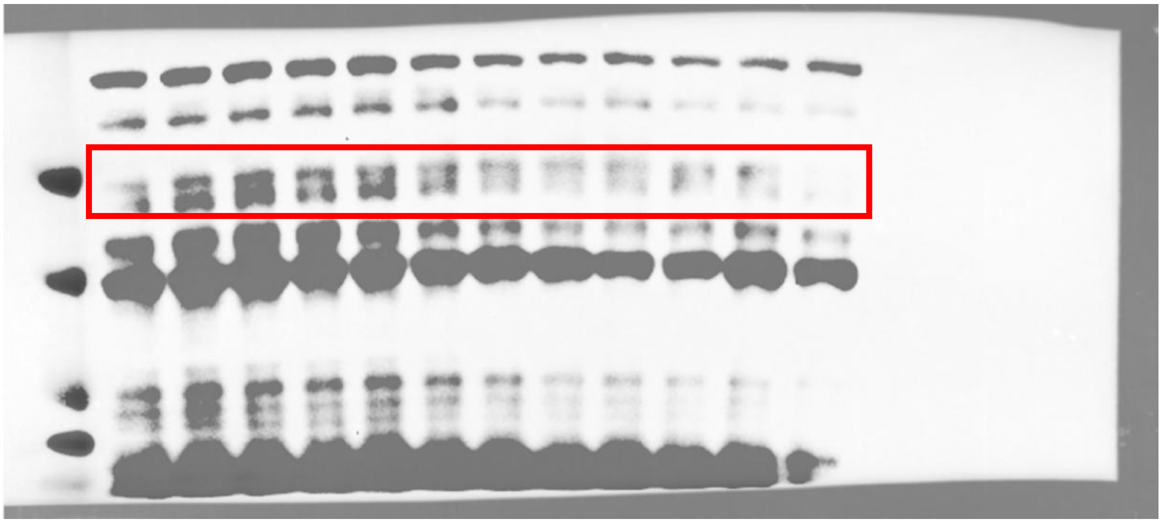

CAMKII

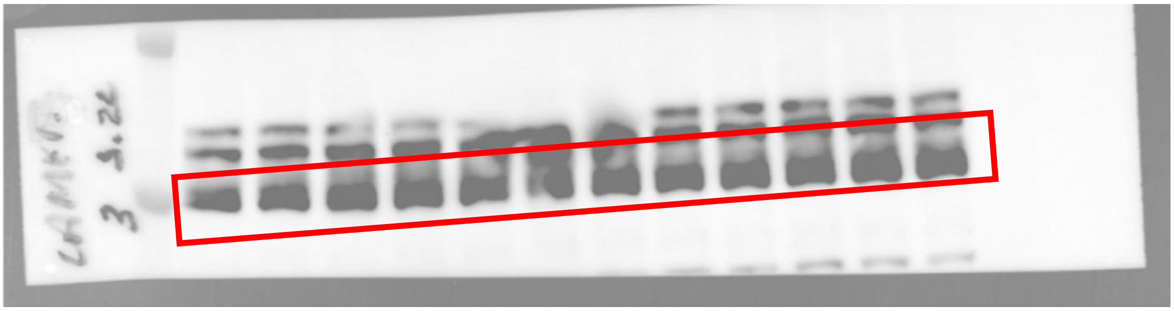

GAPDH

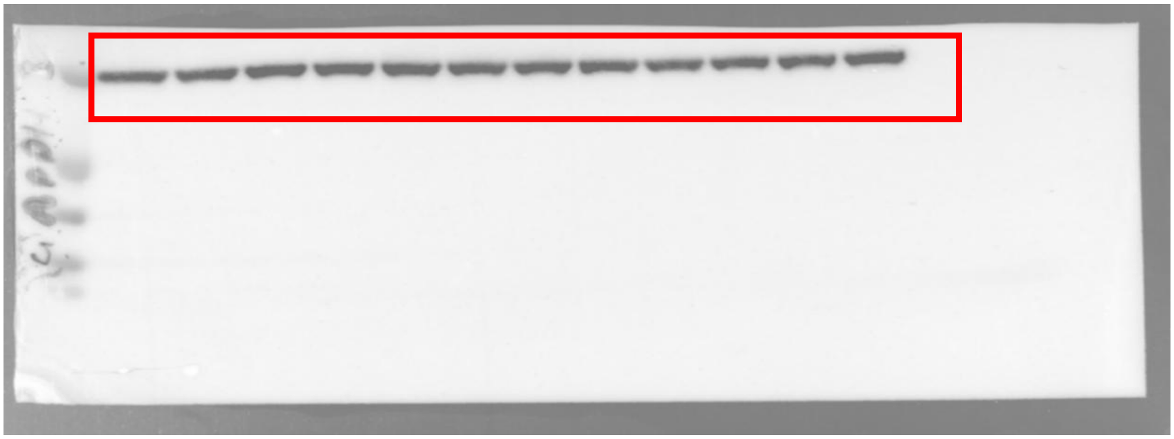

Full and Unedited Blots for Figure 6C

Lanes: ASPN 0, 5, 10, 15, 30, 60, ASPN+Tuc 0, 5, 10, 15, 30, 60 unless noted

P-HER2

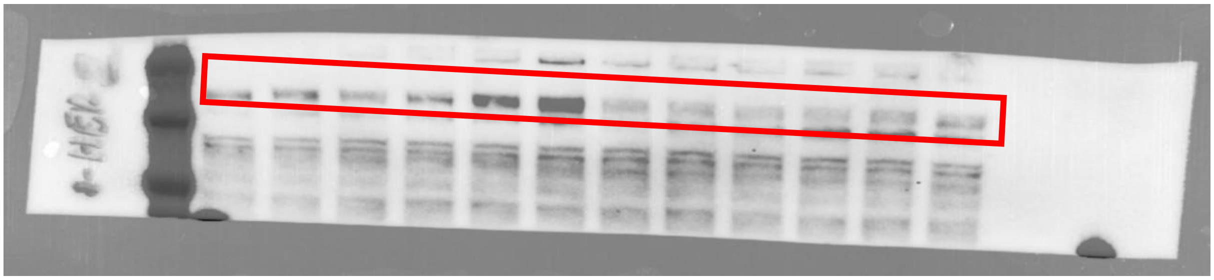

HER2

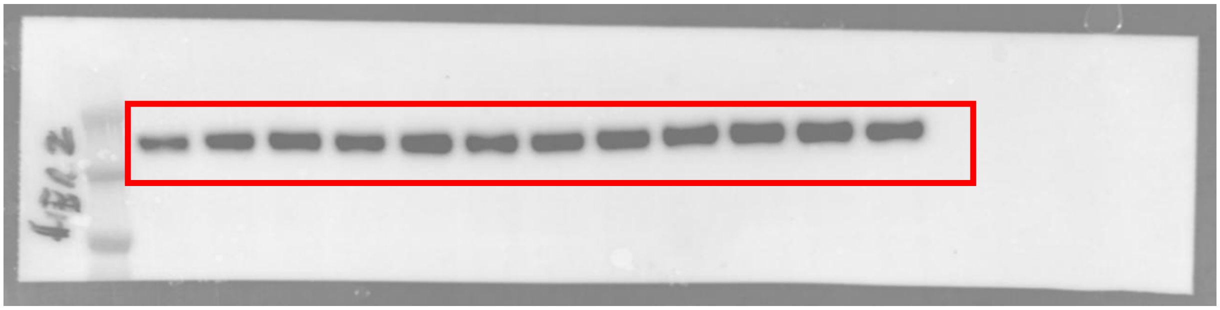

P-HER3

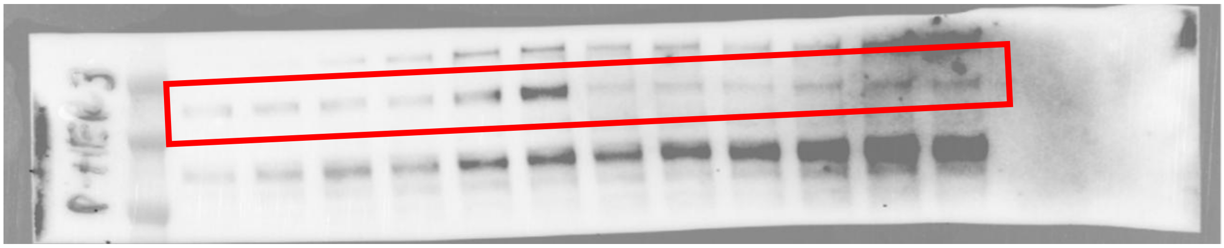

HER3

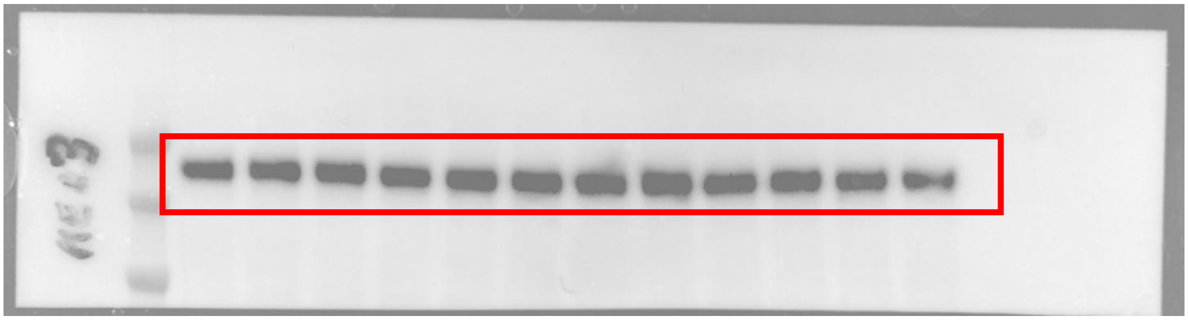

Full and Unedited Blots for Figure 6C Continued

P-EGFR Y845

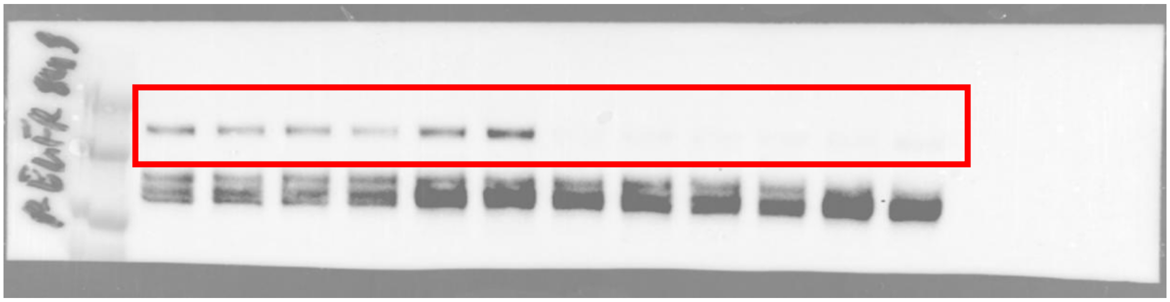

P-EGFR Y1173

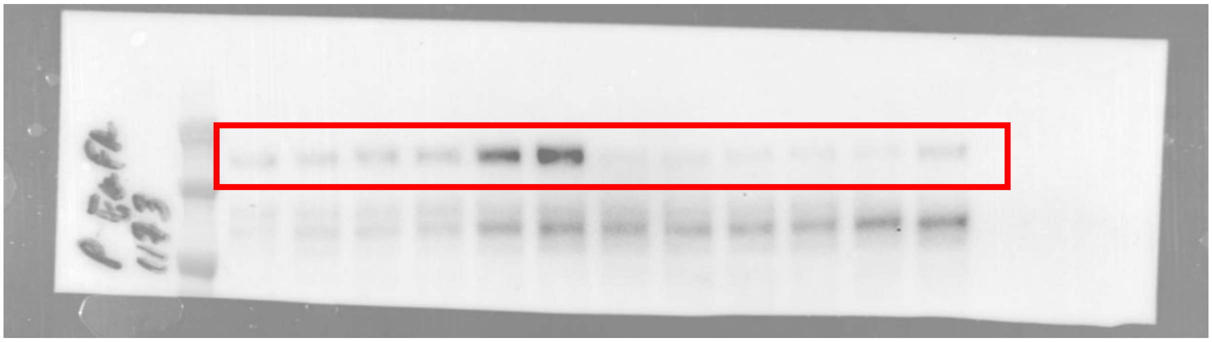

EGFR

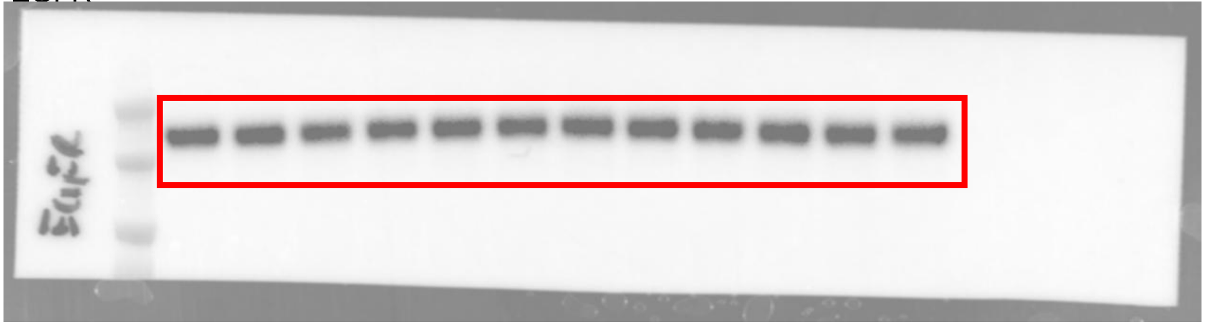

P-AKT

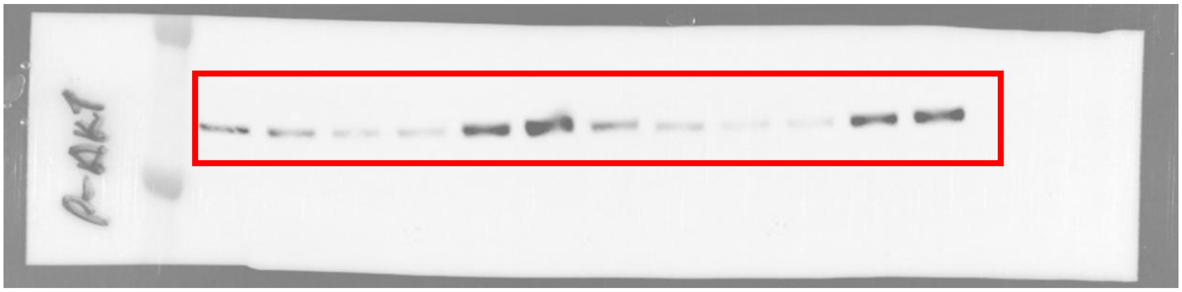

Full and Unedited Blots for Figure 6C Continued

AKT

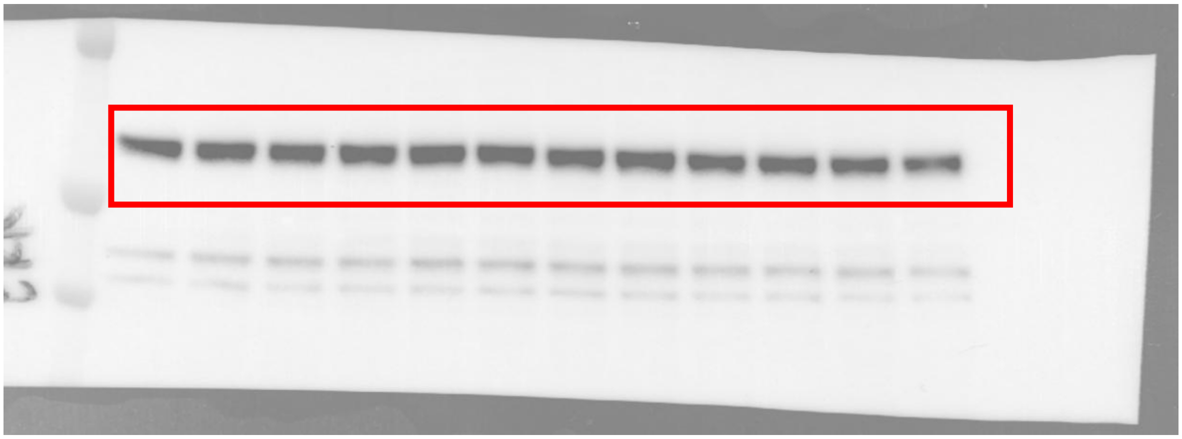

P-ERK

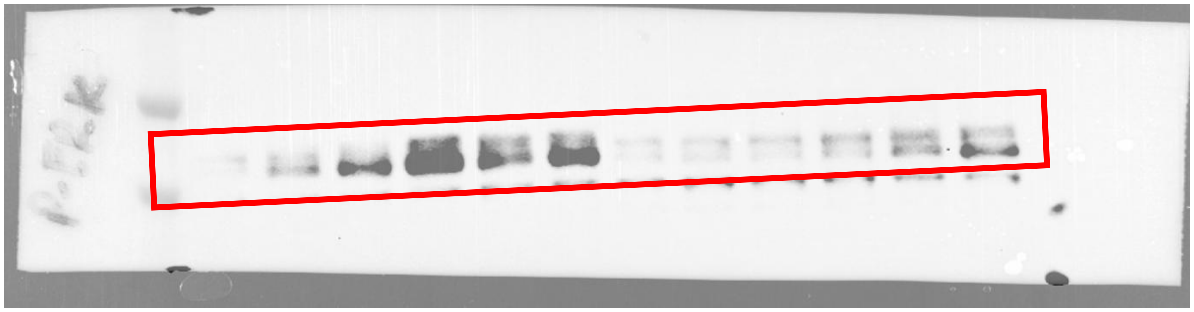

ERK

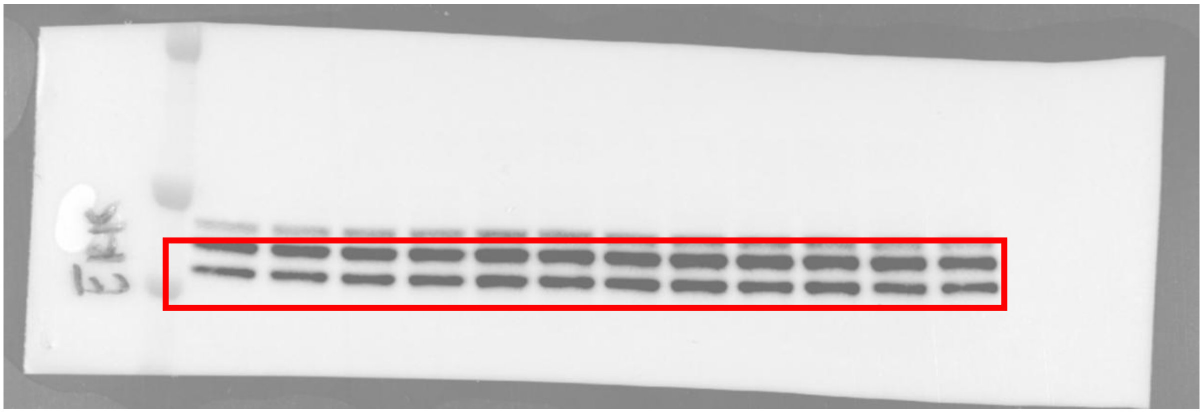

P-PLC

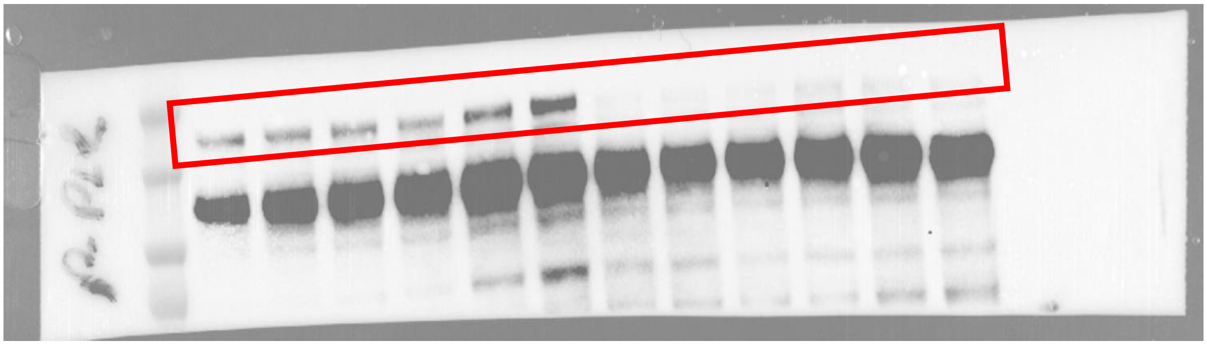

Full and Unedited Blots for Figure 6C Continued

PLC

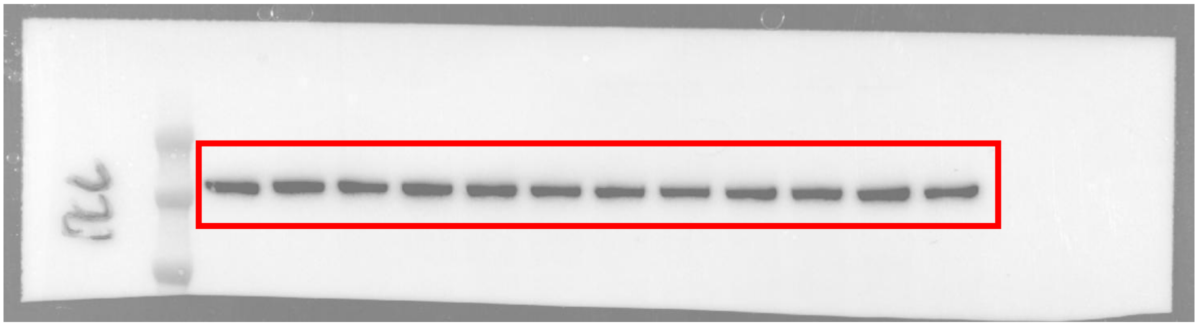

P-CAMKII

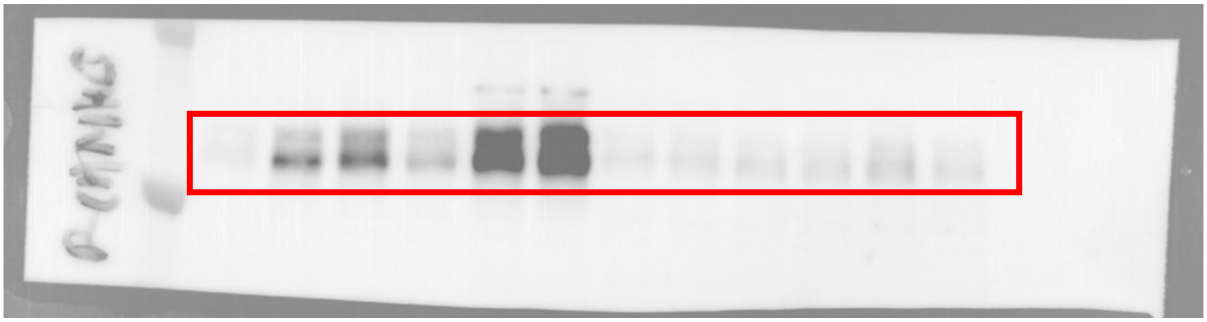

CAMKII

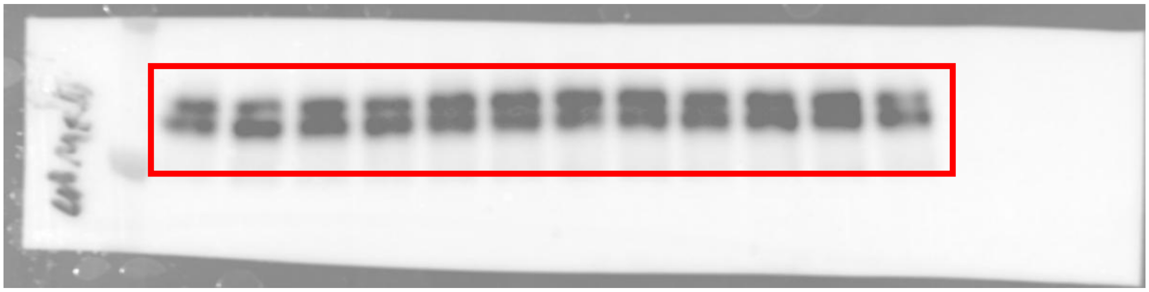

GAPDH

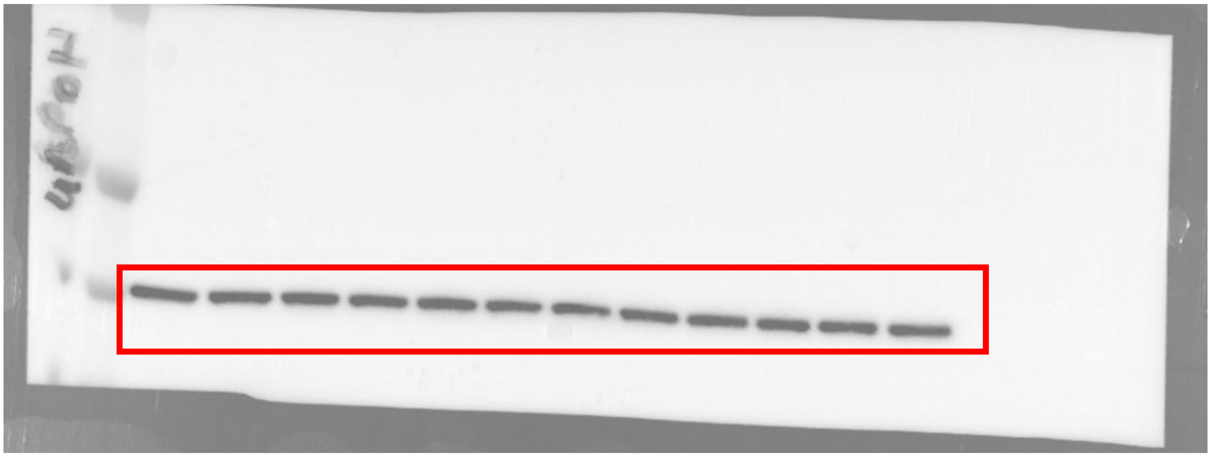

Full and Unedited Blots from Figure 9B

HER2

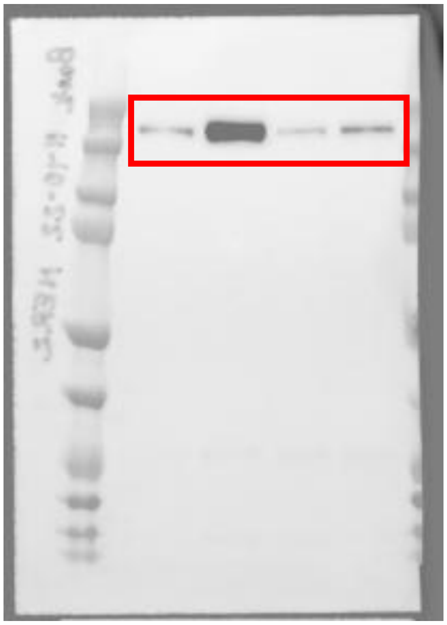

HER3

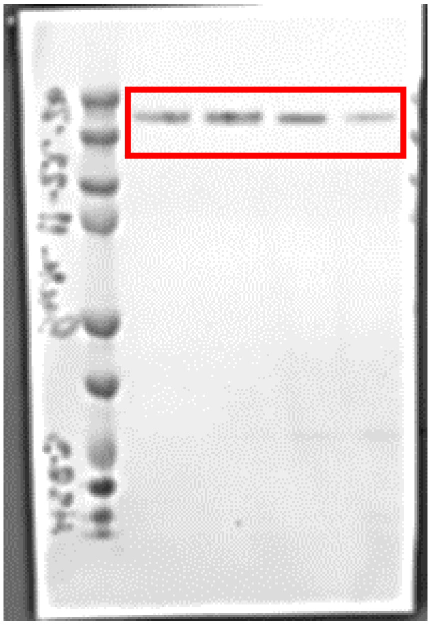

HER4

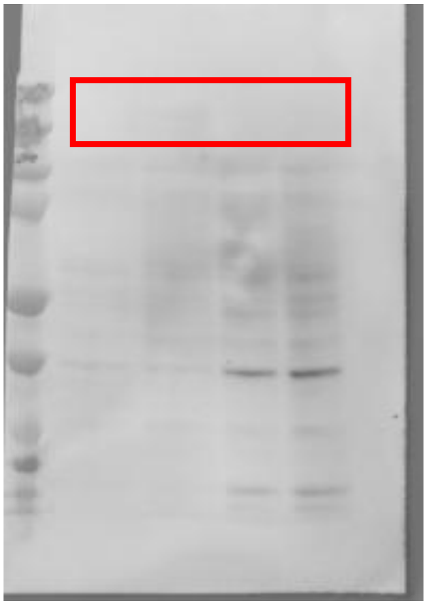

EGFR

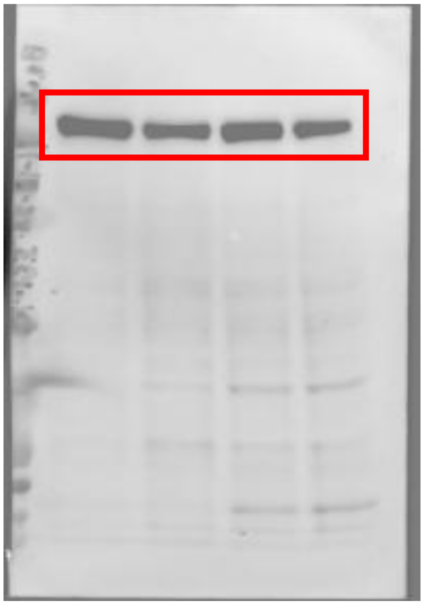

Full and Unedited Blots from Figure 9B Continued

AR

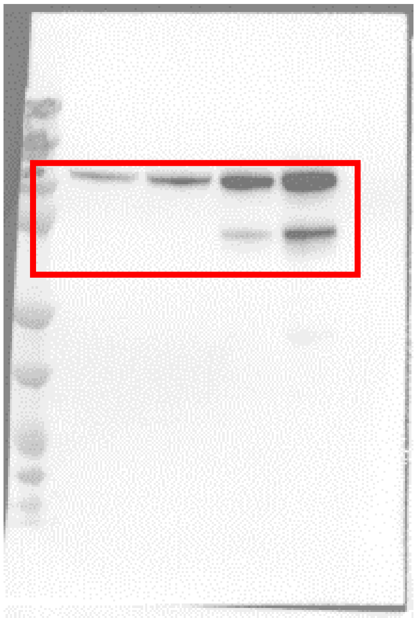

GAPDH

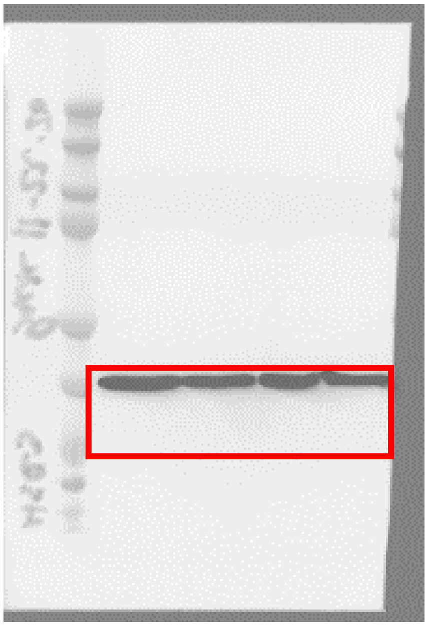

Full and Unedited Blots for Supplemental Figure 2A

VCaP + ASPN

Lanes: 0, 2, 5, 10, 15, 30, 60, 120 min ASPN unless noted

P-HER2

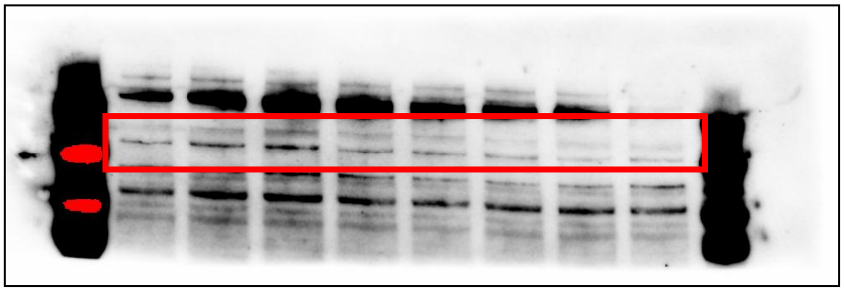

HER2

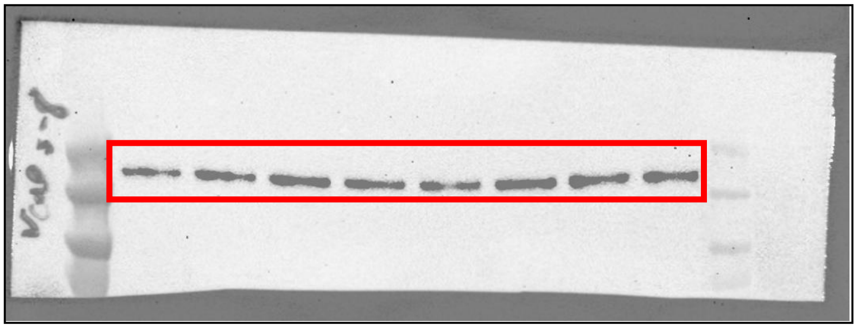

P-HER3

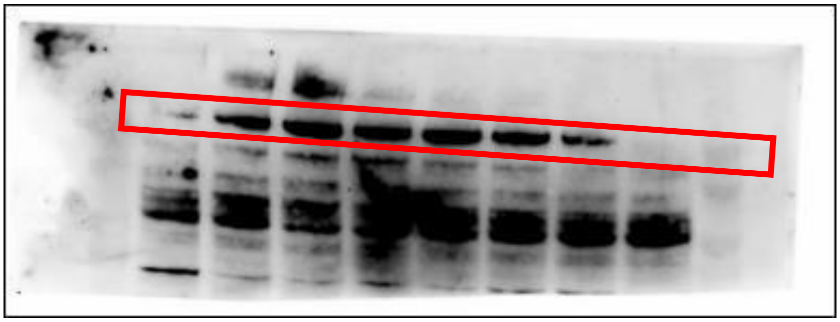

HER3

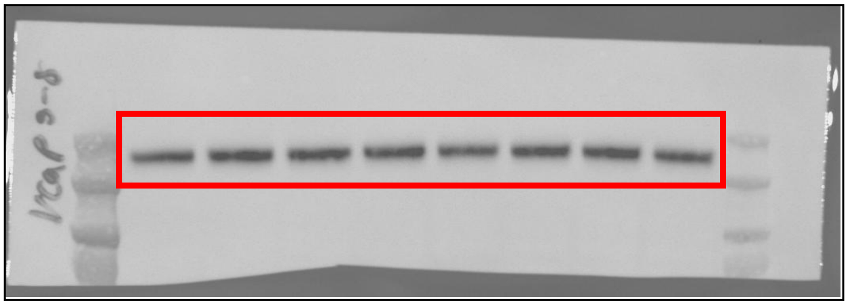

Full and Unedited Blots for Supplemental Figure 2A Continued

P-AKT

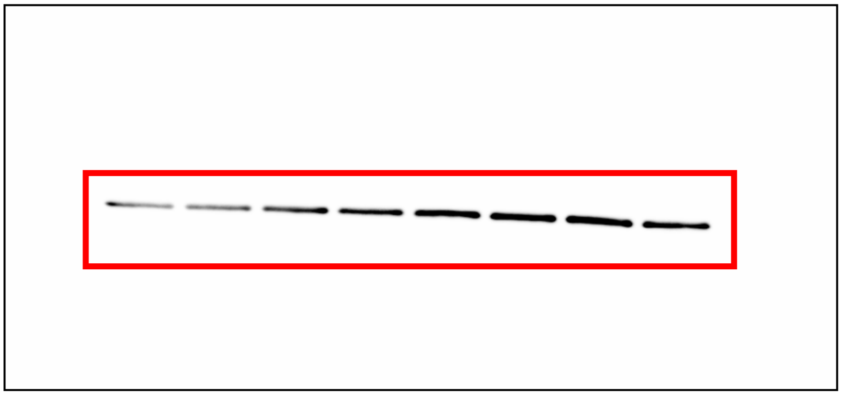

AKT

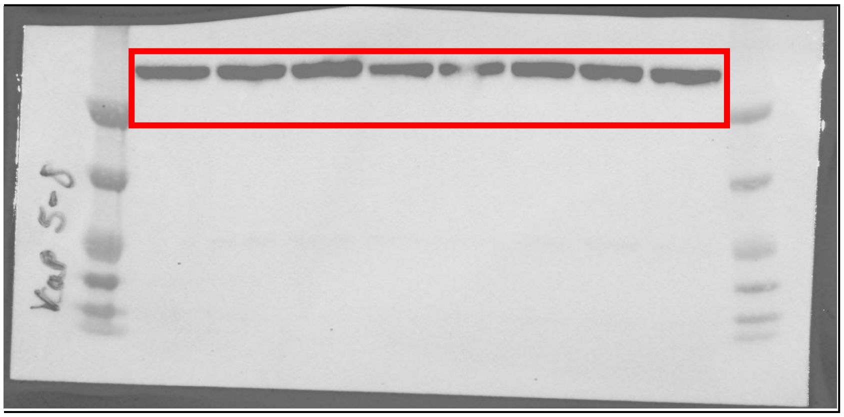

P-ERK

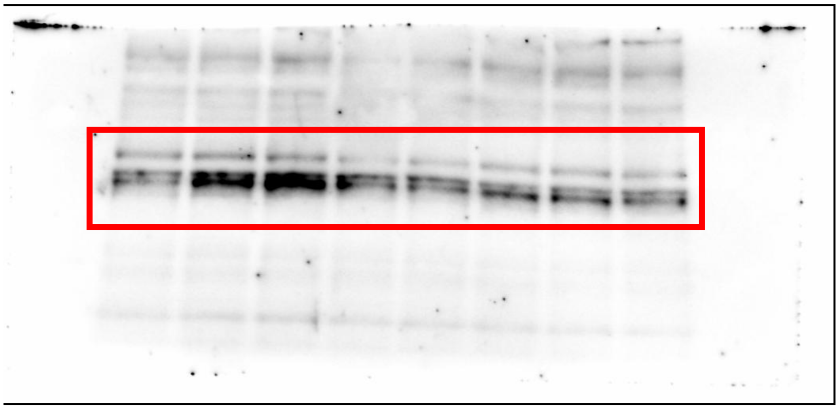

Full and Unedited Blots for Supplemental Figure 2A Continued

ERK

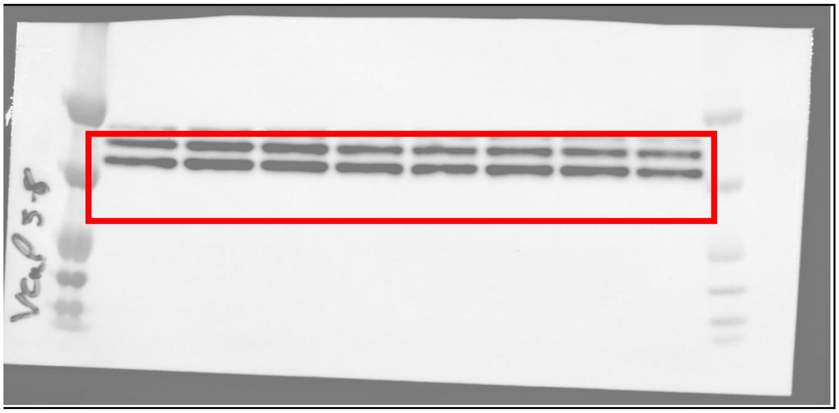

P-PLCg

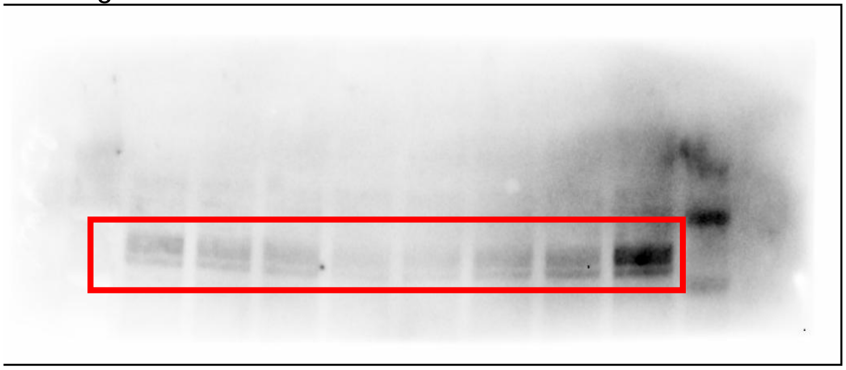

PLCg

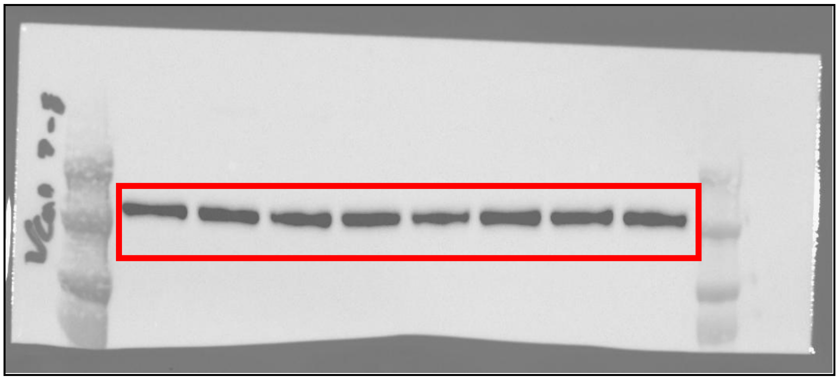

Full and Unedited Blots for Supplemental Figure 2A Continued

P-CAMKII

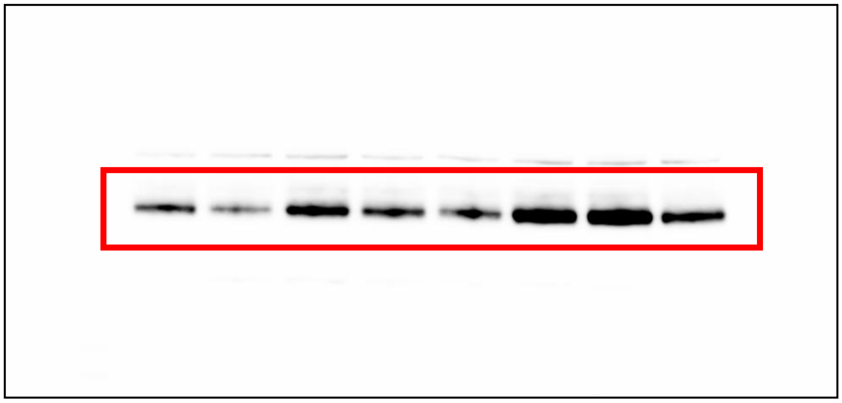

CAMKII

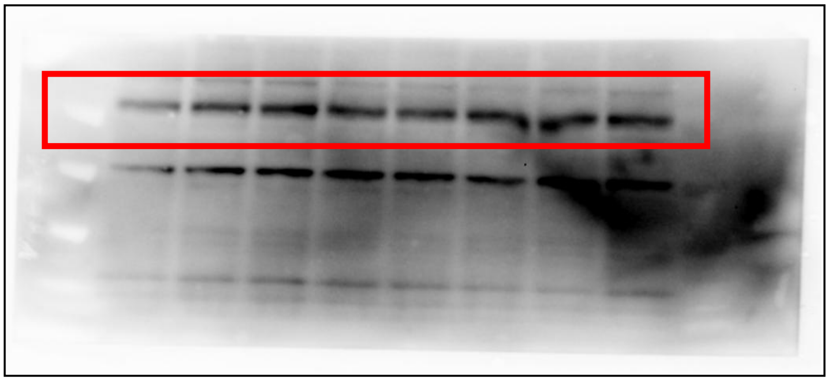

GAPDH

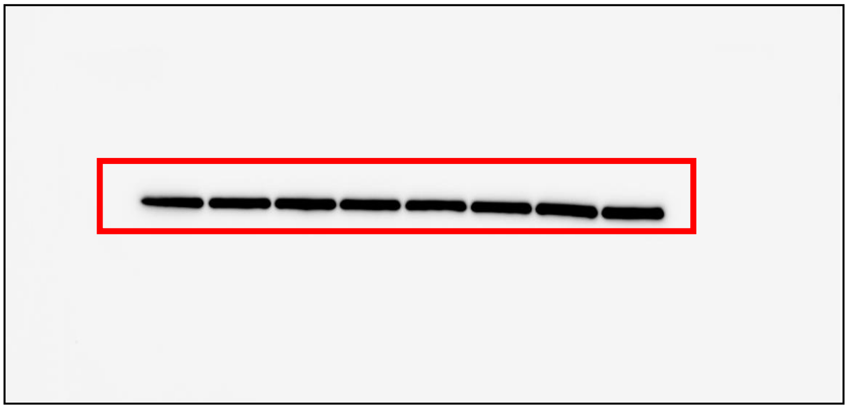

Full and Unedited Blots for Supplemental Figure 2B

DU145 + ASPN (100ng/mL)

Lanes: 0, 2, 5, 10, 15, 30, 60, 120 min ASPN unless noted

P-HER2

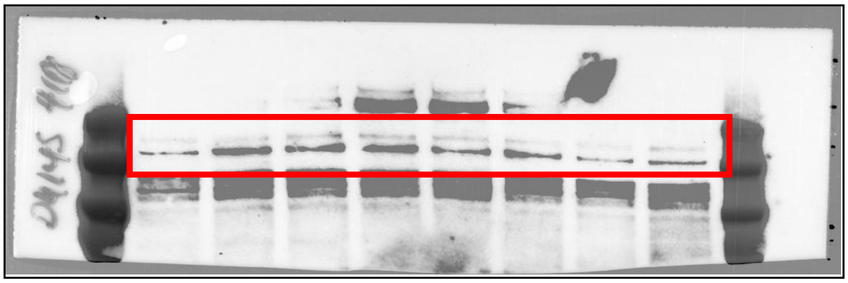

HER2

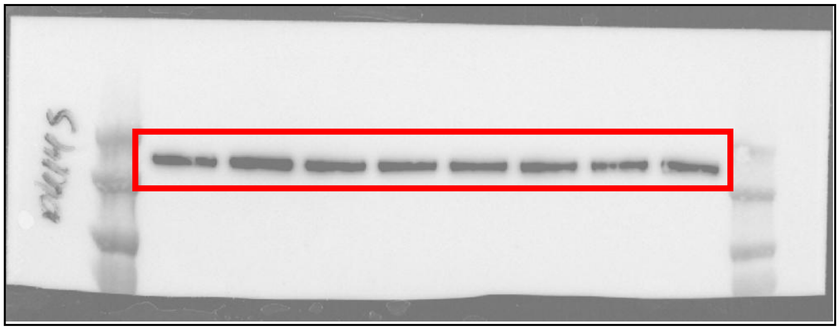

P-HER3

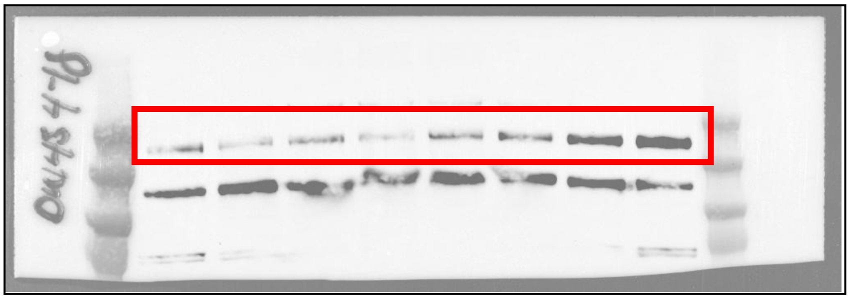

HER3

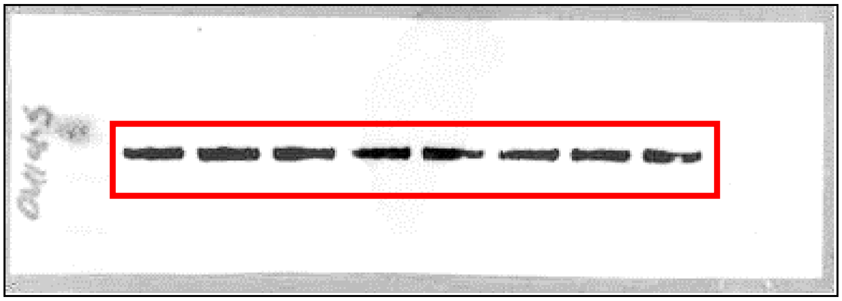

Full and Unedited Blots for Supplemental Figure 2B Continued

P-AKT

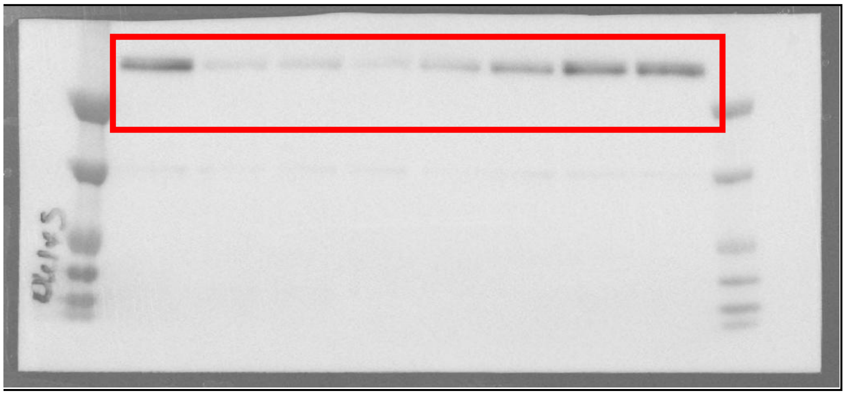

AKT

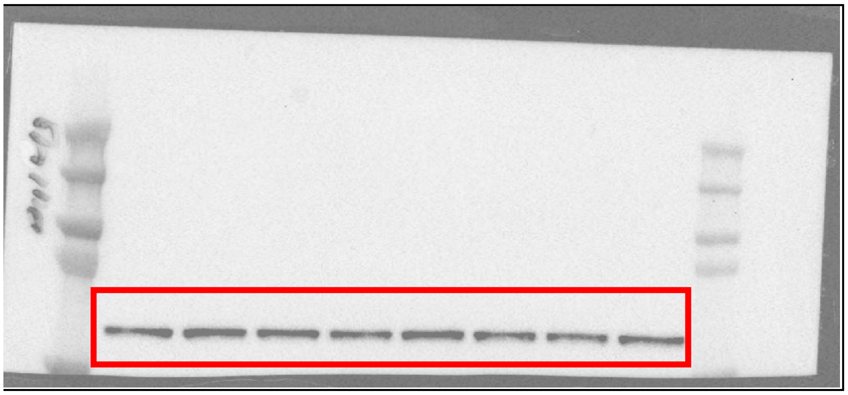

P-ERK

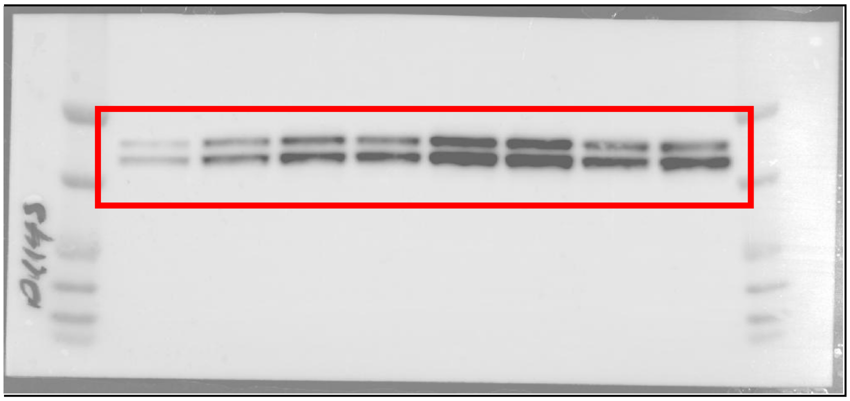

Full and Unedited Blots for Supplemental Figure 2B Continued

ERK

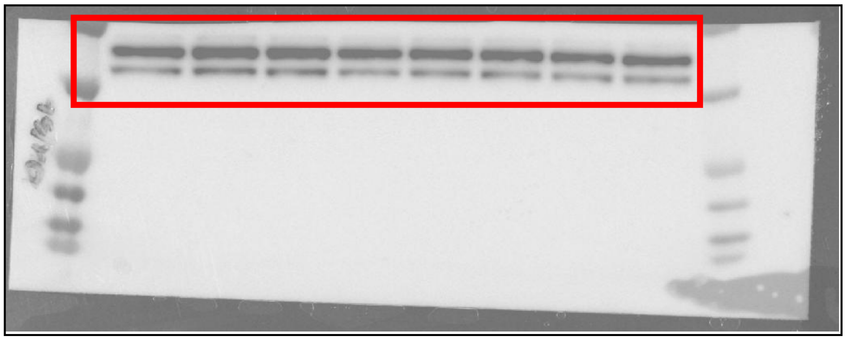

P-PLCg

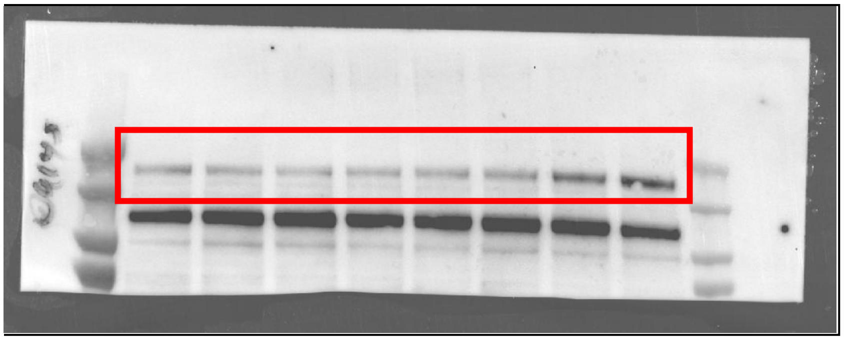

PLCg

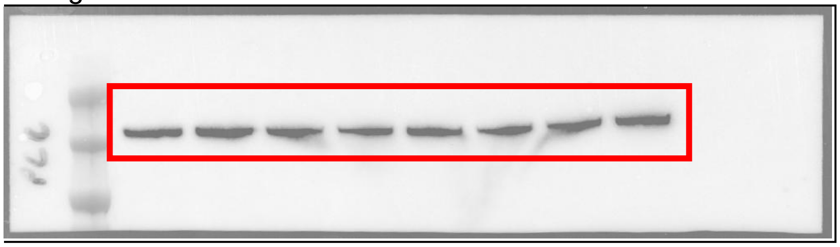

P-CAMKII

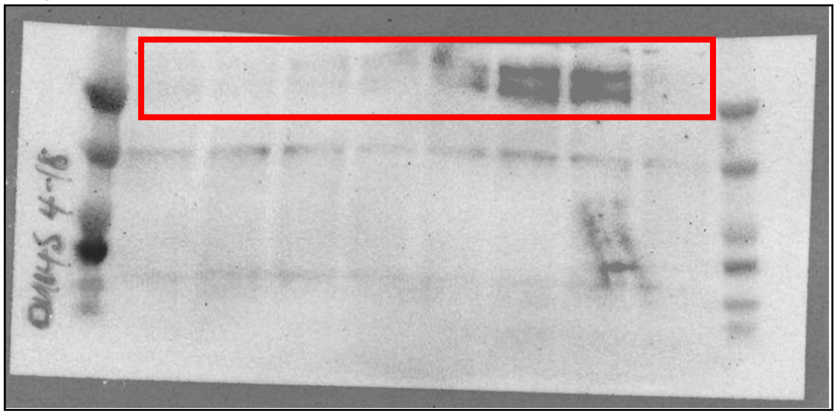

Full and Unedited Blots for Supplemental Figure 2B Continued

CAMKII

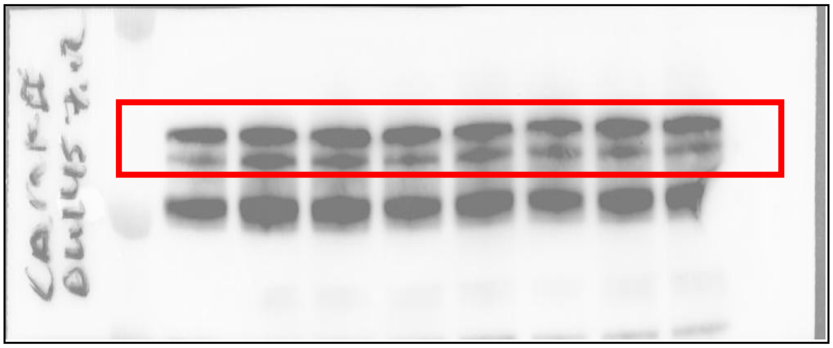

GAPDH

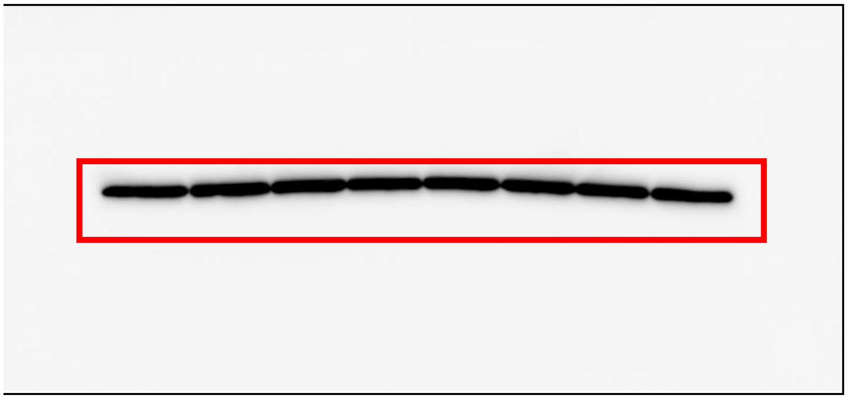

Full and Unedited Blots for Supplemental Figure 2C

PC3 + ASPN (100ng/mL)

Lanes: 0, 2, 5, 10, 15, 30, 60, 120 min ASPN unless noted

P-HER2

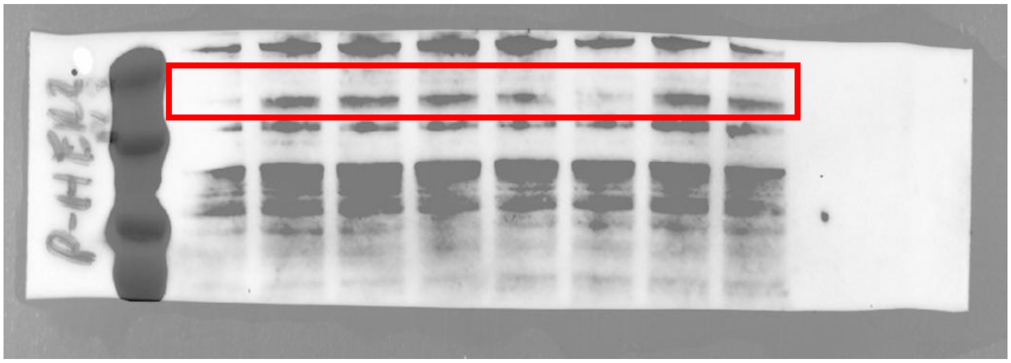

HER2

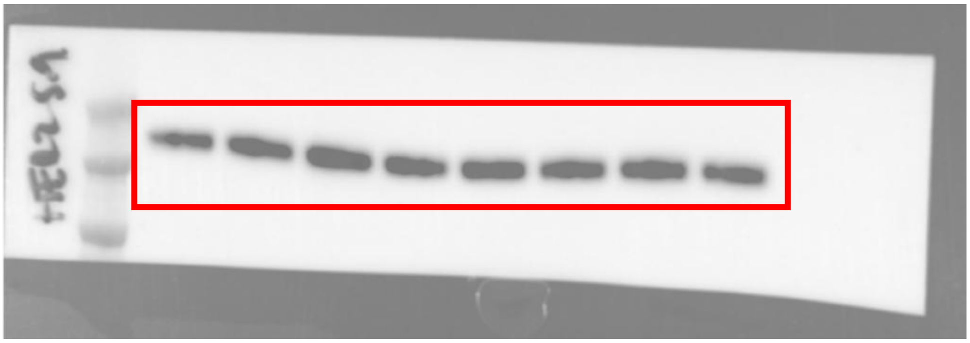

P-HER3

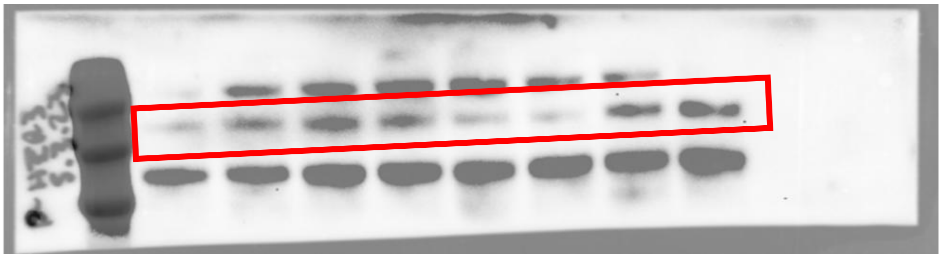

HER3

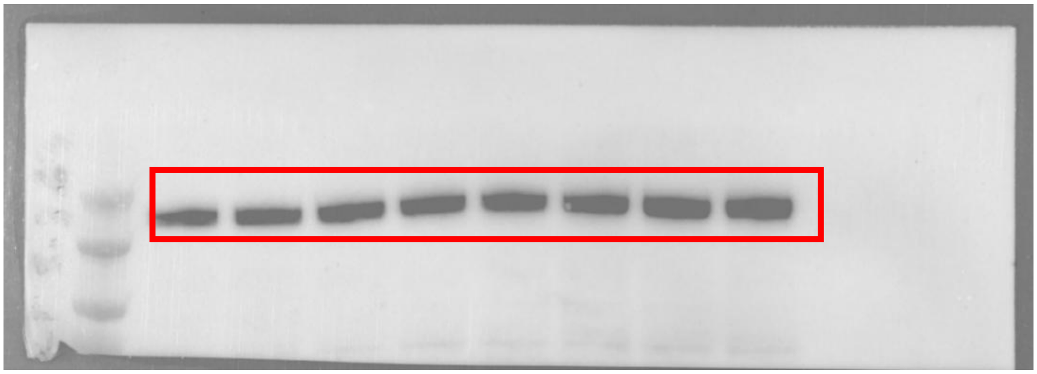

Full and Unedited Blots for Supplemental Figure 2C Continued

P-EGFR Y845

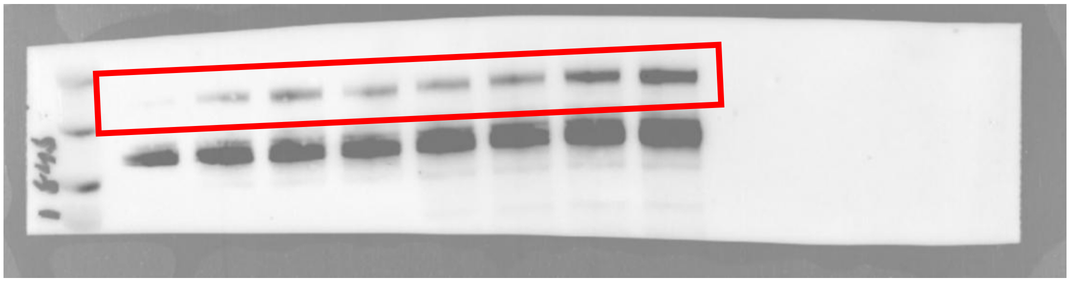

P-EGFR Y1173

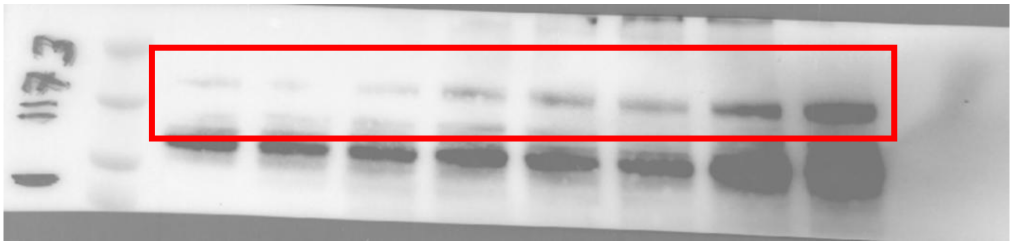

EGFR

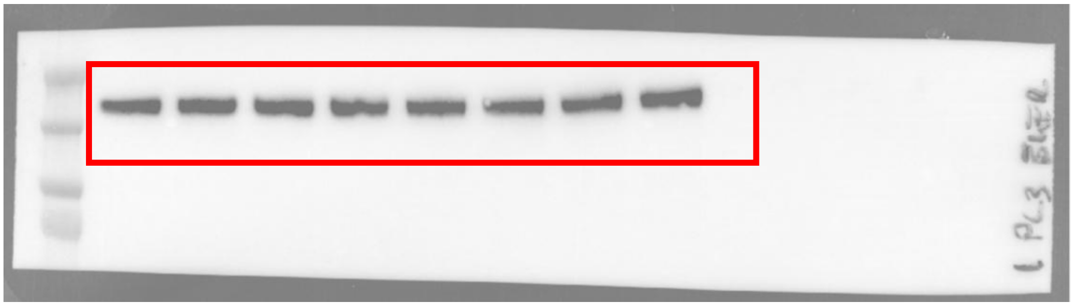

P-AKT

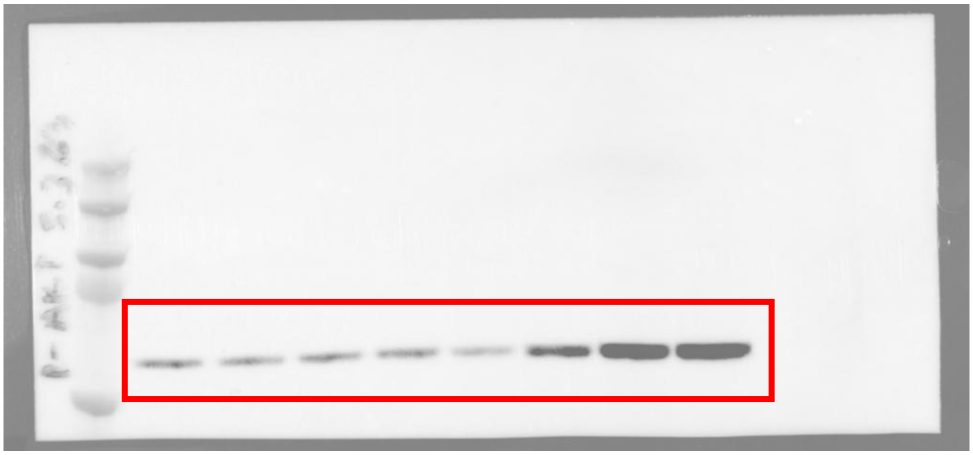

Full and Unedited Blots for Supplemental Figure 2C Continued

AKT

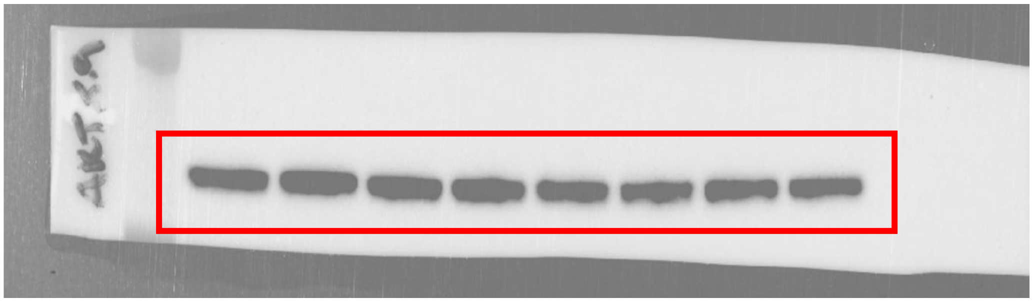

P-ERK

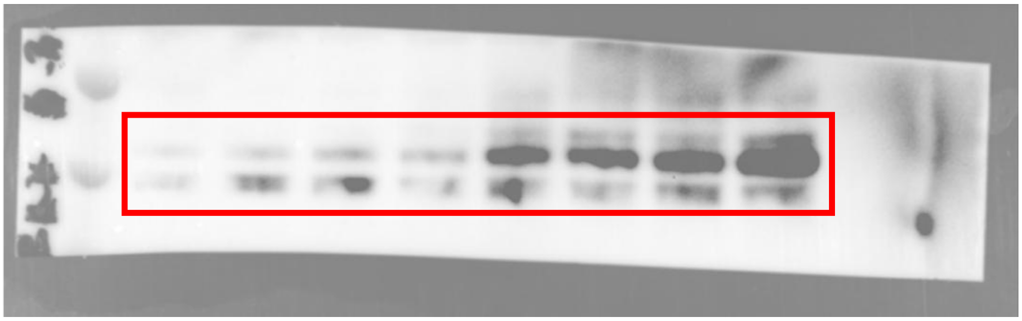

ERK

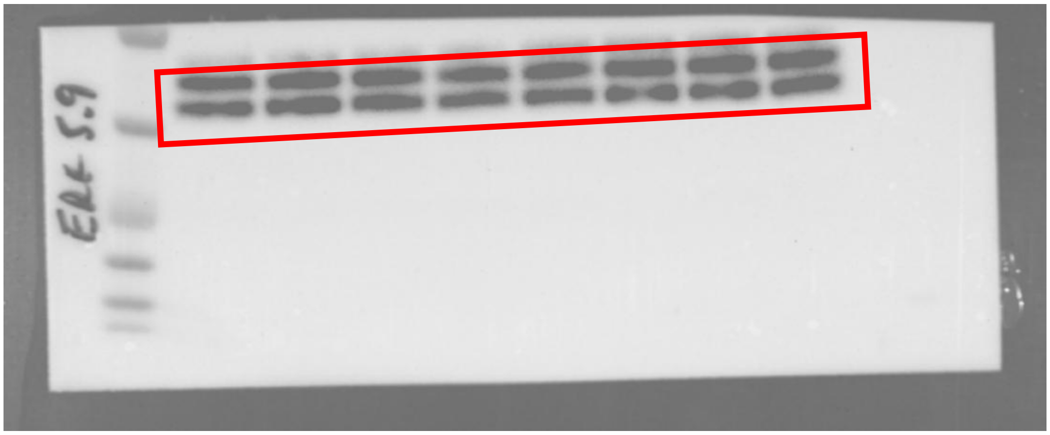

P-PLC

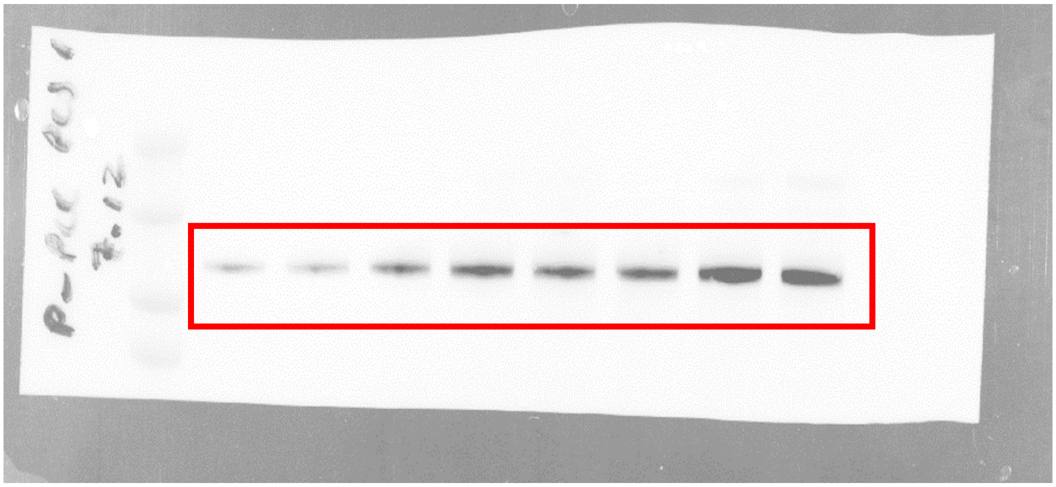

Full and Unedited Blots for Supplemental Figure 2C Continued

PLC

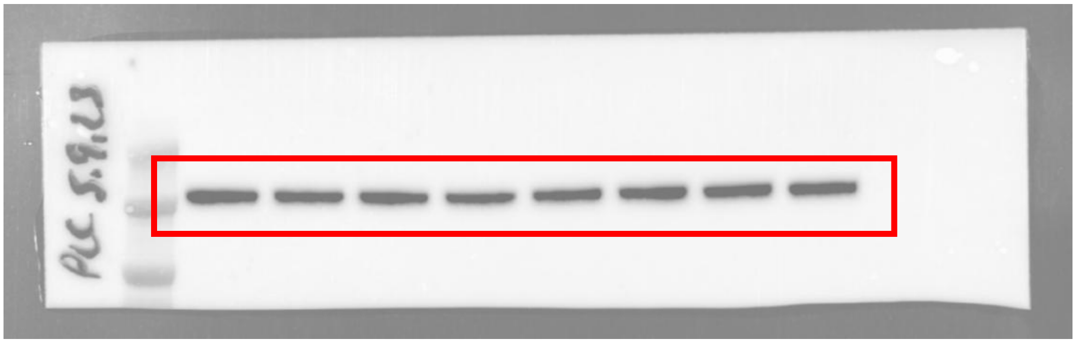

P-CAMKII

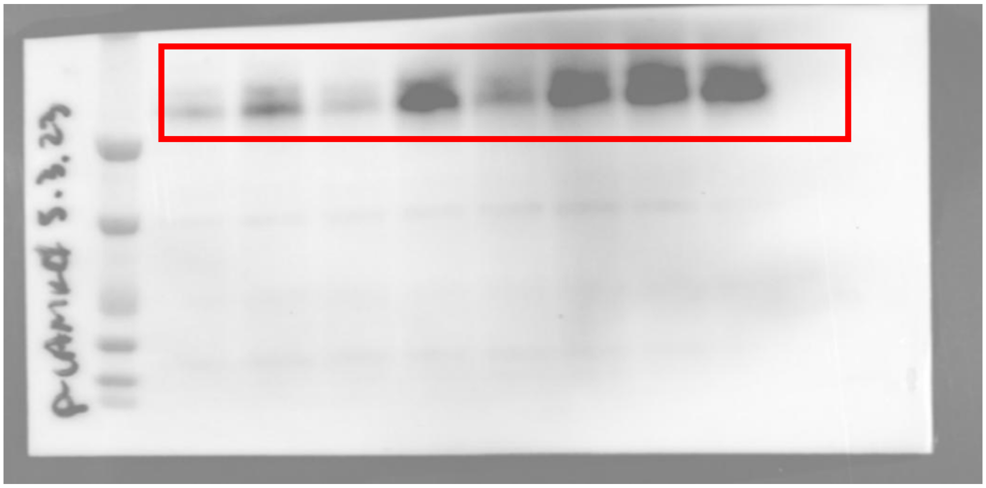

CAMKII

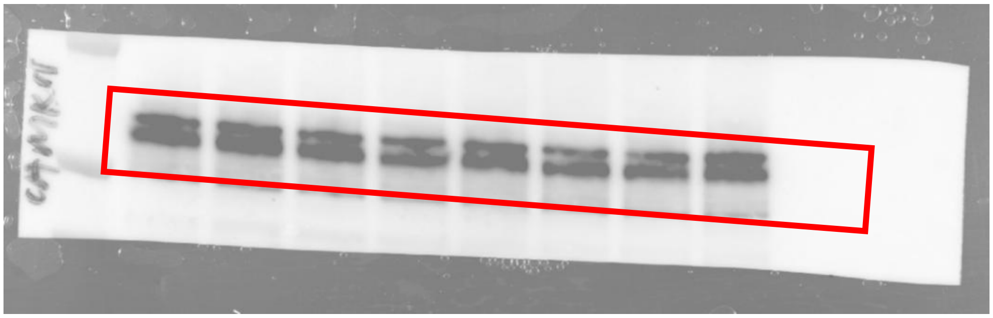

GAPDH

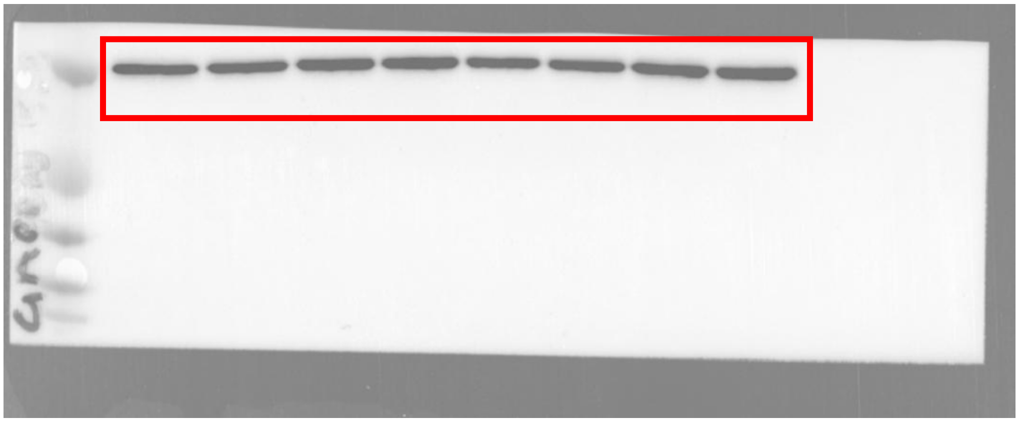

Full and Unedited Blots for Supplemental Figure 2E

LNCaP (Trial 2)

Lanes: EGF Control, ASPN 0, 2, 5, 10, 15, 30, 60, 120

P-EGFR Y845

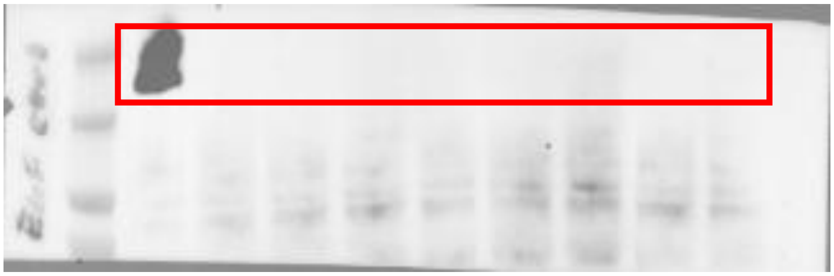

Lanes: EGF control, 0, 2, 5, 10, 15, 30, 60, 120

P-EGFR Y1173

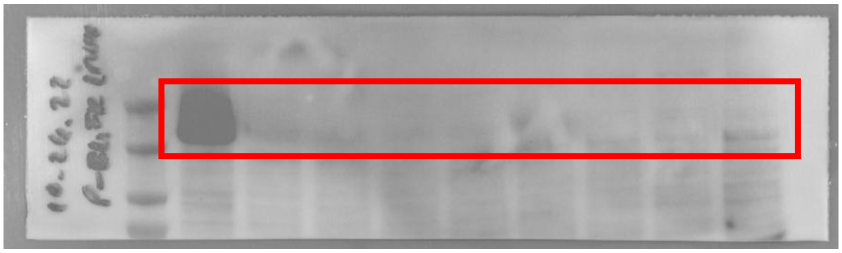

Lanes: EGF control, 0, 2, 5, 10, 15, 30, 60, 120

EGFR

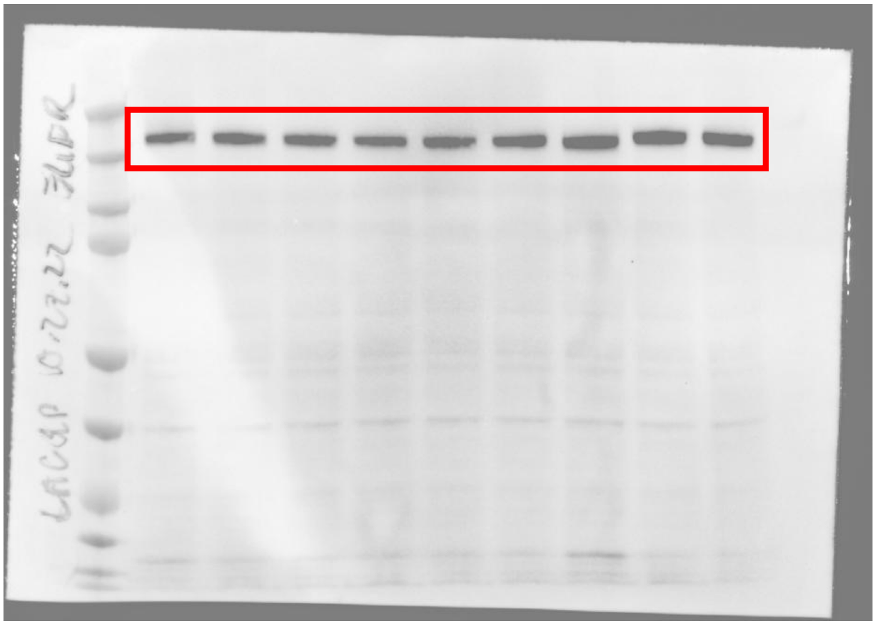

Full and Unedited Blots for Supplemental Figure 2E Continued

GAPDH

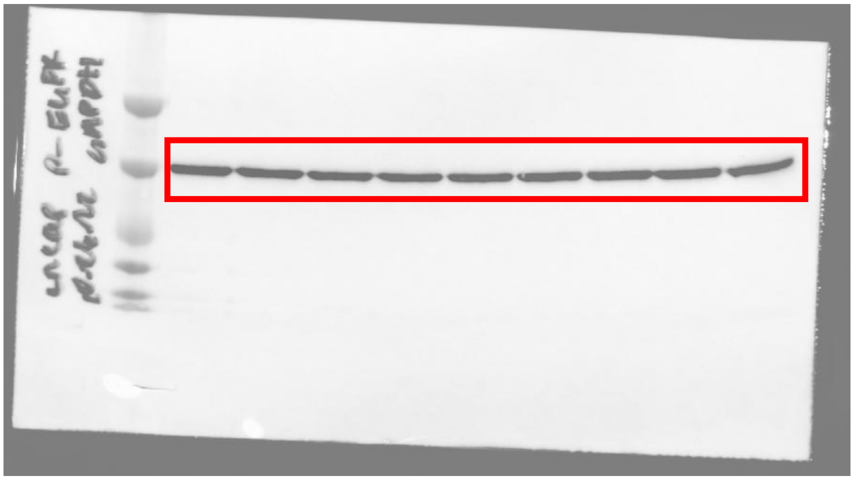

Full and Unedited Blots for Supplemental Figure 2F

VCaP

Lanes: EGF Control, ASPN 0, 2, 5, 10, 15, 30, 60, 120

P-EGFR Y845

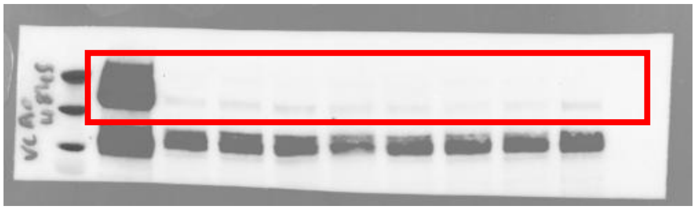

P-EGFR Y1173

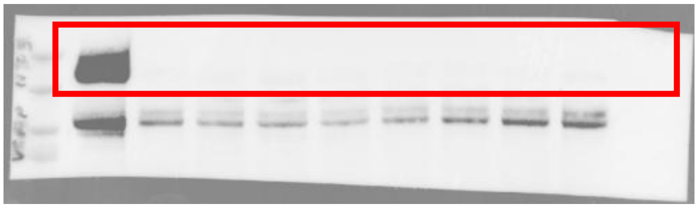

EGFR

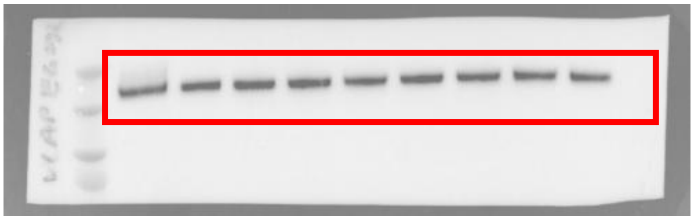

GAPDH

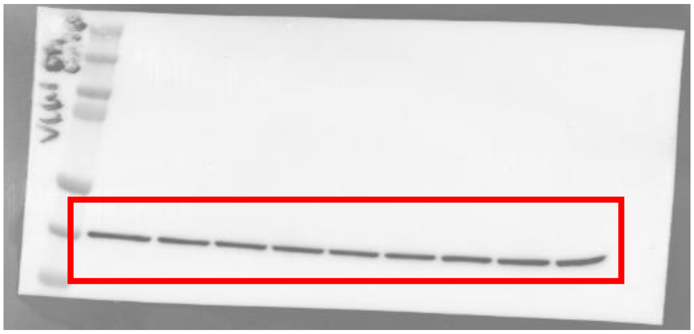

Full and Unedited Blots for Supplemental Figure 2G

LNCaP EnzaR

Lanes: EGF Control, ASPN 0, 2, 5, 10, 15, 30, 60, 120

P-EGFR Y845

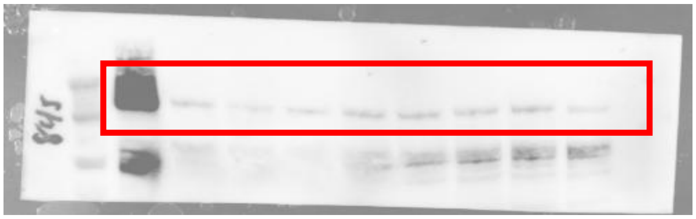

P-EGFR Y1173

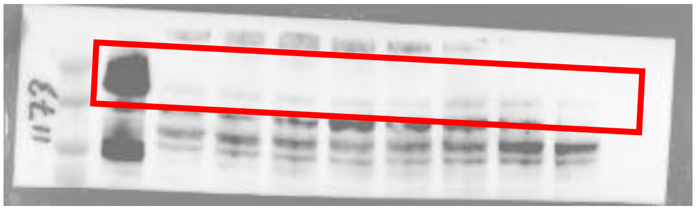

EGFR

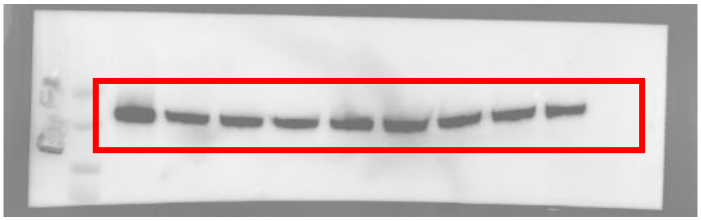

GAPDH

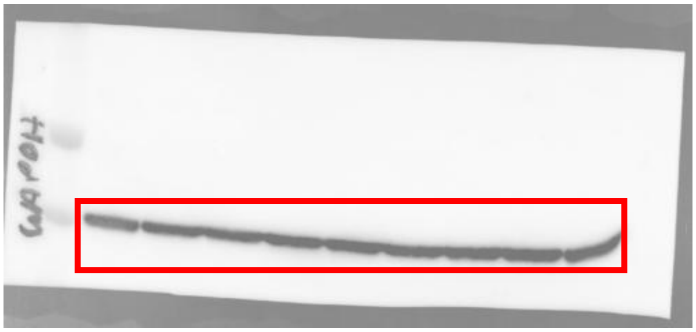

Full and Unedited Blots for Supplemental Figure 2H

VCaP EnzaR

Lanes: EGF Control, ASPN 0, 2, 5, 10, 15, 30, 60, 120

P-EGFR Y845

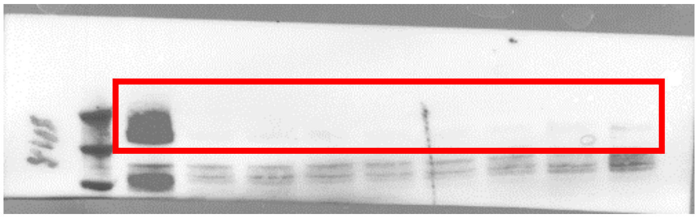

P-EGFR Y1173

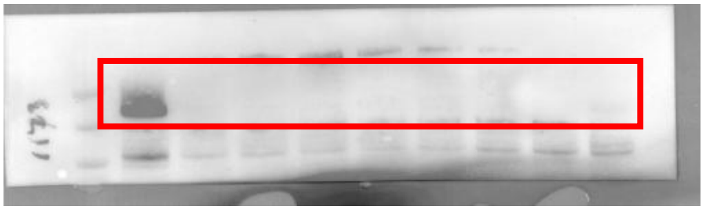

EGFR

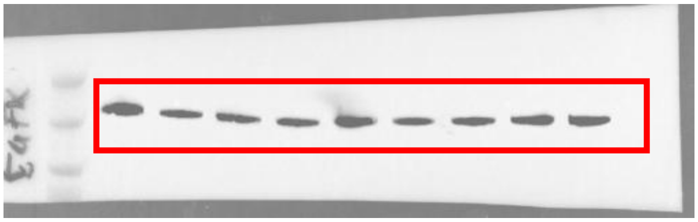

GAPDH

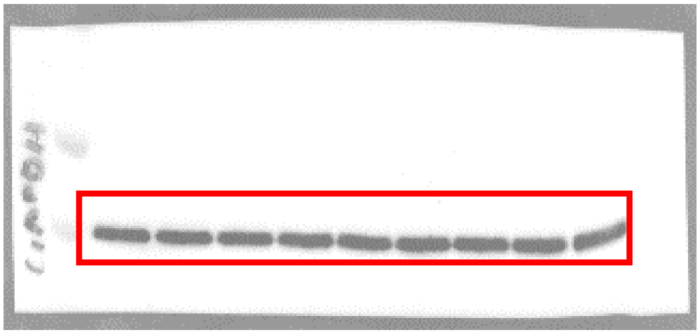

Full and Unedited Blots for Supplemental Figure 2I

DU145

Lanes: EGF Control, ASPN 0, 2, 5, 10, 15, 30, 60, 120

P-EGFR Y845

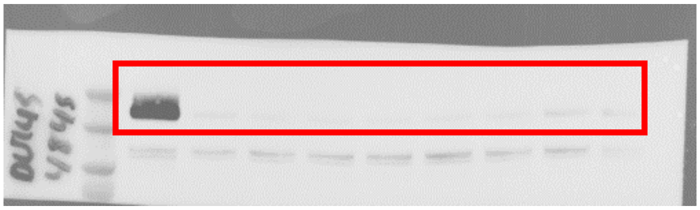

P-EGFR Y1173

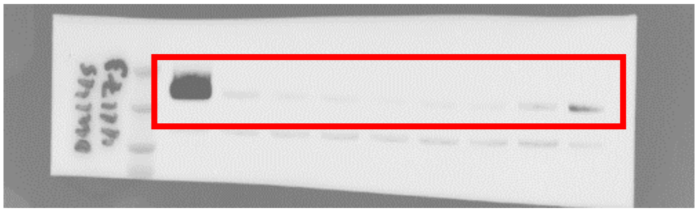

EGFR

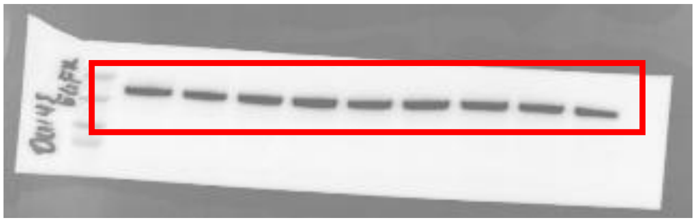

GAPDH

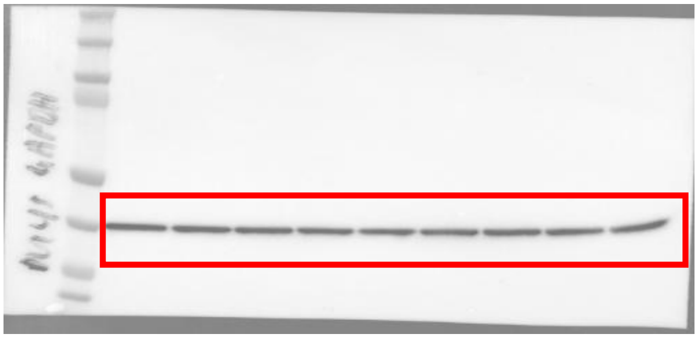

Full and Unedited Blots for Supplemental Figure 2J

MyC-CaP (Trial 2)

Lanes: EGF control, 0, 2, 5, 10, 15, 30, 60, 120

P-EGFR Y845

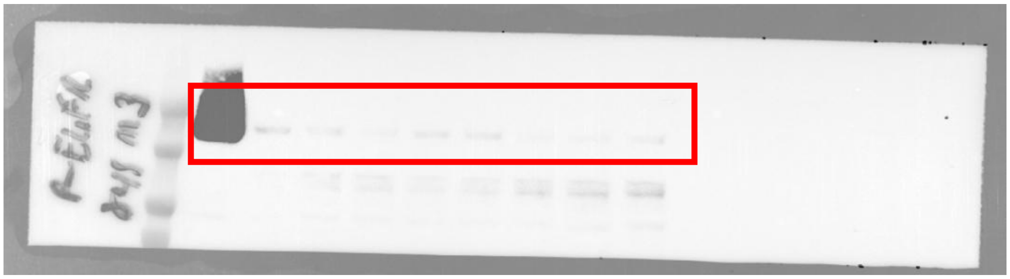

P-EGFR Y1173

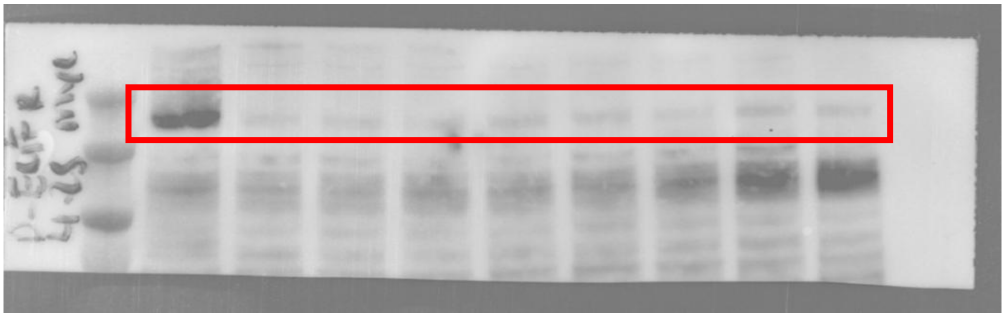

EGFR

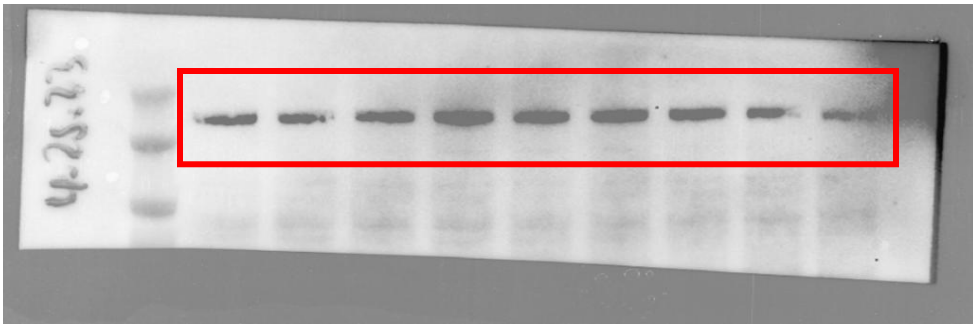

Full and Unedited Blots for Supplemental Figure 2J Continued

GAPDH

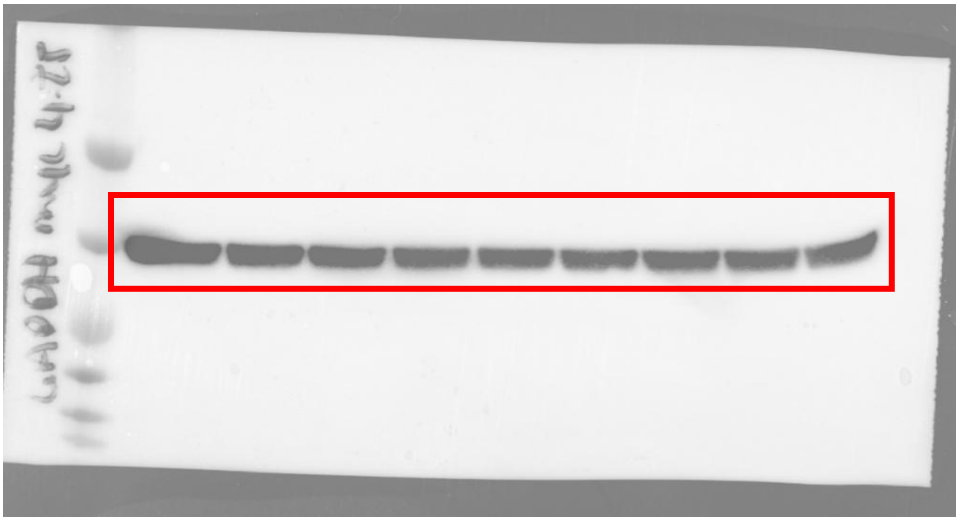

Full and Unedited Blots for Supplemental Figure 2K

Lanes: MCF7, LNCaP, VCaP, 22RV1, PC3, DU145, MyC-CaP, HEK293

EGFR

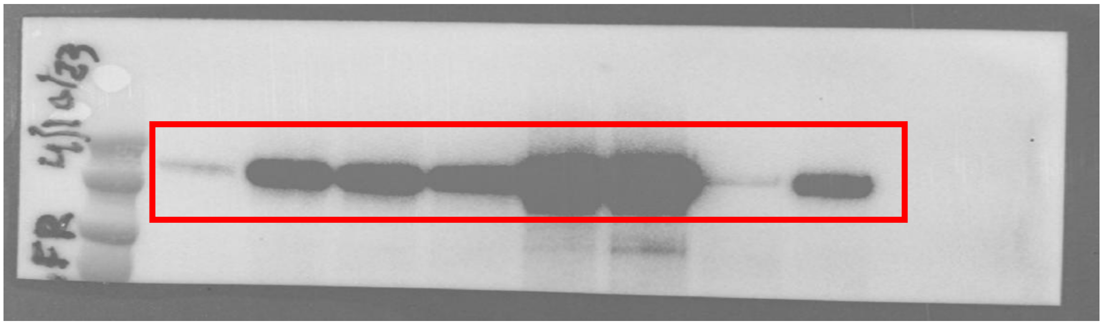

HER2

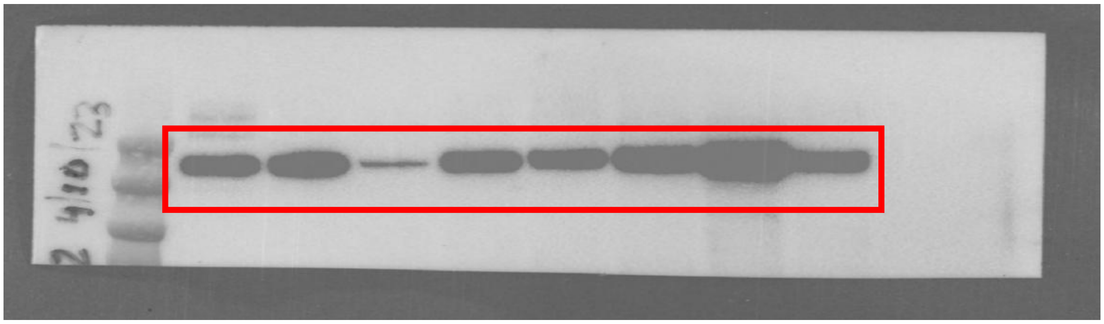

HER3

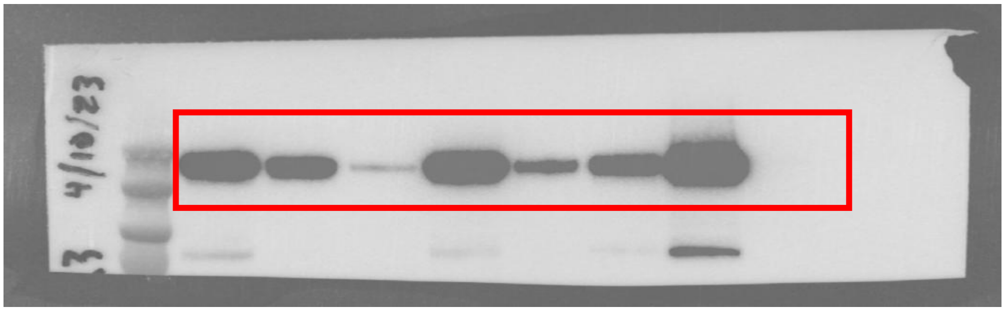

HER4

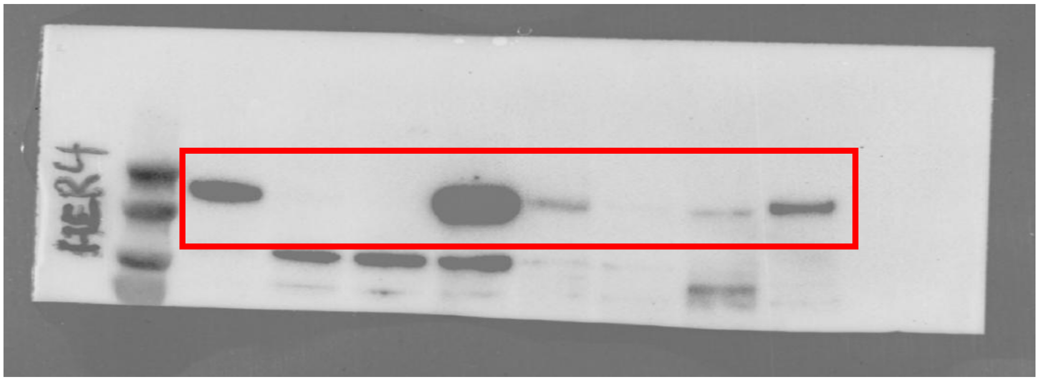

Full and Unedited Blots for Supplemental Figure 2K Continued

GAPDH

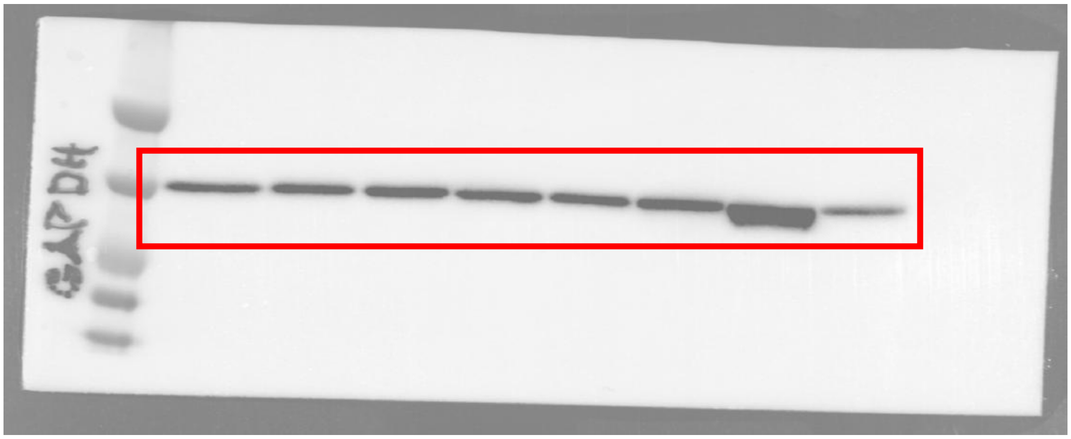

Full and Unedited Blots for Supplemental Figure 2L

Lanes: WPMY EV PCAF 48 hr serum starved
WPMY ASPN D14 3x FLAG
PCAF 24 hr full serum
PCAF 24 hr serum starved
PCAF 48 hr full serum
PCAF 48 hr serum starved

ASPN

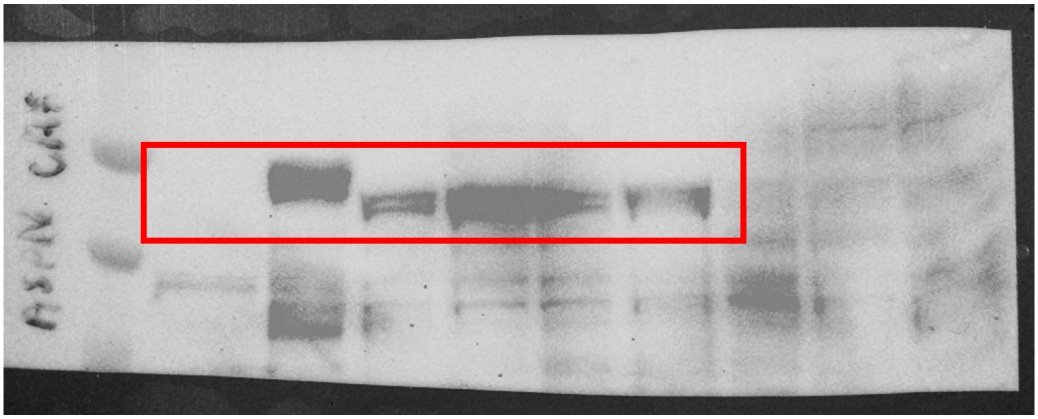

GAPDH (this blot was stripped and re-probed from the same blot above)

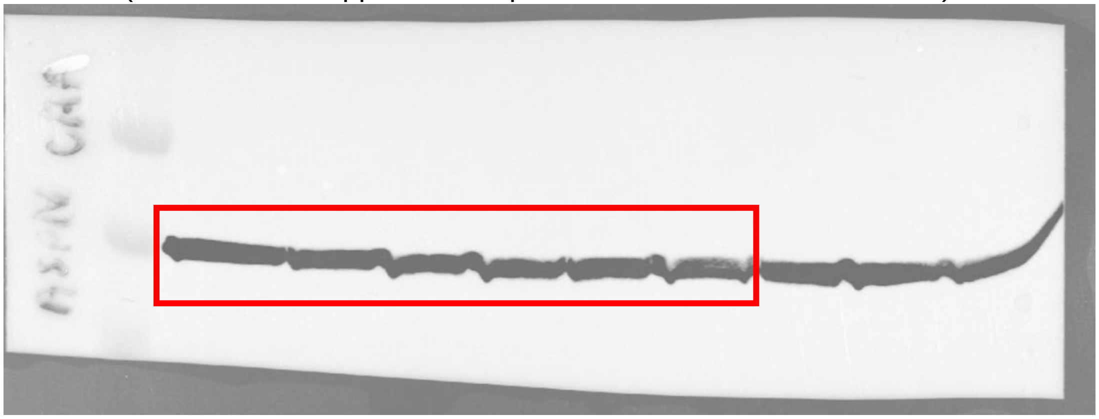

Full and Unedited Blots for Supplemental Figure 2M

Lanes: 0, 10, 15, 30, 60 and 120 min unless noted

P-HER2

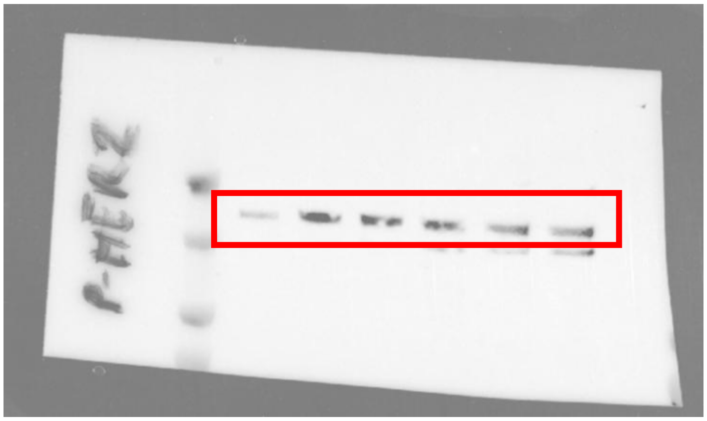

HER2

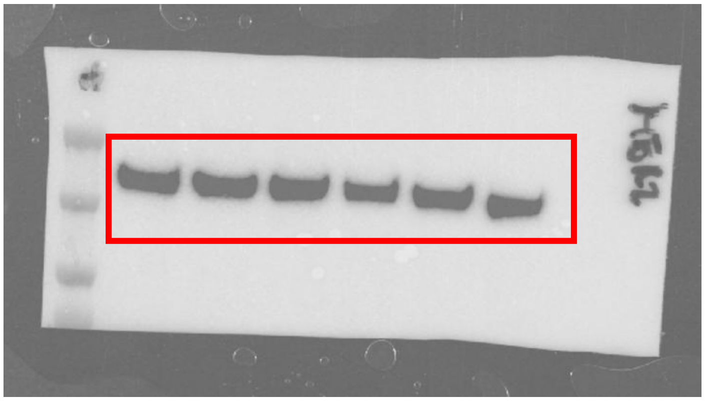

P-HER3

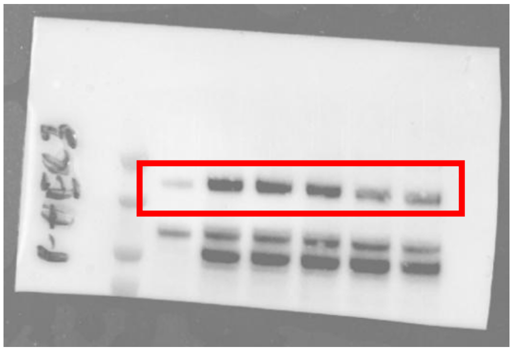

Full and Unedited Blots for Supplemental Figure 2M Continued

HER3

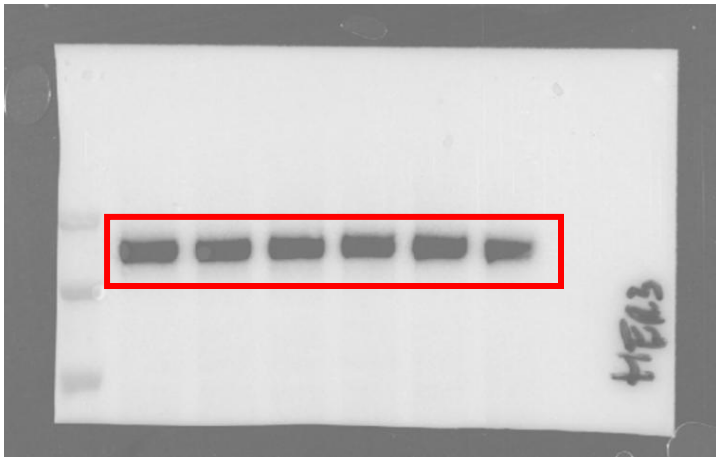

P-EGFR Y845

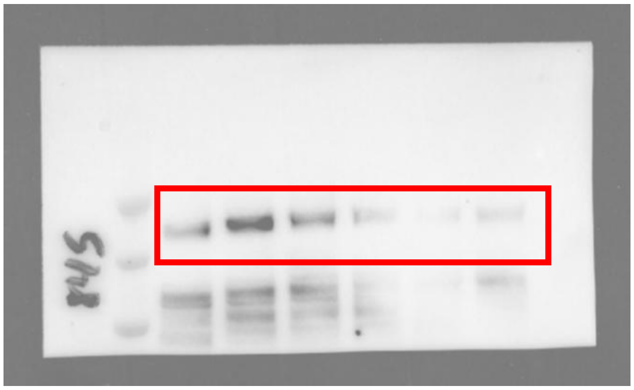

P-EGFR Y1173

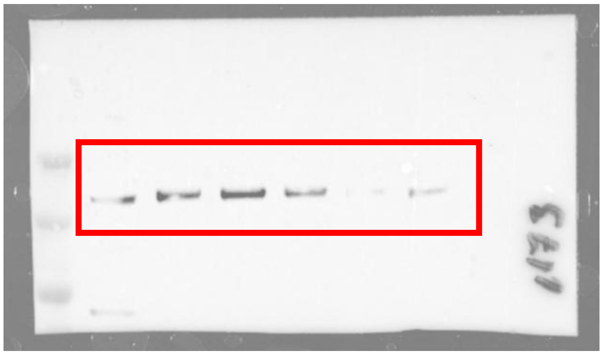

EGFR

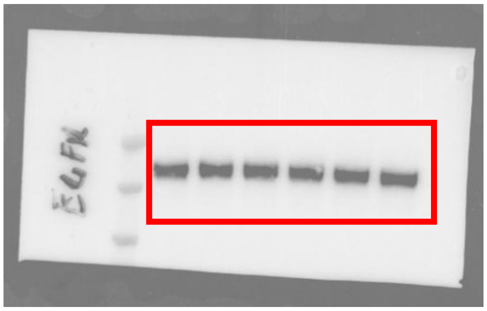

Full and Unedited Blots for Supplemental Figure 2M Continued

P-AKT

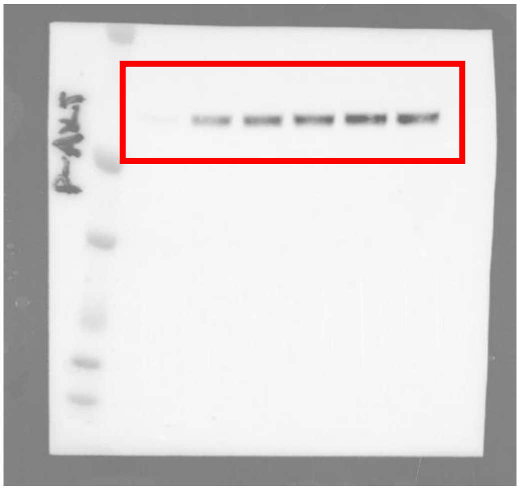

AKT

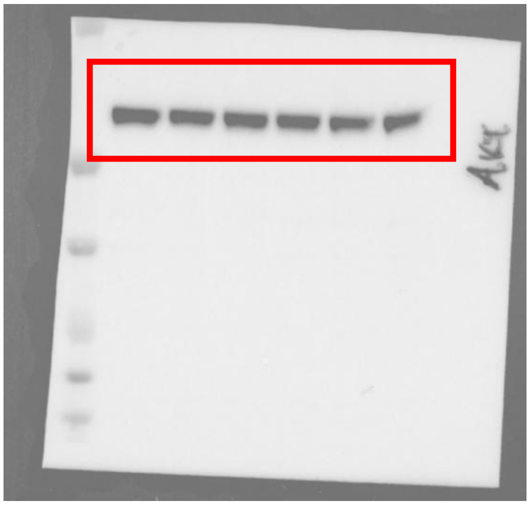

P-ERK

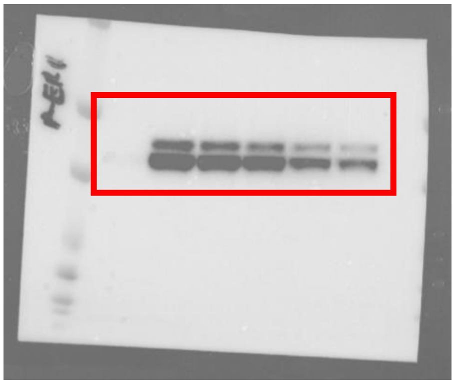

Full and Unedited Blots for Supplemental Figure 2M Continued

ERK

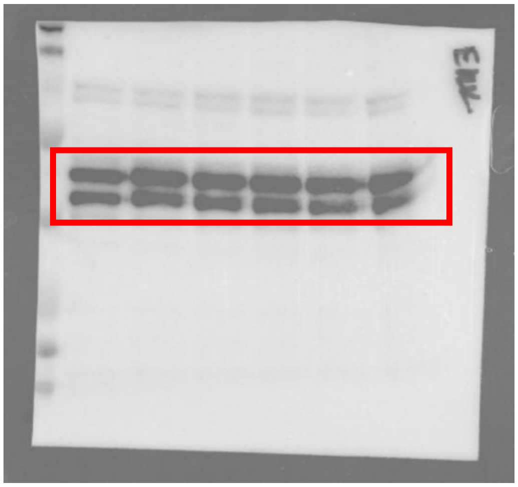

P-PLC

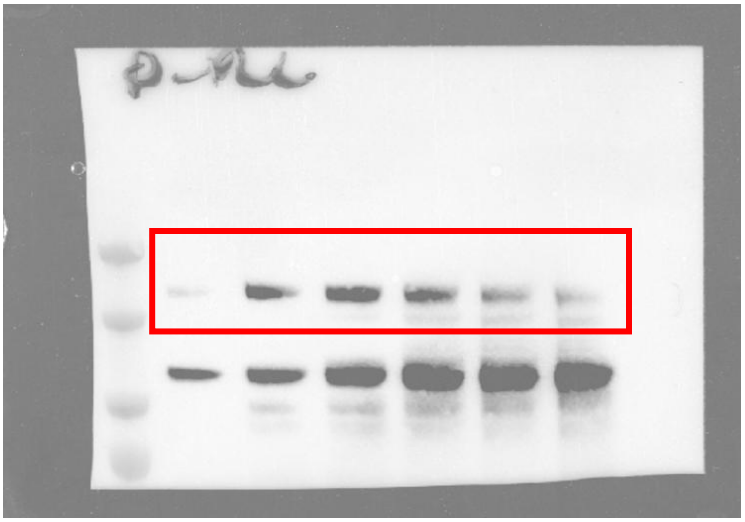

PLC

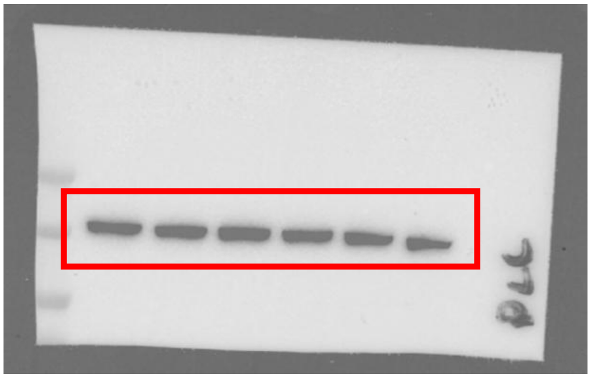

Full and Unedited Blots for Supplemental Figure 2M Continued

P-CAMKII

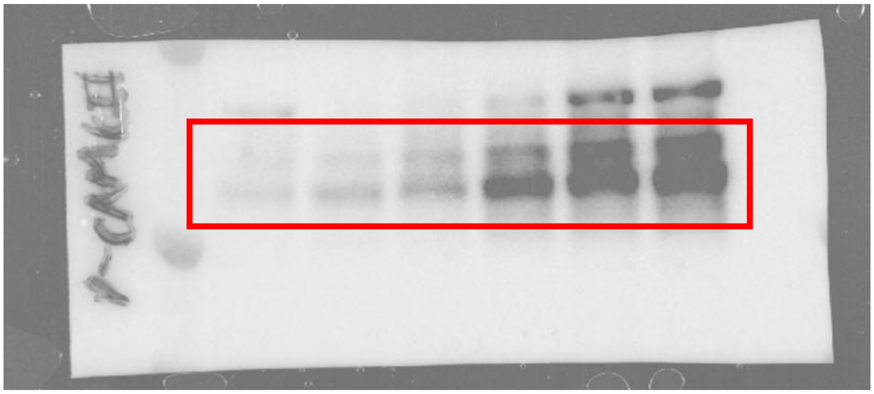

CAMKII

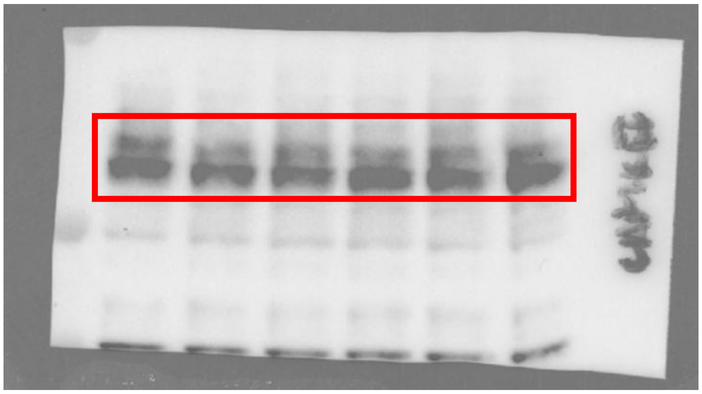

GAPDH

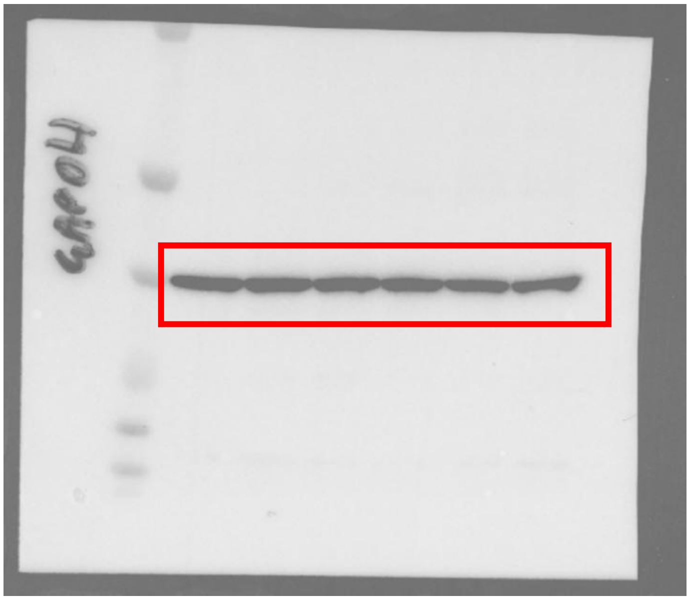

Full Unedited Blots for Supplementary Figure 4B
Recombinant Protein - HER2-FLAG

HER2
Lanes: HER2 input, HER2 IP, HER2 Post-IP
ASPN input, ASPN IP, ASPN Post-IP
HER2+ASPN Input, HER2+ASPN IP, HER2+ASPN Post-IP

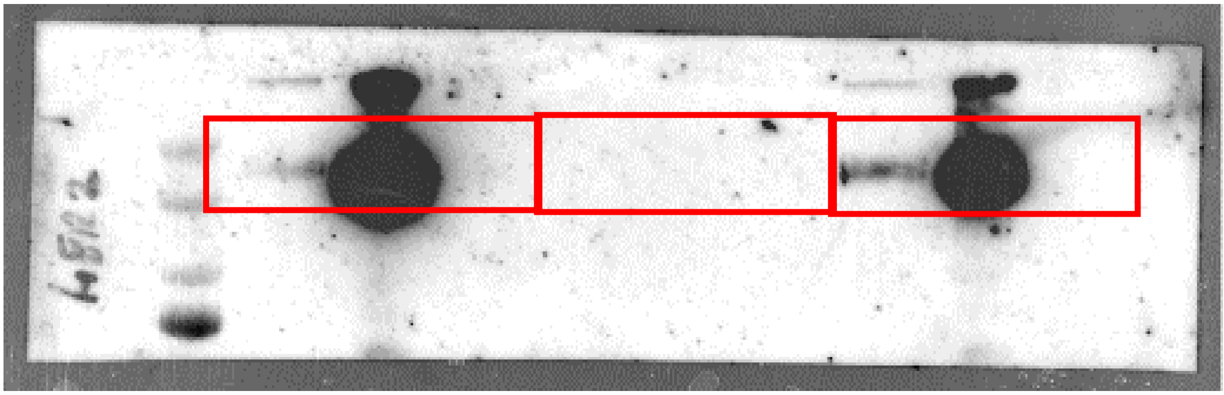

ASPN
Lanes: HER2 input, HER2 IP, HER2 Post-IP
ASPN input, ASPN IP, ASPN Post-IP
HER2+ASPN Input, HER2+ASPN IP, HER2+ASPN Post-IP

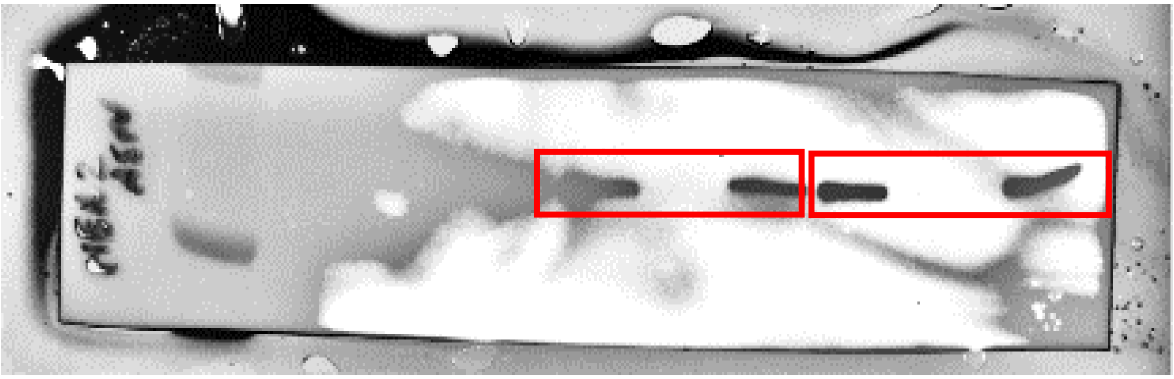

less exposed for HER2 band

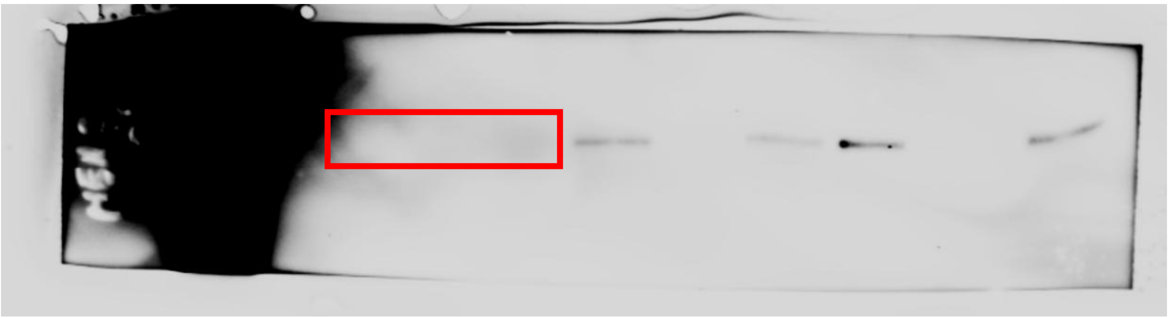

Full Unedited Blots for Supplementary Figure 4C
Recombinant Protein - PDGFRB-FLAG

PDGFRB
Lanes: PDGFRB input, PDGFRB IP, PDGFRB Post-IP
ASPN input, ASPN IP, ASPN Post-IP,
PDGFRB+ASPN Input, PDGFRB+ASPN IP, PDGFRB+ASPN Post-IP

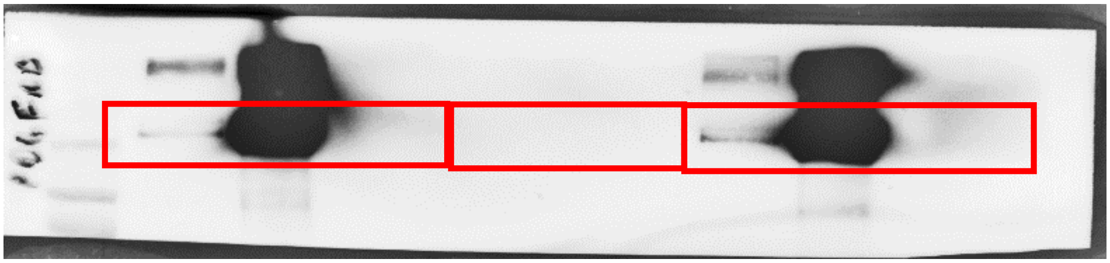

ASPN
Lanes: PDGFRB input, PDGFRB IP, PDGFRB Post-IP
ASPN input, ASPN IP, ASPN Post-IP
PDGFRB+ASPN Input, PDGFRB+ASPN IP, PDGFRB+ASPN Post-IP

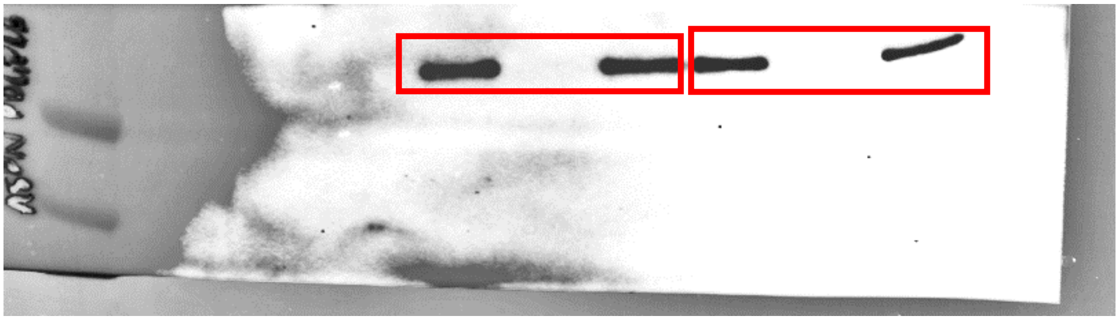

less exposed for PDGFRB band

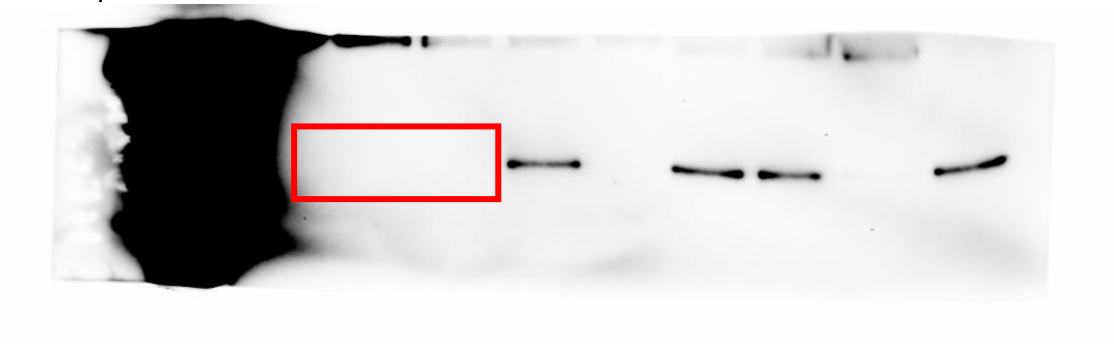

Full and Unedited Blots for Supplemental Figure 4D

PC3 + recombinant ASPN and IP for endogenous HER3 and HER2

HER3
Lanes: Input 0 min, IP 0 min, Post-IP 0 min, Input 15 min, IP 15 min, Post-IP 15 min, Input 30 min, IP 30 min, Post-IP 30 min

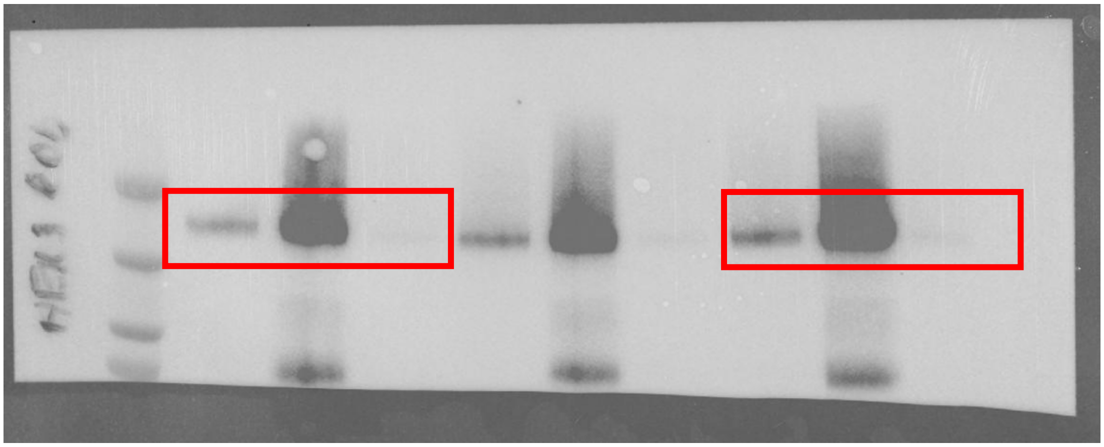

HER2

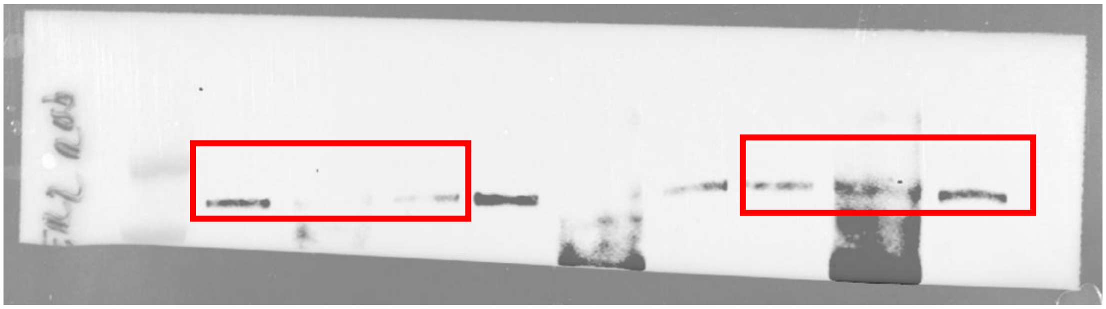

Full and Unedited Blots for Supplemental Figure 4E

PC3 + PCAF CM IP for endogenous HER3 and HER2

Lanes: Input 0 min, IP 0 min, Post-IP 0 min, Input 15 min, IP 15 min, Post-IP 15 min, Input 30 min, IP 30 min, Post-IP 30 min

HER3

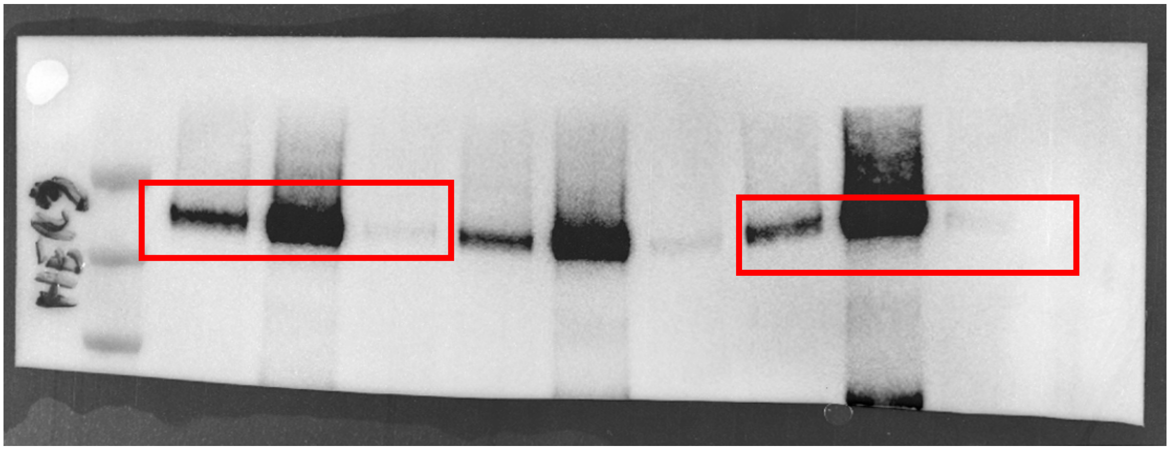

HER2

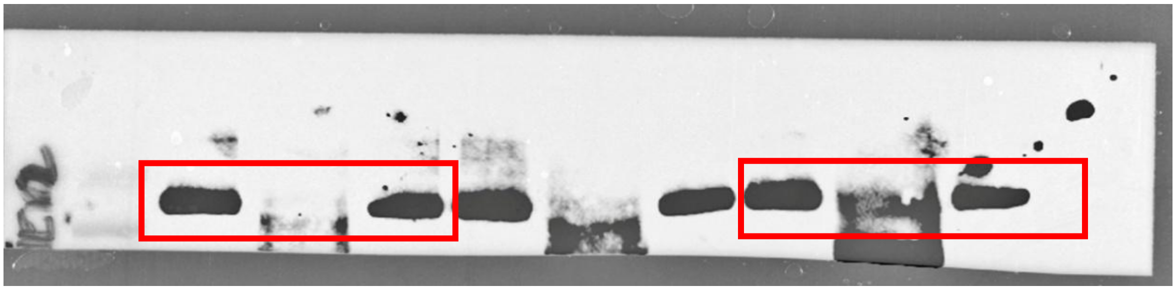

Full and Unedited Blots for Supplemental Figure 4G

LNCaP HER2 KO

Lanes: Parental, TWT C9, TWT, D12, HET C6, HET C10, KO C12, KO D5

HER2

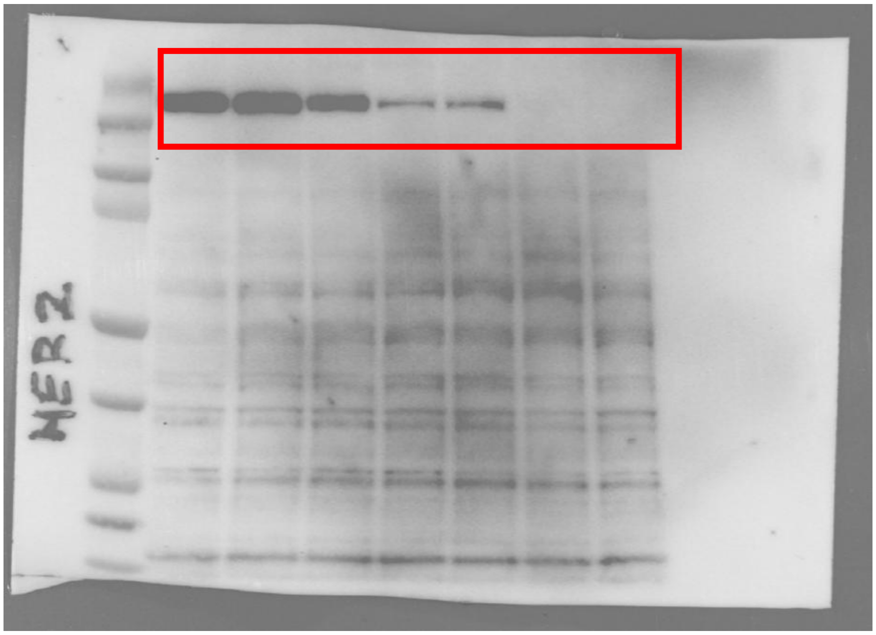

HER3

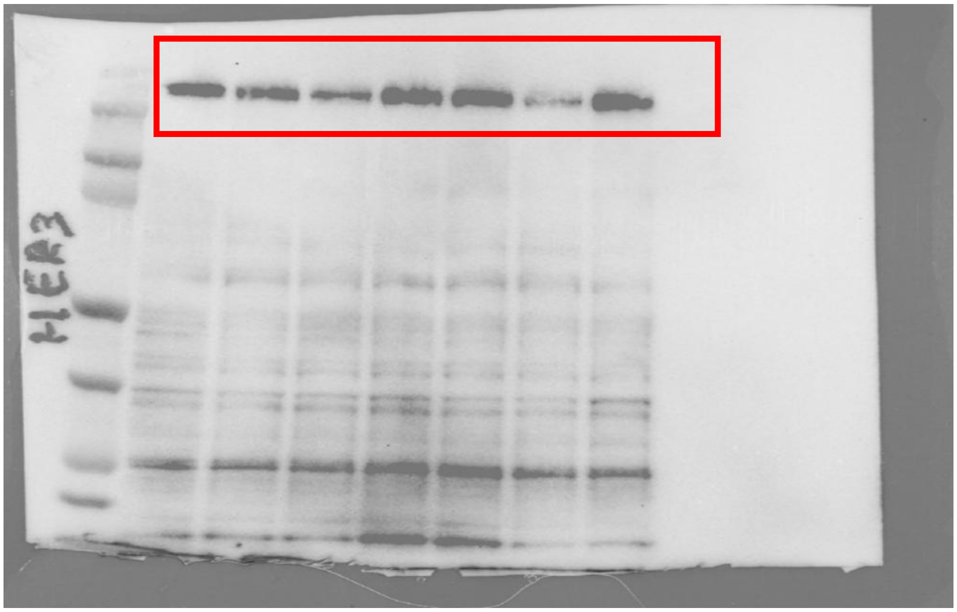

Full and Unedited Blots for Supplemental Figure 4G Continued

EGFR

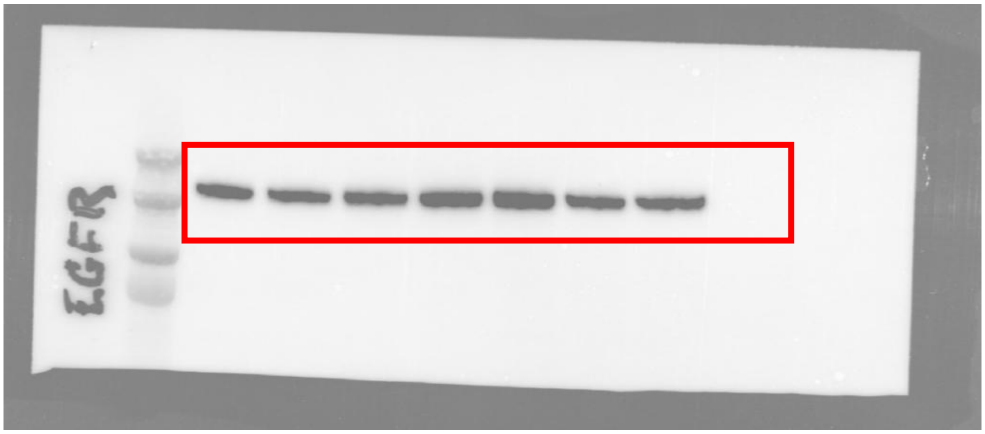

HER4

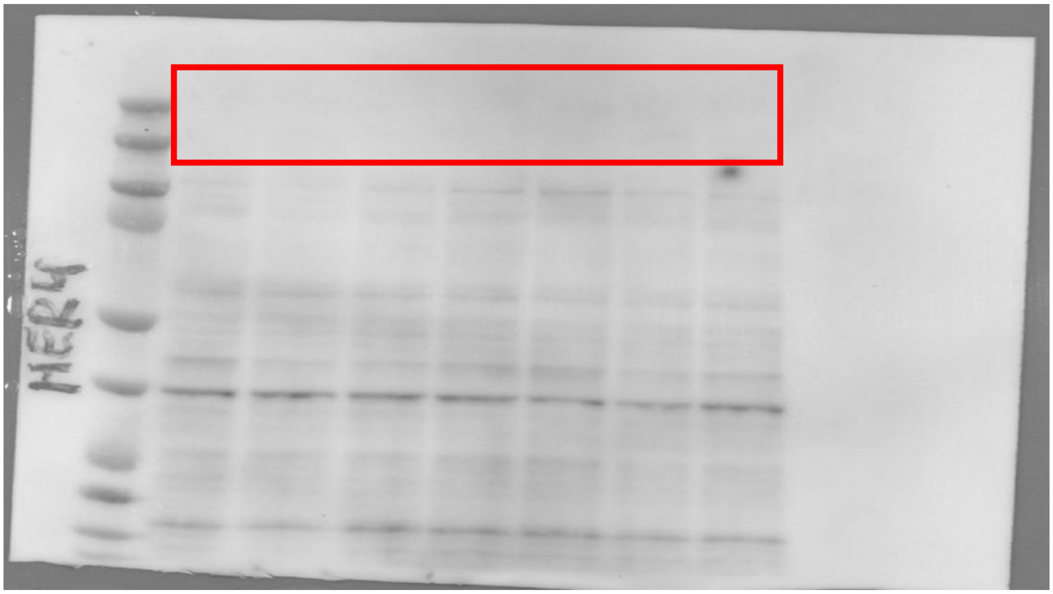

Full and Unedited Blots for Supplemental Figure 4G Continued

GAPDH

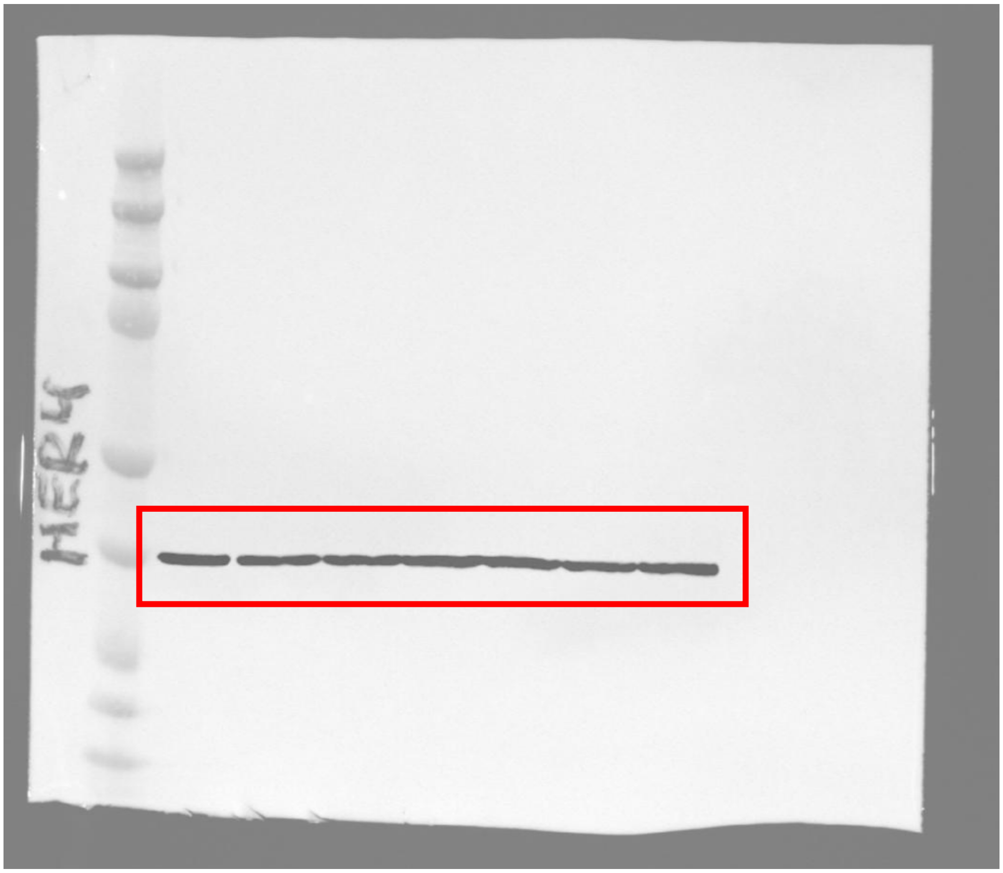

Full and Unedited Blots for Supplemental Figure 4I

LNCaP HER3 TKD

Lanes: Parental, TWT E4, TWT E11, HET E6, HET E10, TKD E3, TKD F1

HER3

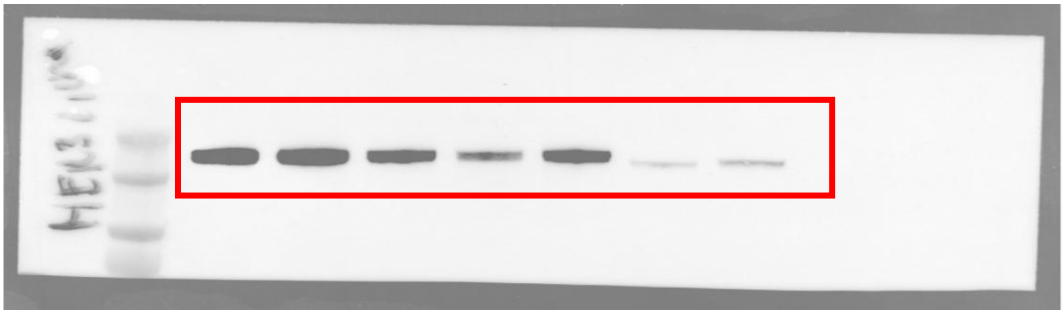

HER2

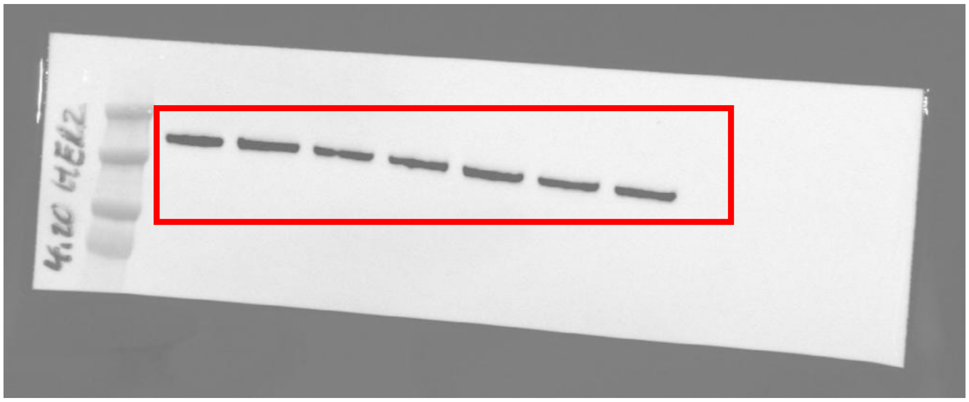

EGFR

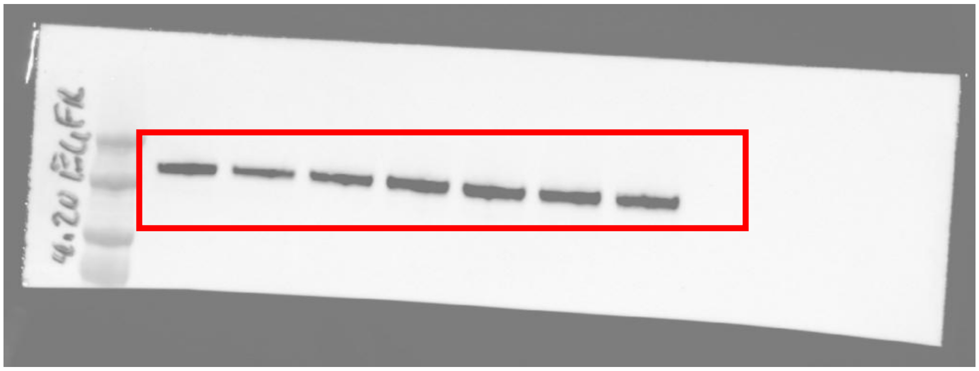

Full and Unedited Blots for Supplemental Figure 4I Continued

HER4

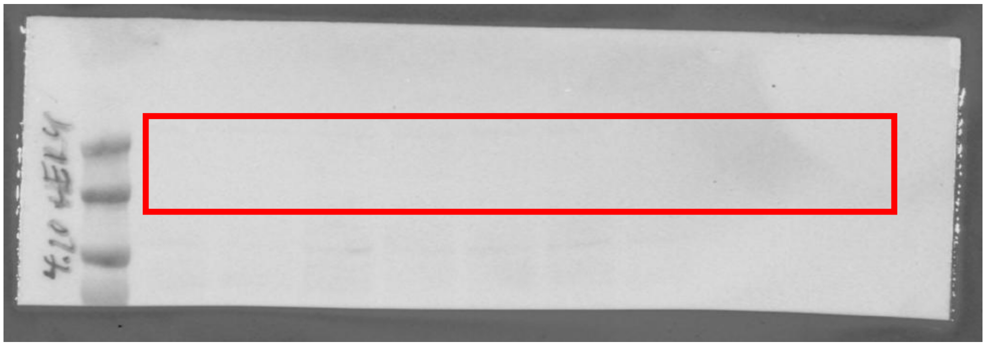

GAPDH

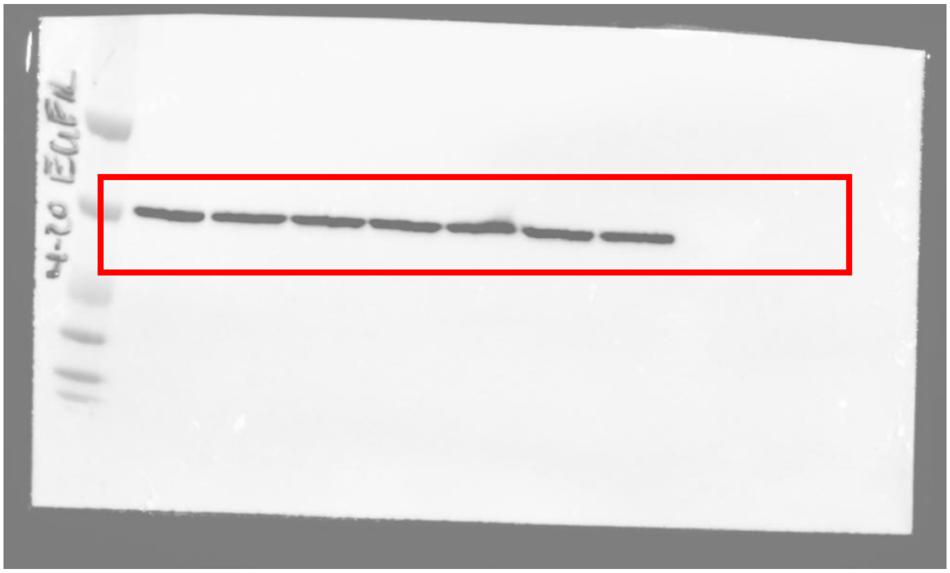

Full and Unedited Blots for Supplemental Figure 5A

LNCaP HER3 TKD + rASPN (100 ng/mL)

Lanes: TWT 0, 5, 10, 15, 30, 60, TKD 0, 5, 10, 15, 30, 60 unless noted

P-HER2

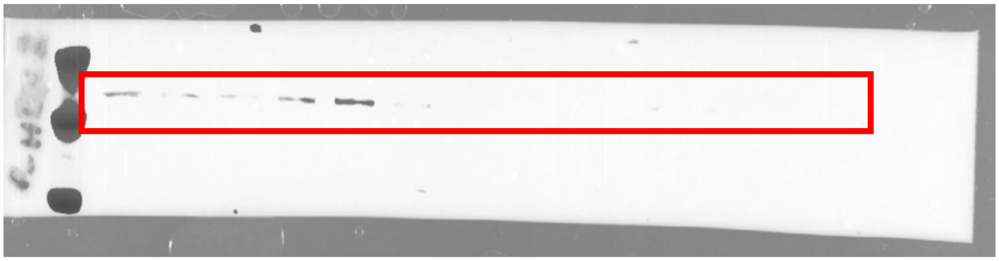

HER2

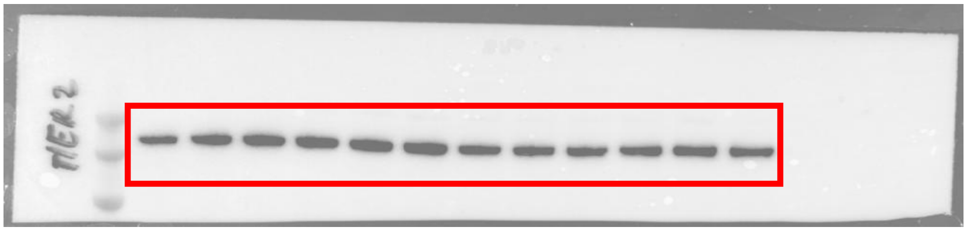

P-HER3

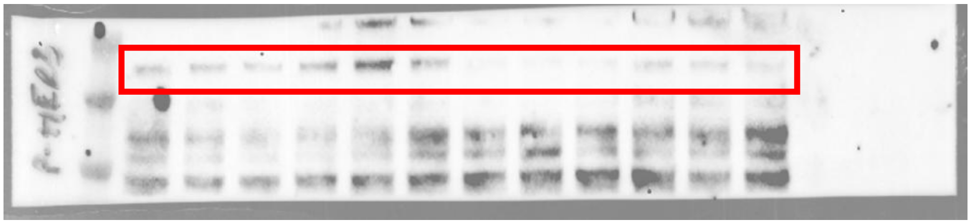

HER3

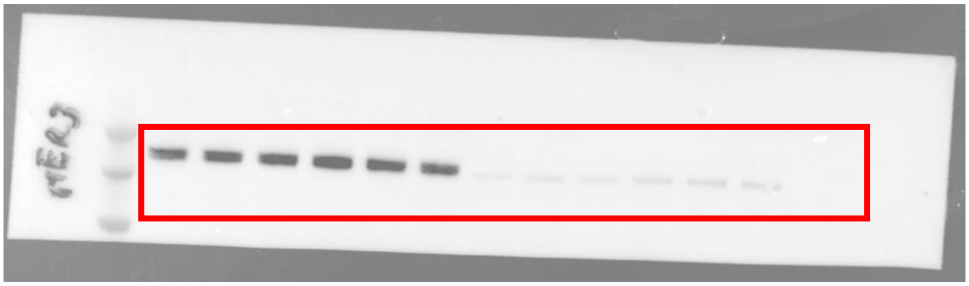

Full and Unedited Blots for Supplemental Figure 5A continued

Lanes: EGF ctrl, TWT 0, 5, 10, 15, 30, 60, KD 0, 5, 10, 15, 30, 60
P-EGFR Y845

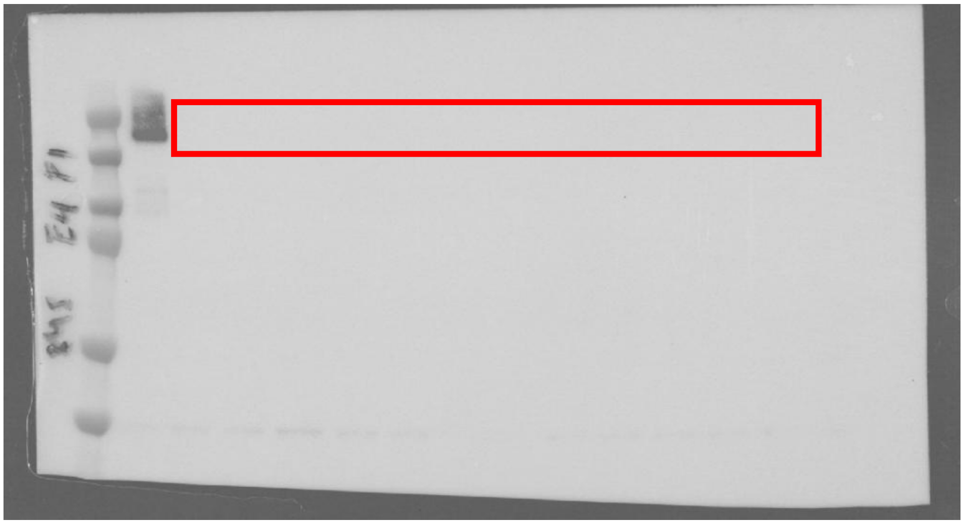

Lanes: EGF ctrl, TWT 0, 5, 10, 15, 30, 60, KD 0, 5, 10, 15, 30, 60
P-EGFR Y1173

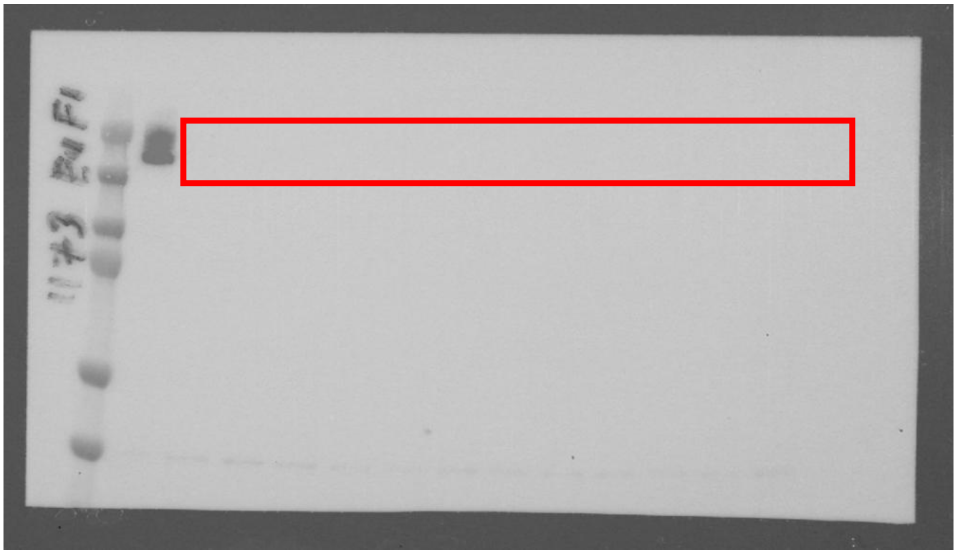

Full and Unedited Blots for Supplemental Figure 5A continued

Lanes: EGF ctrl, TWT 0, 5, 10, 15, 30, 60, KD 0, 5, 10, 15, 30, 60

EGFR

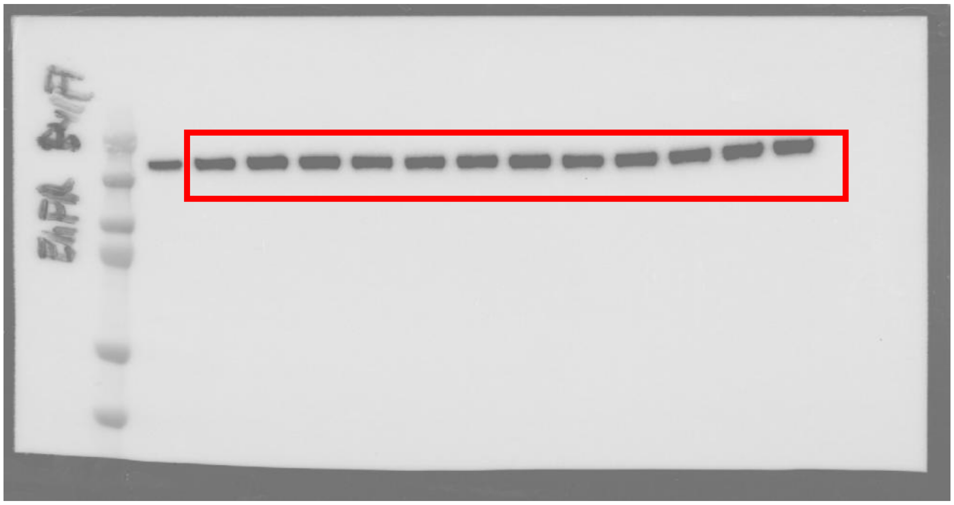

P-AKT

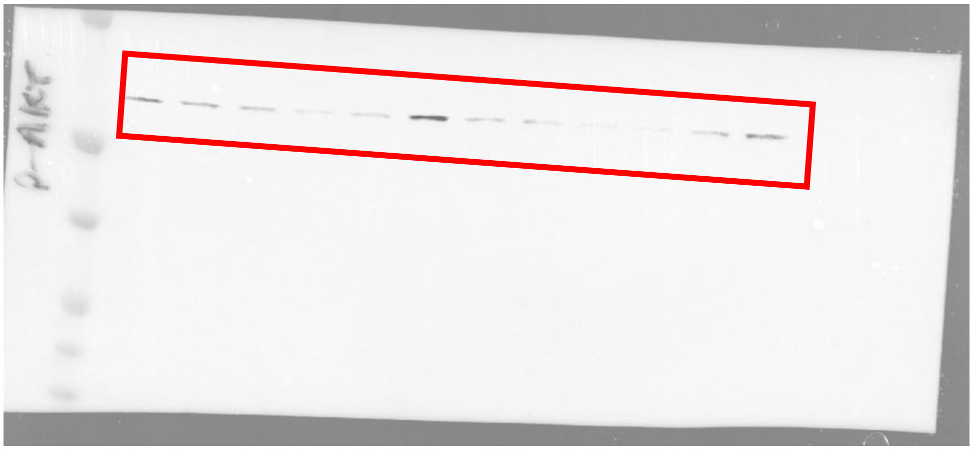

AKT

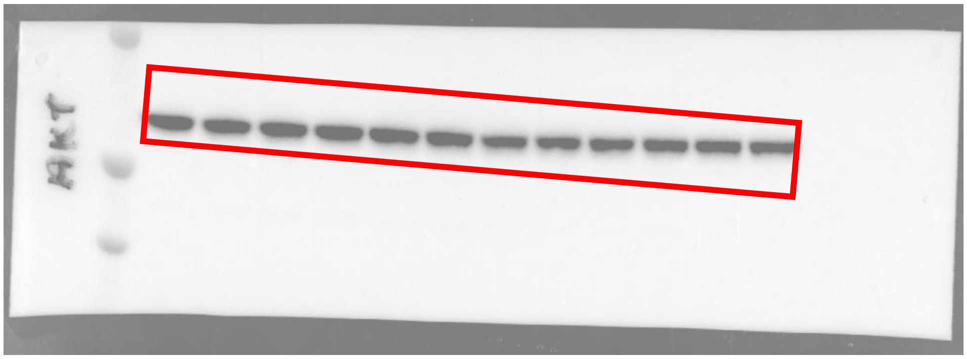

Full and Unedited Blots for Supplemental Figure 5A continued

P-ERK

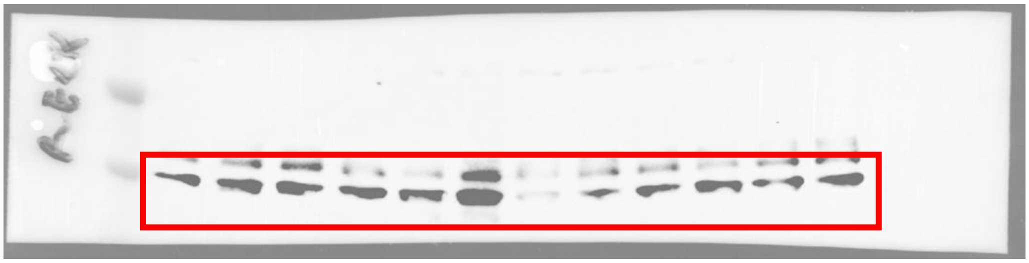

ERK

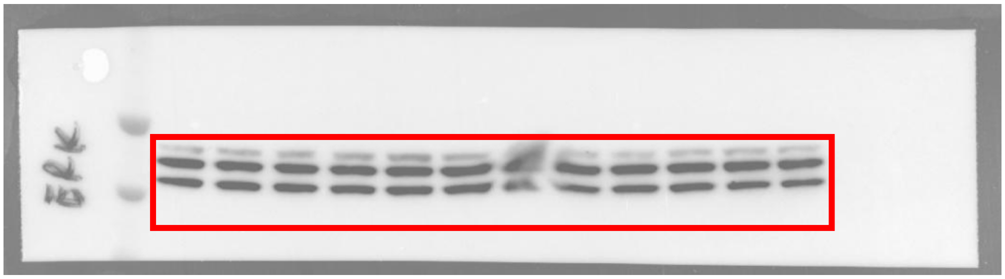

P-PLC

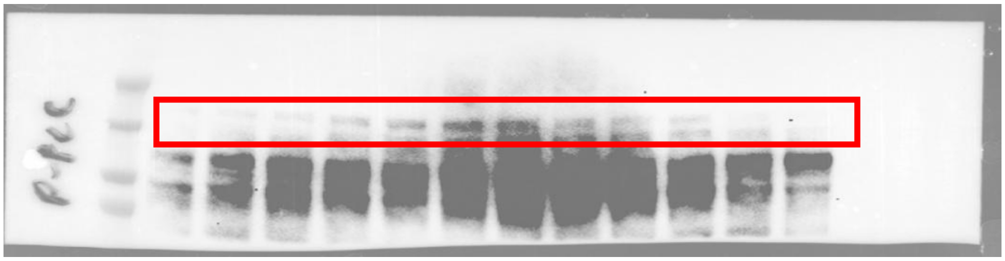

PLC

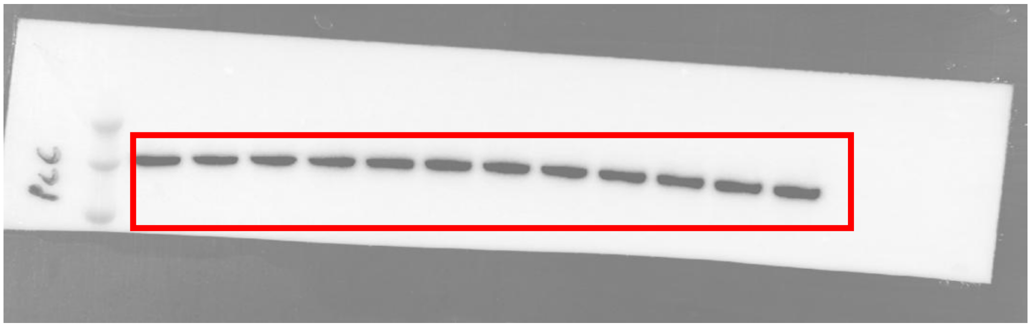

P-CAMKII

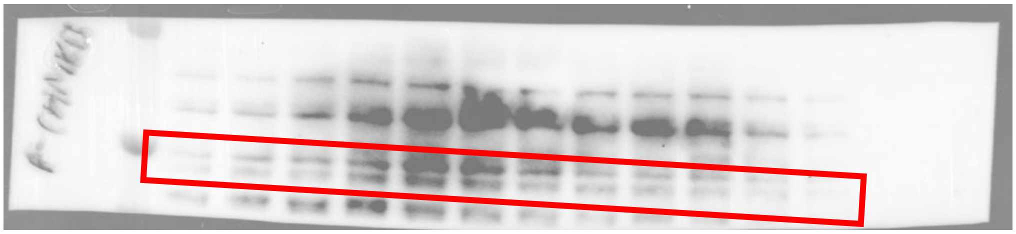

Full and Unedited Blots for Supplemental Figure 5A continued

CAMKII

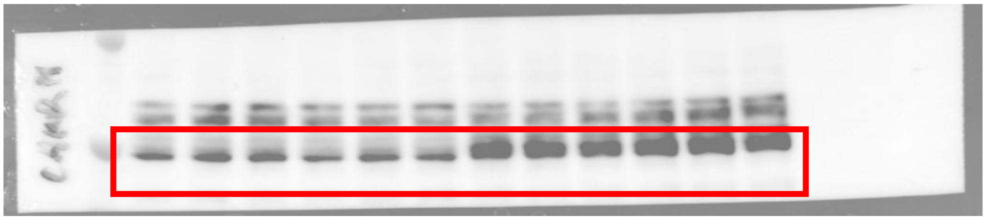

GAPDH

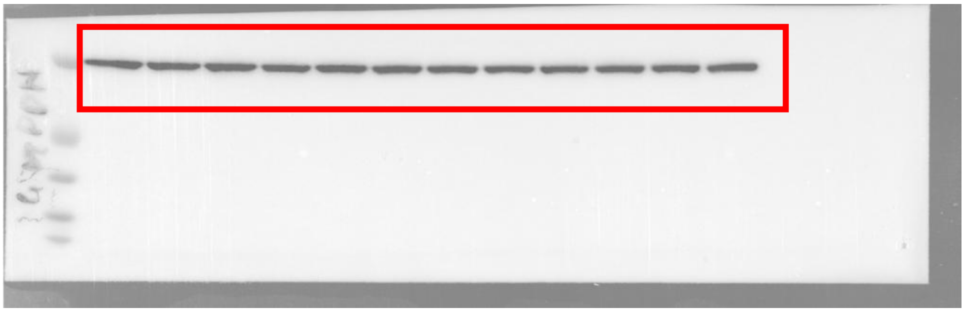

Full and Unedited Blots for Supplemental Figure 5B

HER3 Truncated Knock Down transfected with WT HER3 or ΔI/III HER3
Lanes: WT 0, 10, 30, 60 min ASPN; ΔI/III 0, 10, 30, 60 ASPN
P-HER2

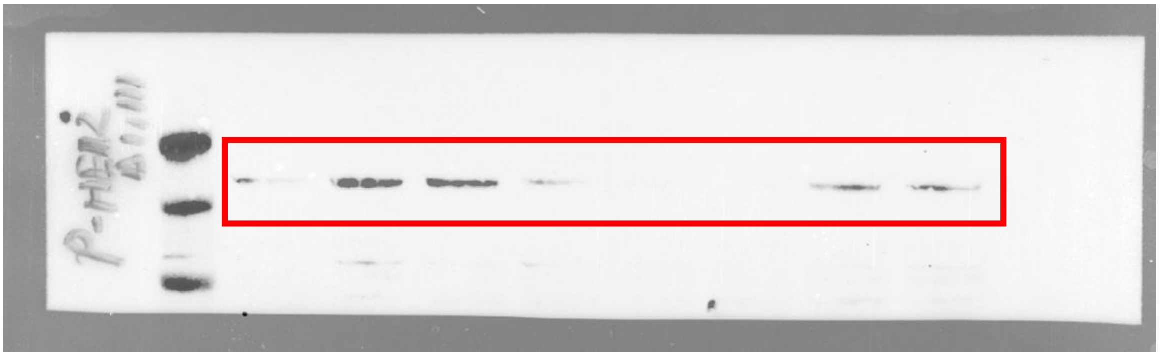

HER2

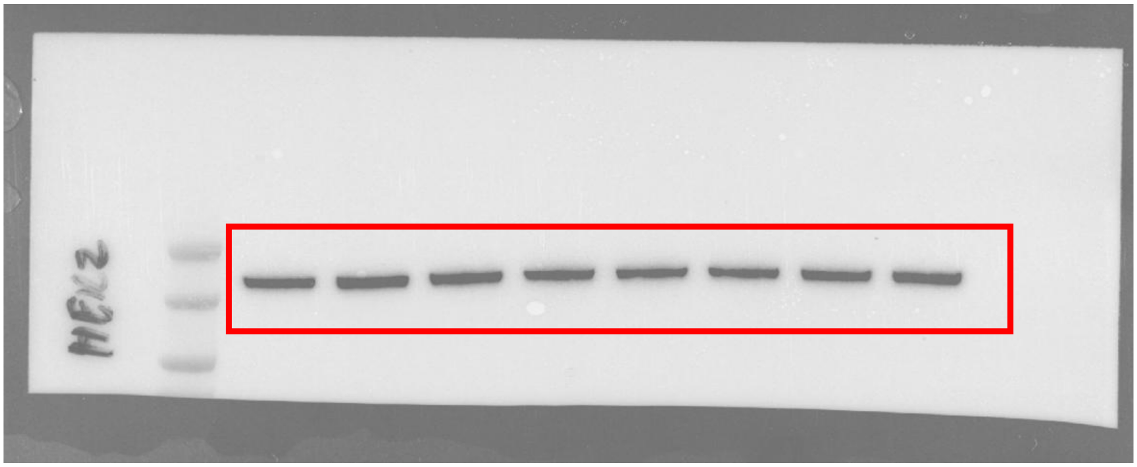

P-HER3

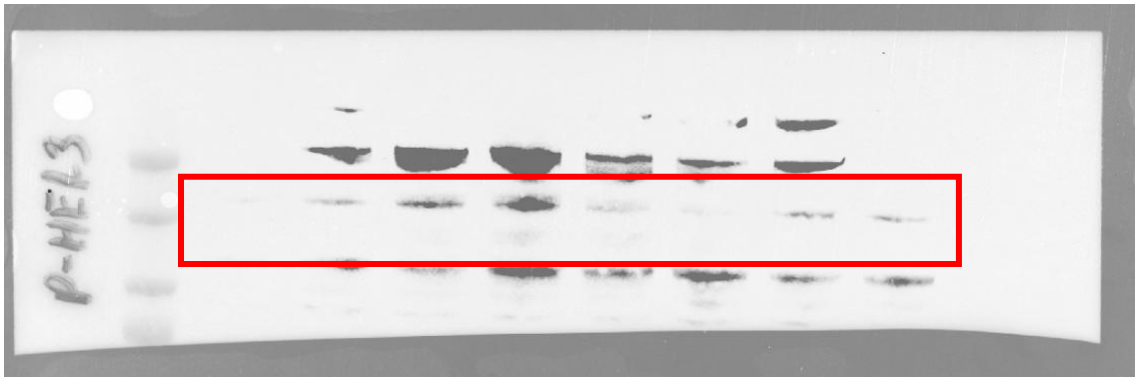

Full and Unedited Blots for Supplemental Figure 5B Continued

HER3

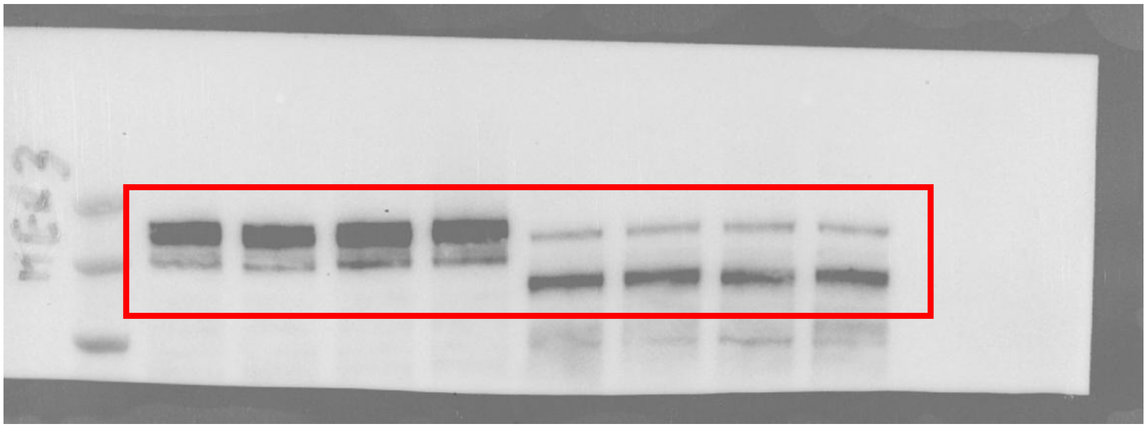

P-EGFR Y845

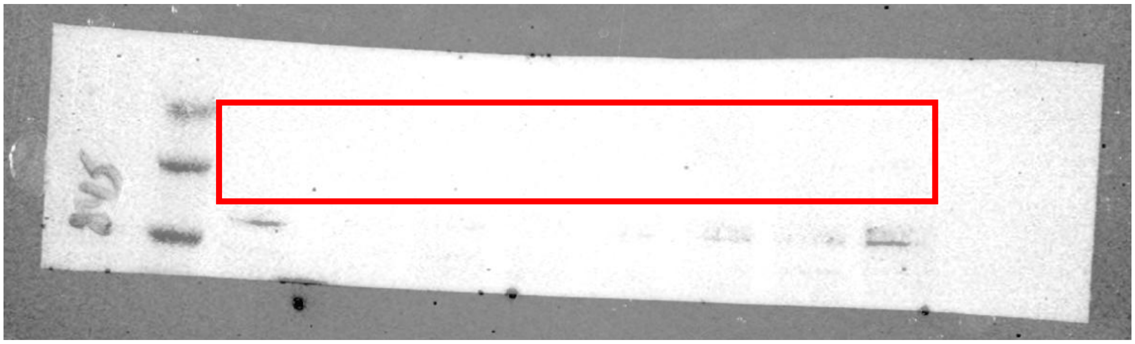

P-EGFR Y1173

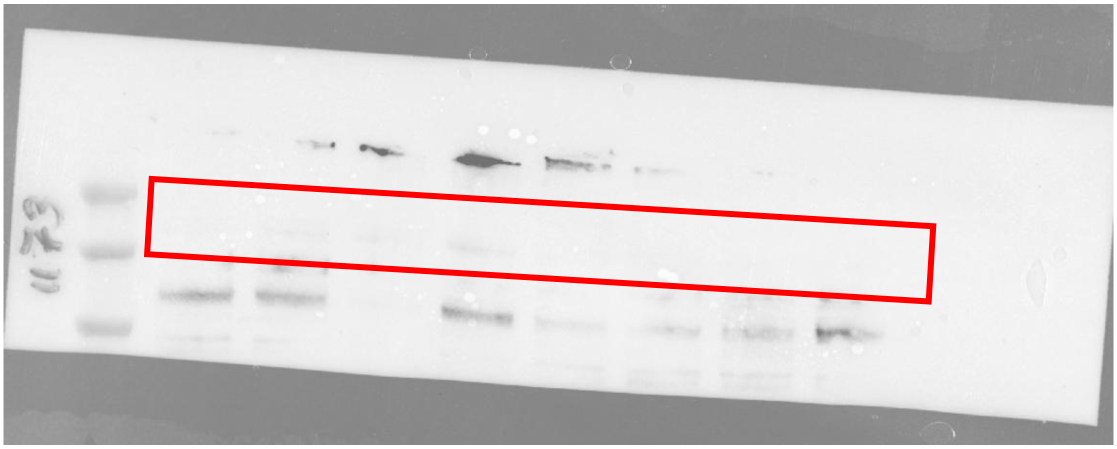

Full and Unedited Blots for Supplemental Figure 5B Continued

EGFR

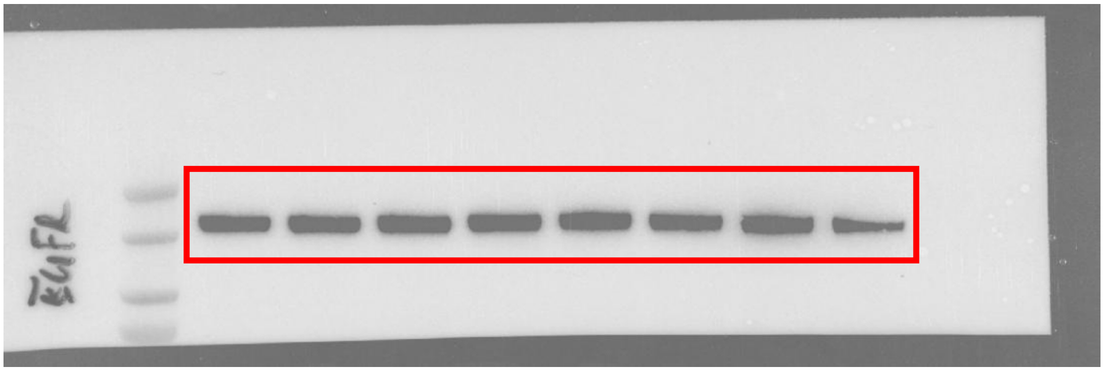

P-AKT

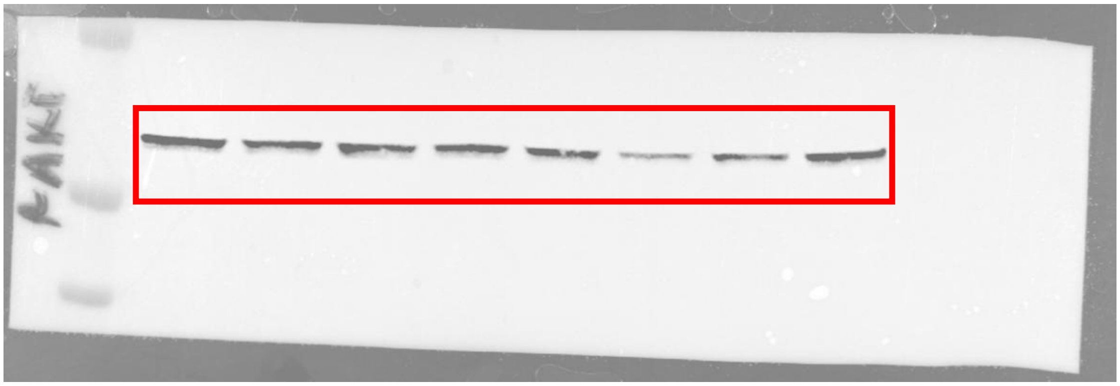

AKT

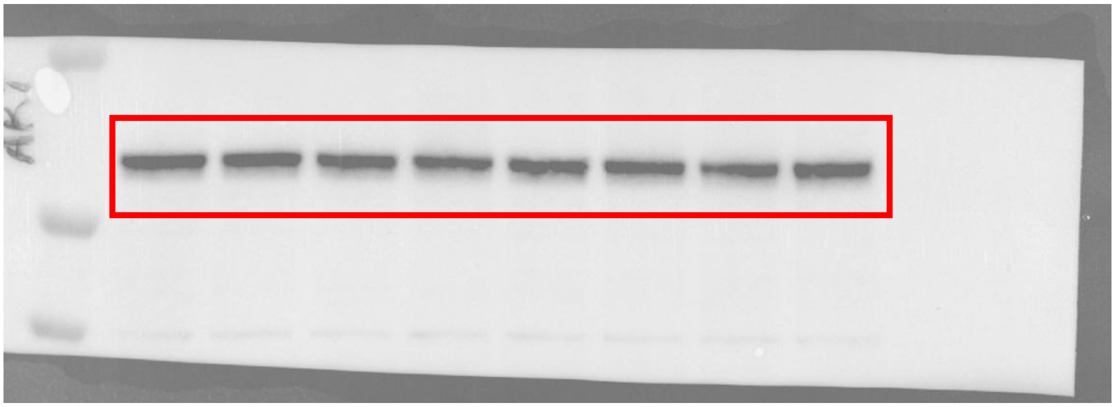

Full and Unedited Blots for Supplemental Figure 5B Continued

P-ERK

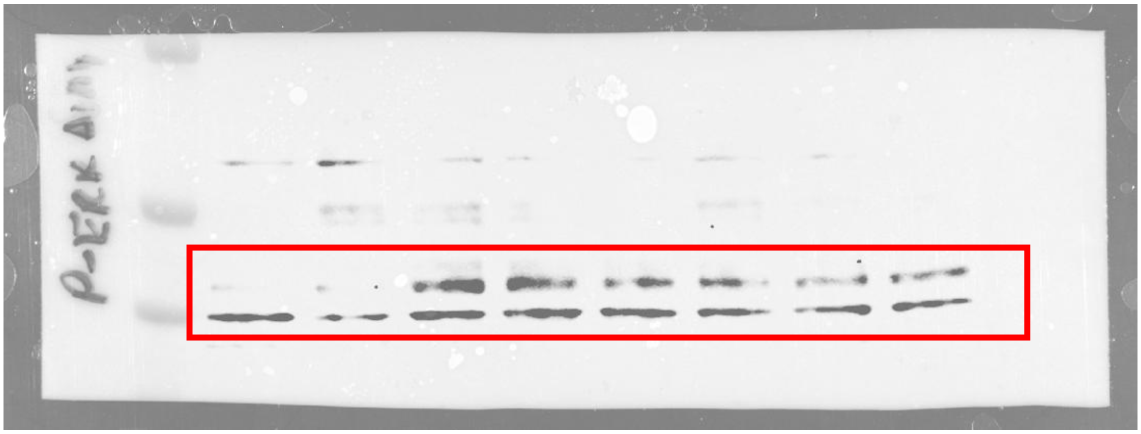

ERK

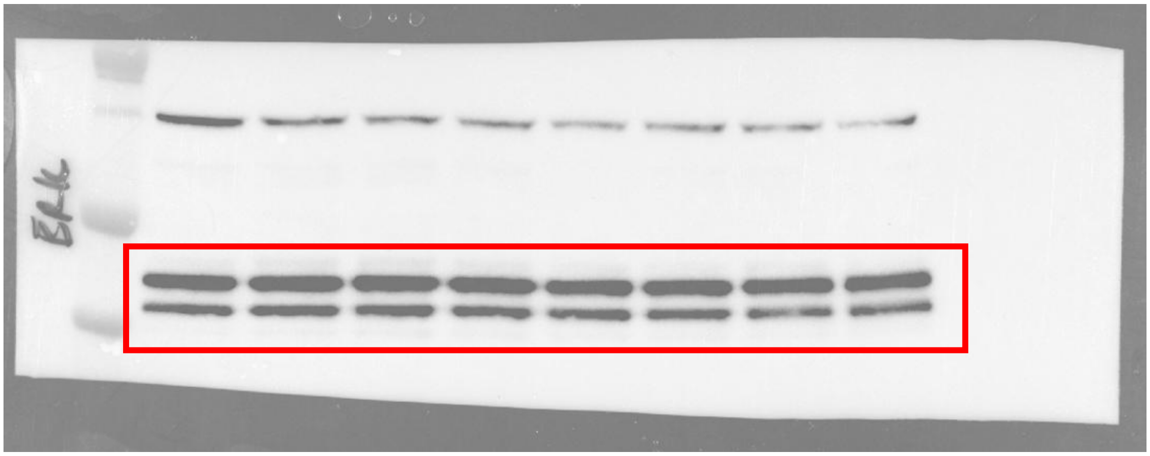

P-PLC

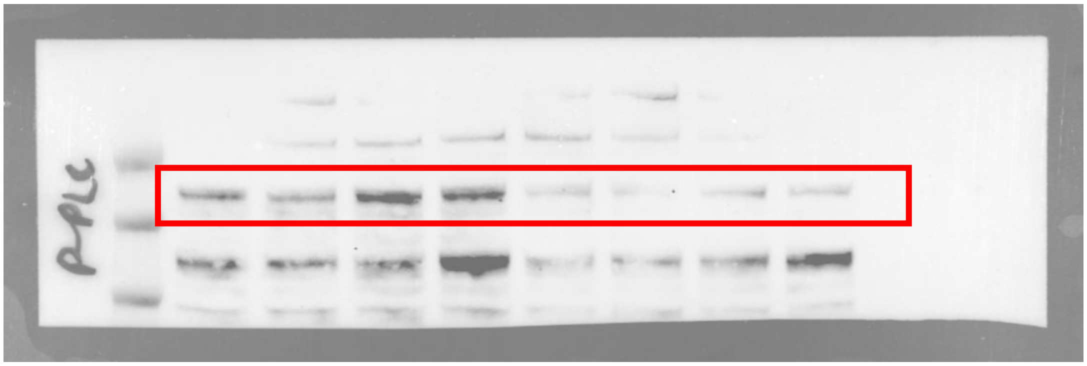

Full and Unedited Blots for Supplemental Figure 5B Continued

PLC

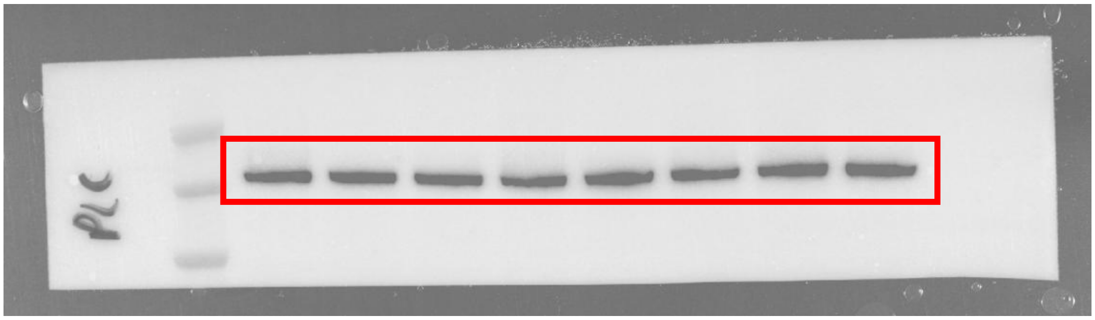

P-CAMKII

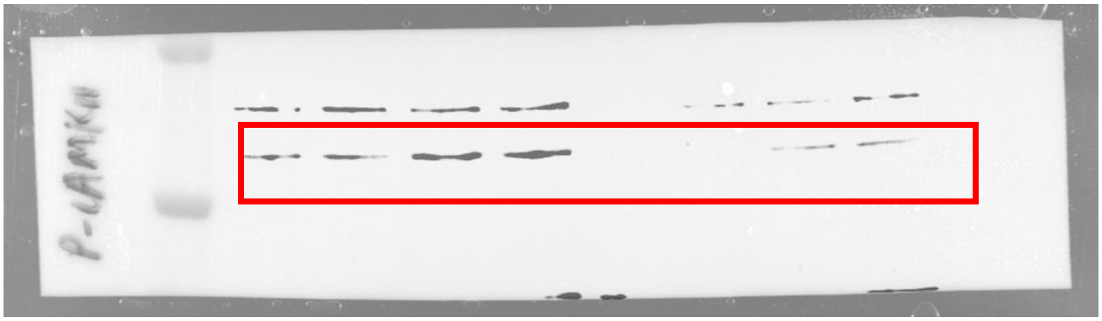

CAMKII

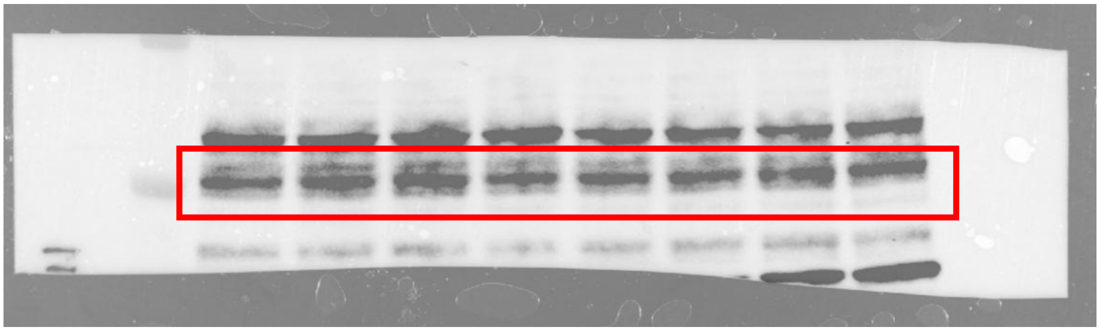

GAPDH

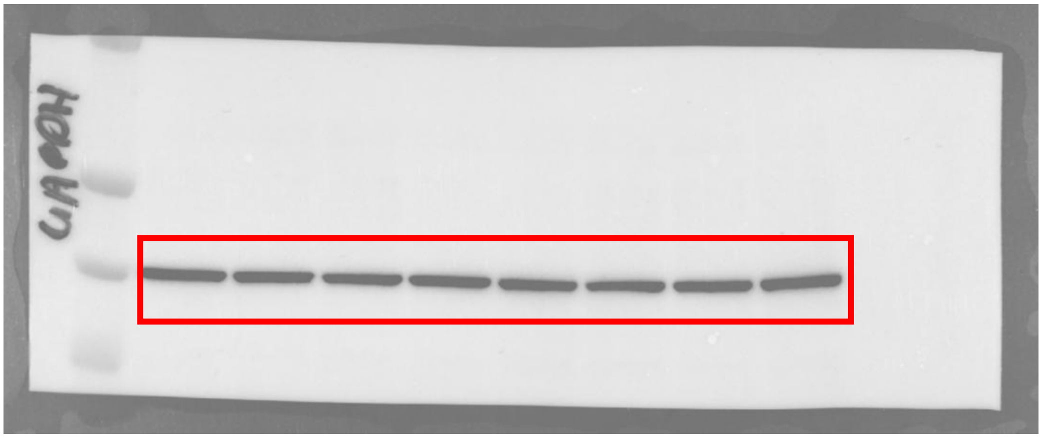

Full and Unedited Blots for Supplemental Figure 5C

HER2 TWT C9, HER2 KO D4

Lanes: TWT 0, 5, 10, 15, 30, 60, KD 0, 5, 10, 15, 30, 60 unless noted

P-HER2

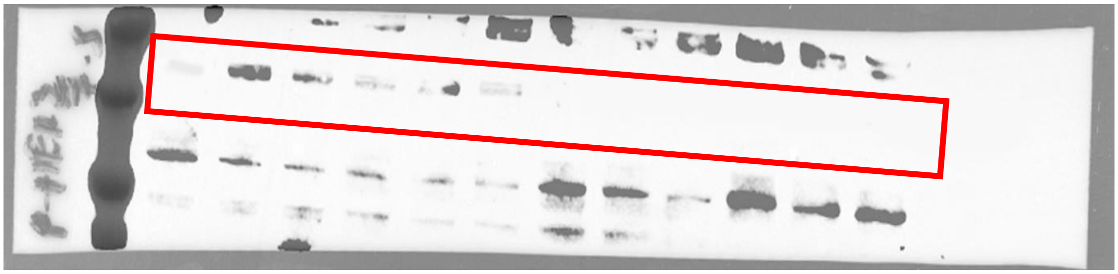

HER2

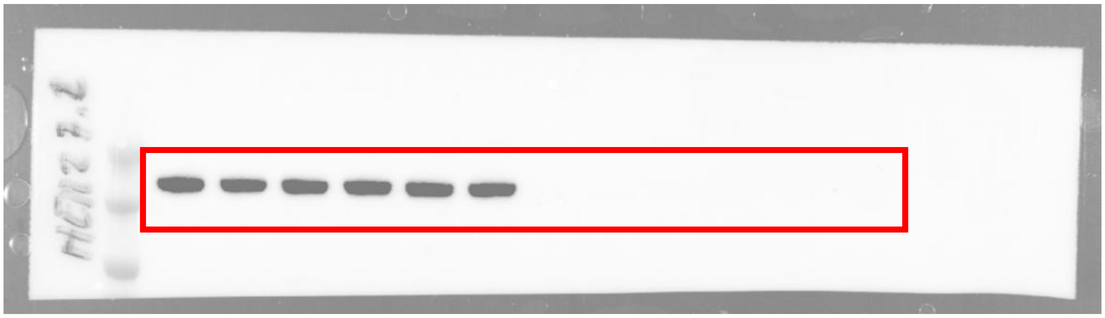

P-HER3

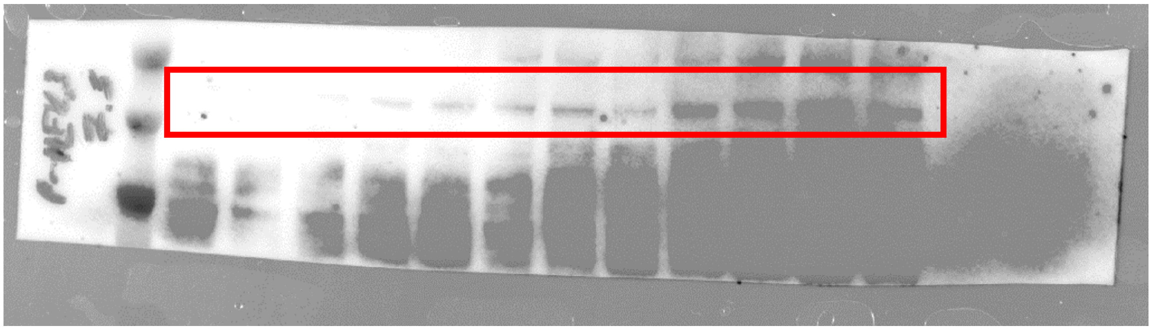

Full and Unedited Blots for Supplemental Figure 5C

HER3

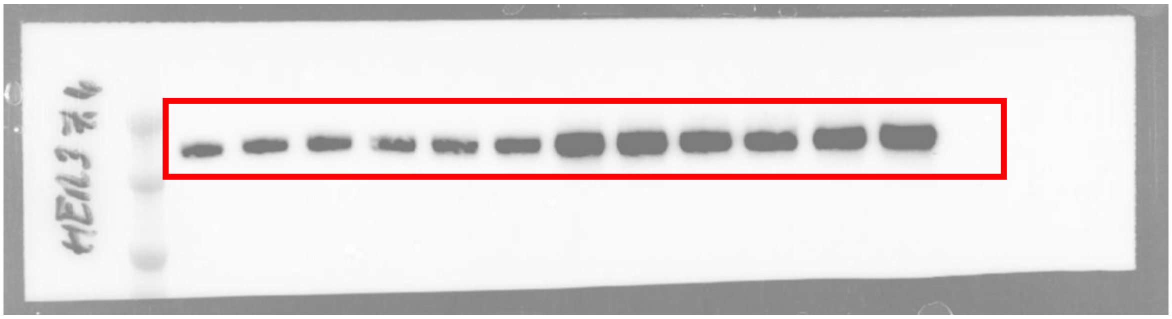

Lanes: EGF ctrl, TWT 0, 5, 10, 15, 30, 60, KO 0, 5, 10, 15, 30, 60
P-EGFR Y845

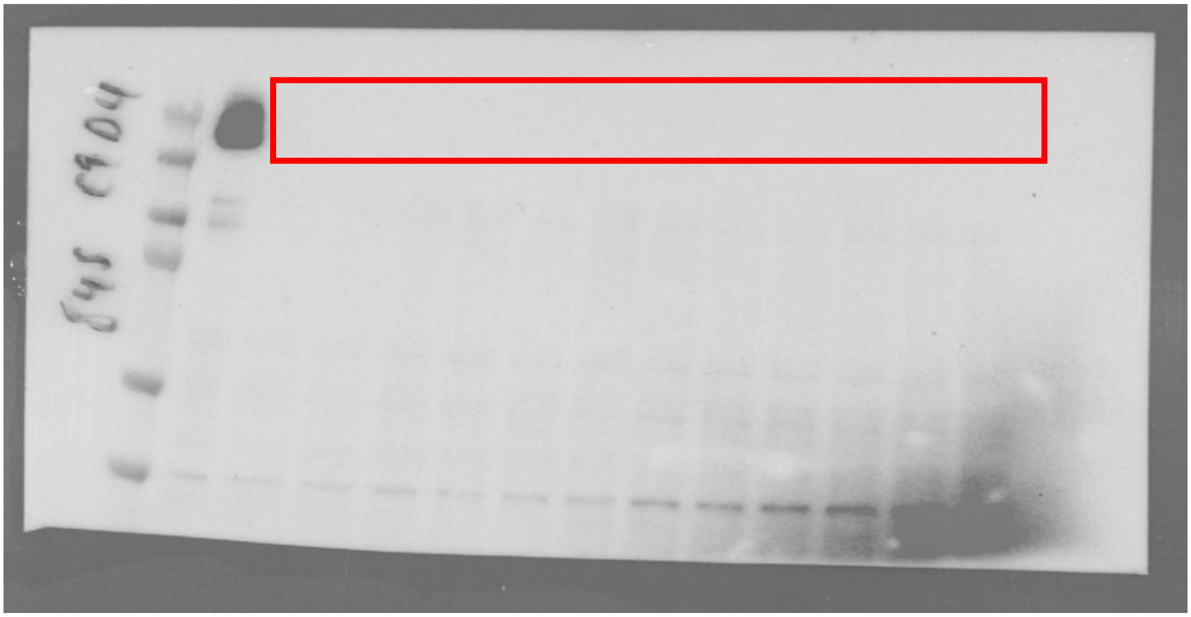

Full and Unedited Blots for Supplemental Figure 5C Continued

Lanes: EGF ctrl, TWT 0, 5, 10, 15, 30, 60, KO 0, 5, 10, 15, 30, 60
P-EGFR Y1173

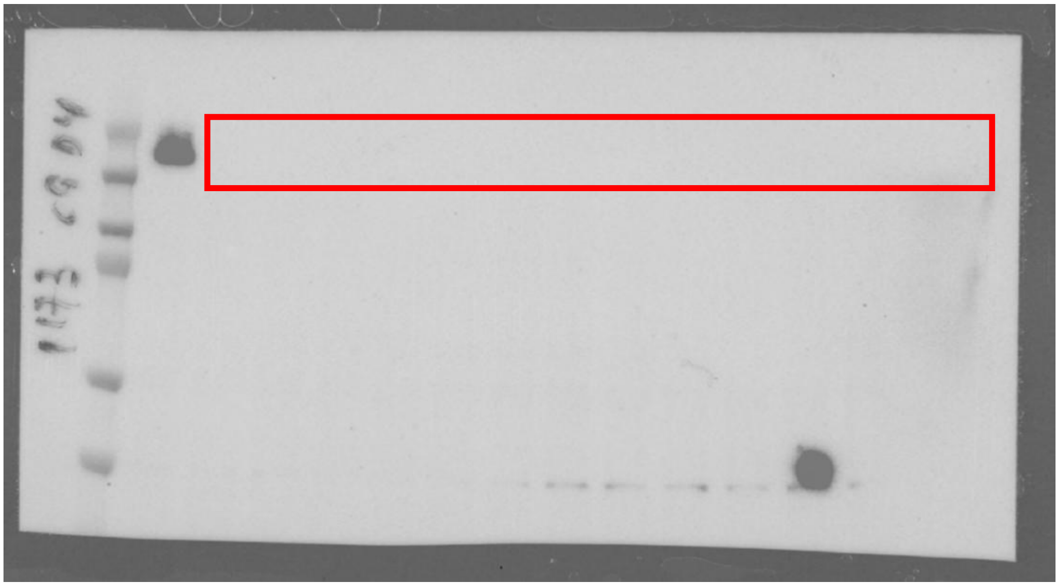

Lanes: EGF ctrl, TWT 0, 5, 10, 15, 30, 60, KO 0, 5, 10, 15, 30, 60
EGFR

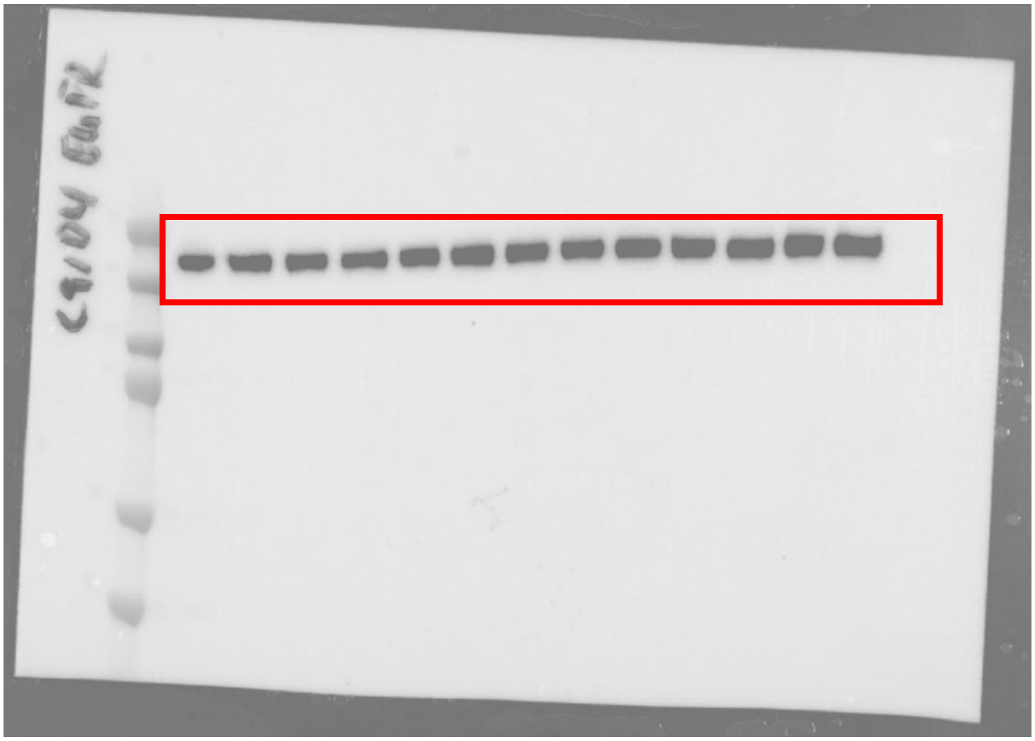

Full and Unedited Blots for Supplemental Figure 5C

P-AKT

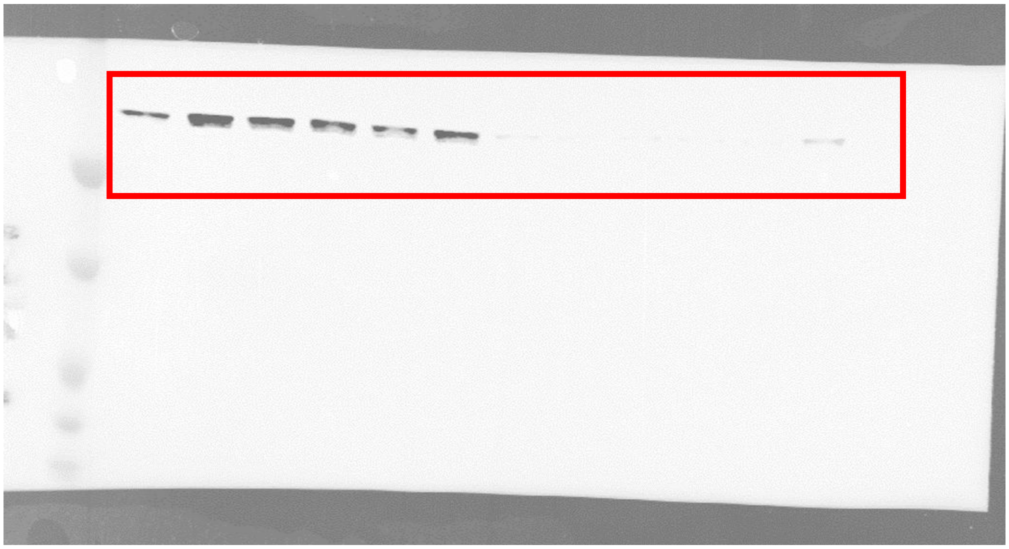

AKT

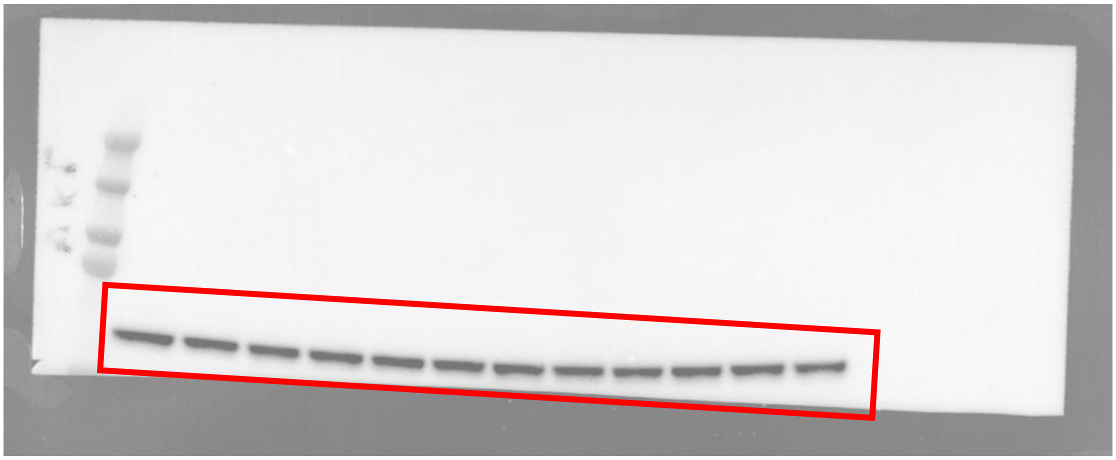

P-ERK

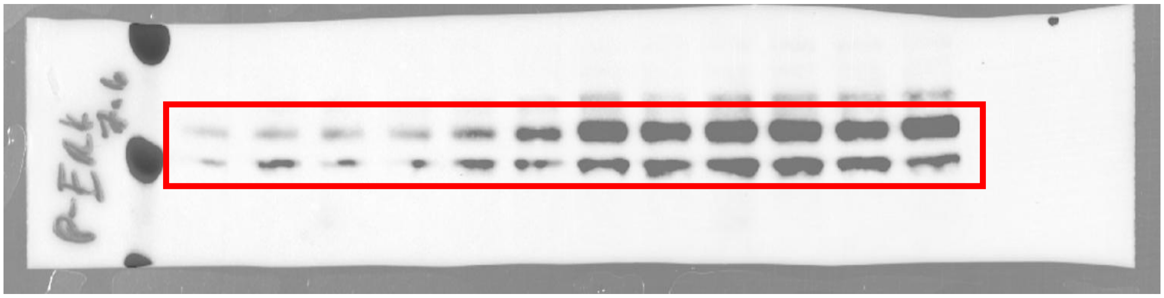

Full and Unedited Blots for Supplemental Figure 5C

ERK

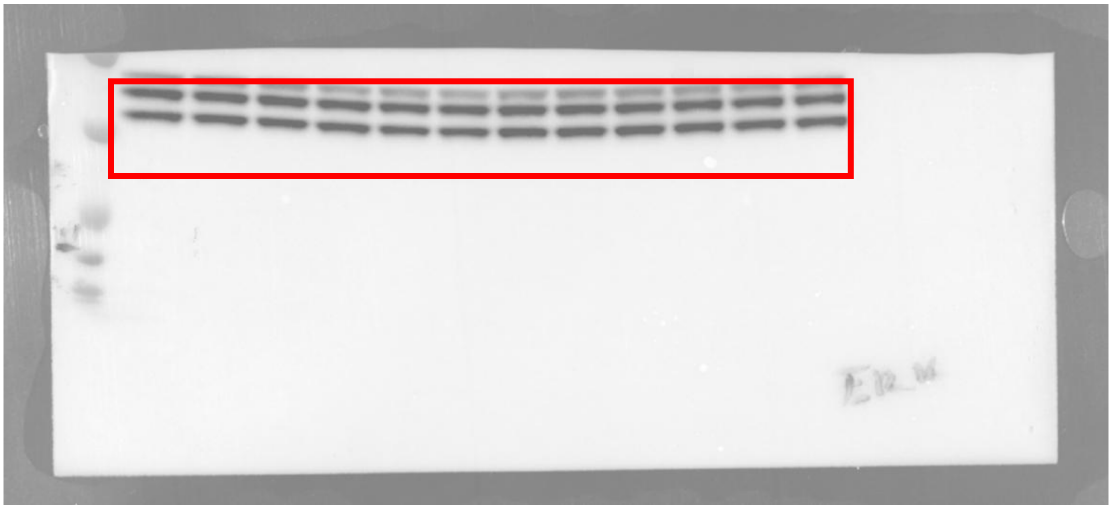

P-PLC

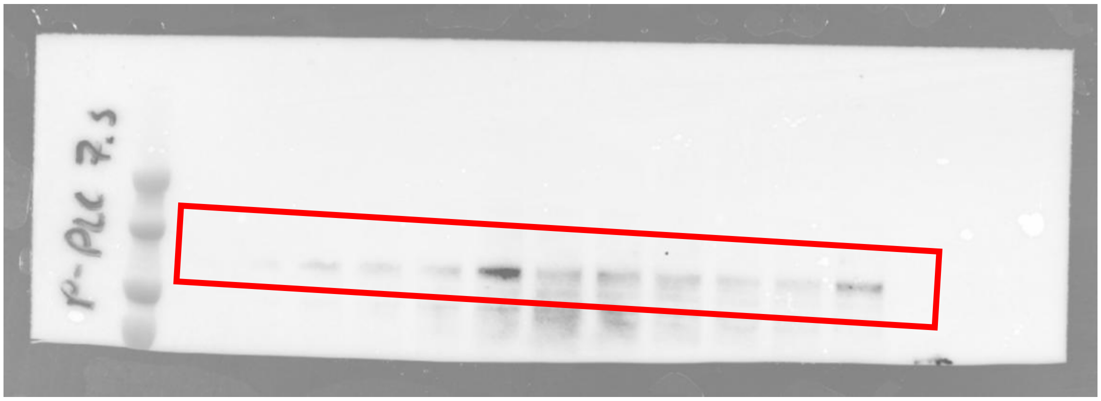

PLC

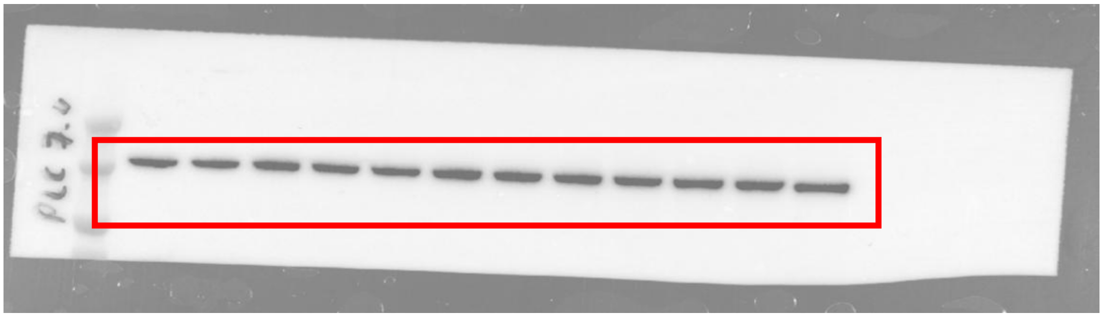

Full and Unedited Blots for Supplemental Figure 5C

P-CAMKII

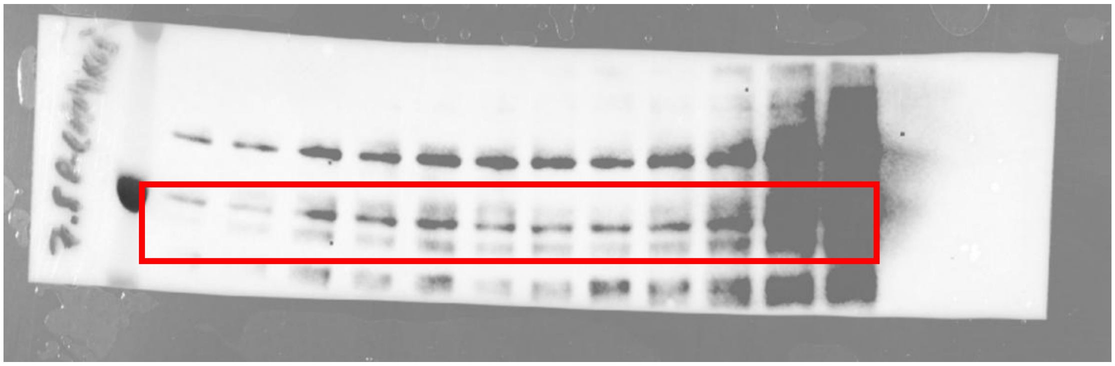

CAMKII

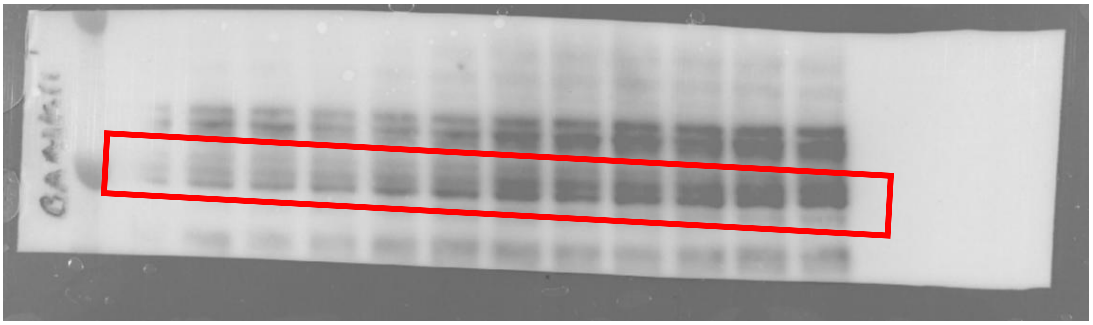

GAPDH

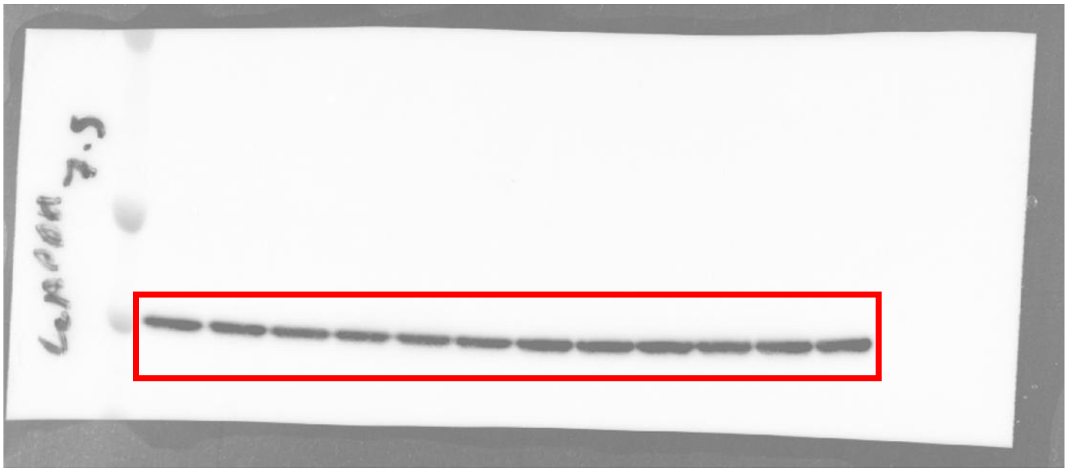

Full and Unedited Blots for Supplemental Figure 5C Continued

HER2 TWT D12, HER2 KO D5

Lanes: TWT 0, 5, 10, 15, 30, 60, KD 0, 5, 10, 15, 30, 60 unless noted

P-HER2

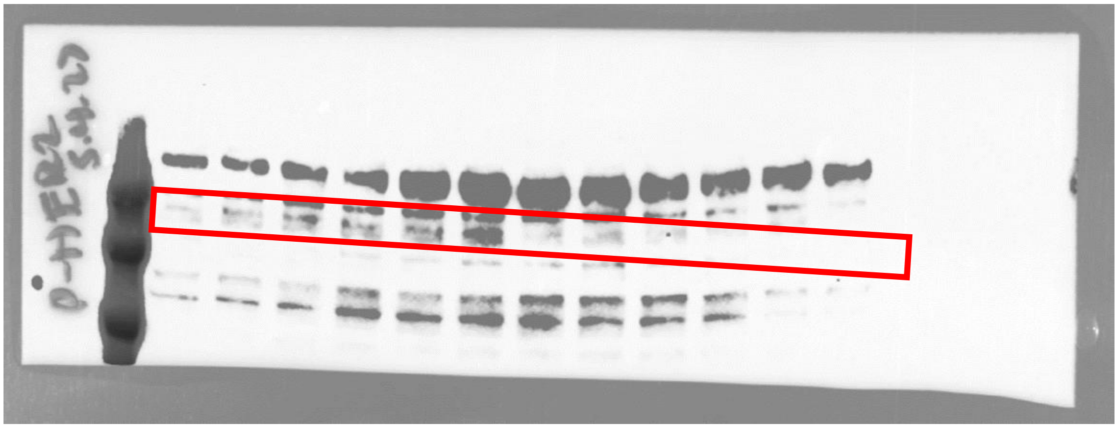

HER2

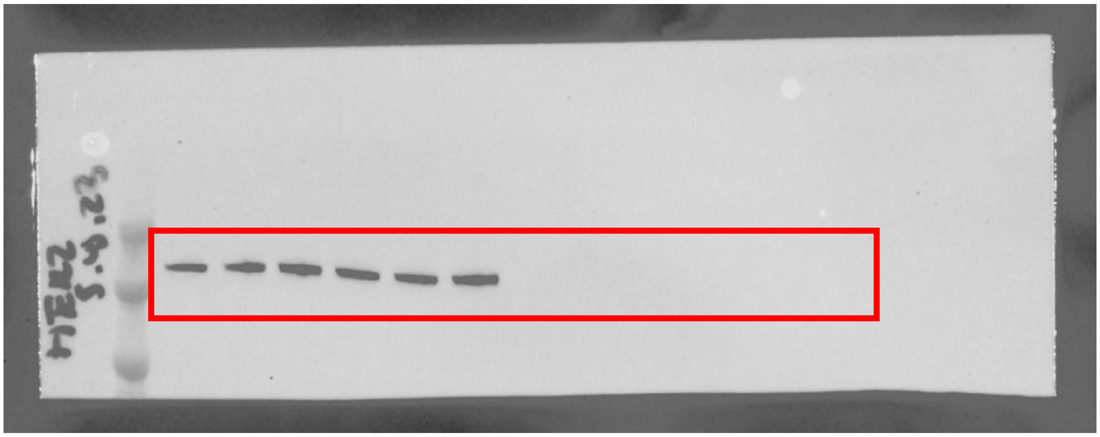

P-HER3

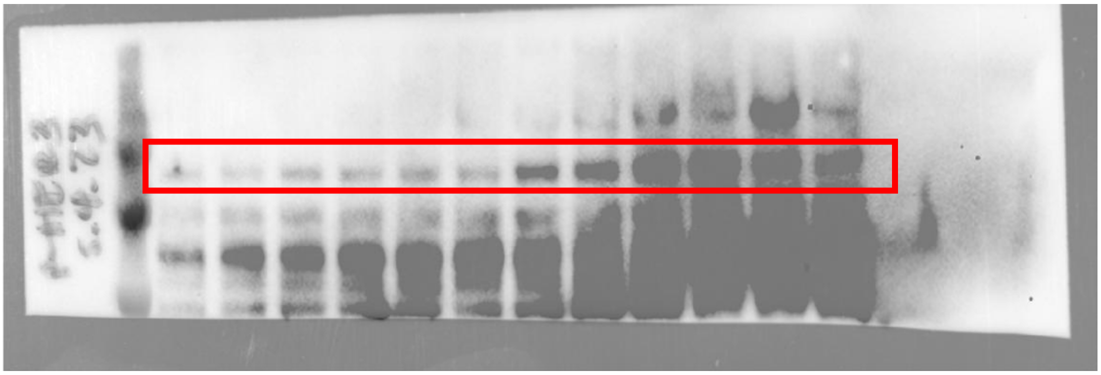

Full and Unedited Blots for Supplemental Figure 5C Continued

HER3

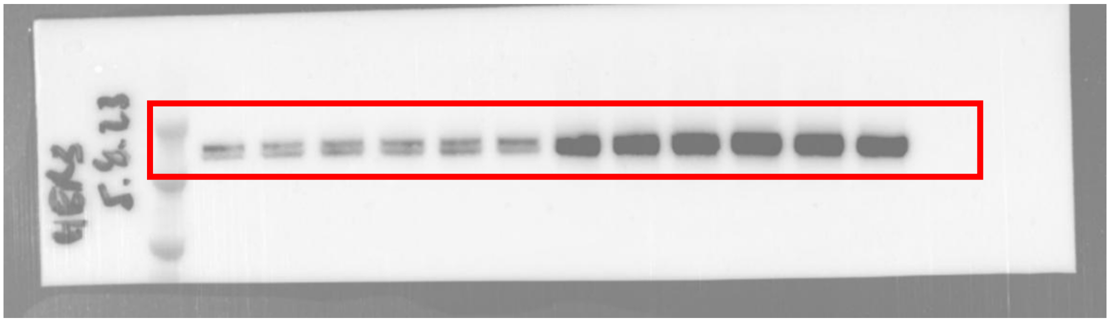

Lanes: EGF ctrl, TWT 0, 5, 10, 15, 30, 60, KO 0, 5, 10, 15, 30, 60

P-EGFR Y845

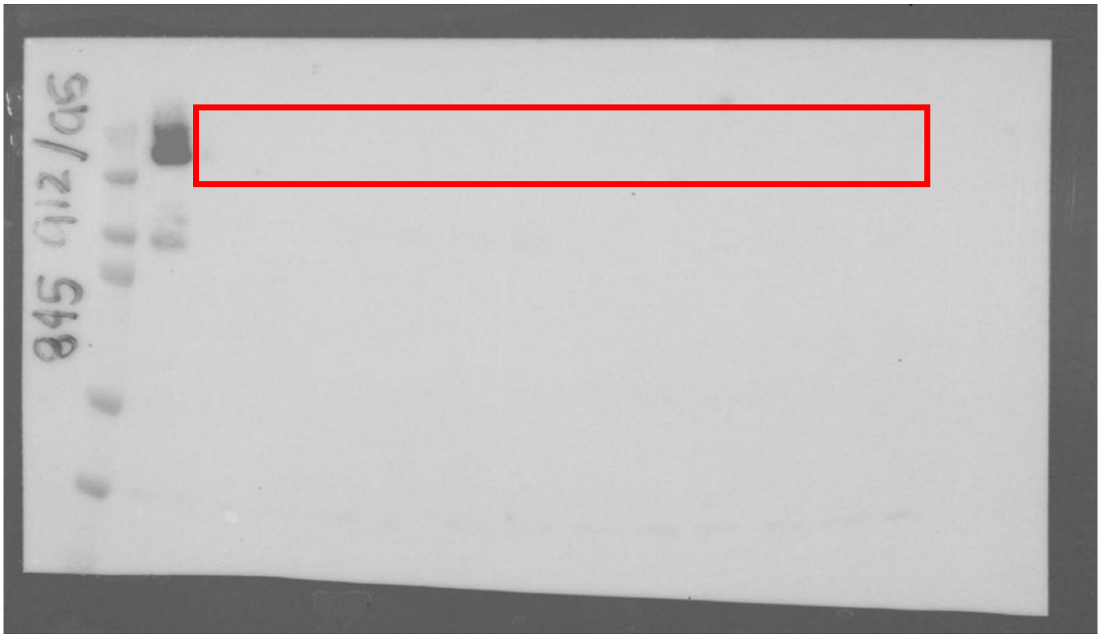

Full and Unedited Blots for Supplemental Figure 5C Continued

Lanes: EGF ctrl, TWT 0, 5, 10, 15, 30, 60, KO 0, 5, 10, 15, 30, 60

P-EGFR Y1173

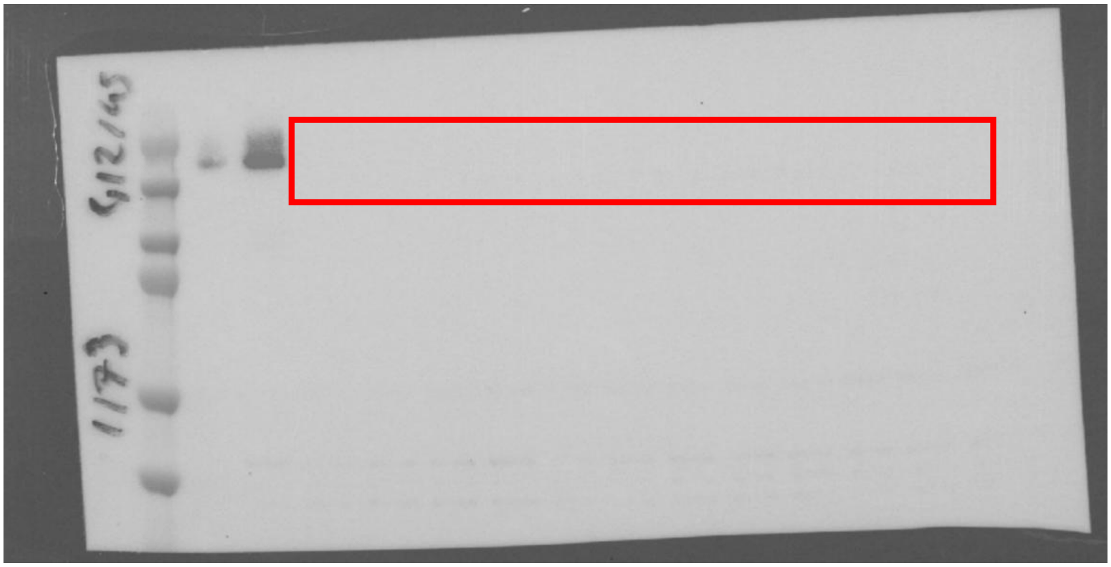

Lanes: EGF ctrl, TWT 0, 5, 10, 15, 30, 60, KO 0, 5, 10, 15, 30, 60

EGFR

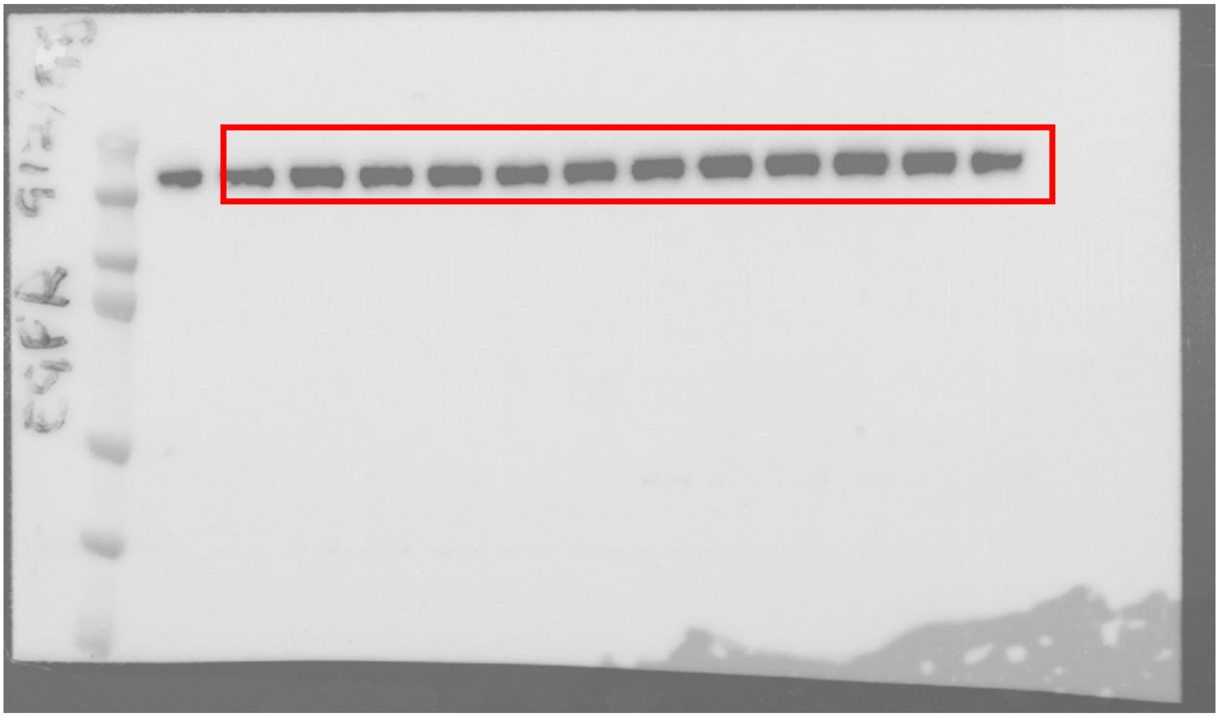

Full and Unedited Blots for Supplemental Figure 5C Continued

P-AKT

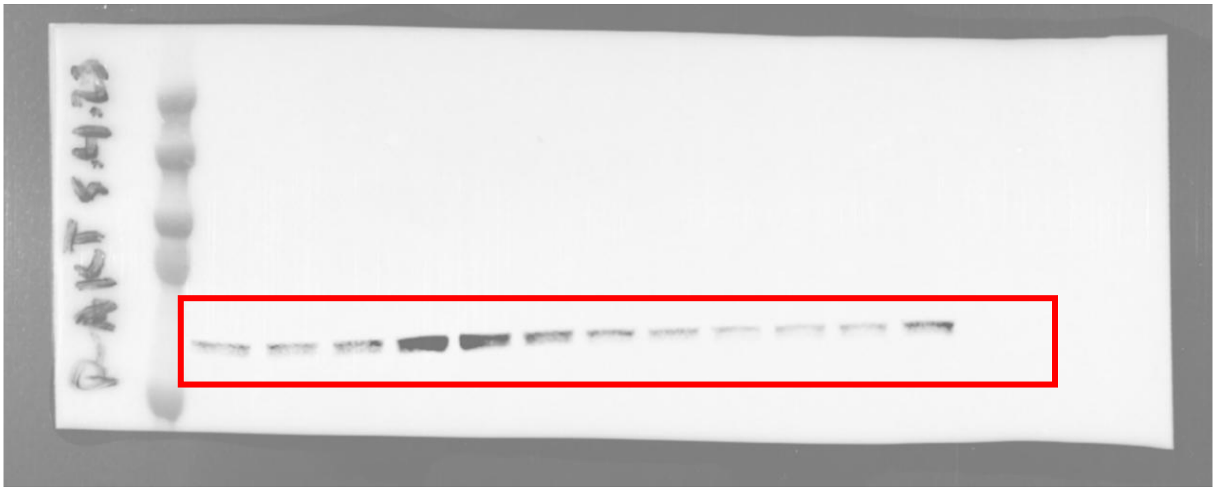

AKT

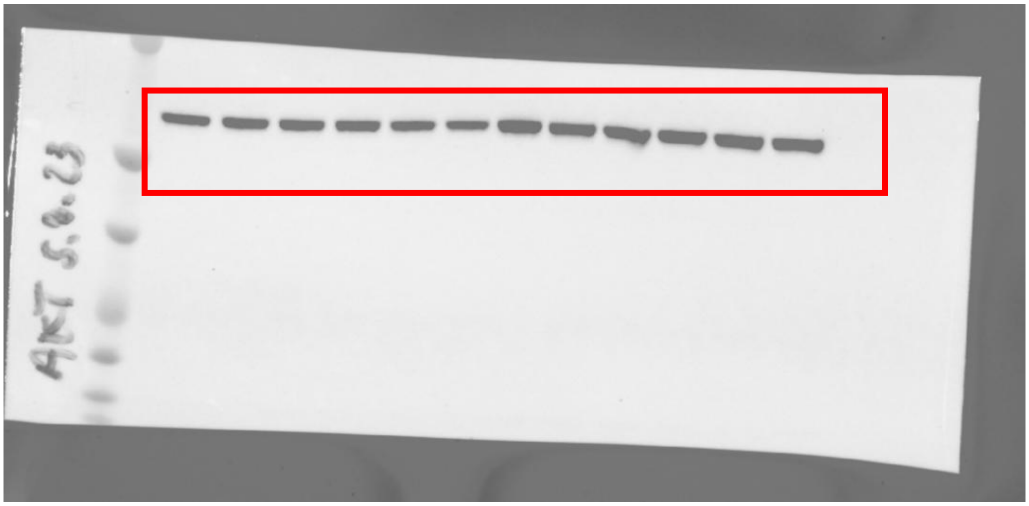

P-ERK

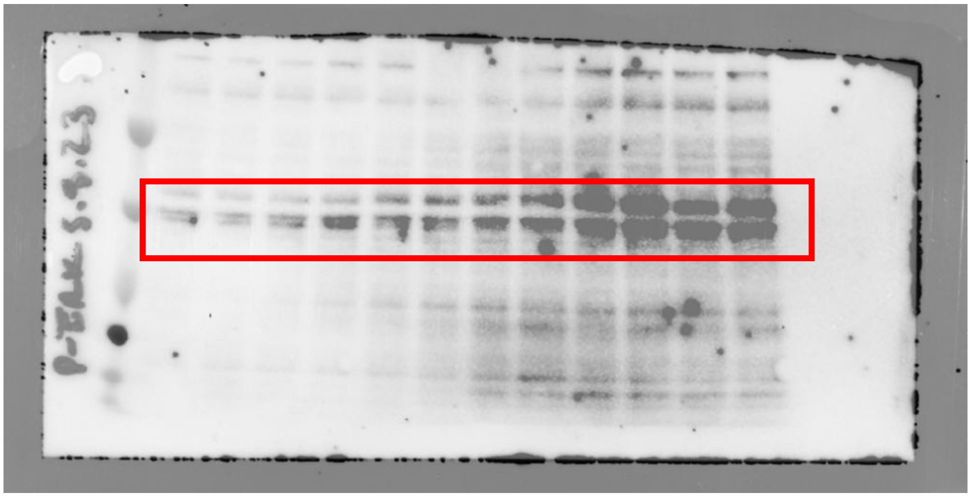

Full and Unedited Blots for Supplemental Figure 5C Continued

ERK

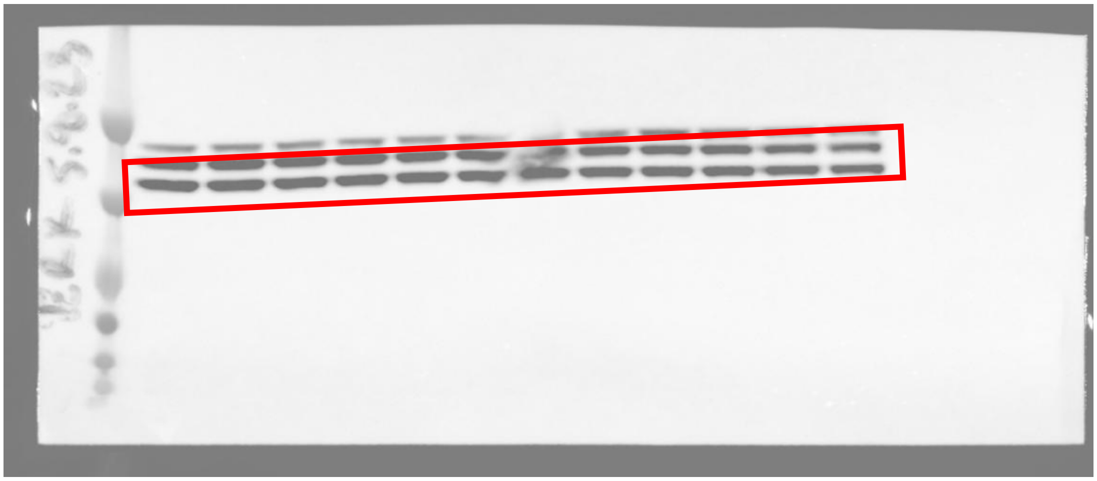

P-PLC

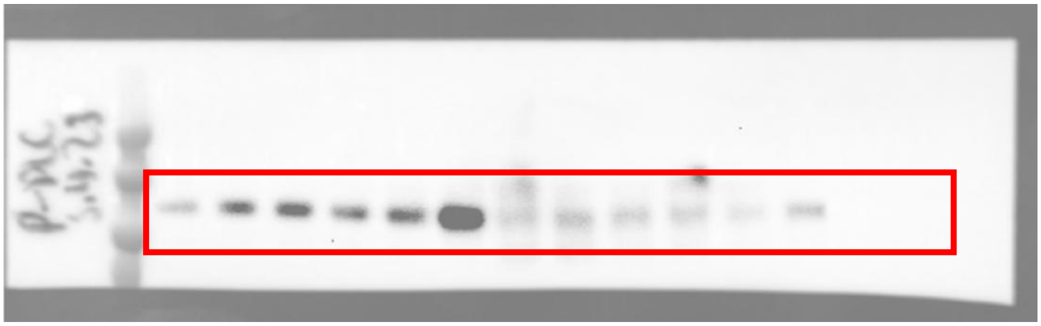

PLC

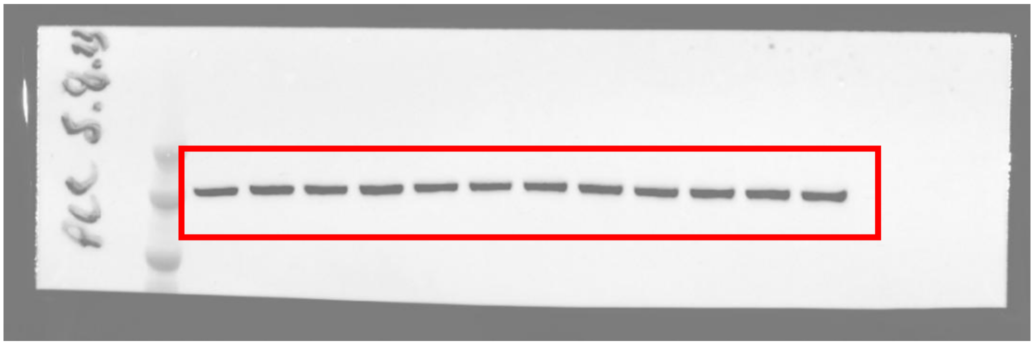

Full and Unedited Blots for Supplemental Figure 5C Continued

P-CAMKII

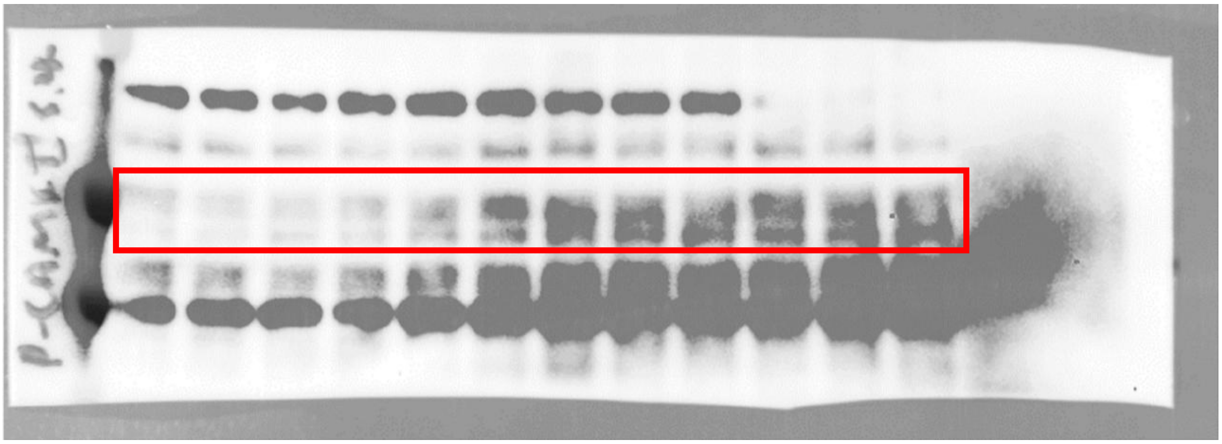

CAMKII

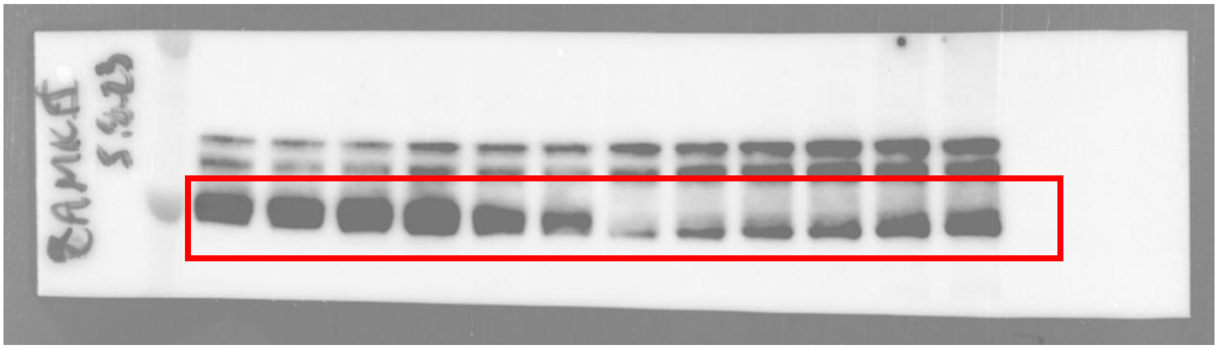

GAPDH

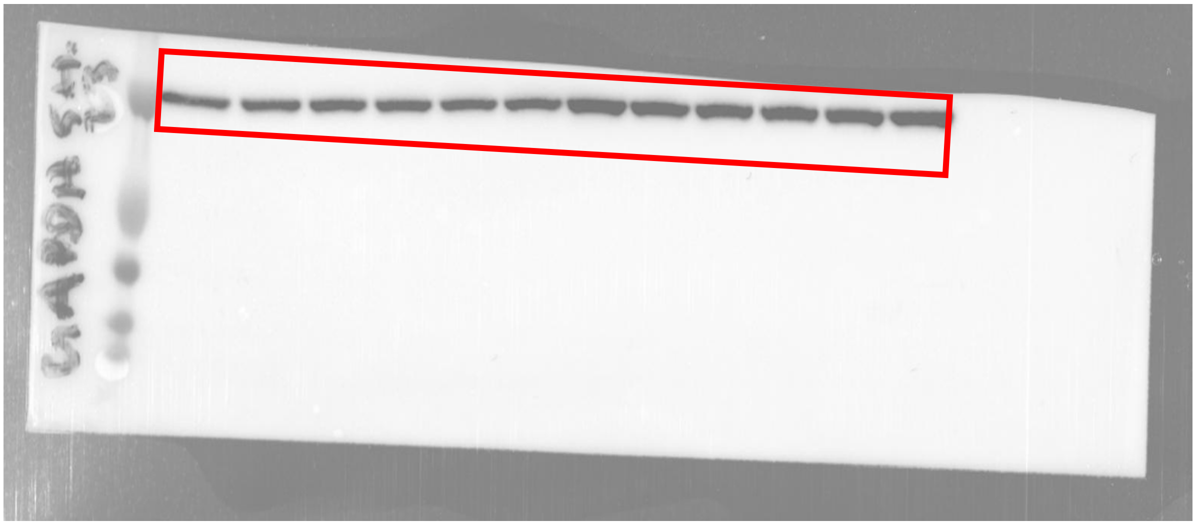

Full and Unedited Blots for Supplemental Figure 9A

PC3+rmASPN

Lanes: 0, 2, 5, 10, 15, 30, 60, 120 min ASPN unless noted

P-HER2

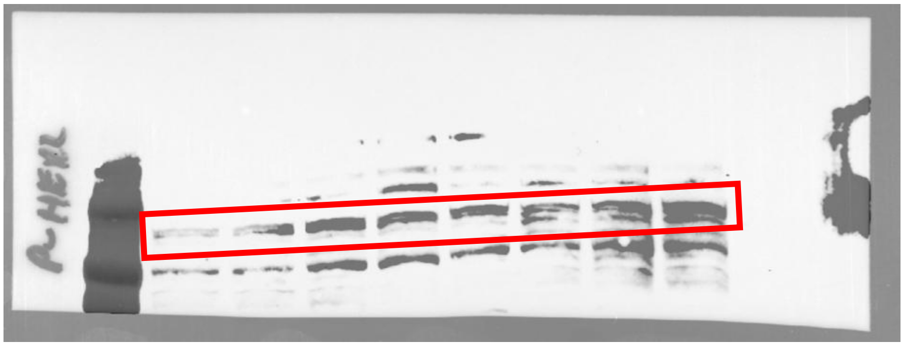

HER2

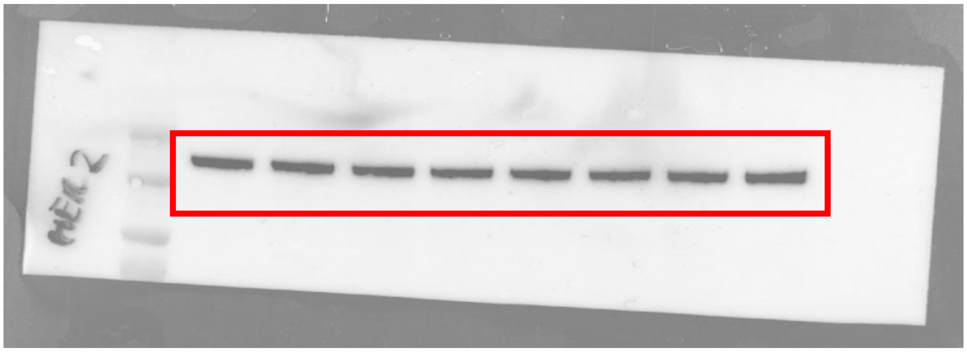

P-HER3

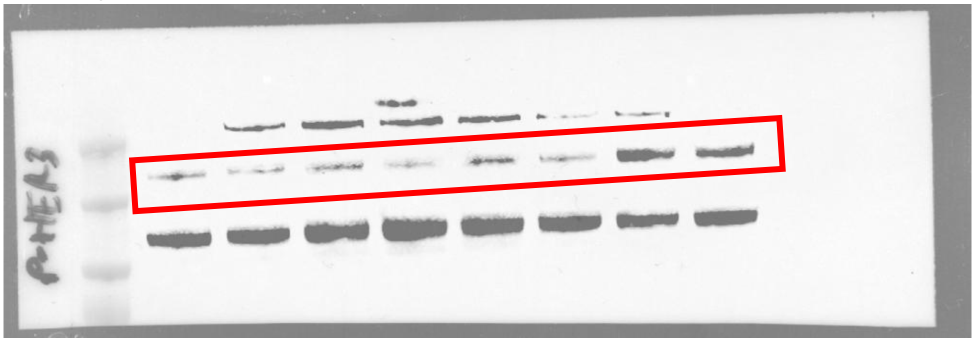

HER3

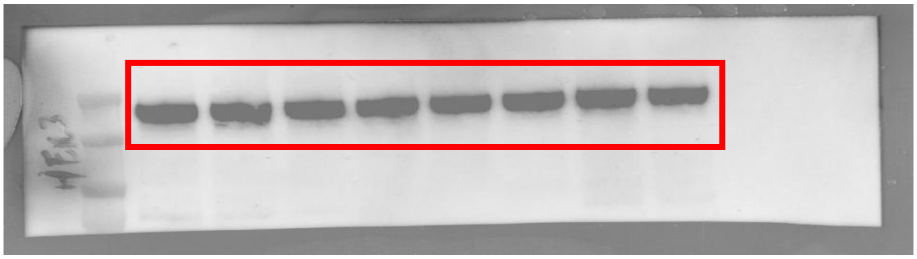

Full and Unedited Blots for Supplemental Figure 9A Continued

P-EGFR Y845

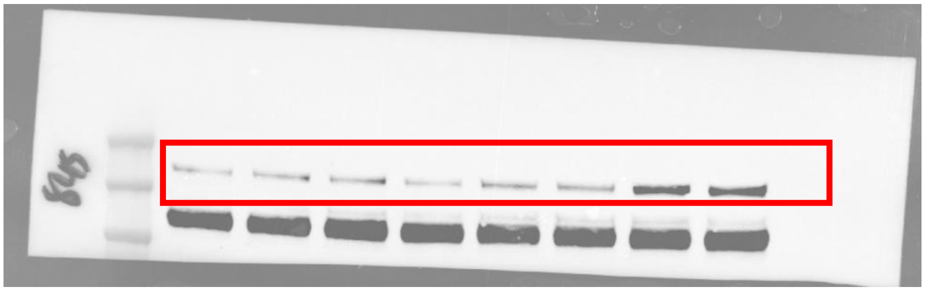

P-EGFR Y1173

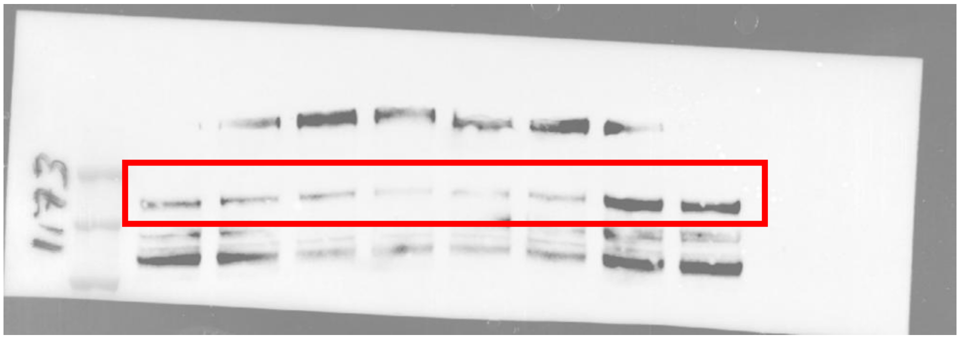

EGFR

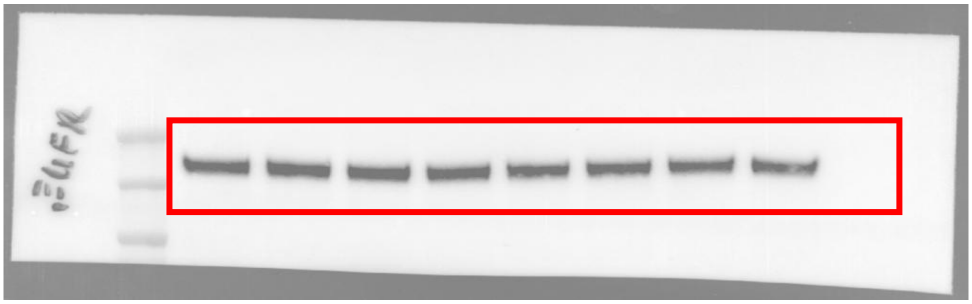

P-AKT

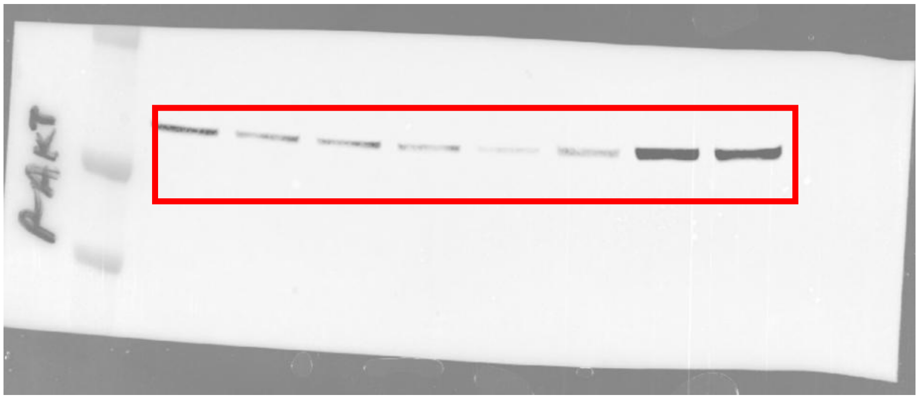

Full and Unedited Blots for Supplemental Figure 9A Continued

AKT

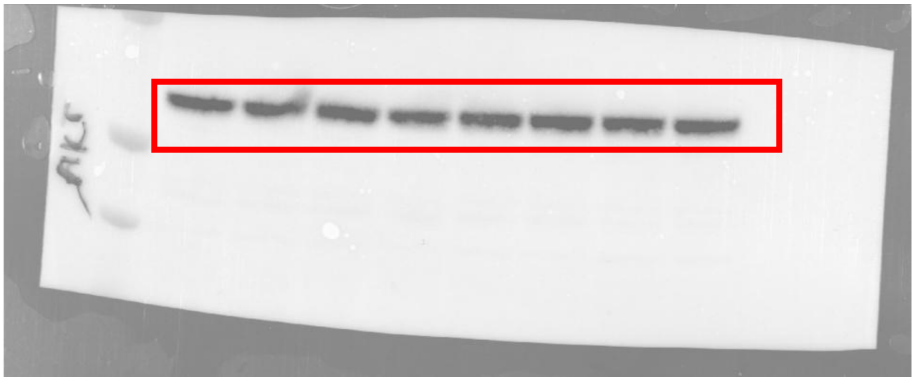

P-ERK

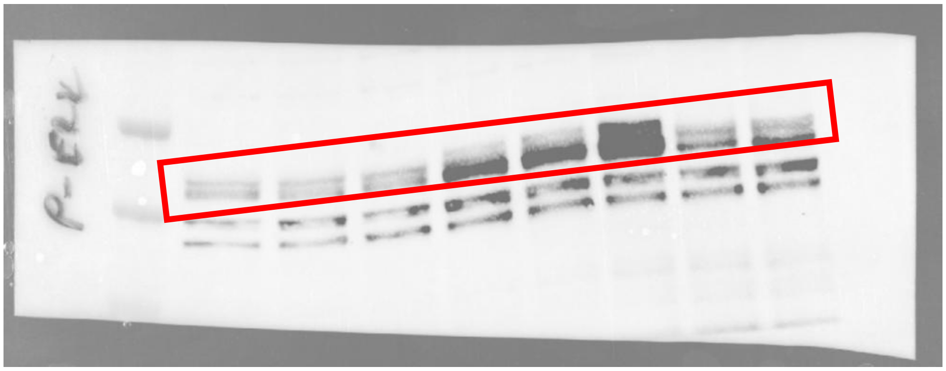

ERK

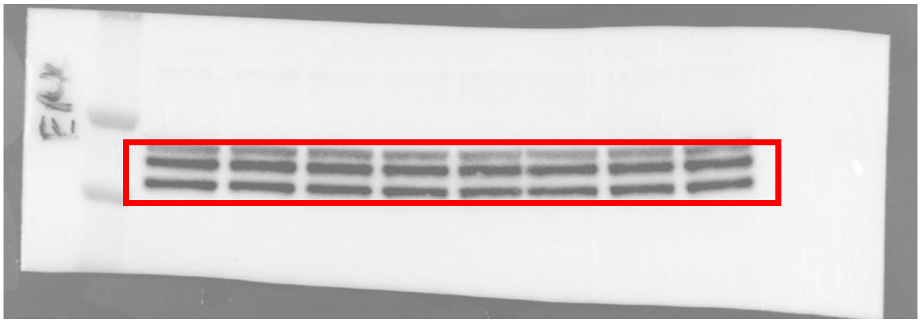

P-PLC

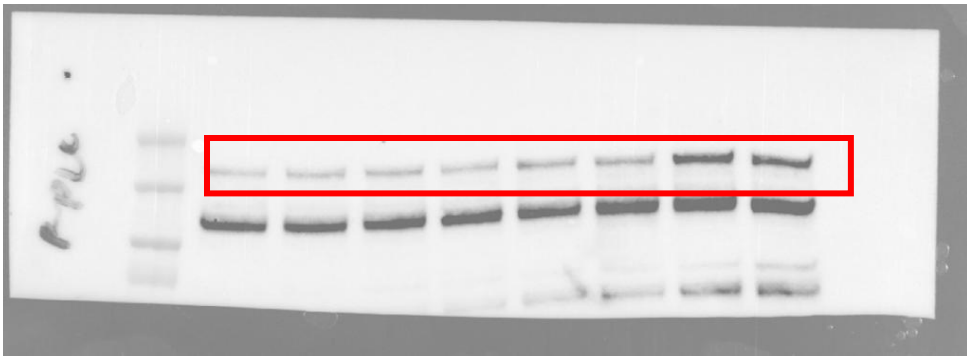

Full and Unedited Blots for Supplemental Figure 9A Continued

PLC

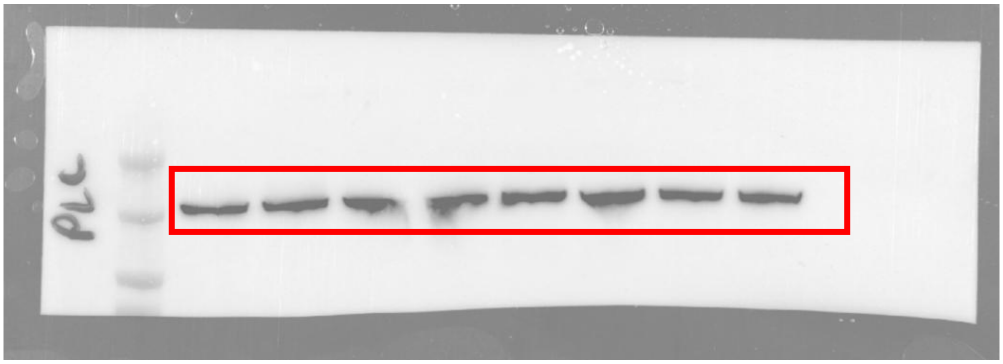

P-CAMKII

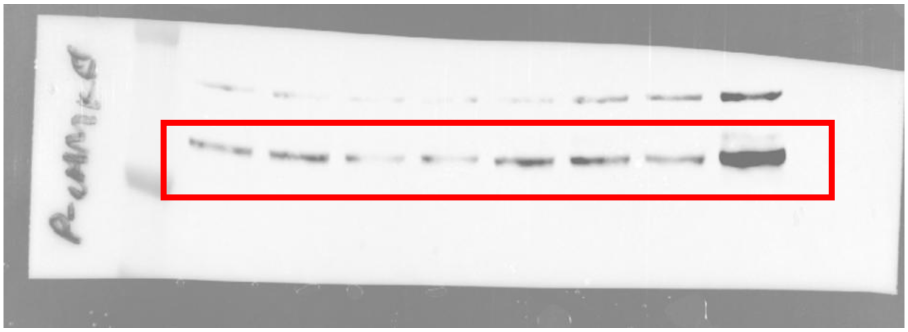

CAMKII

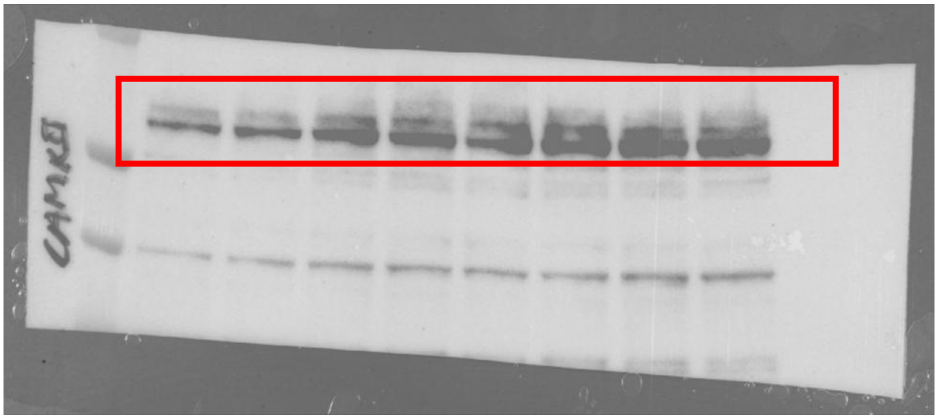

GAPDH

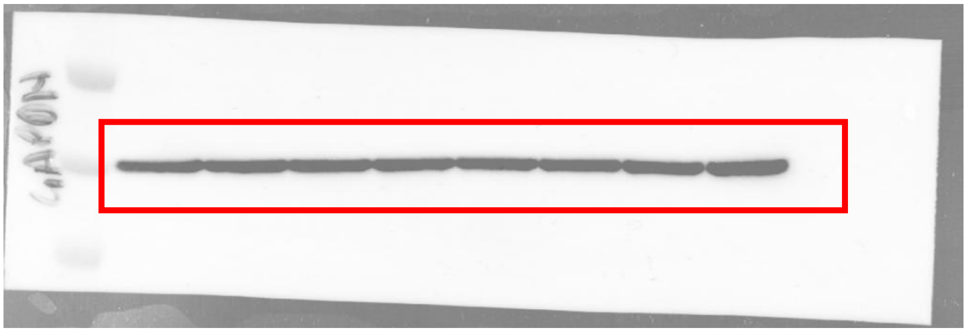

Full and Unedited Blots for Supplemental Figure 9B

PC3+rmASPN+Tucatinib

Lanes: ASPN 0, 5, 10, 15, 30, 60, ASPN+Tuc 0, 5, 10, 15, 30, 60 unless noted

P-HER2

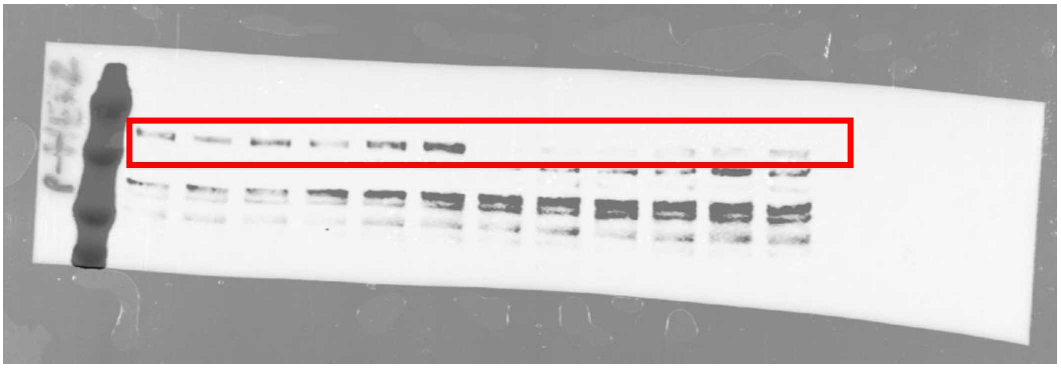

HER2

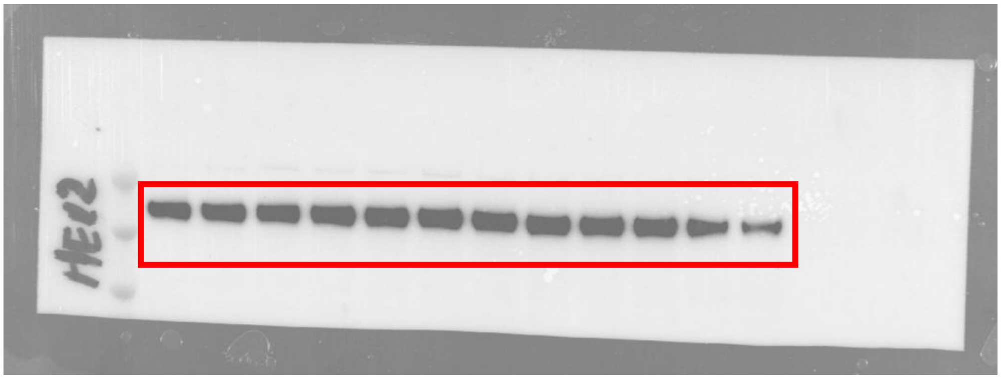

P-HER3

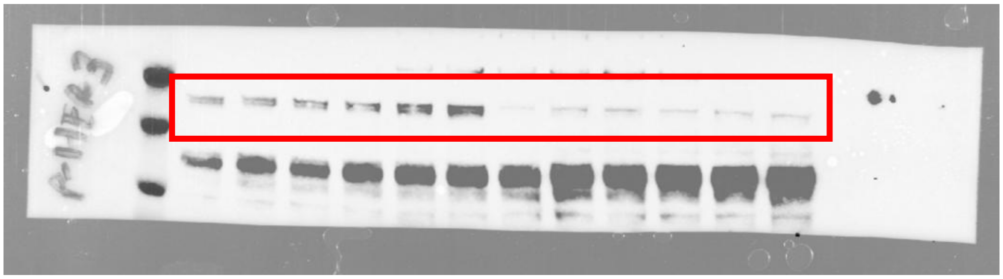

HER3

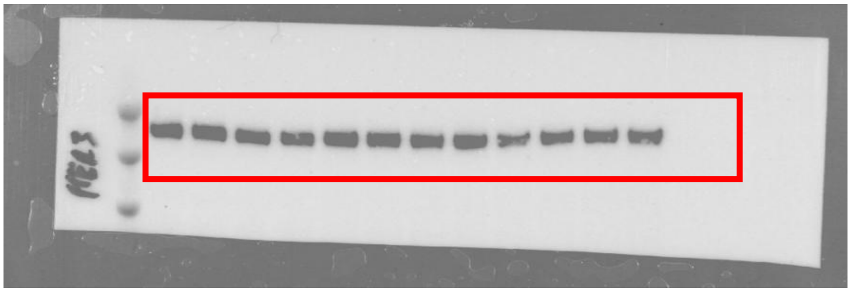

Full and Unedited Blots for Supplemental Figure 9B Continued

P-EGFR Y845

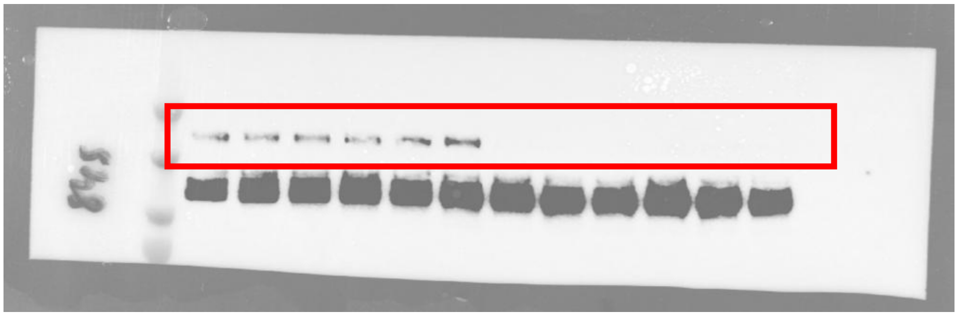

P-EGFR Y1173

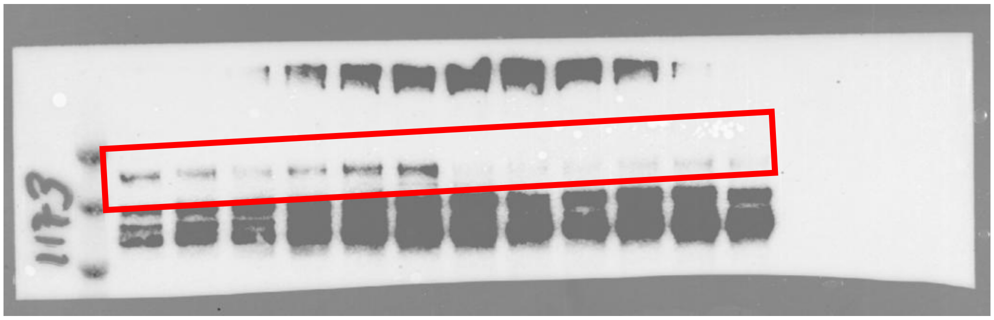

EGFR

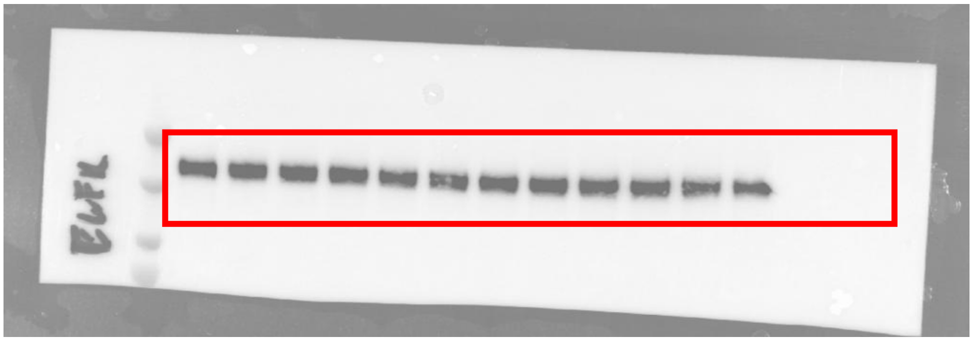

P-AKT

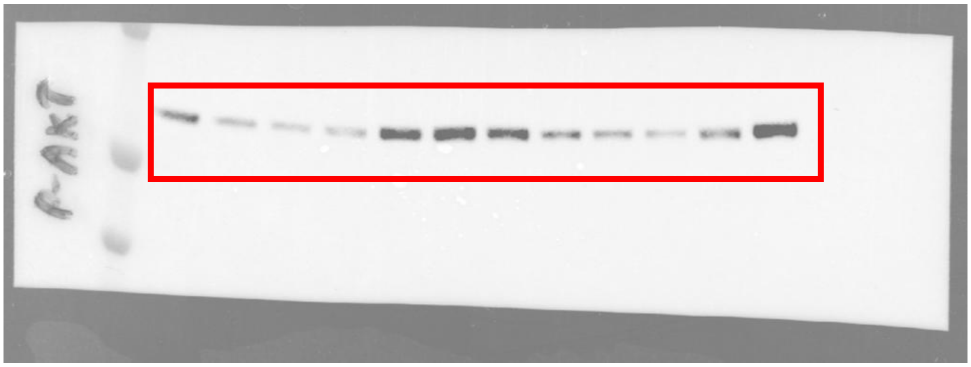

Full and Unedited Blots for Supplemental Figure 9B Continued

AKT

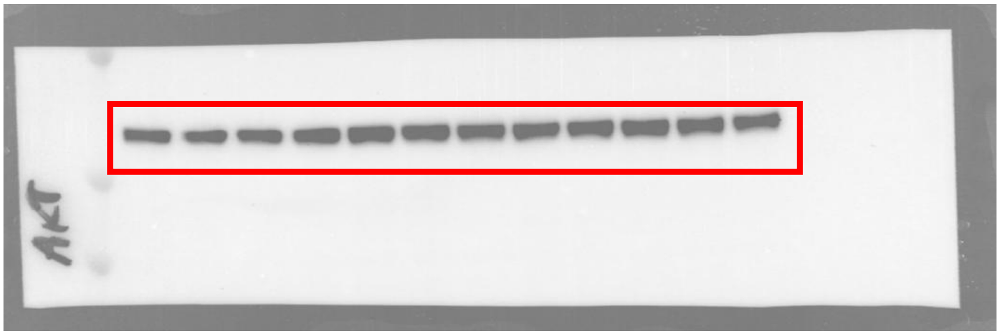

P-ERK

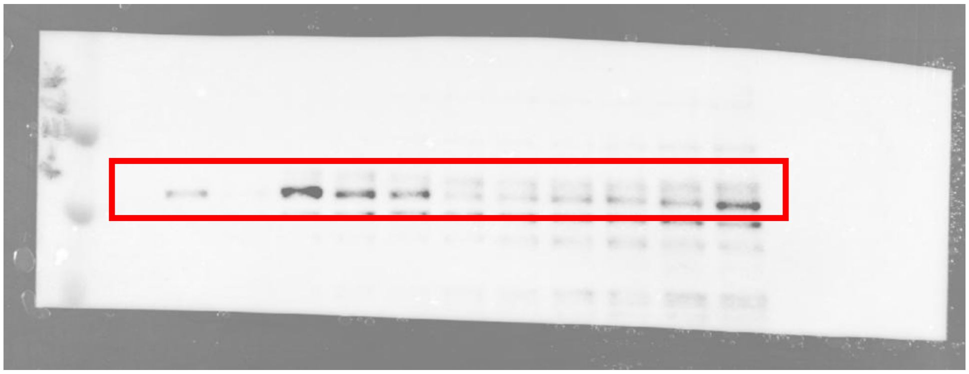

ERK

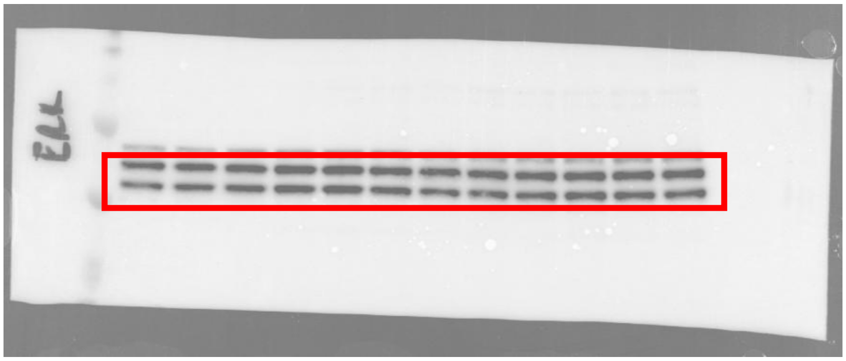

P-PLC

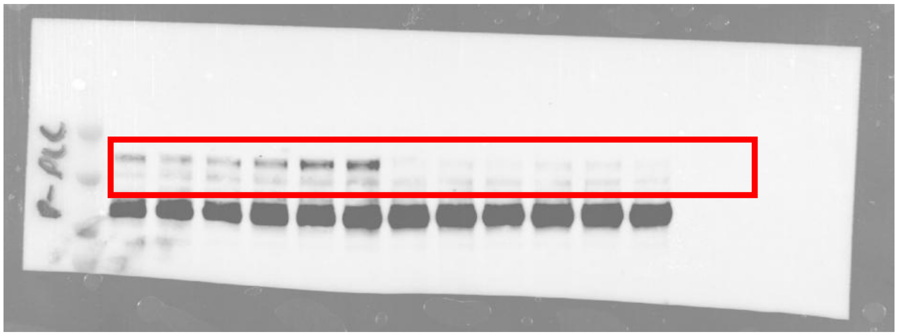

Full and Unedited Blots for Supplemental Figure 9B Continued

PLC

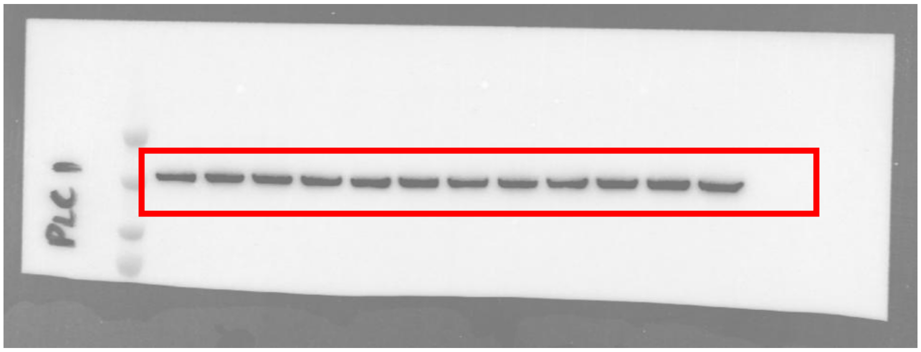

P-CAMKII

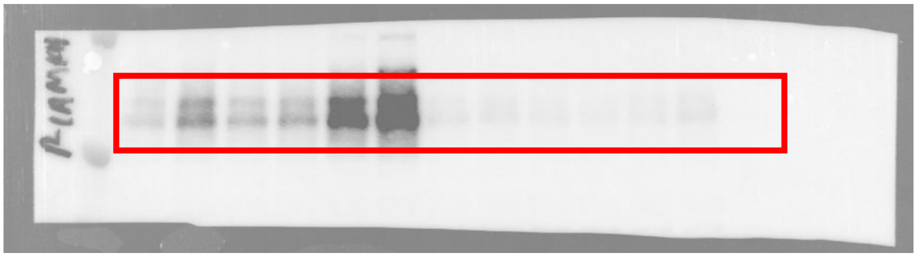

CAMKII

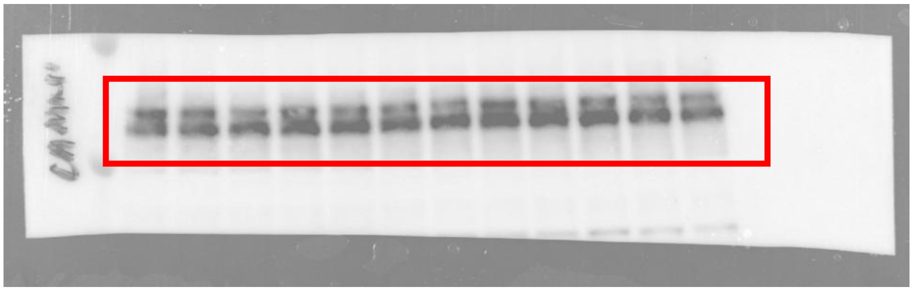

GAPDH

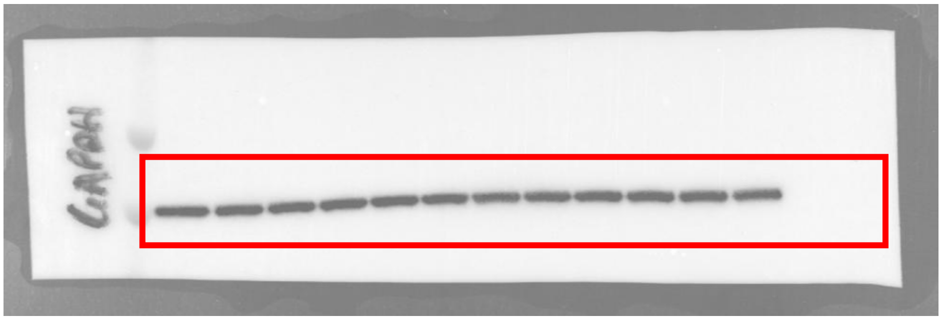

Supplement: Unedited blot and gel images [file jciinsight-10-187151-s058.pdf]
